# Amoebal Endosymbiont *Neochlamydia* Genome Sequence Illuminates the Bacterial Role in the Defense of the Host Amoebae against *Legionella pneumophila*

**DOI:** 10.1371/journal.pone.0095166

**Published:** 2014-04-18

**Authors:** Kasumi Ishida, Tsuyoshi Sekizuka, Kyoko Hayashida, Junji Matsuo, Fumihiko Takeuchi, Makoto Kuroda, Shinji Nakamura, Tomohiro Yamazaki, Mitsutaka Yoshida, Kaori Takahashi, Hiroki Nagai, Chihiro Sugimoto, Hiroyuki Yamaguchi

**Affiliations:** 1 Department of Medical Laboratory Science, Faculty of Health Sciences, Hokkaido University, Sapporo, Hokkaido, Japan; 2 Pathogen Genomics Center, National Institute of Infectious Diseases, Tokyo, Japan; 3 Research Center for Zoonosis Control, Hokkaido University, Sapporo, Hokkaido, Japan; 4 Division of Biomedical Imaging Research, Juntendo University Graduate School of Medicine, Tokyo, Japan; 5 Division of Ultrastructural Research, Juntendo University Graduate School of Medicine, Tokyo, Japan; 6 Research Institute for Microbial Diseases, Osaka University, Osaka, Japan; University of Vienna, Austria

## Abstract

Previous work has shown that the obligate intracellular amoebal endosymbiont *Neochlamydia* S13, an environmental chlamydia strain, has an amoebal infection rate of 100%, but does not cause amoebal lysis and lacks transferability to other host amoebae. The underlying mechanism for these observations remains unknown. In this study, we found that the host amoeba could completely evade *Legionella* infection. The draft genome sequence of *Neochlamydia* S13 revealed several defects in essential metabolic pathways, as well as unique molecules with leucine-rich repeats (LRRs) and ankyrin domains, responsible for protein-protein interaction. *Neochlamydia* S13 lacked an intact tricarboxylic acid cycle and had an incomplete respiratory chain. ADP/ATP translocases, ATP-binding cassette transporters, and secretion systems (types II and III) were well conserved, but no type IV secretion system was found. The number of outer membrane proteins (OmcB, PomS, 76-kDa protein, and OmpW) was limited. Interestingly, genes predicting unique proteins with LRRs (30 genes) or ankyrin domains (one gene) were identified. Furthermore, 33 transposases were found, possibly explaining the drastic genome modification. Taken together, the genomic features of *Neochlamydia* S13 explain the intimate interaction with the host amoeba to compensate for bacterial metabolic defects, and illuminate the role of the endosymbiont in the defense of the host amoebae against *Legionella* infection.

## Introduction

Obligate intracellular chlamydiae have evolved into two groups since the divergence of ancient chlamydiae 0.7–1.4 billion years ago. Pathogenic chlamydiae species (e.g. *Chlamydia trachomatis*) have adapted with their vertebrate hosts, whereas environmental chlamydiae (e.g. *Neochlamydia* species) have evolved as endosymbionts of lower eukaryotes, such as free-living amoebae (*Acanthamoeba*) [1]–[4]. Both types of chlamydiae have unique intracellular developmental cycles, defined by two distinct stages: the elementary body (EB), which is the form that is infectious to host cells, and the reticulate body (RB), which is the replicative form in the cells [4]. Interestingly, pathogenic chlamydiae have evolved through a decrease in genome size, with genomes of approximately 1.0–1.2 Mb, which may be a strategy to evade the host immune network, resulting in a shift to parasitic energy and metabolic requirements [1]–[4]. Meanwhile, the genome of the representative environmental chlamydia, *Protochlamydia* UWE25, is not decreasing and has stabilized at 2.4 Mb [4], implying that environmental chlamydiae still possess certain genes that pathogenic chlamydiae have lost. Therefore, environmental chlamydiae are useful tools for elucidating chlamydial evolution and obligate intracellular parasitism.

Recently, we isolated several environmental amoebae harboring endosymbiotic environmental chlamydiae from Sapporo, Hokkaido, Japan [5]. Of these, the amoebal endosymbiont *Neochlamydia* S13 was particularly interesting because its rate of amoebal infection was always 100%, but no amoebal lysis or transfer to other host amoebae was observed [5], [6]. This suggested an intimate mutualistic interaction of *Neochlamydia* S13 with its host amoebae, which is possibly a unique genomic feature. The reason why amoebae continually feed the endosymbiotic bacteria remains unknown, although the endosymbiotic bacteria may protect the host against *Legionella*, which also grow in and kill amoebae [7]–[9]. Therefore, in this study we evaluated the interaction of *Neochlamydia* S13 with the host amoebae, including its protective role against *Legionella*, through analysis of a draft genome of *Neochlamydia* S13.

## Results and Discussion

### *Neochlamydia* S13 intimately interacts with host amoebae and plays a significant role in the amoebal protection system against *Legionella pneumophila* infection

**Figure 1:**
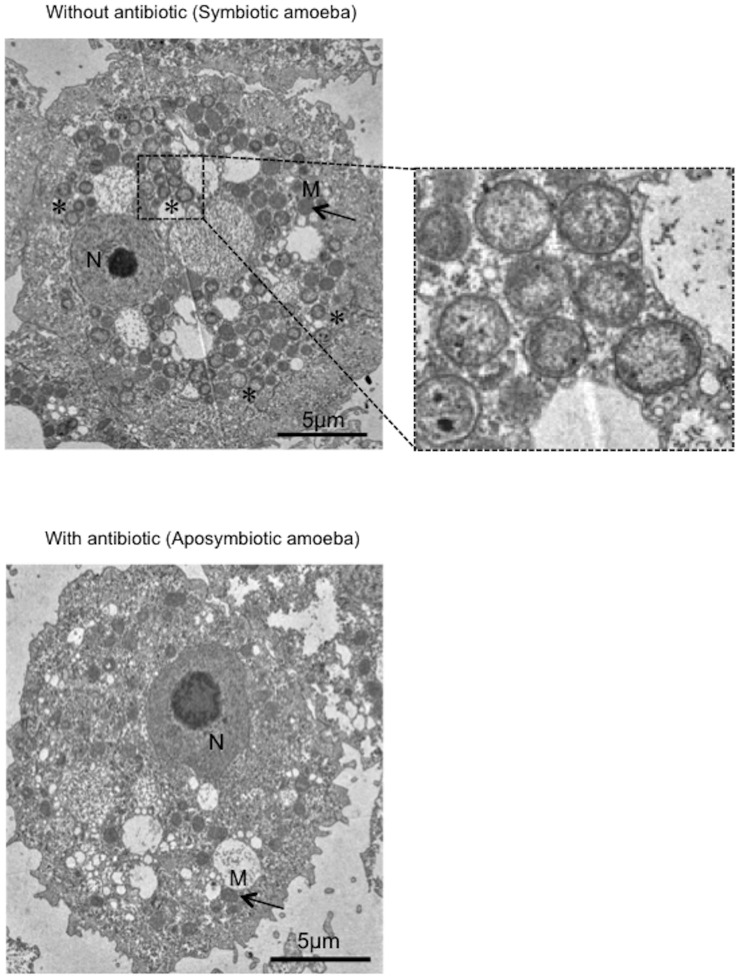
Morphological traits of *Neochlamydia* S13 inside host amoebae. Representative TEM images showing symbiotic amoebae harboring *Neochlamydia* S13 (up and right (enlarged)) and aposymbiotic amoebae constructed by treatment with antibiotics (down). Enlarged image in the square with a dotted line shows the bacterial reticular body (no elementary body was observed in the amoebae). *, bacteria. M, mitochondria (arrows). N, nucleus.

Transmission electron microscopic (TEM) analysis revealed a wide distribution of RBs in the amoebal cytoplasm, but no EBs were observed, suggesting persistent infection and an intimate interaction between the bacteria and the host amoeba (Figure 1).

**Figure 2:**
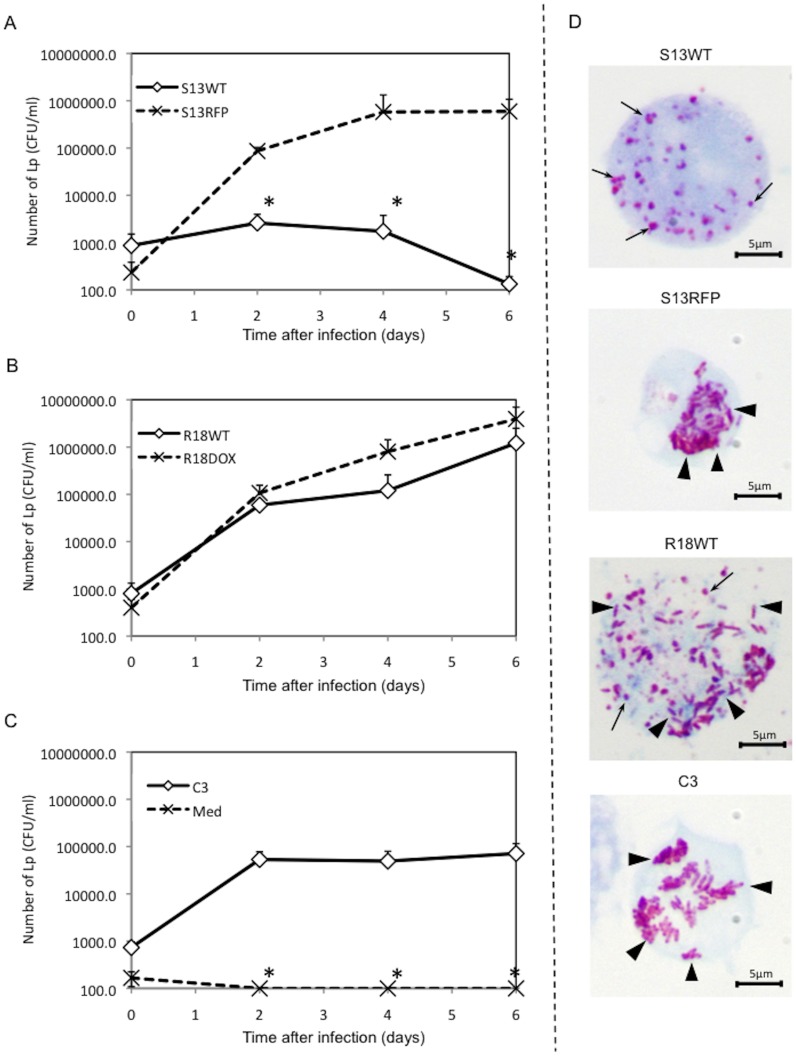
Amoebae harboring *Neochlamydia* S13 are completely protected from *L. pneumophila* infection. (A) *L. pneumophila* survival in *Neochlamydia* S13-infected amoebae (S13WT) and aposymbiotic amoebae (S13RFP). (B) *L. pneumophila* growth in *Protochlamydia* R18-infected amoebae (R18WT) and aposymbiotic amoebae (R18DOX). (C) *L. pneumophila* survival in C3 reference strain amoebae (C3), and PYG medium only (Med). Data are the means ± SD from at least three independent experiments performed in triplicate. * denotes *p*<0.05 at each of the time points. (D) Representative Gimenez staining images showing *L. pneumophila* replication into amoebae at 5 days post-infection. Arrows, *Neochlamydia* S13 (S13WT) or *Protochlamydia* R18 (R18WT). Arrowheads, replicated *L. pneumophila*.

Why the amoebae allow *Neochlamydia* to persist within the cells remains unknown. We therefore assessed whether the amoebae harboring *Neochlamydia* S13 could resist infection by *L. pneumophila*, which can kill amoebae in natural environments [7]–[9]. In contrast to the extensive growth observed in the aposymbiotic strain of amoeba (S13RFP: treatment with rifampicin), *L. pneumophila* failed to replicate in amoebae harboring *Neochlamydia* S13 wild-type (WT) (Figure 2A). Another amoebal strain, harboring *Protochlamydia* R18 (R18WT) [5], also allowed intracellular growth of *L. pneumophila*, as did the aposymbiotic amoeba R18DOX (treatment with doxycycline) and the reference C3 amoebal strain, which lacks any endosymbiotic bacteria (Figure 2B and C). Gimenez staining showed that *L. pneumophila* failed to grow in amoebae harboring *Neochlamydia* S13 (Figure 2D, top (arrows, *Neochlamydia* S13)). Thus, the results strongly suggested that *Neochlamydia* S13 confers a survival advantage on the host by providing resistance to *L. pneumophila* infection in amoebal environments such as biofilms [10], [11].

Phylogenetic analysis of 16S rRNA sequences showed that *Neochlamydia* S13 belonged to its own cluster, sequestered from other chlamydiae (Figure S1, arrow). Thus, the phylogenetic data suggest unique genomic features in *Neochlamydia* S13.

### General genome features and validity

A draft genome of *Neochlamydia* S13 was determined by Illumina GAIIx, assembled with ABySS-pe, and then annotated by the RAST server with manual local BLAST analysis. The draft genome of *Neochlamydia* S13 contained 3,206,086 bp (total contig size) with a GC content of 38.2% in 1,317 scaffold contigs (DDBJ accession number: BASK01000001–BASK01001342). The genome contains 2,832 protein-coding sequences (CDSs), 43 tRNAs, and six ribosomal RNAs (Table S1). About half of the CDSs (1,577; 57%) were classified as hypothetical proteins, including 1,030 unknown proteins (36.4%) that had no significant similarity to those of other chlamydiae (*Protochlamydia amoebophila* UWE25 (NC_005861.1), *Parachlamydia acanthamoebae* UV-7 (NC_015702.1), *Simkania negevensis* Z (NC_015713.1), *Waddlia chondrophila* WSU 86-1044 (NC_014225.1), *Chlamydia trachomatis* D/UW-3/CX (NC_000117.1), *C. trachomatis* L2 434/Bu (NC_010287.1), *Chlamydia pneumoniae* CWL029 (NC_000922.1), *C. pneumoniae* TW183 (NC_005043.1)) in public databases (E-value >1e^−10^) (Figure S2). This suggested that *Neochlamydia* S13 contains unique genomic features, which may provide hints for discovering the intimate mutual relationship underlying symbiosis.

Because of the presence of so many unknown genes with repeat sequences in the predicted genome, we were unable to fill all of the gaps to complete the genome of *Neochlamydia* S13. The validity of our system, including scaffold contig assembly and gene annotation, was confirmed by comparing the reference genome of *Protochlamydia* UWE25 (NC_005861.1) with the draft genome of its related amoebal endosymbiont, *Protochlamydia* R18 (originally isolated from a river in Sapporo City, Japan [5]), assembled using our system for this study. The draft genome of *Protochlamydia* R18 contained 2,727,392 bp with a GC content of 38.8% in 770 scaffold contigs (DDBJ accession number: BASL01000001–BASL01000795). An ORF annotation coverage of 87.6% was observed.

### Glycolytic pathway, tricarboxylic acid cycle, and respiratory chain are incomplete

**Figure 3:**
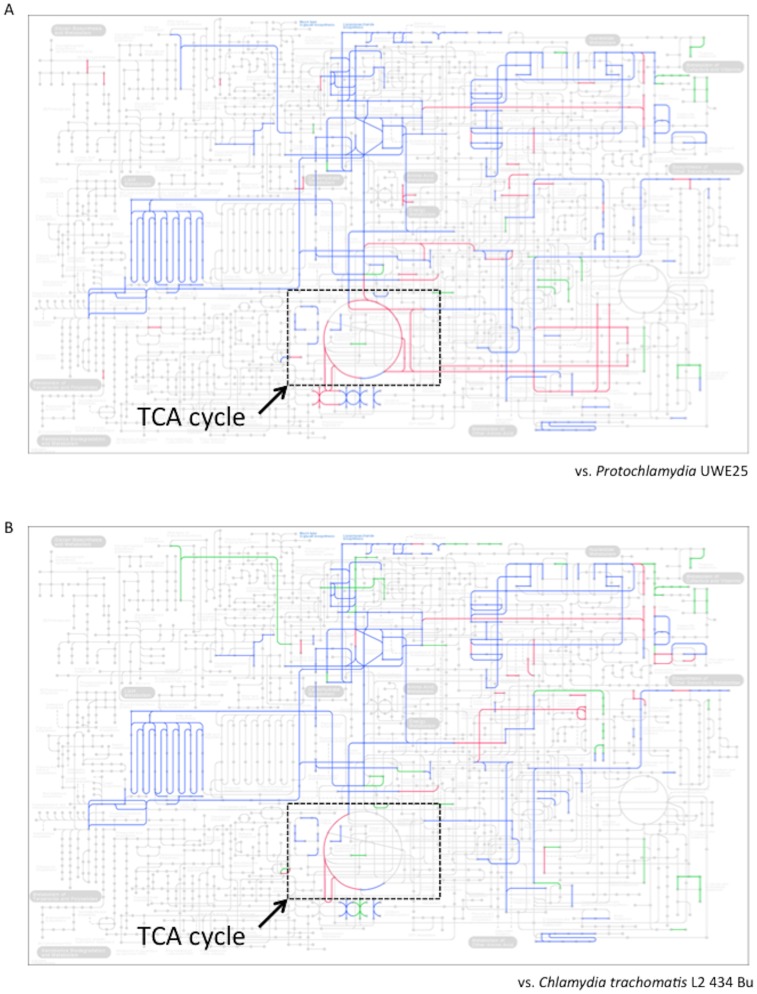
Comparison of metabolic pathways among representative chlamydiae. (A) *Neochlamydia* S13 (this study) versus *Protochlamydia amoebophila* UWE25 (NC005861.1). (B) *Neochlamydia* S13 versus *C. trachomatis* L2 434 Bu (NC010287.1). Green lines, unique in the *Neochlamydia* active modules. Blue lines, shared modules. Red lines; modules specific for *Protochlamydia* or *C. trachomatis*. Squares surrounded with dotted line show TCA cycle.

As mentioned above, the previous findings of an amoebal infection rate of 100% and absence of amoebal lysis and transferability to other host amoebae suggest a defective energy reserve system in *Neochlamydia* S13. We therefore used KEGG-module analysis to determine whether *Neochlamydia* S13 contained complete metabolic pathways. This showed that the *Neochlamydia* S13 modules that mapped onto the metabolic pathways differed significantly from those of other chlamydiae (*Protochlamydia* UWE25 and *C. trachomatis* L2) (Figure 3). While the glycolytic pathway from fructose 1,6-bisphosphate to pyruvate was complete in *Neochlamydia* S13, hexokinase and 6-phosphfructokinase were missing, indicating a truncated Embden-Meyerhof-Parnas pathway (Figure S3). Analysis also confirmed that while the Entner-Doudoroff pathway was truncated, the pentose phosphate pathway was intact, suggesting ribulose-5-phosphate synthesis with folate metabolic activity (Figure S3). Surprisingly, the tricarboxylic acid (TCA) cycle, which oxidizes acetyl-CoA to CO_2_, was almost entirely missing, except for malate dehydrogenase, the dihydrolipoamide succinyltransferase component (E2) of the 2-oxoglutarate dehydrogenase complex, and dihydrolipoamide dehydrogenase of 2-oxoglutarate dehydrogenase (Figure S3). As pathogenic chlamydiae are still viable when carrying at least half of the TCA cycle genes [2], [12], and all previously reported environmental chlamydiae possess complete TCA cycles [4], [13], [14], the lack of the cycle in the *Neochlamydia* S13 genome is unique. We also found a defective respiratory chain, equipped with only the NADH dehydrogenase complex, cytochrome *c* oxidase complex, and V-type ATPase units; the succinate dehydrogenase complex, cytochrome *c* reductase complex, and F-type ATPase units were completely missing (Figure S4). Thus, these data show that the central metabolic pathway of *Neochlamydia* S13 is drastically truncated, even when compared with pathogenic chlamydiae, indicating a strong dependence on host amoebae.

### Fatty acid biosynthesis pathways are conserved

As the genes of the fatty acid initiation and biosynthesis pathways were conserved, as in other chlamydiae (Figure S5), it seemed likely that *Neochlamydia* S13 could produce fatty acid. However, synthesis pathways for CoA, which is a starting material for the acetyl-CoA required for fatty acid initiation [15], [16], and biotin, which is a coenzyme required for fatty acid construction [17], [18] were lacking in *Neochlamydia* S13. These findings therefore suggest that both CoA and biotin may be transported from the amoebal cytoplasm into the bacteria by unknown transporters.

### ATP/ADP translocases, ATP-binding cassette (ABC) transporters, the Sec-dependent type II secretion system, and the type III secretion systems, but not the type IV secretion system, are well conserved

Similar to pathogenic chlamydiae, *Neochlamydia* S13 lacked many key enzymes in the purine and pyrimidine metabolic pathways that are directly connected to nucleotide biosynthesis. It therefore seemed likely that *Neochlamydia* S13 might obtain ATP from the host amoebal cytoplasm via a number of ATP/ADP translocases. As expected, three translocases (NTT1–NTT3) similar to those of pathogenic chlamydiae (Figure S6A) were identified in the genome, although environmental chlamydia strain UWE25 contains five ATP/ADP translocases [19], [20]. We also found several ABC transport systems (spermidine/putrescine, zinc, mannan, lipopolysaccharide, and lipoprotein) in the annotated genome (Figure S6B). These findings suggest significant roles for the ABC transporters in compensating for the defective metabolic systems of the bacteria, possibly explaining the intimate symbiotic interaction and strong host dependency. Meanwhile, the number of ABC transporters identified in the draft genome was limited, although these transporters are generally widespread among living organisms and are highly conserved in all genera. They are responsible for essential biological processes such as material transport, translation elongation, and DNA repair [21]–[23].

We next assessed whether secretion systems (Sec-dependent type II, type III, and type IV) were conserved in the *Neochlamydia* S13 genome. As shown in Figure S7, the type III secretion system, which is widely distributed among chlamydiae [24]–[26], was well conserved in the *Neochlamydia* S13 genome, and the Sec-dependent type II secretion system was nearly completely conserved (Figure S8). These findings suggested that both systems aid *Neochlamydia* S13 survival in the host amoebae. Interestingly, in contrast to previously reported environmental chlamydiae [4], [13], [27], [28], no gene cluster encoding the type IV secretion system was found, similar to pathogenic chlamydiae, although the *Protochlamydia* R18 genome contained a complete type IV gene cluster (Figure S9, *Protochlamydia* UWE25 versus *Protochlamydia* R18). Recent works have strongly suggested that bacterial type IV secretion systems might induce inflammasome or caspase activation, resulting in bacterial elimination via accumulation of professional effector cells [29]–[31]. It is therefore possible that the type IV secretion system is harmful to the symbiotic interaction in host cells, as well as persistent infection that generally occurs in mammalian cells.

### Predicted outer membrane proteins were truncated

**Figure 4:**
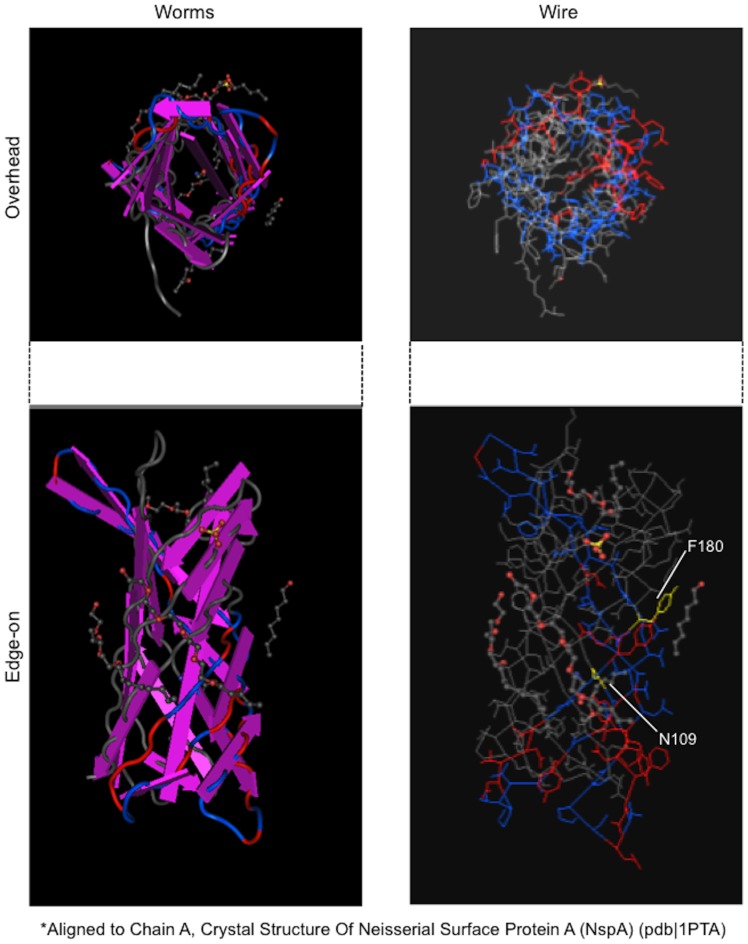
Predicted three-dimensional structure of PomS (NEOS13_1146), which presumably localizes to the outer membrane as a porin. The structure was constructed by alignment with the crystal structure of chain A of *Neisseria* surface protein A (NspA) (pdb:1PTA). N109 (yellow), amino acid aligned at start position. F180 (yellow), amino acid aligned at end position.

In contrast to *Protochlamydia* UWE25 [13], [32], [33], *Neochlamydia* S13 contained fewer annotated genes encoding outer membrane proteins, which presumably localize to the outer leaflet membrane and periplasmic space. These genes, *pomS*, the 76-kDa protein gene (*Protochlamydia* UWE25, pc0004), *ompW*, and *omcB* (Figure S10), indicate successful adaptation to the host amoebal cytoplasm through loss of redundant molecules. The predicted 3D model of PomS (NEOS13_1146), constructed using MMDB (see “Methods”), showed a porin with a β-barrel structure and a channel (Figure 4), suggesting an active transporter.

### Predicted proteins with leucine-rich repeats (LRRs) or ankyrin domains

As mentioned above, we found that amoebae harboring *Neochlamydia* S13 were never infected with *L. pneumophila*, which is a natural killer of amoebae [7]–[9], [10], [11]. We hypothesized that *Neochlamydia* S13 effector molecules secreted into the amoeba might be associated with protection against *L. pneumophila* infection. Recent studies have intriguingly revealed that pathogenic bacteria have evolved effector proteins with LRR or ankyrin domains that may mimic host signaling molecules when injected into host cells [34]–[36]. Therefore, we searched for unique molecules with LRRs [37], [38] or ankyrin domains [39]–[41], which may be responsible for protein-protein interaction and possibly for controlling *L. pneumophila* infection in host amoebae, in the *Neochlamydia* S13 genome. We identified 199 genes encoding predicted candidate molecules with LRRs, 30 of which were unique, showing no homology with other environmental chlamydiae (Table S2). This suggests possible expansion of these genes from a small number of ancestral genes containing LRRs, although the mechanism of expansion remains unknown. However, it is possible that *L. pneumophila* infection could stimulate the expansion of the *Neochlamydia* genes encoding LRR domains. Among these genes, 15 were well conserved with those of *Micromonas* (algae) and *Nostoc punctiforme* (a nitrogen-fixing cyanobacterium), with 45–74% identity (Table S2). These results suggest horizontal gene transfer between *Neochlamydia* S13 and such plant-related microbes, allowing us to hypothesize an ancestral relationship between chlamydiae and algae or cyanobacteria [42]–[44].

As it is well known that molecules with ankyrin domains play a critical role in protein-protein interaction, we also searched for these genes in the *Neochlamydia* S13 genome. RAST analysis with manual local BLAST analysis predicted eight genes (NEOS13_0151, NEOS13_0209, NEOS13_0435, NEOS13_0856, NEOS13_1517, NEOS13_1563, NEOS13_2364, NEOS13_2796) that encode molecules with ankyrin domains. Interestingly, NEOS13_0151 had a unique coiled-coil structure that was not similar to other chlamydial proteins (Table S1, Figure S11). Meanwhile, phylogenetic analysis of NEOS13_0151 revealed close similarity with functional molecules found in eukaryotes (Figure S12), presumably associated with host cell modification or cellular functions. Recent work has shown that the *L. pneumophila* (strain AA100/130b) F-box ankyrin effector is involved in eukaryotic host cell exploitation, allowing intracellular growth [45]. Thus, we suggest that *Neochlamydia* S13 possesses unique genes encoding ankyrin domains, possibly responsible for resisting *L. pneumophila* infection via host amoebae, although the underlying mechanism remains to be determined.

### Presence of transposases implies drastic genome modification

We found 33 genes encoding transposases in the *Neochlamydia* S13 genome, as annotated by RAST analysis with manual local BLAST analysis (Table S1). It has been reported that *Chlamydia suis* possesses a novel insertion element (IScs605) encoding two predicted transposases [46], and that *Protochlamydia* UWE25 contains 82 transposases [47]. Thus, the features of the *Neochlamydia* S13 genome were unique, without genome reduction, but with specified genes for controlling host-parasite interaction, resulting in successful adaptation to the host amoeba. Although the reason why the *Neochlamydia* S13 genome size has not reduced remains unknown, such transposases may be responsible for genome modification without genome reduction.

## Conclusions

We determined a draft genome sequence of *Neochlamydia* S13, which provided hints as to why the mutualistic interaction between the bacteria and the host amoebae is maintained, and how the bacteria manipulate the host amoebae. Such unique genome features of *Neochlamydia* S13 strongly indicate an intimate dependency on the host amoebae to compensate for lost bacterial metabolic activity, and a possible role for the bacterial endosymbiont in defense against *L. pneumophila*. These findings provide new insight into not only the extraordinary diversity between chlamydiae, but also why symbiosis occurred between the amoebae and environmental chlamydiae.

## Materials and Methods

### Amoebae

As described previously [5], two different amoebal (*Acanthamoeba*) strains persistently infected with *Protochlamydia* R18 or *Neochlamydia* S13 were isolated from a river water sample and a soil sample, respectively. The prevalence of amoebae with endosymbionts, as defined by 4′,6-diamidino-2-phenylindole staining, was always approximately 100% [6]. *Acanthamoeba castellanii* C3 (ATCC 50739) was purchased from the American Type Culture Collection (ATCC) and used as a reference strain. Aposymbiotic amoebae derived from *Protochlamydia* R18-infected amoebae (designated R18DOX, established by treatment with doxycycline (64 µg/ml)) and *Neochlamydia* S13-infected amoebae (designated S13RFP, established by treatment with rifampicin (64 µg/ml)) [6] were also used for this study. All amoebae were maintained in PYG broth (0.75% (w/v) peptone, 0.75% (w/v) yeast extract, and 1.5% (w/v) glucose) at 30°C [5].

### Bacteria

Human isolate *L. pneumophila* Philadelphia I (JR32), equipped with a complete *dot/icm* gene set encoding a type IV secretion system, which is required for intracellular amoebal growth [48], was kindly provided by Dr. Masaki Miyake of the University of Shizuoka, Japan. *L. pneumophila* was cultured on BCYE agar (OXOID, Hampshire, UK) at 37°C for 2 days.

### Infection of amoebae with *L. pneumophila*

Amoebae (5×10^5^ cells) were infected with *L. pneumophila* (5×10^5^ colony-forming units [CFU]) at a multiplicity of infection of one for 2 h at 30°C, and then uninfected bacteria were killed by the addition of 50 µg/ml gentamycin. After washing with PYG medium, the infected amoebae were incubated for up to 6 days. The infected amoebae were collected every other day, and bacterial CFUs were estimated by serial dilution on BCYE agar.

### Bacterial purification and genomic DNA extraction

Both *Neochlamydia* S13- and *Protochlamydia* R18-infected amoebae were collected by centrifugation at 1,500× *g* for 30 min. The resulting pellets were suspended in PYG medium. Each of the amoebae were disrupted by bead-beating for 5 min according to a previously described method [49], and then centrifuged at 150× *g* for 5 min to remove unbroken cells and nuclei. The supernatant including intact bacteria was incubated with DNase (Sigma) for 30 min at room temperature, and then the bacteria were washed and suspended in 10 mM HEPES buffer containing 145 mM NaCl. The suspension was carefully overlayed onto 30% Percoll. Following centrifugation at 30,000× *g* for 30 min, the bacteria were collected from the lower layer. Finally, bacterial pellets were stored at −20°C until use. DNA was extracted with a phenol-chloroform method.

### Genome sequencing, annotation and prediction of metabolic pathway modules, and genome comparison

Bacterial 1 kb insert DNA libraries (Purified *Neochlamydia* S13 and *Protochlamydia* R18) were prepared using a genomic DNA Sample Prep Kit (Illumina, San Diego, CA). DNA clusters were generated on a slide using a Cluster Generation Kit (ver. 4) on an Illumina Cluster Station, according to the manufacturer's instructions. Sequencing runs for 81-mer paired-end sequence were performed using an Illumina Genome Analyzer IIx (GA IIx). The 81-mer paired-end reads were assembled (parameters k64, n51, c32.1373) using ABySS-pe v1.2.0 [50]. Annotation of genes from the draft genome sequences was performed using Rapid Annotation using Subsystem Technology (RAST: http://rast.nmpdr.org/) [51] with a local manual BLASTp search. Metabolic pathway modules were predicted using the Kyoto Encyclopedia of Genes and Genomes (KEGG: http://www.genome.jp/kegg/) [52]. Genome comparison was performed using RAST, and then manually visualized by GenomeMatcher 1.69 (http://www.ige.tohoku.ac.jp/joho/gmProject/gmhome.html) [53].

### Prediction of 3D structure for annotated genes

Three-dimensional structures of annotated protein sequences of interest were predicted using a web program, protein BLAST with the Molecular Modeling Database (MMDB) (http://www.ncbi.nlm.nih.gov/Structure/MMDB/mmdb.shtml) [54]. Cn3D 4.3 (http://www.ncbi.nlm.nih.gov/Structure/CN3D/cn3dmac.shtml) was used to display the predicted structure [55].

### Phylogenetic analysis

Phylogenetic analyses of all nucleotide sequences were conducted using the neighbor-joining method with 1,000 bootstrap replicates in ClustalW2 (http://blast.ncbi.nlm.nih.gov/Blast.cgi) [55]. The website viewer was also used to display the generated tree for Figure S12. Other tree in supplementary figure (Figure S1) was visualized using TreeViewX (version 0.5.0) [56].

### TEM

For TEM analysis, amoebal cells were immersed in a fixative containing 3% glutaraldehyde in 0.1 M phosphate buffered saline (PBS), pH 7.4, for 24 h at 4°C. Following a brief wash with PBS, cells were processed by alcohol dehydration and embedding in Epon 813. Ultrathin cell sections were stained with lead citrate and uranium acetate prior to visualization by electron microscopy (Hitachi H7100; Hitachi, Tokyo, Japan) as described previously [57].

### Statistical analysis

Data were compared using a Student's *t*-test. A *P*-value of less than 0.05 was considered significant.

### Contig sequence accession numbers

The draft genome sequences for the *Neochlamydia* S13 and *Protochlamydia* R18 strains have been deposited in the DNA Data Bank of Japan [DDBJ accession numbers: BASK01000001–BASK01001342 (*Neochlamydia* S13), BASL01000001–BASL010 00795 (*Protochlamydia* R18)].

## Supporting Information

Figure S1 **Phylogenetic analysis of chlamydial 16S rRNA sequences.** Bacterial names follow the accession numbers. Arrow denotes *Neochlamydia* 16S rRNA. The gene accession numbers are as follows: *Chlamydia trachomatis* D/UW_3/CX, NC_000117.1; *Chlamydia trachomatis* L2/434/Bu, NC_010287.1; *Chlamydia pneumoniae* TW-183, NC_005043.1; *Simkania* Z gsn131, NC_015713.1; *Parachlamydia acanthamoebae* UV7, NC_015702.1; endosymbiont *Acanthamoeba* UWC22, AF083616.1; endosymbiont *Acanthamoeba* TUME1, AF098330.1; *Neochlamydia hartmannellae*, AF177275.1; *Parachlamydia acanthamoebae* Bn_9_, NR_026357.1; *Parachlamydia* Hall's coccus, AF366365.1; *Parachlamydia acanthamoebae* Seine, DQ309029.1; *Protochlamydia naegleriophila* KNic, DQ632609.1.
(TIFF)

Figure S2 **Venn diagram showing the numbers of common and unique proteins among three chlamydiae.** Red, *Chlamydia trachomatis* D/UW_3/CX (NC000117.1). Green, *Protochlamydia* R18 (this study). Blue, *Neochlamydia* S13 (this study).
(TIFF)

Figure S3 **Predicted genes annotated as glycolytic pathway and TCA cycle with pentose phosphate pathway and Entner-Doudoroff pathway.** Black lines with arrows show predicted active modules. Gray lines show incomplete modules. Red names with numbers indicate *Neochlamydia* S13 gene IDs.
(TIFF)

Figure S4 **Predicted genes annotated as oxidative phosphorylation pathway.** Solid lines with arrows show predicted active modules. Gray lines show incomplete modules. Red names with numbers indicate *Neochlamydia* S13 gene IDs.
(TIFF)

Figure S5 **Predicted genes annotated as fatty acid initiation and elongation.** Black lines with arrows show predicted active modules. Red names with numbers indicate *Neochlamydia* S13 gene IDs.
(TIFF)

Figure S6 **Predicted genes annotated as ATP/ADP translocases (NTTs) (A) and ABC transporters (B).** Black lines with arrows show predicted active modules. Gray lines show incomplete modules. Red names with numbers indicate *Neochlamydia* S13 gene IDs.
(TIFF)

Figure S7 **Comparison of genes encoding a type III secretion system from** ***Neochlamydia*** **S13 and** ***Protochlamydia*** **UWE25.** The type III operon structures of the two chlamydiae are compared. Top panel, T3SS-T1; second panel, T3SS-A1; third panel, T3SS-A2; bottom panel, T3SS-A3. Right scale values show % identity estimated by BLASTp. Each of the gene cluster sequences in *Protochlamydia amoebophila* UWE25 (NC_005861.1) was obtained from NCBI (http:www.ncbi.nlm.nih.gov/genome).
(TIFF)

Figure S8 **Predicted genes annotated as Sec-dependent type II secretion machinery.** Black line with arrow shows predicted active module. Gray boxes show incomplete molecules. Red names with numbers indicate *Neochlamydia* S13 gene IDs.
(TIFF)

Figure S9 **Comparative analysis of genes encoding type IV secretion machinery from** ***Protochlamydia*** **UWE25 and** ***Protochlamydia*** **R18.** No annotated type IV genes were found in the *Neochlamydia* S13 genome. Blue boxes indicate individual coding regions of the type IV cluster.
(TIFF)

Figure S10 **Predicted outer membrane structures.** Blue molecules were predicted to be active. Gray molecules are absent. Red names with numbers indicate *Neochlamydia* S13 gene IDs. This figure depicts the predicted outer membrane structure based on a findings described by Heinz et al. [34] and previous findings published by Aistleitner et al. [33].
(TIFF)

Figure S11 **Characterization of a unique molecule with ankyrin domains (NEOS13_0151).** (A) Detection of ankyrin domains in the molecule encoded by NEOS13_0151. (B) Alignment scores and 3D prediction. The scores and prediction were performed using the web program, protein BLAST with the MMDB (http://www.ncbi.nlm.nih.gov/Structure/MMDB/docs/mmdb_search.html).
(TIFF)

Figure S12 **Phylogenetic comparison of the predicted protein sequence encoded by NEOS13_0151 with other eukaryotic proteins.** The predicted protein sequence encoded by NEOS13_0151 was phylogenetically compared with previously reported sequences obtained from the GenBank database using ClustalW2. The phylogenetic trees generated from the aligned sequences were constructed by neighbor-joining in ClustalW2, and then visualized with the website viewer.
(TIFF)

Table S1 ***Neochlamydia*** **S13 gene IDs with features.**
(PDF)

Table S2 **Homologs of eukaryote genes in the** ***Neochlamydia*** **S13 genome encoding predicted LRR-molecules.**
(PDF)
