# Supplementary figures and images for: Amoebal Endosymbiont Neochlamydia Genome Sequence Illuminates the Bacterial Role in the Defense of the Host Amoebae against Legionella pneumophila
Source: PLoS One. 2014 Apr 18;9(4):e95166. doi: 10.1371/journal.pone.0095166 (PMC3991601; doi:10.1371/journal.pone.0095166)

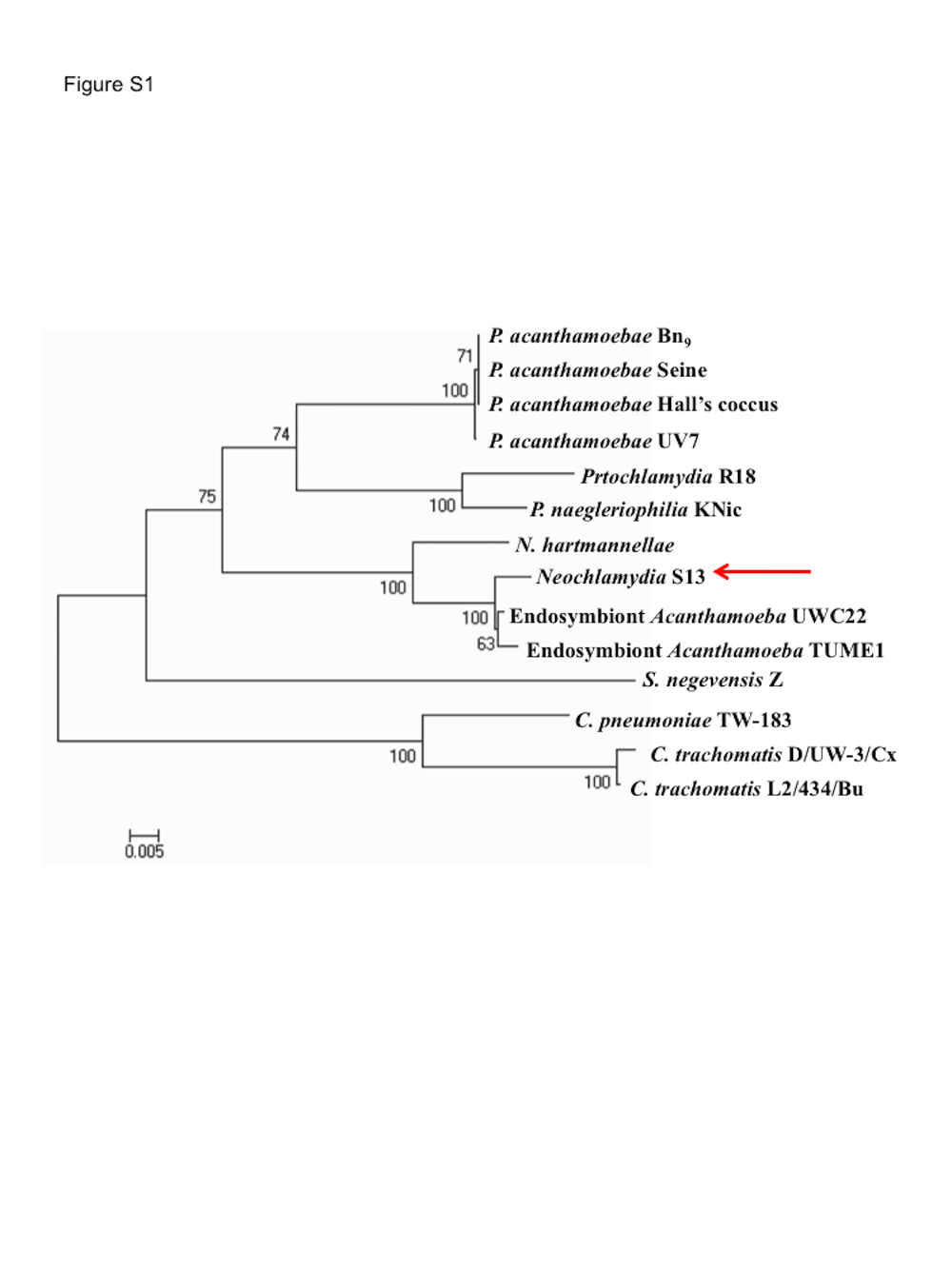

Supplement: Figure S1 — Phylogenetic analysis of chlamydial 16S rRNA sequences. Bacterial names follow the accession numbers. Arrow denotes Neochlamydia 16S rRNA. The gene accession numbers are as follows: Chlamydia trachomatis D/UW_3/CX, NC_000117.1; Chlamydia trachomatis L2/434/Bu, NC_010287.1; Chlamydia pneumoniae TW-183, NC_005043.1; Simkania Z gsn131, NC_015713.1; Parachlamydia acanthamoebae UV7, NC_015702.1; endosymbiont Acanthamoeba UWC22, AF083616.1; endosymbiont Acanthamoeba TUME1, AF098330.1; Neochlamydia hartmannellae, AF177275.1; Parachlamydia acanthamoebae Bn9, NR_026357.1; Parachlamydia Hall's coccus, AF366365.1; Parachlamydia acanthamoebae Seine, DQ309029.1; Protochlamydia naegleriophila KNic, DQ632609.1. (TIFF) [file pone.0095166.s001.tiff]

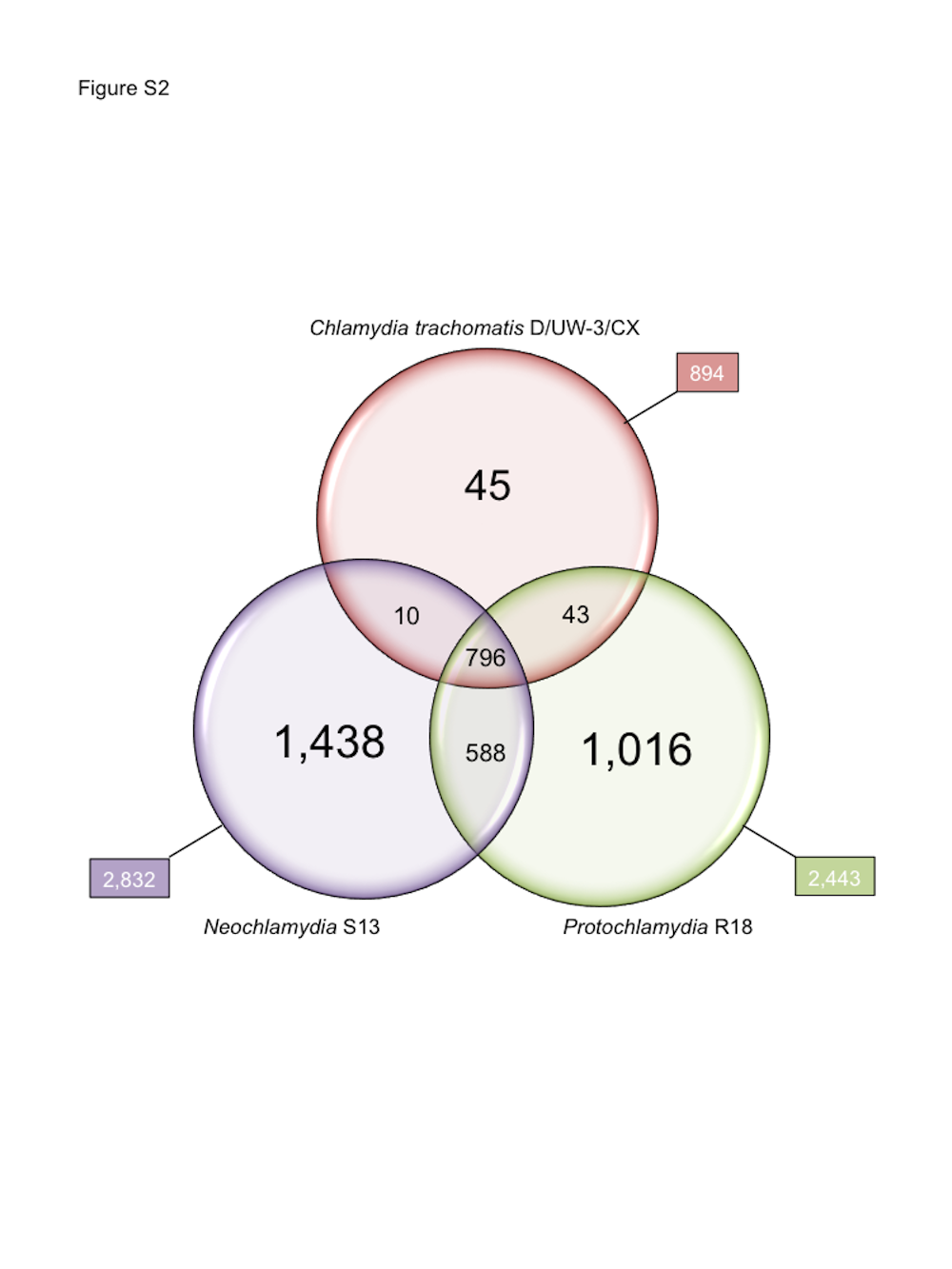

Supplement: Figure S2 — Venn diagram showing the numbers of common and unique proteins among three chlamydiae. Red, Chlamydia trachomatis D/UW_3/CX (NC000117.1). Green, Protochlamydia R18 (this study). Blue, Neochlamydia S13 (this study). (TIFF) [file pone.0095166.s002.tiff]

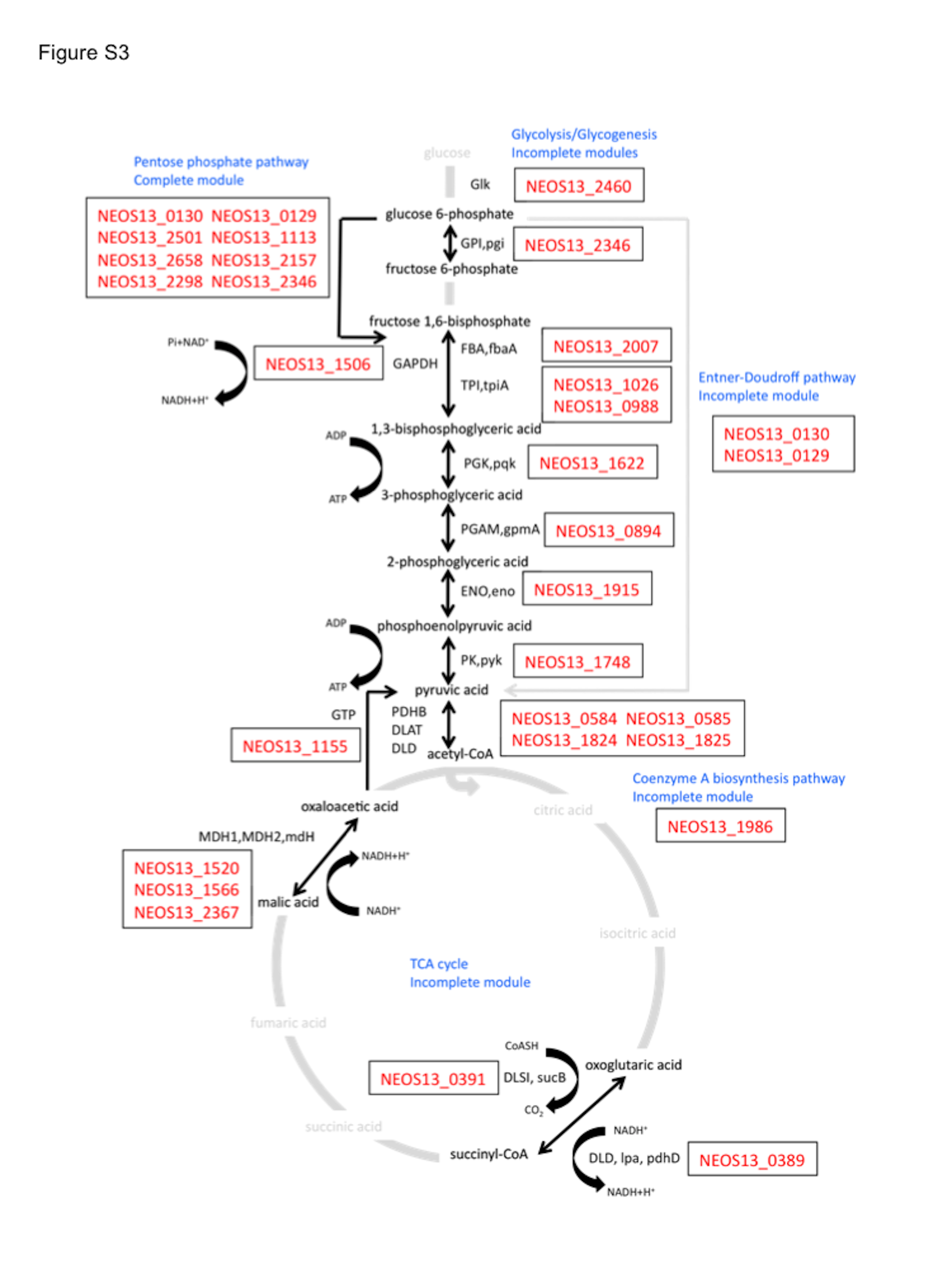

Supplement: Figure S3 — Predicted genes annotated as glycolytic pathway and TCA cycle with pentose phosphate pathway and Entner-Doudoroff pathway. Black lines with arrows show predicted active modules. Gray lines show incomplete modules. Red names with numbers indicate Neochlamydia S13 gene IDs. (TIFF) [file pone.0095166.s003.tiff]

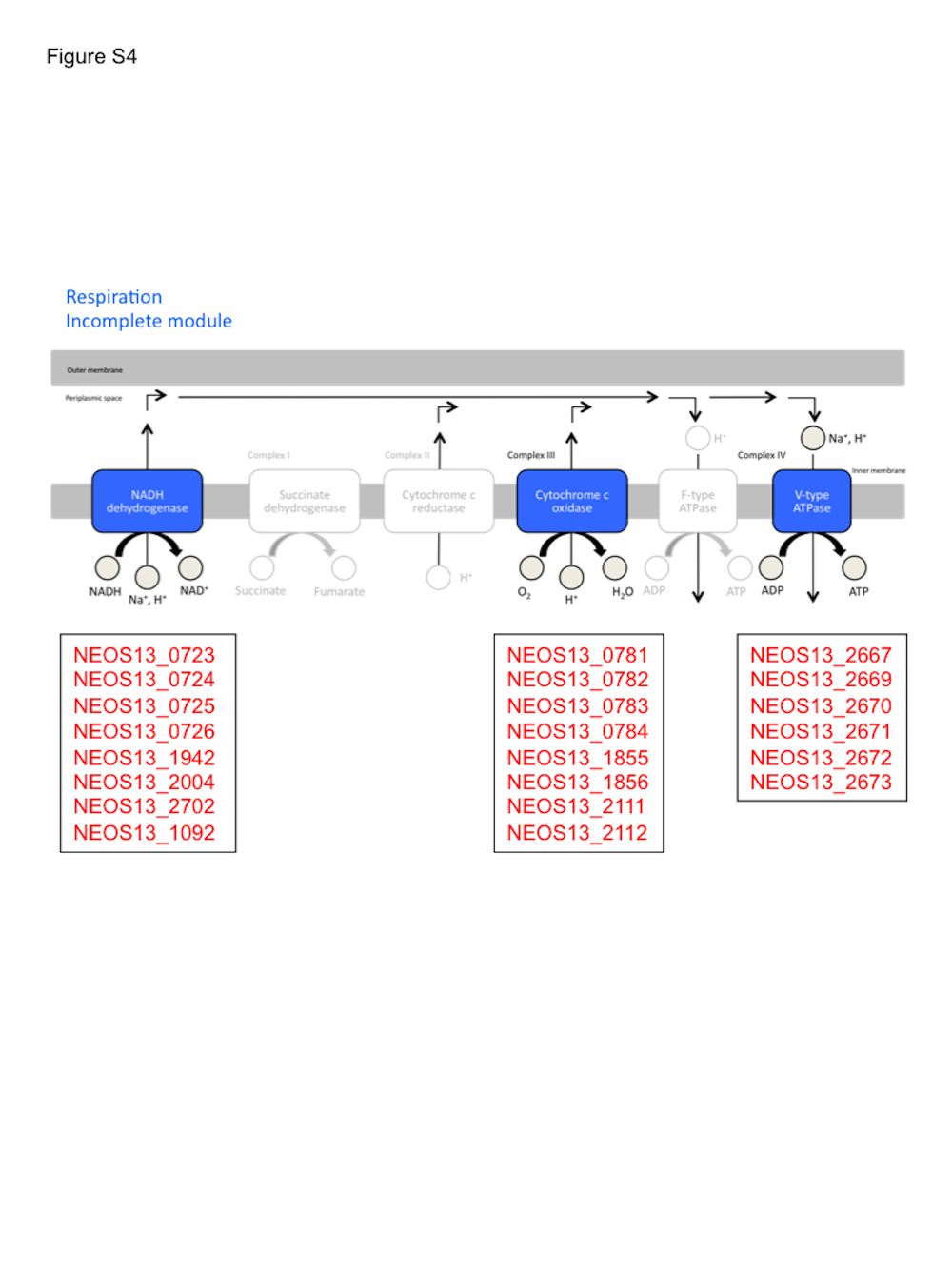

Supplement: Figure S4 — Predicted genes annotated as oxidative phosphorylation pathway. Solid lines with arrows show predicted active modules. Gray lines show incomplete modules. Red names with numbers indicate Neochlamydia S13 gene IDs. (TIFF) [file pone.0095166.s004.tiff]

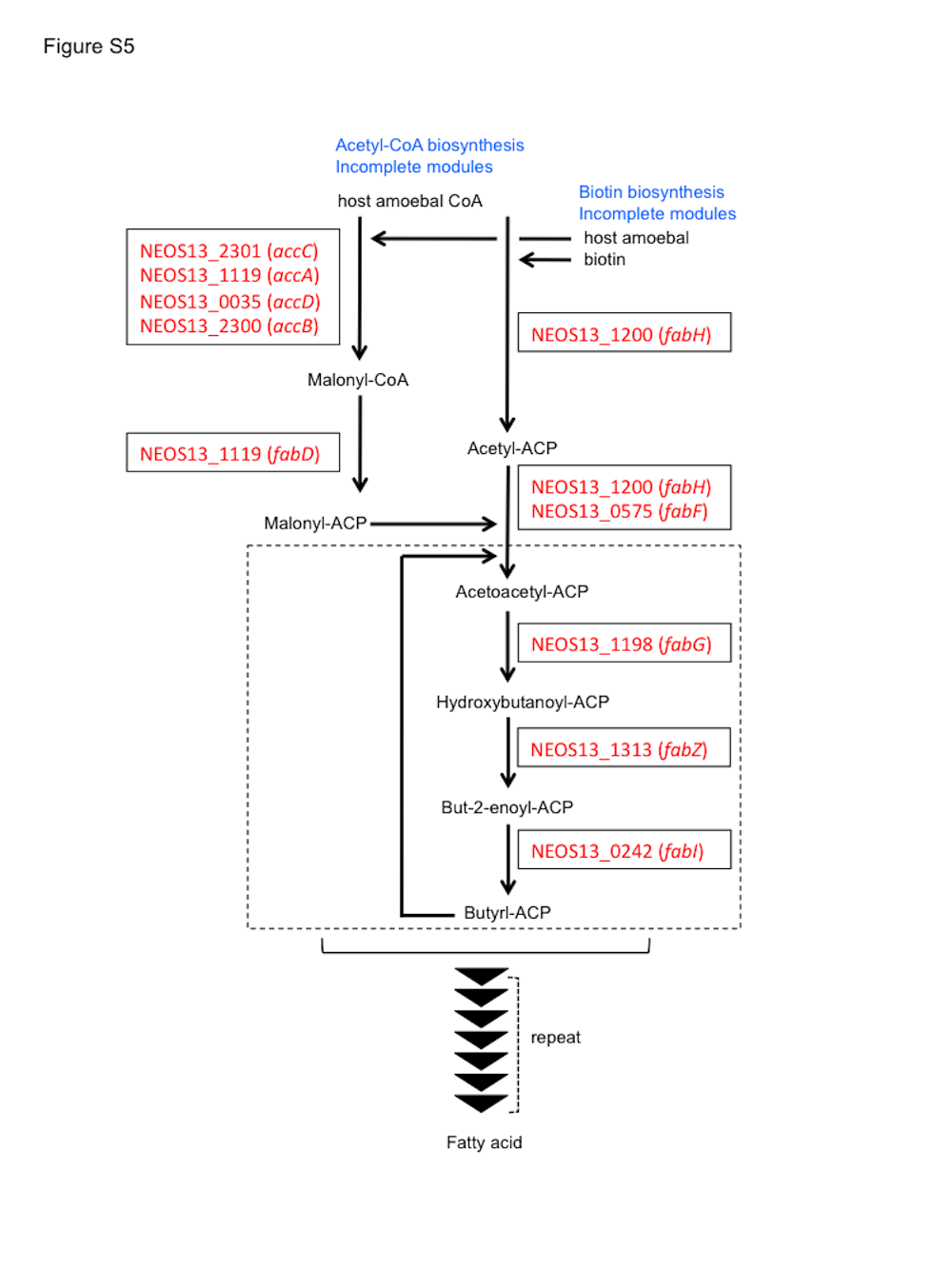

Supplement: Figure S5 — Predicted genes annotated as fatty acid initiation and elongation. Black lines with arrows show predicted active modules. Red names with numbers indicate Neochlamydia S13 gene IDs. (TIFF) [file pone.0095166.s005.tiff]

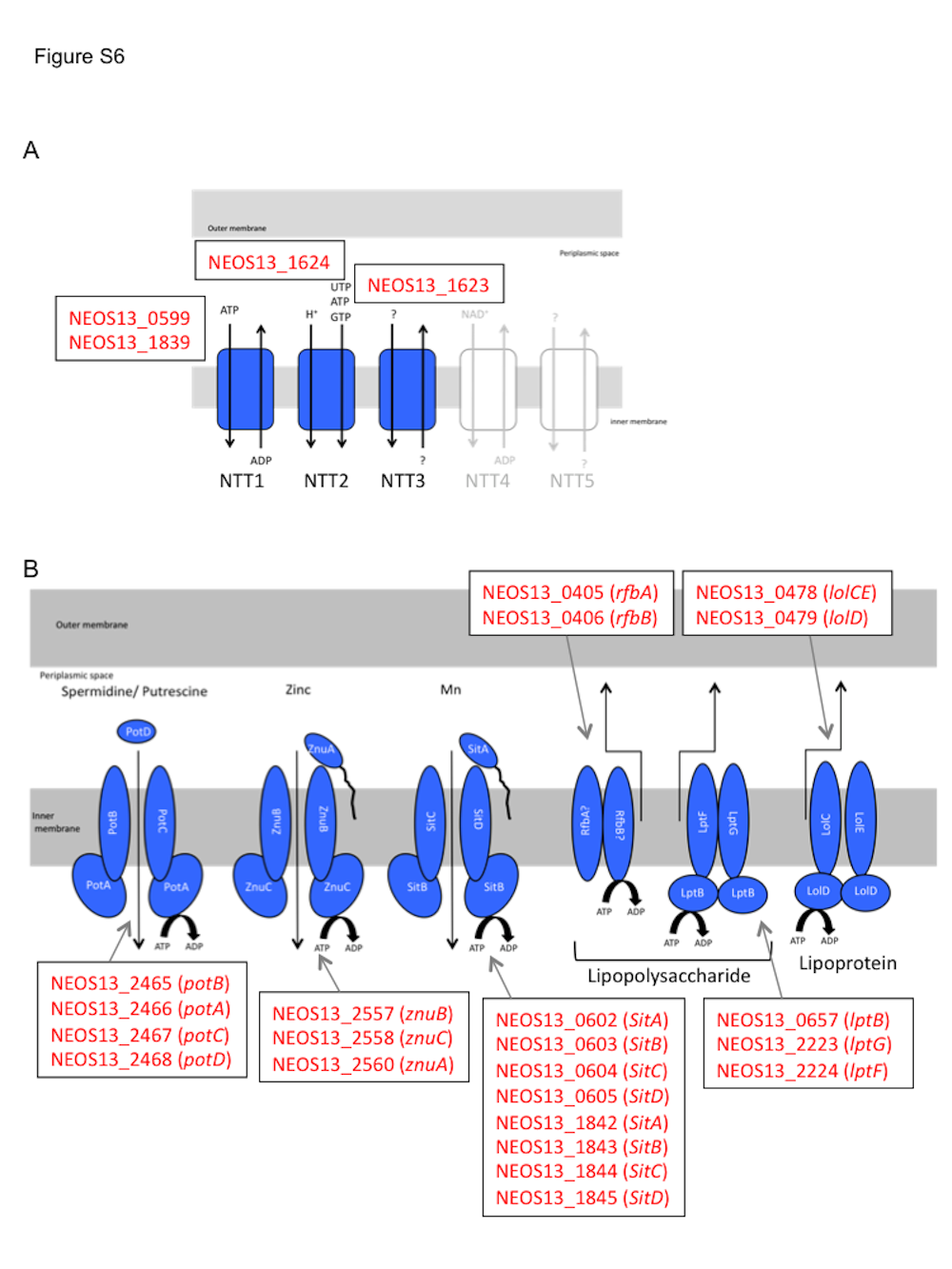

Supplement: Figure S6 — Predicted genes annotated as ATP/ADP translocases (NTTs) (A) and ABC transporters (B). Black lines with arrows show predicted active modules. Gray lines show incomplete modules. Red names with numbers indicate Neochlamydia S13 gene IDs. (TIFF) [file pone.0095166.s006.tiff]

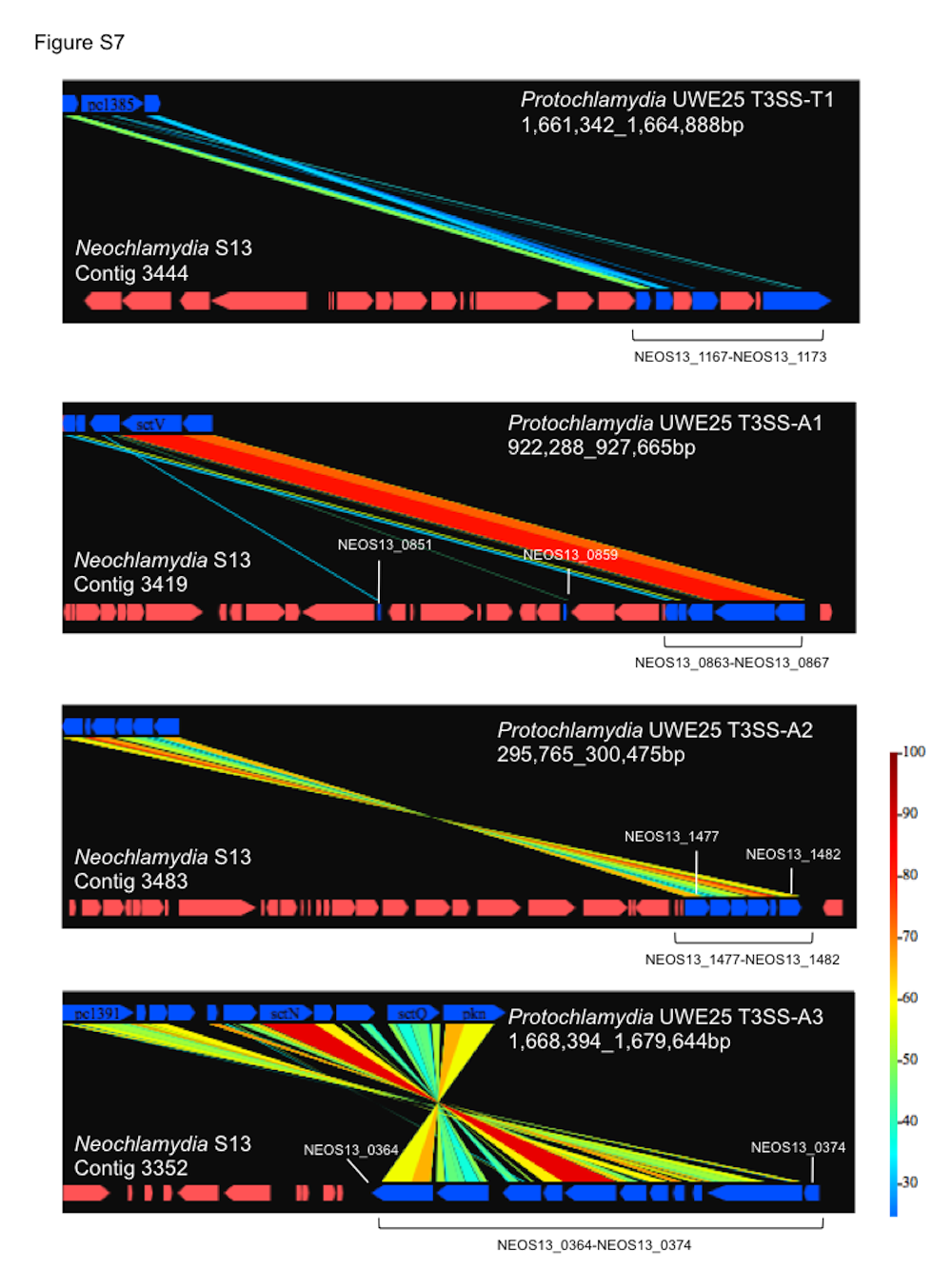

Supplement: Figure S7 — Comparison of genes encoding a type III secretion system from Neochlamydia S13 and Protochlamydia UWE25. The type III operon structures of the two chlamydiae are compared. Top panel, T3SS-T1; second panel, T3SS-A1; third panel, T3SS-A2; bottom panel, T3SS-A3. Right scale values show % identity estimated by BLASTp. Each of the gene cluster sequences in Protochlamydia amoebophila UWE25 (NC_005861.1) was obtained from NCBI (http:www.ncbi.nlm.nih.gov/genome). (TIFF) [file pone.0095166.s007.tiff]

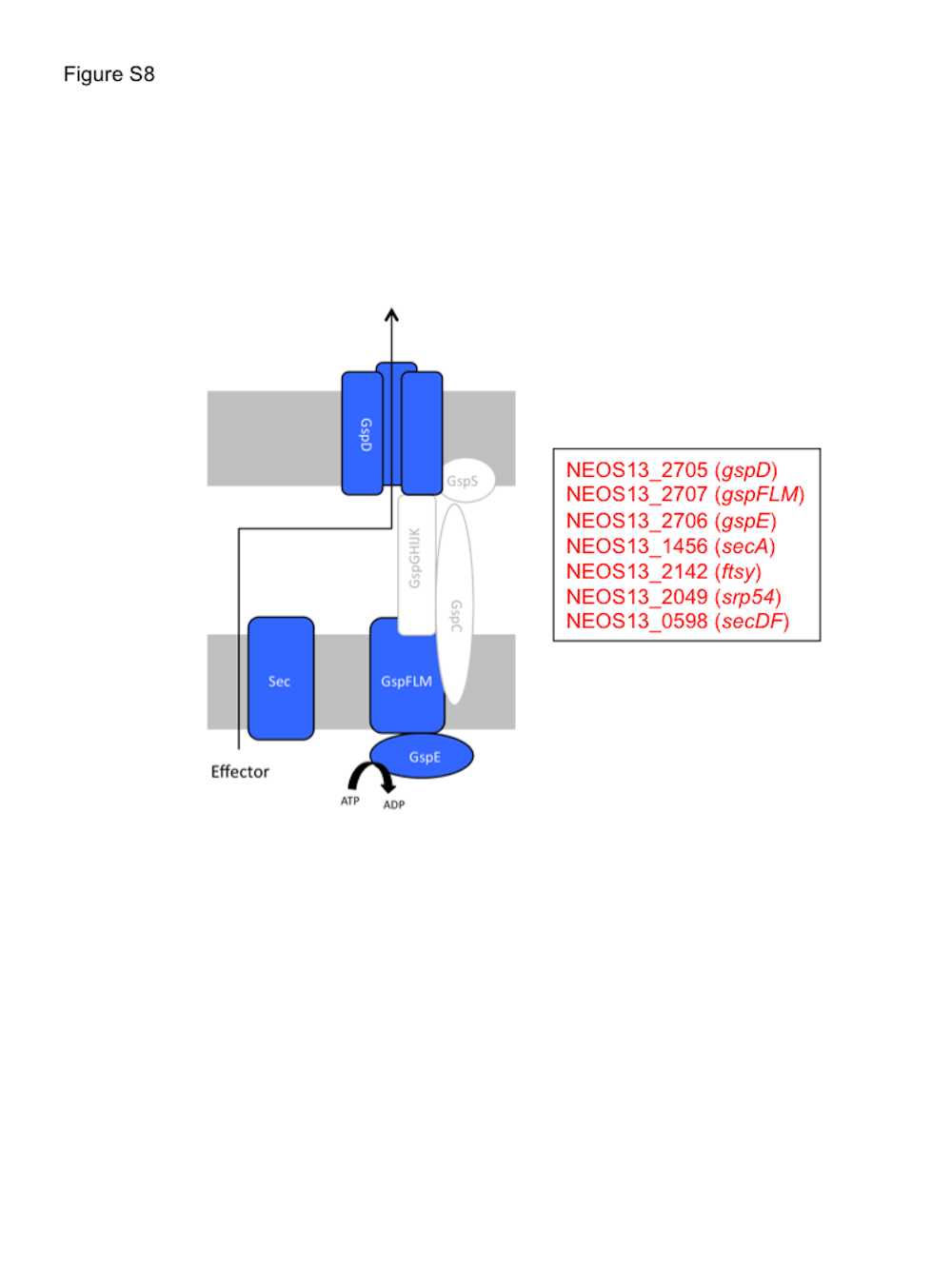

Supplement: Figure S8 — Predicted genes annotated as Sec-dependent type II secretion machinery. Black line with arrow shows predicted active module. Gray boxes show incomplete molecules. Red names with numbers indicate Neochlamydia S13 gene IDs. (TIFF) [file pone.0095166.s008.tiff]

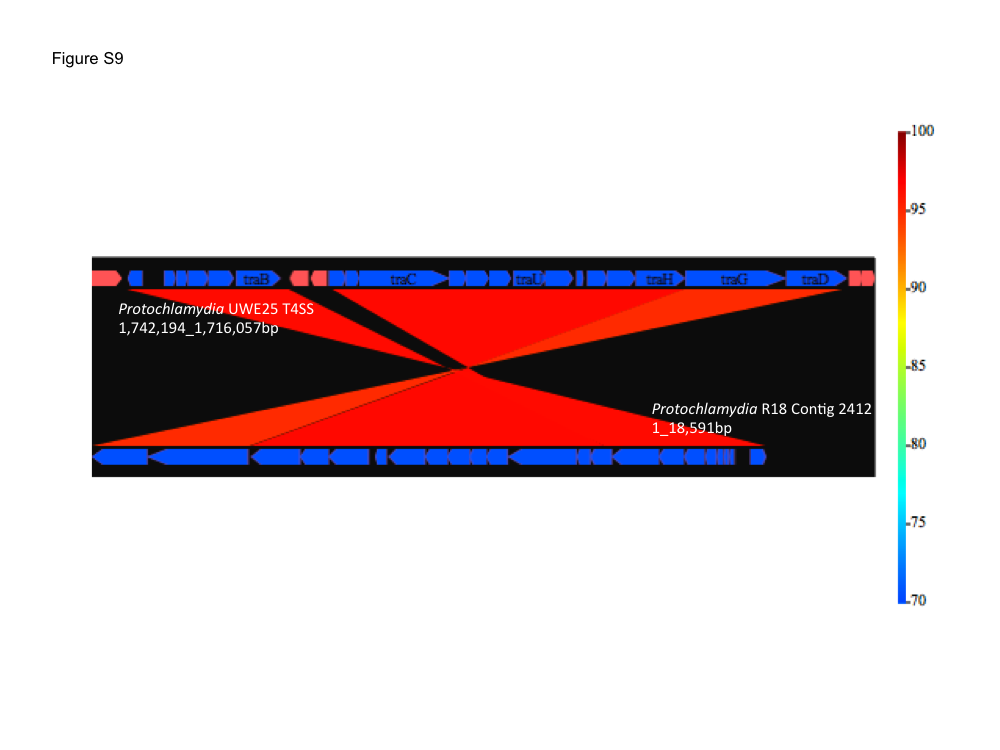

Supplement: Figure S9 — Comparative analysis of genes encoding type IV secretion machinery from Protochlamydia UWE25 and Protochlamydia R18. No annotated type IV genes were found in the Neochlamydia S13 genome. Blue boxes indicate individual coding regions of the type IV cluster. (TIFF) [file pone.0095166.s009.tiff]

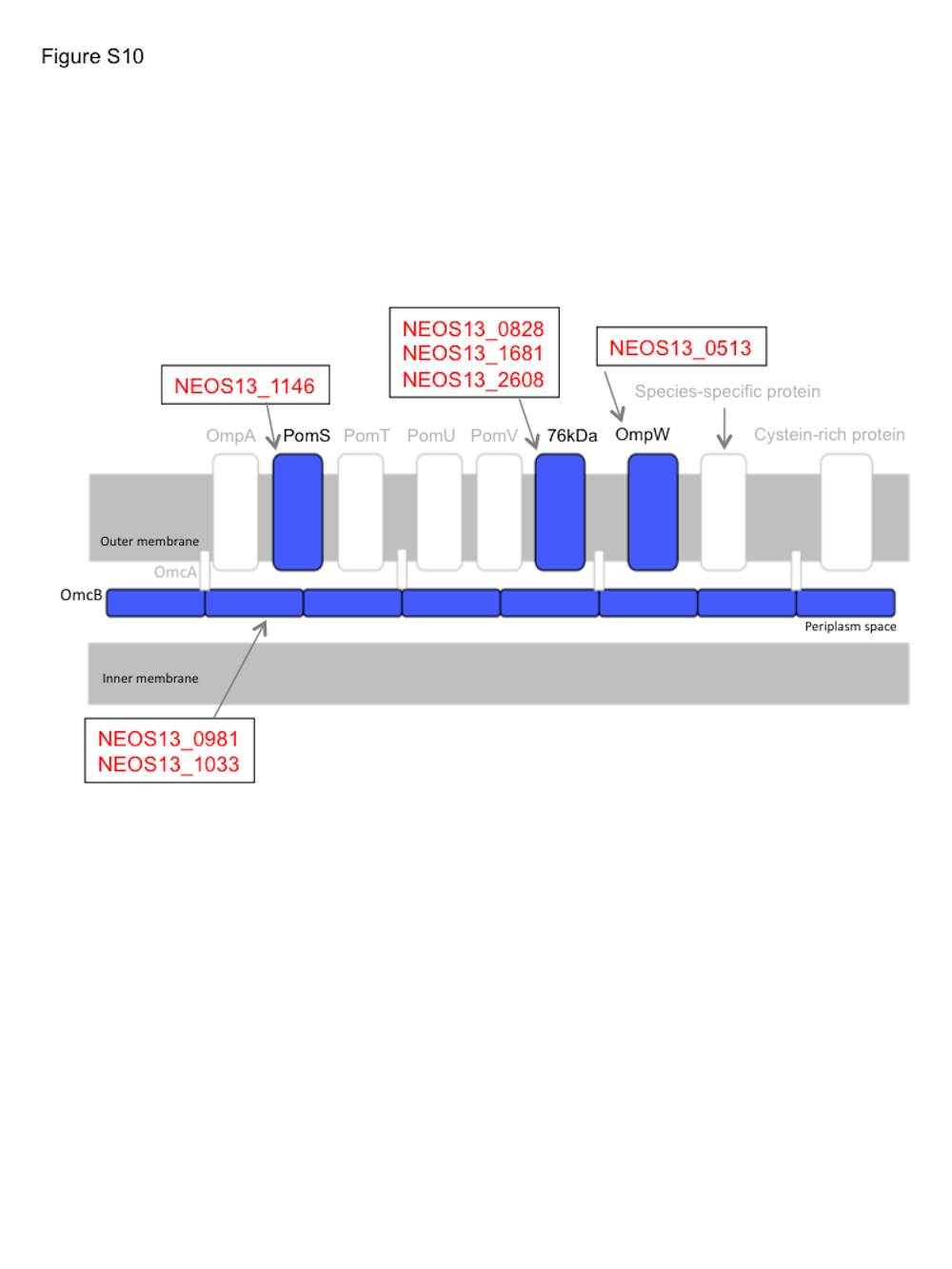

Supplement: Figure S10 — Predicted outer membrane structures. Blue molecules were predicted to be active. Gray molecules are absent. Red names with numbers indicate Neochlamydia S13 gene IDs. This figure depicts the predicted outer membrane structure based on a findings described by Heinz et al. [34] and previous findings published by Aistleitner et al. [33]. (TIFF) [file pone.0095166.s010.tiff]

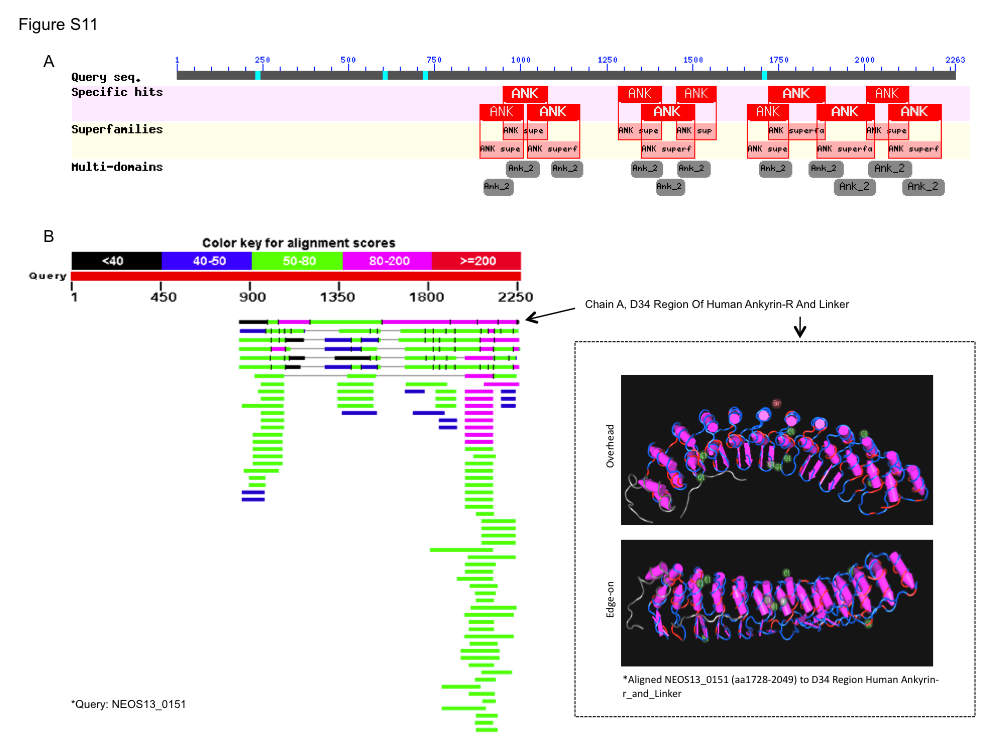

Supplement: Figure S11 — Characterization of a unique molecule with ankyrin domains (NEOS13_0151). (A) Detection of ankyrin domains in the molecule encoded by NEOS13_0151. (B) Alignment scores and 3D prediction. The scores and prediction were performed using the web program, protein BLAST with the MMDB (http://www.ncbi.nlm.nih.gov/Structure/MMDB/docs/mmdb_search.html). (TIFF) [file pone.0095166.s011.tiff]

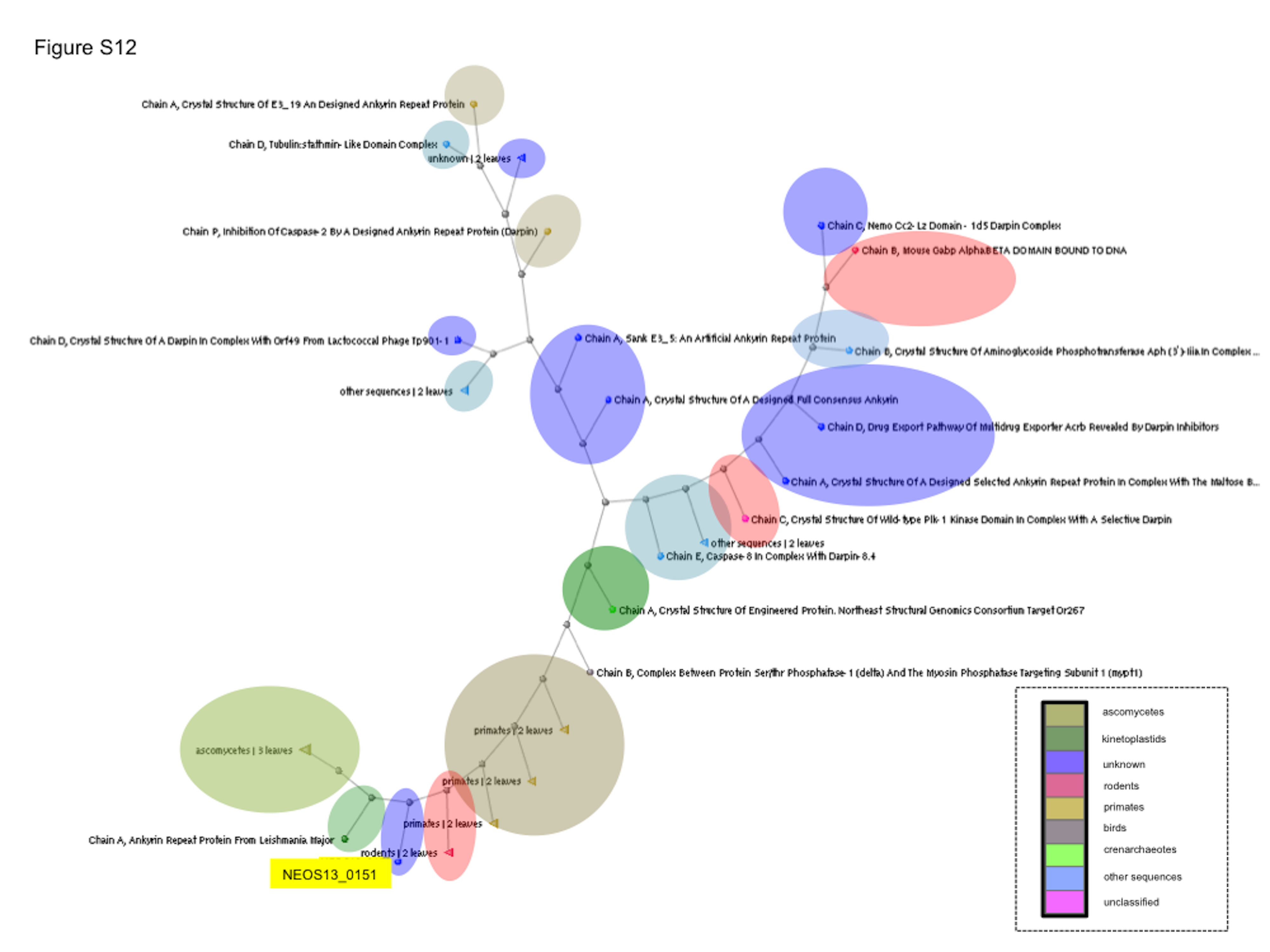

Supplement: Figure S12 — Phylogenetic comparison of the predicted protein sequence encoded by NEOS13_0151 with other eukaryotic proteins. The predicted protein sequence encoded by NEOS13_0151 was phylogenetically compared with previously reported sequences obtained from the GenBank database using ClustalW2. The phylogenetic trees generated from the aligned sequences were constructed by neighbor-joining in ClustalW2, and then visualized with the website viewer. (TIFF) [file pone.0095166.s012.tiff]
