# Supplementary material for: Amoebal Endosymbiont Neochlamydia Genome Sequence Illuminates the Bacterial Role in the Defense of the Host Amoebae against Legionella pneumophila
Source: PLoS One. 2014 Apr 18;9(4):e95166. doi: 10.1371/journal.pone.0095166 (PMC3991601; doi:10.1371/journal.pone.0095166)
Supplement: Table S1 — Neochlamydia S13 gene IDs with features. (PDF) [file pone.0095166.s013.pdf]

Table S1. *Neochlamydia* S13 gene IDs with features

Number	contig_id	Gene feature_ID	type	location	start	stop	strand	function	Categoly
1	1145	NEOS13_0001	peg	1145_88_237	88	237	+	Leucine-rich repeat containing protein	Leucine-rich repeat
2	1206	NEOS13_0002	peg	1206_274_146	274	146	-	hypothetical protein	
3	1444	NEOS13_0003	peg	1444_32_166	32	166	+	hypothetical protein	
4	1480	NEOS13_0004	peg	1480_35_169	35	169	+	hypothetical protein	
5	1534	NEOS13_0005	peg	1534_190_71	190	71	-	hypothetical protein	
6	1565	NEOS13_0006	peg	1565_43_228	43	228	+	hypothetical protein	
7	1614	NEOS13_0007	peg	1614_181_41	181	41	-	hypothetical protein	
8	1707	NEOS13_0008	peg	1707_215_352	215	352	+	hypothetical protein	
9	1746	NEOS13_0009	peg	1746_2986_2348	2986	2348	-	hypothetical protein	
10	1969	NEOS13_0010	peg	1969_198_25	198	25	-	hypothetical protein	
11	2031	NEOS13_0011	peg	2031_286_110	286	110	-	hypothetical protein	
12	209	NEOS13_0012	peg	209_2_361	2	361	+	Leucine-rich repeat containing protein	Leucine-rich repeat
13	2168	NEOS13_0013	peg	2168_243_127	243	127	-	hypothetical protein	
14	2168	NEOS13_0014	peg	2168_518_1780	518	1780	+	putative phosphoprotein phosphatase	
15	2168	NEOS13_0015	peg	2168_1907_2023	1907	2023	+	hypothetical protein	
16	2168	NEOS13_0016	peg	2168_4084_2618	4084	2618	-	Photolyase protein family	
17	2168	NEOS13_0017	peg	2168_6567_4846	6567	4846	-	Leucine-rich repeat containing protein	Leucine-rich repeat
18	2168	NEOS13_0018	peg	2168_6718_6834	6718	6834	+	hypothetical protein	
19	2168	NEOS13_0019	peg	2168_7328_7726	7328	7726	+	hypothetical protein	
20	2168	NEOS13_0020	peg	2168_7970_8515	7970	8515	+	hypothetical protein	
21	2220	NEOS13_0021	peg	2220_25_297	25	297	+	hypothetical protein	
22	2310	NEOS13_0022	peg	2310_709_233	709	233	-	hypothetical protein	
23	2310	NEOS13_0023	peg	2310_2288_840	2288	840	-	Probable low-affinity inorganic phosphate transporter	
24	2310	NEOS13_0024	peg	2310_2976_2299	2976	2299	-	Phosphate transport regulator (distant homolog of PhoU)	
25	2310	NEOS13_0025	peg	2310_3345_3491	3345	3491	+	hypothetical protein	
26	2406	NEOS13_0026	peg	2406_169_47	169	47	-	hypothetical protein	
27	2436	NEOS13_0027	peg	2436_153_443	153	443	+	putative lipoprotein	
28	2706	NEOS13_0028	peg	2706_13_156	13	156	+	hypothetical protein	
29	2750	NEOS13_0029	peg	2750_912_3479	912	3479	+	ATP-dependent Clp protease, ATP-binding subunit ClpC / Negative regulator of genetic competence clcC/mecB	Genetic_competence
30	2750	NEOS13_0030	peg	2750_3612_5360	3612	5360	+	hypothetical protein	
31	2750	NEOS13_0031	peg	2750_5369_7138	5369	7138	+	hypothetical protein	
32	2750	NEOS13_0032	peg	2750_7913_7224	7913	7224	-	PTS system, IIA component	
33	2750	NEOS13_0033	peg	2750_8377_7928	8377	7928	-	PTS system,IIA component	
34	2750	NEOS13_0034	peg	2750_8941_8489	8941	8489	-	Deoxyuridine 5'-triphosphate nucleotidohydrolase (EC 3.6.1.23)	
35	2750	NEOS13_0035	peg	2750_9873_8947	9873	8947	-	Acetyl-coenzyme A carboxyl transferase beta chain (EC 6.4.1.2)	Fatty _acid_initiation_elongation
36	2750	NEOS13_0036	peg	2750_9858_9971	9858	9971	+	hypothetical protein	
37	2750	NEOS13_0037	peg	2750_10668_10033	10668	10033	-	Superoxide dismutase [Mn] (EC 1.15.1.1)	
38	2858	NEOS13_0038	peg	2858_121_2	121	2	-	hypothetical protein	
39	2903	NEOS13_0039	peg	2903_122_235	122	235	+	hypothetical protein	
40	2903	NEOS13_0040	peg	2903_1559_429	1559	429	-	hypothetical protein	
41	2903	NEOS13_0041	peg	2903_2020_3399	2020	3399	+	GTPase and tRNA-U34 5-formylation enzyme TrmE	
42	2903	NEOS13_0042	peg	2903_3404_4027	3404	4027	+	Endonuclease III (EC 4.2.99.18)	
43	2903	NEOS13_0043	peg	2903_4194_6311	4194	6311	+	hypothetical protein	
44	2903	NEOS13_0044	peg	2903_6627_6274	6627	6274	-	hypothetical protein	
45	2936	NEOS13_0045	peg	2936_408_560	408	560	+	hypothetical protein	
46	2936	NEOS13_0046	peg	2936_1617_547	1617	547	-	RecA protein	
47	2936	NEOS13_0047	peg	2936_2969_1842	2969	1842	-	hypothetical protein	
48	2936	NEOS13_0048	peg	2936_3751_3131	3751	3131	-	hypothetical protein	
49	2936	NEOS13_0049	peg	2936_5460_3772	5460	3772	-	hypothetical protein	
50	2936	NEOS13_0050	peg	2936_5583_5834	5583	5834	+	Cell division protein YlmG/Ycf19 (putative), YggT family	
51	2936	NEOS13_0051	peg	2936_5858_6874	5858	6874	+	tRNA dihydrouridine synthase B (EC 1.-.-.-)	
52	2936	NEOS13_0052	peg	2936_7111_7284	7111	7284	+	hypothetical protein	
53	2936	NEOS13_0053	peg	2936_7914_7798	7914	7798	-	hypothetical protein	
54	2936	NEOS13_0054	peg	2936_8110_8250	8110	8250	+	hypothetical protein	
55	2936	NEOS13_0055	peg	2936_8824_8372	8824	8372	-	hypothetical protein	
56	2936	NEOS13_0056	peg	2936_9189_9317	9189	9317	+	hypothetical protein	
57	2936	NEOS13_0057	peg	2936_9686_9474	9686	9474	-	hypothetical protein	
58	2936	NEOS13_0058	peg	2936_10176_9874	10176	9874	-	Bsr0701 protein	
59	2936	NEOS13_0059	peg	2936_13199_10548	13199	10548	-	Osmosensitive K+ channel histidine kinase KdpD (EC 2.7.3.-)	
60	2936	NEOS13_0060	peg	2936_14499_17528	14499	17528	+	hypothetical protein	
61	2936	NEOS13_0061	peg	2936_17625_17792	17625	17792	+	hypothetical protein	
62	2936	NEOS13_0062	peg	2936_18797_20242	18797	20242	+	Heat shock protein Hsp70:G-protein beta WD-40 repeat	
63	2936	NEOS13_0063	peg	2936_22798_20537	22798	20537	-	DinG family ATP-dependent helicase YoaA	
64	2936	NEOS13_0064	peg	2936_24776_23112	24776	23112	-	RNA polymerase sigma factor RpoD	
65	2936	NEOS13_0065	peg	2936_25055_25927	25055	25927	+	competence protein CoiA	Genetic_competence
66	2936	NEOS13_0066	peg	2936_26283_25993	26283	25993	-	30S ribosomal protein S20	
67	2936	NEOS13_0067	peg	2936_26424_27269	26424	27269	+	Ribosomal large subunit pseudouridine synthase D (EC 4.2.1.70)	
68	2936	NEOS13_0068	peg	2936_27277_27705	27277	27705	+	hypothetical protein	
69	2936	NEOS13_0069	peg	2936_28224_29357	28224	29357	+	hypothetical protein	

70	2936	NEOS13_0070	peg	2936_29439_30023	29439	30023	+	16S rRNA (guanine(966)-N(2))-methyltransferase (EC 2.1.1.171) ## SSU rRNA m(2)G966	
71	2937	NEOS13_0071	peg	2937_152_27	152	27	-	hypothetical protein	
72	2937	NEOS13_0072	peg	2937_528_1121	528	1121	+	hypothetical protein	
73	2937	NEOS13_0073	peg	2937_1513_1313	1513	1313	-	hypothetical protein	
74	3111	NEOS13_0074	peg	3111_280_149	280	149	-	hypothetical protein	
75	3214	NEOS13_0075	peg	3214_54_1730	54	1730	+	hypothetical protein	
76	3214	NEOS13_0076	peg	3214_2174_2007	2174	2007	-	hypothetical protein	
77	3214	NEOS13_0077	peg	3214_2686_2814	2686	2814	+	hypothetical protein	
78	3319	NEOS13_0078	peg	3319_1067_285	1067	285	-	conserved hypothetical protein	
79	3320	NEOS13_0079	peg	3320_470_180	470	180	-	hypothetical protein	
80	3320	NEOS13_0080	peg	3320_1869_895	1869	895	-	tRNA dihydrouridine synthase B (EC 1.-.-.-)	
81	3320	NEOS13_0081	peg	3320_2632_2321	2632	2321	-	hypothetical protein	
82	3320	NEOS13_0082	peg	3320_2774_4216	2774	4216	+	2-oxoglutarate/malate translocator	
83	3320	NEOS13_0083	peg	3320_5157_4303	5157	4303	-	Peptide methionine sulfoxide reductase MsrA (EC 1.8.4.11)	
84	3320	NEOS13_0084	peg	3320_5596_5826	5596	5826	+	hypothetical protein	
85	3321	NEOS13_0085	peg	3321_202_71	202	71	-	hypothetical protein	
86	3321	NEOS13_0086	peg	3321_265_381	265	381	+	hypothetical protein	
87	3321	NEOS13_0087	peg	3321_1868_546	1868	546	-	Leucine-rich repeat containing protein	Leucine-rich repeat
88	3322	NEOS13_0088	peg	3322_95_844	95	844	+	hypothetical protein	
89	3322	NEOS13_0089	peg	3322_1860_850	1860	850	-	hypothetical protein	
90	3322	NEOS13_0090	peg	3322_2605_1826	2605	1826	-	hypothetical protein	
91	3322	NEOS13_0091	peg	3322_2806_2606	2806	2606	-	hypothetical protein	
92	3322	NEOS13_0092	peg	3322_3554_6112	3554	6112	+	Glycogen phosphorylase (EC 2.4.1.1)	
93	3322	NEOS13_0093	peg	3322_6892_6158	6892	6158	-	putative amino acid ABC transporter, periplasmic amino acid-binding protein	ABC_transporter
94	3322	NEOS13_0094	peg	3322_7350_7237	7350	7237	-	hypothetical protein	
95	3322	NEOS13_0095	peg	3322_8014_9360	8014	9360	+	Glucose-1-phosphate adenylyltransferase (EC 2.7.7.27)	
96	3322	NEOS13_0096	peg	3322_9376_10128	9376	10128	+	hypothetical protein	
97	3322	NEOS13_0097	peg	3322_10612_10737	10612	10737	+	hypothetical protein	
98	3322	NEOS13_0098	peg	3322_11641_11862	11641	11862	+	hypothetical protein	
99	3322	NEOS13_0099	peg	3322_12080_14659	12080	14659	+	hypothetical protein	
100	3322	NEOS13_0100	peg	3322_14990_14826	14990	14826	-	hypothetical protein	
101	3322	NEOS13_0101	peg	3322_15165_15043	15165	15043	-	hypothetical protein	
102	3322	NEOS13_0102	peg	3322_15750_16610	15750	16610	+	Putative secreted protein	
103	3322	NEOS13_0103	peg	3322_17025_17771	17025	17771	+	Mobile element protein	
104	3322	NEOS13_0104	peg	3322_18327_18106	18327	18106	-	Leucine-rich repeat containing protein	Leucine-rich repeat
105	3324	NEOS13_0105	peg	3324_2067_595	2067	595	-	Leucine-rich repeat containing protein	Leucine-rich repeat
106	3324	NEOS13_0106	peg	3324_2453_2722	2453	2722	+	hypothetical protein	
107	3326	NEOS13_0001ma	rna	3326_422_3449	422	3449	+	Large Subunit Ribosomal RNA; lsuRNA; LSU rRNA	RNA
108	3327	NEOS13_0002ma	rna	3327_234_306	234	306	+	tRNA-Ala-TGC	RNA
109	3327	NEOS13_0003ma	rna	3327_319_392	319	392	+	tRNA-Ile-GAT	RNA
110	3327	NEOS13_0004ma	rna	3327_769_3796	769	3796	+	Large Subunit Ribosomal RNA; lsuRNA; LSU rRNA	RNA
111	3328	NEOS13_0107	peg	3328_38_415	38	415	+	Mobile element protein	
112	3328	NEOS13_0108	peg	3328_1293_538	1293	538	-	Leucine-rich repeat containing protein	Leucine-rich repeat
113	3329	NEOS13_0109	peg	3329_89_850	89	850	+	Leucine-rich repeat containing protein	Leucine-rich repeat
114	3329	NEOS13_0110	peg	3329_823_1044	823	1044	+	Leucine-rich repeat containing protein	Leucine-rich repeat
115	3330	NEOS13_0111	peg	3330_27_467	27	467	+	Leucine-rich repeat containing protein	Leucine-rich repeat
116	3331	NEOS13_0112	peg	3331_1051_110	1051	110	-	Leucine-rich repeat containing protein	Leucine-rich repeat
117	3332	NEOS13_0113	peg	3332_165_350	165	350	+	hypothetical protein	
118	3332	NEOS13_0114	peg	3332_483_361	483	361	-	hypothetical protein	
119	3332	NEOS13_0115	peg	3332_1015_533	1015	533	-	hypothetical protein	
120	3332	NEOS13_0116	peg	3332_2067_1516	2067	1516	-	hypothetical protein	
121	3332	NEOS13_0117	peg	3332_4464_2080	4464	2080	-	hypothetical protein	
122	3333	NEOS13_0118	peg	3333_1274_513	1274	513	-	hypothetical protein	
123	3333	NEOS13_0119	peg	3333_2701_1676	2701	1676	-	Transposase	Transposase
124	3333	NEOS13_0120	peg	3333_3010_2729	3010	2729	-	hypothetical protein	
125	3333	NEOS13_0121	peg	3333_3325_3441	3325	3441	+	hypothetical protein	
126	3333	NEOS13_0122	peg	3333_3751_3626	3751	3626	-	hypothetical protein	
127	3333	NEOS13_0123	peg	3333_3713_3910	3713	3910	+	hypothetical protein	
128	3333	NEOS13_0124	peg	3333_4068_4472	4068	4472	+	Mobile element protein	
129	3334	NEOS13_0125	peg	3334_471_938	471	938	+	Retron-type RNA-directed DNA polymerase (EC 2.7.7.49)	
130	3336	NEOS13_0126	peg	3336_2729_195	2729	195	-	Leucine-rich repeat containing protein	Leucine-rich repeat
131	3336	NEOS13_0127	peg	3336_4101_3121	4101	3121	-	YgjD/Kae1/Qri7 family, required for threonylcarbamoyladenosine (t(6)A) formation in tRNA	
132	3336	NEOS13_0128	peg	3336_4177_4308	4177	4308	+	hypothetical protein	
133	3336	NEOS13_0129	peg	3336_5244_4477	5244	4477	-	6-phosphogluconolactonase (EC 3.1.1.31), eukaryotic type	Pentose_phospate_pathway Enter_Doudroff_pathway
134	3336	NEOS13_0130	peg	3336_6946_5381	6946	5381	-	Glucose-6-phosphate 1-dehydrogenase (EC 1.1.1.49)	Pentose_phospate_pathway Enter_Doudroff_pathway
135	3336	NEOS13_0131	peg	3336_7580_7053	7580	7053	-	Protein of unknown function DUF55	
136	3336	NEOS13_0132	peg	3336_8061_7570	8061	7570	-	Putative Holliday junction resolvase (EC 3.1.-.-)	
137	3336	NEOS13_0133	peg	3336_9677_8058	9677	8058	-	CTP synthase (EC 6.3.4.2)	
138	3336	NEOS13_0134	peg	3336_10432_9653	10432	9653	-	3-deoxy-manno-octulosonate cytidylyltransferase (EC 2.7.7.38)	

139	3336	NEOS13_0135	peg	3336_11868_10630	11868	10630	-	Inner membrane protein YihY, formerly thought to be RNase BN	
140	3336	NEOS13_0136	peg	3336_11871_11987	11871	11987	+	hypothetical protein	
141	3336	NEOS13_0137	peg	3336_12314_12057	12314	12057	-	hypothetical protein	
142	3336	NEOS13_0138	peg	3336_12731_12600	12731	12600	-	hypothetical protein	
143	3336	NEOS13_0139	peg	3336_12735_12929	12735	12929	+	hypothetical protein	
144	3336	NEOS13_0140	peg	3336_14334_13009	14334	13009	-	hypothetical protein	
145	3336	NEOS13_0141	peg	3336_15080_14958	15080	14958	-	hypothetical protein	
146	3336	NEOS13_0142	peg	3336_15497_15345	15497	15345	-	Mobile element protein	
147	3336	NEOS13_0143	peg	3336_15984_15625	15984	15625	-	putative transposase	Transposase
148	3336	NEOS13_0144	peg	3336_16521_16117	16521	16117	-	transcriptional regulator, XRE family	
149	3336	NEOS13_0145	peg	3336_16853_16527	16853	16527	-	Protein of unknown function DUF891	
150	3336	NEOS13_0146	peg	3336_17475_18062	17475	18062	+	hypothetical protein	
151	3336	NEOS13_0147	peg	3336_18398_18285	18398	18285	-	hypothetical protein	
152	3336	NEOS13_0148	peg	3336_18732_18848	18732	18848	+	hypothetical protein	
153	3336	NEOS13_0149	peg	3336_19031_19978	19031	19978	+	hypothetical protein	
154	3336	NEOS13_0150	peg	3336_20061_21554	20061	21554	+	hypothetical protein	
155	3336	NEOS13_0151	peg	3336_21581_28372	21581	28372	+	ankyrin repeat protein	Ankyrin
156	3336	NEOS13_0152	peg	3336_28582_32010	28582	32010	+	hypothetical protein	
157	3337	NEOS13_0153	peg	3337_9_143	9	143	+	hypothetical protein	
158	3337	NEOS13_0154	peg	3337_1481_873	1481	873	-	hypothetical protein	
159	3338	NEOS13_0155	peg	3338_556_954	556	954	+	hypothetical protein	
160	3338	NEOS13_0156	peg	3338_947_1228	947	1228	+	hypothetical protein	
161	3338	NEOS13_0157	peg	3338_1260_1424	1260	1424	+	hypothetical protein	
162	3339	NEOS13_0158	peg	3339_809_1909	809	1909	+	GTP-binding and nucleic acid-binding protein YchF	
163	3339	NEOS13_0159	peg	3339_1922_3064	1922	3064	+	Methyltransferase (EC 2.1.1.-)	
164	3339	NEOS13_0160	peg	3339_5839_3323	5839	3323	-	ATP-dependent protease La (EC 3.4.21.53) Type I	
165	3339	NEOS13_0161	peg	3339_6217_7371	6217	7371	+	Deoxyguanosinetriphosphate triphosphohydrolase (EC 3.1.5.1)	
166	3339	NEOS13_0162	peg	3339_7359_8045	7359	8045	+	Inactive homolog of metal-dependent proteases, putative molecular chaperone	
167	3339	NEOS13_0163	peg	3339_8144_8323	8144	8323	+	SSU ribosomal protein S21p	
168	3339	NEOS13_0164	peg	3339_8461_9657	8461	9657	+	Chaperone protein DnaJ	
169	3339	NEOS13_0165	peg	3339_10138_11112	10138	11112	+	hypothetical protein	
170	3339	NEOS13_0166	peg	3339_11330_11461	11330	11461	+	hypothetical protein	
171	3340	NEOS13_0167	peg	3340_1664_6	1664	6	-	Leucine-rich repeat containing protein	Leucine-rich repeat
172	3340	NEOS13_0168	peg	3340_2290_2147	2290	2147	-	hypothetical protein	
173	3340	NEOS13_0169	peg	3340_2406_2582	2406	2582	+	hypothetical protein	
174	3341	NEOS13_0170	peg	3341_179_400	179	400	+	hypothetical protein	
175	3341	NEOS13_0171	peg	3341_733_2337	733	2337	+	V8-like Glu-specific endopeptidase	
176	3341	NEOS13_0172	peg	3341_2601_3221	2601	3221	+	hypothetical protein	
177	3341	NEOS13_0173	peg	3341_3722_3465	3722	3465	-	hypothetical protein	
178	3341	NEOS13_0174	peg	3341_4957_4103	4957	4103	-	hypothetical protein	
179	3341	NEOS13_0175	peg	3341_5613_4960	5613	4960	-	hypothetical protein	
180	3342	NEOS13_0176	peg	3342_553_428	553	428	-	hypothetical protein	
181	3342	NEOS13_0005ma	rna	3342_4071_1044	4071	1044	-	Large Subunit Ribosomal RNA; lsuRNA; LSU rRNA	RNA
182	3343	NEOS13_0177	peg	3343_711_589	711	589	-	hypothetical protein	
183	3346	NEOS13_0178	peg	3346_39_476	39	476	+	Mobile element protein	
184	3347	NEOS13_0179	peg	3347_765_1430	765	1430	+	hypothetical protein	
185	3347	NEOS13_0180	peg	3347_1551_1685	1551	1685	+	hypothetical protein	
186	3347	NEOS13_0181	peg	3347_2187_2303	2187	2303	+	hypothetical protein	
187	3347	NEOS13_0182	peg	3347_2973_2848	2973	2848	-	hypothetical protein	
188	3347	NEOS13_0183	peg	3347_3469_3290	3469	3290	-	Protein of unknown function DUF891	
189	3347	NEOS13_0184	peg	3347_3950_4876	3950	4876	+	hypothetical protein	
190	3347	NEOS13_0185	peg	3347_4997_5143	4997	5143	+	hypothetical protein	
191	3347	NEOS13_0186	peg	3347_5645_5436	5645	5436	-	hypothetical protein	
192	3347	NEOS13_0187	peg	3347_5681_6472	5681	6472	+	ISSod10, transposase OrfB	Transposase
193	3347	NEOS13_0188	peg	3347_7967_6705	7967	6705	-	Proton/glutamate symport protein @ Sodium/glutamate symport protein	
194	3347	NEOS13_0189	peg	3347_8870_8193	8870	8193	-	Hypothetical protein YggS, proline synthase co-transcribed bacterial homolog PROSC	
195	3347	NEOS13_0190	peg	3347_9436_8873	9436	8873	-	hypothetical protein	
196	3347	NEOS13_0191	peg	3347_10616_9597	10616	9597	-	DUF1432 domain-containing protein	
197	3347	NEOS13_0192	peg	3347_11081_10620	11081	10620	-	YqeZ	
198	3347	NEOS13_0193	peg	3347_11283_11143	11283	11143	-	hypothetical protein	
199	3347	NEOS13_0194	peg	3347_11890_11249	11890	11249	-	Inorganic pyrophosphatase (EC 3.6.1.1)	
200	3347	NEOS13_0195	peg	3347_12151_13371	12151	13371	+	cytosine permease	
201	3347	NEOS13_0196	peg	3347_13355_14434	13355	14434	+	protein of unknown function DUF917	
202	3347	NEOS13_0197	peg	3347_14431_15999	14431	15999	+	N-methylhydantoinase (ATP-hydrolyzing) (EC 3.5.2.14)	
203	3347	NEOS13_0198	peg	3347_17346_16039	17346	16039	-	Glutamate-1-semialdehyde aminotransferase (EC 5.4.3.8)	
204	3347	NEOS13_0199	peg	3347_18851_17574	18851	17574	-	hypothetical protein	
205	3347	NEOS13_0200	peg	3347_19157_20938	19157	20938	+	hypothetical protein	
206	3347	NEOS13_0201	peg	3347_20940_24485	20940	24485	+	hypothetical protein	
207	3347	NEOS13_0202	peg	3347_24603_24463	24603	24463	-	hypothetical protein	
208	3347	NEOS13_0203	peg	3347_24737_25651	24737	25651	+	FIG00899508: hypothetical protein	
209	3347	NEOS13_0204	peg	3347_25662_26456	25662	26456	+	Methionine ABC transporter ATP-binding protein	ABC_transporter
210	3347	NEOS13_0205	peg	3347_26453_27640	26453	27640	+	FIG00899462: hypothetical protein	
211	3347	NEOS13_0206	peg	3347_27711_28280	27711	28280	+	transcriptional regulator, putative	
212	3347	NEOS13_0207	peg	3347_28306_29016	28306	29016	+	conserved hypothetical protein	
213	3347	NEOS13_0208	peg	3347_29233_29358	29233	29358	+	hypothetical protein	
214	3347	NEOS13_0209	peg	3347_29514_30863	29514	30863	+	ankyrin repeat protein	Ankyrin

215	3347	NEOS13_0210	peg	3347_31354_31094	31354	31094	-	hypothetical protein	
216	3347	NEOS13_0211	peg	3347_31960_31574	31960	31574	-	SSU ribosomal protein S9p (S16e)	
217	3347	NEOS13_0212	peg	3347_32421_31975	32421	31975	-	LSU ribosomal protein L13p (L13Ae)	
218	3347	NEOS13_0213	peg	3347_32690_34027	32690	34027	+	tRNA-i(6)A37 methylthiotransferase	
219	3347	NEOS13_0214	peg	3347_36236_34245	36236	34245	-	DNA ligase (EC 6.5.1.2)	
220	3347	NEOS13_0215	peg	3347_38415_36244	38415	36244	-	1,4-alpha-glucan (glycogen) branching enzyme, GH-13-type (EC 2.4.1.18)	
221	3347	NEOS13_0216	peg	3347_38914_38798	38914	38798	-	hypothetical protein	
222	3347	NEOS13_0217	peg	3347_39481_40833	39481	40833	+	hypothetical protein	
223	3347	NEOS13_0218	peg	3347_41108_40992	41108	40992	-	hypothetical protein	
224	3347	NEOS13_0219	peg	3347_41147_41266	41147	41266	+	hypothetical protein	
225	3347	NEOS13_0220	peg	3347_41418_41293	41418	41293	-	hypothetical protein	
226	3347	NEOS13_0221	peg	3347_41540_41412	41540	41412	-	hypothetical protein	
227	3347	NEOS13_0222	peg	3347_42009_41587	42009	41587	-	hypothetical protein	
228	3347	NEOS13_0223	peg	3347_42361_41987	42361	41987	-	hypothetical protein	
229	3347	NEOS13_0224	peg	3347_42536_42375	42536	42375	-	hypothetical protein	
230	3347	NEOS13_0225	peg	3347_43065_42694	43065	42694	-	hypothetical protein	
231	3347	NEOS13_0226	peg	3347_43582_43457	43582	43457	-	hypothetical protein	
232	3347	NEOS13_0227	peg	3347_43694_43807	43694	43807	+	hypothetical protein	
233	3347	NEOS13_0228	peg	3347_44937_43924	44937	43924	-	FIG00494467: hypothetical protein	
234	3347	NEOS13_0229	peg	3347_46234_44942	46234	44942	-	MiaB family protein, possibly involved in tRNA or rRNA modification	
235	3347	NEOS13_0230	peg	3347_47069_46359	47069	46359	-	DNA repair protein RadC	
236	3347	NEOS13_0231	peg	3347_47617_47120	47617	47120	-	hypothetical protein	
237	3347	NEOS13_0232	peg	3347_47940_48176	47940	48176	+	hypothetical protein	
238	3347	NEOS13_0233	peg	3347_48308_48967	48308	48967	+	N-Acetylneuraminate cytidylyltransferase (EC 2.7.7.43)	
239	3347	NEOS13_0234	peg	3347_48970_50535	48970	50535	+	N-acetylneuraminate synthase (EC 2.5.1.56)	
240	3347	NEOS13_0235	peg	3347_50571_51206	50571	51206	+	HAD-superfamily hydrolase, subfamily IA, variant 3 protein family protein	
241	3347	NEOS13_0236	peg	3347_51193_51903	51193	51903	+	hypothetical protein	
242	3348	NEOS13_0237	peg	3348_15_1688	15	1688	+	hypothetical protein	
243	3348	NEOS13_0238	peg	3348_1973_1857	1973	1857	-	hypothetical protein	
244	3348	NEOS13_0239	peg	3348_2235_3125	2235	3125	+	hypothetical protein	
245	3348	NEOS13_0240	peg	3348_3252_5162	3252	5162	+	ABC transporter ATP-binding protein	ABC_transporter
246	3348	NEOS13_0241	peg	3348_6626_5670	6626	5670	-	unknown protein	
247	3348	NEOS13_0242	peg	3348_6951_7853	6951	7853	+	Enoyl-[acyl-carrier-protein] reductase [NADH] (EC 1.3.1.9)	Fatty _acid_initiation_elongation
248	3348	NEOS13_0243	peg	3348_7896_8078	7896	8078	+	hypothetical protein	
249	3348	NEOS13_0244	peg	3348_8699_8163	8699	8163	-	hypothetical protein	
250	3348	NEOS13_0245	peg	3348_10528_9035	10528	9035	-	hypothetical protein	
251	3348	NEOS13_0246	peg	3348_11244_10531	11244	10531	-	FolM Alternative dihydrofolate reductase 1	
252	3348	NEOS13_0247	peg	3348_11393_11241	11393	11241	-	hypothetical protein	
253	3348	NEOS13_0248	peg	3348_12015_11608	12015	11608	-	putative dGTP pyrophosphohydrolase/dihydroneopterin aldolase (mutT/folB, fusion protein)	
254	3348	NEOS13_0249	peg	3348_12843_12058	12843	12058	-	hypothetical protein	
255	3348	NEOS13_0250	peg	3348_16233_13690	16233	13690	-	DNA mismatch repair protein MutS	
256	3348	NEOS13_0251	peg	3348_17087_18535	17087	18535	+	hypothetical protein	
257	3348	NEOS13_0252	peg	3348_18589_18744	18589	18744	+	hypothetical protein	
258	3348	NEOS13_0253	peg	3348_19395_20486	19395	20486	+	hypothetical protein	
259	3348	NEOS13_0254	peg	3348_21024_20788	21024	20788	-	Mobile element protein	
260	3348	NEOS13_0255	peg	3348_21195_21326	21195	21326	+	hypothetical protein	
261	3348	NEOS13_0256	peg	3348_21571_21323	21571	21323	-	Mobile element protein	
262	3348	NEOS13_0257	peg	3348_21698_21582	21698	21582	-	hypothetical protein	
263	3348	NEOS13_0258	peg	3348_22703_21843	22703	21843	-	hypothetical protein	
264	3348	NEOS13_0259	peg	3348_23244_22696	23244	22696	-	hypothetical protein	
265	3348	NEOS13_0260	peg	3348_23682_25682	23682	25682	+	FIG00899452: hypothetical protein	
266	3348	NEOS13_0261	peg	3348_26257_26475	26257	26475	+	hypothetical protein	
267	3348	NEOS13_0262	peg	3348_26459_27184	26459	27184	+	Ubiquinone/menaquinone biosynthesis methyltransferase UbiE (EC 2.1.1.-) @ 2-heptaprenyl-1,4-naphthoquinone methyltransferase (EC 2.1.1.163)	
268	3348	NEOS13_0263	peg	3348_28485_27460	28485	27460	-	O-succinylbenzoic acid--CoA ligase (EC 6.2.1.26)	
269	3348	NEOS13_0264	peg	3348_29638_28823	29638	28823	-	transposase	Transposase
270	3348	NEOS13_0265	peg	3348_30041_31246	30041	31246	+	hypothetical protein	
271	3348	NEOS13_0266	peg	3348_31373_31489	31373	31489	+	hypothetical protein	
272	3348	NEOS13_0267	peg	3348_32861_31464	32861	31464	-	High-affinity carbon uptake protein Hat/HatR	
273	3348	NEOS13_0268	peg	3348_34204_33263	34204	33263	-	hypothetical protein	
274	3348	NEOS13_0269	peg	3348_35109_34201	35109	34201	-	1,4-dihydroxy-2-naphthoate octaprenyltransferase (EC 2.5.1.74)	
275	3348	NEOS13_0270	peg	3348_35960_35130	35960	35130	-	Naphthoate synthase (EC 4.1.3.36)	
276	3348	NEOS13_0271	peg	3348_36664_35951	36664	35951	-	2-succinyl-6-hydroxy-2,4-cyclohexadiene-1-carboxylate synthase (EC 4.2.99.20)	
277	3348	NEOS13_0272	peg	3348_38259_36661	38259	36661	-	2-succinyl-5-enolpyruvyl-6-hydroxy-3-cyclohexene-1-carboxylic-acid synthase (EC 2.2.1.9)	
278	3348	NEOS13_0273	peg	3348_39326_38256	39326	38256	-	menaquinone-specific isochorismate synthase	
279	3348	NEOS13_0274	peg	3348_39987_39388	39987	39388	-	hypothetical protein	
280	3348	NEOS13_0275	peg	3348_40163_41161	40163	41161	+	hypothetical protein	
281	3348	NEOS13_0276	peg	3348_41401_42549	41401	42549	+	Branched-chain amino acid transport system carrier protein	

282	3348	NEOS13_0277	peg	3348_42657_43433	42657	43433	+	hypothetical protein	
283	3348	NEOS13_0278	peg	3348_44329_43943	44329	43943	-	hypothetical protein	
284	3348	NEOS13_0279	peg	3348_44458_45918	44458	45918	+	3'-to-5' exoribonuclease RNase R	
285	3348	NEOS13_0280	peg	3348_46110_45952	46110	45952	-	hypothetical protein	
286	3348	NEOS13_0281	peg	3348_46290_46150	46290	46150	-	hypothetical protein	
287	3348	NEOS13_0282	peg	3348_46448_46708	46448	46708	+	DNA-damage-inducible protein J	
288	3348	NEOS13_0283	peg	3348_46701_46967	46701	46967	+	hypothetical protein	
289	3348	NEOS13_0284	peg	3348_50114_47253	50114	47253	-	hypothetical protein	
290	3348	NEOS13_0285	peg	3348_50537_50710	50537	50710	+	hypothetical protein	
291	3348	NEOS13_0286	peg	3348_51077_54445	51077	54445	+	hypothetical protein	
292	3348	NEOS13_0287	peg	3348_55703_54699	55703	54699	-	DNA polymerase III delta prime subunit (EC 2.7.7.7)	
293	3348	NEOS13_0288	peg	3348_56426_55782	56426	55782	-	Thymidylate kinase (EC 2.7.4.9)	
294	3348	NEOS13_0289	peg	3348_59002_56423	59002	56423	-	DNA gyrase subunit A (EC 5.99.1.3)	
295	3348	NEOS13_0290	peg	3348_61536_59032	61536	59032	-	DNA gyrase subunit B (EC 5.99.1.3)	
296	3348	NEOS13_0291	peg	3348_61896_61558	61896	61558	-	hypothetical protein	
297	3348	NEOS13_0292	peg	3348_63066_62128	63066	62128	-	4-hydroxy-3-methylbut-2-enyl diphosphate reductase (EC 1.17.1.2)	
298	3348	NEOS13_0293	peg	3348_63708_63217	63708	63217	-	Low molecular weight protein tyrosine phosphatase (EC 3.1.3.48)	
299	3348	NEOS13_0294	peg	3348_65634_63718	65634	63718	-	Serine/threonine kinase	
300	3348	NEOS13_0295	peg	3348_66550_65645	66550	65645	-	hypothetical protein	
301	3348	NEOS13_0296	peg	3348_67058_69574	67058	69574	+	Inner membrane protein translocase component YidC, long form / Inner membrane protein translocase component YidC, short form OxaI-like	
302	3348	NEOS13_0297	peg	3348_69651_71024	69651	71024	+	Chromosomal replication initiator protein DnaA	
303	3348	NEOS13_0006ma	rna	3348_71411_71485	71411	71485	+	tRNA-Pro-TGG	RNA
304	3348	NEOS13_0298	peg	3348_71542_72438	71542	72438	+	Prolipoprotein diacylglyceryl transferase (EC 2.4.99.-)	
305	3348	NEOS13_0299	peg	3348_72549_72773	72549	72773	+	Ferredoxin	
306	3348	NEOS13_0300	peg	3348_72790_73371	72790	73371	+	hypothetical protein	
307	3348	NEOS13_0301	peg	3348_74761_73871	74761	73871	-	hypothetical protein	
308	3348	NEOS13_0302	peg	3348_75116_76582	75116	76582	+	hypothetical protein	
309	3350	NEOS13_0303	peg	3350_1797_799	1797	799	-	hypothetical protein	
310	3350	NEOS13_0304	peg	3350_4206_1822	4206	1822	-	Phenylalanyl-tRNA synthetase beta chain (EC 6.1.1.20)	
311	3350	NEOS13_0305	peg	3350_4874_4662	4874	4662	-	hypothetical protein	
312	3350	NEOS13_0306	peg	3350_5151_4945	5151	4945	-	hypothetical protein	
313	3350	NEOS13_0307	peg	3350_5296_5412	5296	5412	+	hypothetical protein	
314	3350	NEOS13_0308	peg	3350_6629_6441	6629	6441	-	hypothetical protein	
315	3351	NEOS13_0309	peg	3351_256_1890	256	1890	+	hypothetical protein	
316	3351	NEOS13_0310	peg	3351_3032_1959	3032	1959	-	Putative ATP:guanido phosphotransferase YacI (EC 2.7.3.-)	
317	3351	NEOS13_0311	peg	3351_3579_3019	3579	3019	-	FIG00493978: hypothetical protein	
318	3351	NEOS13_0007ma	rna	3351_4221_4149	4221	4149	-	tRNA-Lys-TTT	RNA
319	3351	NEOS13_0008ma	rna	3351_4303_4229	4303	4229	-	tRNA-Glu-TTC	RNA
320	3351	NEOS13_0312	peg	3351_4956_4405	4956	4405	-	Ribosome recycling factor	
321	3351	NEOS13_0313	peg	3351_5777_5007	5777	5007	-	Uridylate kinase (EC 2.7.4.-)	
322	3351	NEOS13_0314	peg	3351_6476_6003	6476	6003	-	hypothetical protein	
323	3351	NEOS13_0315	peg	3351_7532_6486	7532	6486	-	hypothetical protein	
324	3351	NEOS13_0316	peg	3351_8683_7754	8683	7754	-	Permease of the drug/metabolite transporter (DMT) superfamily	
325	3351	NEOS13_0317	peg	3351_12055_8816	12055	8816	-	hypothetical protein	
326	3351	NEOS13_0318	peg	3351_12214_12909	12214	12909	+	Phosphate regulon transcriptional regulatory protein PhoB (SphR)	
327	3351	NEOS13_0319	peg	3351_12977_13222	12977	13222	+	hypothetical protein	
328	3351	NEOS13_0320	peg	3351_13206_13349	13206	13349	+	hypothetical protein	
329	3351	NEOS13_0321	peg	3351_13703_13843	13703	13843	+	hypothetical protein	
330	3351	NEOS13_0322	peg	3351_14309_16063	14309	16063	+	Leucine-rich repeat containing protein	Leucine-rich repeat
331	3351	NEOS13_0323	peg	3351_16232_16089	16232	16089	-	hypothetical protein	
332	3351	NEOS13_0324	peg	3351_17740_16343	17740	16343	-	Fibronectin/fibrinogen-binding protein	
333	3351	NEOS13_0325	peg	3351_18925_17744	18925	17744	-	S-adenosylmethionine synthetase (EC 2.5.1.6)	
334	3351	NEOS13_0326	peg	3351_19956_19141	19956	19141	-	Glutamate racemase (EC 5.1.1.3)	
335	3351	NEOS13_0327	peg	3351_20510_19953	20510	19953	-	hypothetical protein	
336	3351	NEOS13_0328	peg	3351_21403_20675	21403	20675	-	Outer membrane lipoprotein omp16 precursor	
337	3351	NEOS13_0329	peg	3351_23180_21804	23180	21804	-	tolB protein precursor, periplasmic protein involved in the tonb-independent uptake of group A colicins	
338	3351	NEOS13_0330	peg	3351_24205_23177	24205	23177	-	putative tolA protein of Tol-Pal system	
339	3351	NEOS13_0331	peg	3351_24722_24291	24722	24291	-	Biopolymer transport protein ExbD/TolR	
340	3351	NEOS13_0332	peg	3351_25468_24719	25468	24719	-	MotA/TolQ/ExbB proton channel family protein	
341	3351	NEOS13_0333	peg	3351_25564_27774	25564	27774	+	Cytochrome c-type biogenesis protein DsbD, protein-disulfide reductase (EC 1.8.1.8)	
342	3351	NEOS13_0334	peg	3351_28281_29441	28281	29441	+	Leucine-rich repeat containing protein	Leucine-rich repeat
343	3351	NEOS13_0335	peg	3351_29727_29960	29727	29960	+	hypothetical protein	
344	3351	NEOS13_0336	peg	3351_30061_30189	30061	30189	+	hypothetical protein	
345	3351	NEOS13_0337	peg	3351_30289_30423	30289	30423	+	hypothetical protein	
346	3351	NEOS13_0338	peg	3351_33166_30500	33166	30500	-	hypothetical protein	

347	3351	NEOS13_0339	peg	3351_33429_34622	33429	34622	+	Heavy metal RND efflux outer membrane protein, CzcC family	
348	3351	NEOS13_0340	peg	3351_34647_35783	34647	35783	+	hypothetical protein	
349	3351	NEOS13_0341	peg	3351_35776_38997	35776	38997	+	Cobalt-zinc-cadmium resistance protein CzcA; Cation efflux system protein CusA	
350	3351	NEOS13_0342	peg	3351_39577_41184	39577	41184	+	hypothetical protein	
351	3351	NEOS13_0343	peg	3351_42519_41488	42519	41488	-	Ferrochelatase, protoheme ferro-lyase (EC 4.99.1.1)	
352	3351	NEOS13_0344	peg	3351_42719_42522	42719	42522	-	hypothetical protein	
353	3351	NEOS13_0345	peg	3351_44355_42706	44355	42706	-	Outer membrane protein romA	
354	3351	NEOS13_0346	peg	3351_44978_44367	44978	44367	-	Phosphoserine phosphatase (EC 3.1.3.3)	
355	3351	NEOS13_0347	peg	3351_45068_46030	45068	46030	+	probable UDP-glucuronat epimerase	
356	3351	NEOS13_0348	peg	3351_46176_46796	46176	46796	+	hypothetical protein	
357	3351	NEOS13_0349	peg	3351_46898_48772	46898	48772	+	hypothetical protein	
358	3351	NEOS13_0350	peg	3351_49853_49089	49853	49089	-	protein of unknown function DUF152	
359	3351	NEOS13_0351	peg	3351_50097_52277	50097	52277	+	ATP-dependent DNA helicase UvrD/PcrA	
360	3351	NEOS13_0352	peg	3351_59243_52350	59243	52350	-	hypothetical protein	
361	3351	NEOS13_0353	peg	3351_59449_59303	59449	59303	-	hypothetical protein	
362	3352	NEOS13_0354	peg	3352_20_1213	20	1213	+	Leucine-rich repeat containing protein	Leucine-rich repeat
363	3352	NEOS13_0355	peg	3352_1665_1790	1665	1790	+	hypothetical protein	
364	3352	NEOS13_0356	peg	3352_2088_2294	2088	2294	+	Mobile element protein	
365	3352	NEOS13_0357	peg	3352_2563_2790	2563	2790	+	Mobile element protein	
366	3352	NEOS13_0358	peg	3352_3960_2917	3960	2917	-	hypothetical protein	
367	3352	NEOS13_0359	peg	3352_5281_4103	5281	4103	-	hypothetical protein	
368	3352	NEOS13_0360	peg	3352_5933_6076	5933	6076	+	hypothetical protein	
369	3352	NEOS13_0361	peg	3352_6079_6258	6079	6258	+	hypothetical protein	
370	3352	NEOS13_0362	peg	3352_6617_6928	6617	6928	+	transposase, IS4 family protein	Transposase
371	3352	NEOS13_0363	peg	3352_6969_7121	6969	7121	+	hypothetical protein	
372	3352	NEOS13_0364	peg	3352_9404_7839	9404	7839	-	FIG047302: Type III secretion S/T Protein Kinase	T3SA3
373	3352	NEOS13_0365	peg	3352_10811_9489	10811	9489	-	Flagellar motor switch protein FliN	T3SA3
374	3352	NEOS13_0366	peg	3352_12141_11167	12141	11167	-	hypothetical protein	T3SA3
375	3352	NEOS13_0367	peg	3352_12685_12197	12685	12197	-	FIG016940: Type III secretion protein	T3SA3
376	3352	NEOS13_0368	peg	3352_14041_12725	14041	12725	-	Type III secretion cytoplasmic ATP synthase (EC 3.6.3.14, YscN,SpaL,MxiB,HrcN,EscN)	T3SA3
377	3352	NEOS13_0369	peg	3352_14821_14141	14821	14141	-	hypothetical protein	T3SA3
378	3352	NEOS13_0370	peg	3352_15363_14890	15363	14890	-	FIG016921: Type III secretion protein	T3SA3
379	3352	NEOS13_0371	peg	3352_15779_15486	15779	15486	-	unknown protein	T3SA3
380	3352	NEOS13_0372	peg	3352_16229_15990	16229	15990	-	FIG016943: Type III secretion	T3SA3
381	3352	NEOS13_0373	peg	3352_18768_16351	18768	16351	-	FIG016943: Forkhead domain protein	T3SA3
382	3352	NEOS13_0374	peg	3352_19205_18813	19205	18813	-	hypothetical protein	T3SA3
383	3353	NEOS13_0375	peg	3353_210_326	210	326	+	hypothetical protein	
384	3353	NEOS13_0376	peg	3353_486_854	486	854	+	hypothetical protein	
385	3353	NEOS13_0377	peg	3353_899_1018	899	1018	+	hypothetical protein	
386	3353	NEOS13_0378	peg	3353_1148_1336	1148	1336	+	hypothetical protein	
387	3353	NEOS13_0379	peg	3353_1958_1833	1958	1833	-	hypothetical protein	
388	3353	NEOS13_0380	peg	3353_2488_4377	2488	4377	+	Acetyl-coenzyme A synthetase (EC 6.2.1.1)	
389	3353	NEOS13_0381	peg	3353_4882_4436	4882	4436	-	hypothetical protein	
390	3353	NEOS13_0382	peg	3353_5488_4886	5488	4886	-	hypothetical protein	
391	3353	NEOS13_0383	peg	3353_5925_5491	5925	5491	-	hypothetical protein	
392	3353	NEOS13_0384	peg	3353_6507_5998	6507	5998	-	hypothetical protein	
393	3353	NEOS13_0385	peg	3353_7713_6643	7713	6643	-	protein of unknown function UPF0118	
394	3353	NEOS13_0386	peg	3353_7937_7815	7937	7815	-	hypothetical protein	
395	3353	NEOS13_0387	peg	3353_9274_8129	9274	8129	-	LSU m5C1962 methyltransferase RlmI	
396	3353	NEOS13_0388	peg	3353_9650_9321	9650	9321	-	COG2363	
397	3353	NEOS13_0389	peg	3353_11601_10210	11601	10210	-	Dihydrolipoamide dehydrogenase of 2-oxoglutarate dehydrogenase (EC 1.8.1.4)	TCA
398	3353	NEOS13_0390	peg	3353_11642_11767	11642	11767	+	hypothetical protein	
399	3353	NEOS13_0391	peg	3353_12978_11845	12978	11845	-	Dihydrolipoamide succinyltransferase component (E2) of 2-oxoglutarate dehydrogenase complex (EC 2.3.1.61)	TCA
400	3353	NEOS13_0392	peg	3353_13718_13557	13718	13557	-	hypothetical protein	
401	3353	NEOS13_0393	peg	3353_15092_14247	15092	14247	-	Leucine-rich repeat containing protein	Leucine-rich repeat
402	3353	NEOS13_0394	peg	3353_15295_15140	15295	15140	-	Leucine-rich repeat containing protein	Leucine-rich repeat
403	3353	NEOS13_0395	peg	3353_15484_15344	15484	15344	-	hypothetical protein	
404	3353	NEOS13_0396	peg	3353_17177_15486	17177	15486	-	Leucine-rich repeat containing protein	Leucine-rich repeat
405	3353	NEOS13_0397	peg	3353_17815_17531	17815	17531	-	hypothetical protein	
406	3358	NEOS13_0398	peg	3358_541_164	541	164	-	Mobile element protein	
407	3358	NEOS13_0399	peg	3358_1081_1821	1081	1821	+	hypothetical protein	
408	3358	NEOS13_0400	peg	3358_1818_2666	1818	2666	+	hypothetical protein	
409	3358	NEOS13_0401	peg	3358_2670_2795	2670	2795	+	hypothetical protein	
410	3358	NEOS13_0402	peg	3358_2853_4922	2853	4922	+	hypothetical protein	
411	3358	NEOS13_0403	peg	3358_4948_5862	4948	5862	+	UDP-glucose 4-epimerase (EC 5.1.3.2)	
412	3358	NEOS13_0404	peg	3358_6158_6547	6158	6547	+	hypothetical protein	
413	3358	NEOS13_0405	peg	3358_6874_7710	6874	7710	+	O-antigen export system, permease protein	ABC_transporter
414	3358	NEOS13_0406	peg	3358_7711_8529	7711	8529	+	Teichoic acid export ATP-binding protein TagH (EC 3.6.3.40)	ABC_transporter
415	3358	NEOS13_0407	peg	3358_8530_9825	8530	9825	+	hypothetical protein	
416	3358	NEOS13_0408	peg	3358_9809_10756	9809	10756	+	Alpha-L-Rha alpha-1,3-L-rhamnosyltransferase (EC 2.4.1.-)	
417	3358	NEOS13_0409	peg	3358_10825_12099	10825	12099	+	hypothetical protein	

418	3358	NEOS13_0410	peg	3358_12092_13927	12092	13927	+	glycosyl transferase, group 2 family protein	
419	3358	NEOS13_0411	peg	3358_13958_15415	13958	15415	+	Probable poly(beta-D-mannuronate) O-acetylase (EC 2.3.1.-)	
420	3358	NEOS13_0412	peg	3358_15417_16559	15417	16559	+	hypothetical protein	
421	3358	NEOS13_0413	peg	3358_17684_16851	17684	16851	-	hypothetical protein	
422	3358	NEOS13_0414	peg	3358_17813_18403	17813	18403	+	Phosphoglycolate phosphatase (EC 3.1.3.18)	
423	3358	NEOS13_0415	peg	3358_18435_18638	18435	18638	+	hypothetical protein	
424	3358	NEOS13_0416	peg	3358_18836_18702	18836	18702	-	hypothetical protein	
425	3358	NEOS13_0417	peg	3358_19539_21698	19539	21698	+	hypothetical protein	
426	3358	NEOS13_0418	peg	3358_21711_21917	21711	21917	+	hypothetical protein	
427	3358	NEOS13_0419	peg	3358_22192_21923	22192	21923	-	hypothetical protein	
428	3358	NEOS13_0420	peg	3358_22879_22484	22879	22484	-	Mobile element protein	
429	3358	NEOS13_0421	peg	3358_23450_23322	23450	23322	-	hypothetical protein	
430	3358	NEOS13_0422	peg	3358_23445_25205	23445	25205	+	Leucine-rich repeat containing protein	Leucine-rich repeat
431	3359	NEOS13_0423	peg	3359_1375_302	1375	302	-	Leucine-rich repeat containing protein	Leucine-rich repeat
432	3360	NEOS13_0424	peg	3360_1050_349	1050	349	-	hypothetical protein	
433	3360	NEOS13_0425	peg	3360_2592_1126	2592	1126	-	Mg/Co/Ni transporter MgtE / CBS domain	
434	3360	NEOS13_0426	peg	3360_2784_4586	2784	4586	+	GTP-binding protein TypA/BipA	
435	3360	NEOS13_0427	peg	3360_4856_4731	4856	4731	-	hypothetical protein	
436	3360	NEOS13_0428	peg	3360_5087_4938	5087	4938	-	hypothetical protein	
437	3360	NEOS13_0429	peg	3360_6270_5629	6270	5629	-	FIG006285: hypothetical protein	
438	3360	NEOS13_0430	peg	3360_8678_6267	8678	6267	-	FIG003033: Helicase domain protein	
439	3360	NEOS13_0431	peg	3360_10260_8668	10260	8668	-	ATP-dependent DNA ligase (EC 6.5.1.1) LigC	
440	3360	NEOS13_0432	peg	3360_11183_10257	11183	10257	-	mRNA 3-end processing factor	
441	3360	NEOS13_0433	peg	3360_11291_11410	11291	11410	+	hypothetical protein	
442	3360	NEOS13_0434	peg	3360_13650_11566	13650	11566	-	hypothetical protein	
443	3360	NEOS13_0435	peg	3360_16952_14934	16952	14934	-	ankyrin repeat protein	Ankyrin
444	3360	NEOS13_0436	peg	3360_18167_18313	18167	18313	+	hypothetical protein	
445	3360	NEOS13_0437	peg	3360_18403_18660	18403	18660	+	Mobile element protein	
446	3360	NEOS13_0438	peg	3360_19418_18681	19418	18681	-	Mobile element protein	
447	3360	NEOS13_0439	peg	3360_19579_19415	19579	19415	-	hypothetical protein	
448	3360	NEOS13_0440	peg	3360_19934_19605	19934	19605	-	IS1478 transposase	Transposase
449	3360	NEOS13_0441	peg	3360_20831_20538	20831	20538	-	hypothetical protein	
450	3360	NEOS13_0442	peg	3360_20877_20996	20877	20996	+	hypothetical protein	
451	3360	NEOS13_0443	peg	3360_22372_20993	22372	20993	-	putative zinc protease	
452	3360	NEOS13_0009rna	rna	3360_22534_22615	22534	22615	+	tRNA-Leu-TAG	RNA
453	3360	NEOS13_0444	peg	3360_22782_22648	22782	22648	-	hypothetical protein	
454	3360	NEOS13_0445	peg	3360_23168_22974	23168	22974	-	hypothetical protein	
455	3361	NEOS13_0446	peg	3361_82_1269	82	1269	+	Leucine-rich repeat containing protein	Leucine-rich repeat
456	3361	NEOS13_0447	peg	3361_1681_1517	1681	1517	-	hypothetical protein	
457	3361	NEOS13_0448	peg	3361_1814_1674	1814	1674	-	Mobile element protein	
458	3361	NEOS13_0449	peg	3361_2006_1875	2006	1875	-	hypothetical protein	
459	3361	NEOS13_0450	peg	3361_2151_1981	2151	1981	-	Mobile element protein	
460	3361	NEOS13_0451	peg	3361_2449_2255	2449	2255	-	Mobile element protein	
461	3361	NEOS13_0452	peg	3361_3144_3287	3144	3287	+	hypothetical protein	
462	3361	NEOS13_0453	peg	3361_3284_4294	3284	4294	+	hypothetical protein	
463	3361	NEOS13_0454	peg	3361_4816_6045	4816	6045	+	hypothetical protein	
464	3361	NEOS13_0455	peg	3361_6566_6396	6566	6396	-	hypothetical protein	
465	3361	NEOS13_0456	peg	3361_6852_6643	6852	6643	-	hypothetical protein	
466	3361	NEOS13_0457	peg	3361_6923_7036	6923	7036	+	hypothetical protein	
467	3361	NEOS13_0458	peg	3361_7619_7287	7619	7287	-	hypothetical protein	
468	3361	NEOS13_0459	peg	3361_7753_7878	7753	7878	+	hypothetical protein	
469	3361	NEOS13_0460	peg	3361_8932_7946	8932	7946	-	putative tyrosine/tryptophan transport protein	
470	3361	NEOS13_0461	peg	3361_8922_9155	8922	9155	+	hypothetical protein	
471	3361	NEOS13_0462	peg	3361_9438_10355	9438	10355	+	hypothetical protein	
472	3361	NEOS13_0463	peg	3361_10549_11643	10549	11643	+	hypothetical protein	
473	3361	NEOS13_0464	peg	3361_11667_14630	11667	14630	+	putative Tyrosine-protein kinase	
474	3361	NEOS13_0465	peg	3361_15679_14852	15679	14852	-	NADPH dependent preQ0 reductase (EC 1.7.1.13)	
475	3361	NEOS13_0466	peg	3361_19752_16360	19752	16360	-	Penicillin-binding protein PBP2	
476	3361	NEOS13_0010rna	rna	3361_19880_19950	19880	19950	+	tRNA-Gly-TCC	RNA
477	3361	NEOS13_0467	peg	3361_20124_20846	20124	20846	+	SSU ribosomal protein S2p (SAe)	
478	3361	NEOS13_0468	peg	3361_20850_21704	20850	21704	+	Translation elongation factor Ts	
479	3361	NEOS13_0469	peg	3361_21867_22742	21867	22742	+	Permease of the drug/metabolite transporter (DMT) superfamily	
480	3361	NEOS13_0470	peg	3361_23664_22921	23664	22921	-	hypothetical protein	
481	3361	NEOS13_0471	peg	3361_25170_23728	25170	23728	-	hypothetical protein	
482	3362	NEOS13_0472	peg	3362_88_219	88	219	+	hypothetical protein	
483	3362	NEOS13_0473	peg	3362_784_1380	784	1380	+	hypothetical protein	
484	3362	NEOS13_0474	peg	3362_1377_2009	1377	2009	+	hypothetical protein	
485	3362	NEOS13_0475	peg	3362_2186_2013	2186	2013	-	hypothetical protein	
486	3363	NEOS13_0476	peg	3363_226_59	226	59	-	hypothetical protein	
487	3363	NEOS13_0477	peg	3363_710_570	710	570	-	hypothetical protein	
488	3363	NEOS13_0478	peg	3363_1278_2942	1278	2942	+	Lipoprotein releasing system transmembrane protein LolE	ABC_transporter
489	3363	NEOS13_0479	peg	3363_2944_3636	2944	3636	+	Lipoprotein releasing system ATP-binding protein LolD	ABC_transporter
490	3363	NEOS13_0480	peg	3363_3858_3983	3858	3983	+	hypothetical protein	
491	3363	NEOS13_0481	peg	3363_4032_4184	4032	4184	+	hypothetical protein	
492	3364	NEOS13_0482	peg	3364_15_1199	15	1199	+	Leucine-rich repeat containing protein	Leucine-rich repeat
493	3365	NEOS13_0483	peg	3365_585_746	585	746	+	hypothetical protein	

494	3366	NEOS13_0484	peg	3366_265_540	265	540	+	hypothetical protein	
495	3366	NEOS13_0485	peg	3366_2037_850	2037	850	-	Leucine-rich repeat containing protein	Leucine-rich repeat
496	3367	NEOS13_0011ma	rna	3367_1608_70	1608	70	-	Small Subunit Ribosomal RNA; ssuRNA; SSU rRNA	RNA
497	3367	NEOS13_0486	peg	3367_5660_3537	5660	3537	-	Adenosine deaminase (EC 3.5.4.4)	
498	3367	NEOS13_0487	peg	3367_7828_5849	7828	5849	-	Pyrophosphate-energized proton pump (EC 3.6.1.1)	
499	3367	NEOS13_0488	peg	3367_7962_8078	7962	8078	+	hypothetical protein	
500	3367	NEOS13_0489	peg	3367_9319_8036	9319	8036	-	Error-prone, lesion bypass DNA polymerase V (UmuC)	
501	3367	NEOS13_0490	peg	3367_10256_10131	10256	10131	-	hypothetical protein	
502	3367	NEOS13_0491	peg	3367_10580_12385	10580	12385	+	COG0488: ATPase components of ABC transporters with duplicated ATPase domains	ABC_transporter
503	3367	NEOS13_0492	peg	3367_13178_12837	13178	12837	-	membrane protein of unknown function	
504	3367	NEOS13_0493	peg	3367_13868_13746	13868	13746	-	Mobile element protein	
505	3367	NEOS13_0494	peg	3367_14067_13936	14067	13936	-	hypothetical protein	
506	3367	NEOS13_0495	peg	3367_15520_14495	15520	14495	-	Leucine-rich repeat containing protein	Leucine-rich repeat
507	3368	NEOS13_0496	peg	3368_49_186	49	186	+	hypothetical protein	
508	3369	NEOS13_0497	peg	3369_107_1165	107	1165	+	Leucine-rich repeat containing protein	Leucine-rich repeat
509	3369	NEOS13_0498	peg	3369_2040_3353	2040	3353	+	Leucine-rich repeat containing protein	Leucine-rich repeat
510	3372	NEOS13_0499	peg	3372_22_168	22	168	+	hypothetical protein	
511	3372	NEOS13_0500	peg	3372_380_126	380	126	-	Mobile element protein	
512	3372	NEOS13_0501	peg	3372_636_869	636	869	+	hypothetical protein	
513	3372	NEOS13_0502	peg	3372_1120_920	1120	920	-	hypothetical protein	
514	3372	NEOS13_0503	peg	3372_1242_1379	1242	1379	+	hypothetical protein	
515	3372	NEOS13_0504	peg	3372_1475_2548	1475	2548	+	hypothetical protein	
516	3372	NEOS13_0505	peg	3372_2703_3974	2703	3974	+	hypothetical protein	
517	3372	NEOS13_0506	peg	3372_4011_4703	4011	4703	+	hypothetical protein	
518	3372	NEOS13_0507	peg	3372_5067_4927	5067	4927	-	hypothetical protein	
519	3373	NEOS13_0508	peg	3373_1734_961	1734	961	-	Leucine-rich repeat containing protein	Leucine-rich repeat
520	3374	NEOS13_0509	peg	3374_29_1651	29	1651	+	Leucine-rich repeat containing protein	Leucine-rich repeat
521	3374	NEOS13_0510	peg	3374_1887_1672	1887	1672	-	Mobile element protein	
522	3374	NEOS13_0511	peg	3374_2605_2483	2605	2483	-	hypothetical protein	
523	3374	NEOS13_0512	peg	3374_3050_2931	3050	2931	-	hypothetical protein	
524	3374	NEOS13_0513	peg	3374_4094_3507	4094	3507	-	hypothetical protein	Outer_membrane_protein
525	3374	NEOS13_0514	peg	3374_6012_4087	6012	4087	-	internalin, putative	
526	3374	NEOS13_0515	peg	3374_6278_6439	6278	6439	+	hypothetical protein	
527	3375	NEOS13_0516	peg	3375_118_1035	118	1035	+	Leucine-rich repeat containing protein	Leucine-rich repeat
528	3376	NEOS13_0517	peg	3376_57_272	57	272	+	hypothetical protein	
529	3376	NEOS13_0518	peg	3376_1377_604	1377	604	-	Leucine-rich repeat containing protein	Leucine-rich repeat
530	3377	NEOS13_0519	peg	3377_427_233	427	233	-	hypothetical protein	
531	3377	NEOS13_0520	peg	3377_952_593	952	593	-	Leucine-rich repeat containing protein	Leucine-rich repeat
532	3378	NEOS13_0521	peg	3378_678_403	678	403	-	hypothetical protein	
533	3379	NEOS13_0522	peg	3379_747_541	747	541	-	conserved hypothetical protein	
534	3379	NEOS13_0523	peg	3379_1055_747	1055	747	-	conserved hypothetical protein	
535	3379	NEOS13_0524	peg	3379_1275_1117	1275	1117	-	hypothetical protein	
536	3379	NEOS13_0525	peg	3379_1529_1254	1529	1254	-	hypothetical protein	
537	3379	NEOS13_0526	peg	3379_1489_1617	1489	1617	+	hypothetical protein	
538	3379	NEOS13_0527	peg	3379_1851_1666	1851	1666	-	hypothetical protein	
539	3379	NEOS13_0528	peg	3379_1959_2078	1959	2078	+	hypothetical protein	
540	3380	NEOS13_0529	peg	3380_2699_324	2699	324	-	hypothetical protein	
541	3380	NEOS13_0530	peg	3380_3076_2909	3076	2909	-	hypothetical protein	
542	3381	NEOS13_0531	peg	3381_19_741	19	741	+	hypothetical protein	Outer_membrane_protein
543	3381	NEOS13_0532	peg	3381_1069_1185	1069	1185	+	hypothetical protein	
544	3381	NEOS13_0533	peg	3381_2203_1235	2203	1235	-	Ribosomal large subunit pseudouridine synthase D (EC 4.2.1.70)	
545	3381	NEOS13_0534	peg	3381_2612_2268	2612	2268	-	hypothetical protein	
546	3381	NEOS13_0535	peg	3381_2837_2619	2837	2619	-	hypothetical protein	
547	3381	NEOS13_0536	peg	3381_2755_2949	2755	2949	+	hypothetical protein	
548	3381	NEOS13_0537	peg	3381_3358_3083	3358	3083	-	hypothetical protein	
549	3381	NEOS13_0538	peg	3381_4317_3709	4317	3709	-	hypothetical protein	
550	3381	NEOS13_0539	peg	3381_6215_4335	6215	4335	-	Topoisomerase IV subunit A (EC 5.99.1.-)	
551	3381	NEOS13_0540	peg	3381_8220_6424	8220	6424	-	Topoisomerase IV subunit B (EC 5.99.1.-)	
552	3381	NEOS13_0541	peg	3381_8394_9413	8394	9413	+	UDP-N-acetylglucosamine 4,6-dehydratase (EC 4.2.1.-)	
553	3381	NEOS13_0542	peg	3381_9468_10496	9468	10496	+	Glutamyl-tRNA reductase (EC 1.2.1.70)	
554	3381	NEOS13_0543	peg	3381_10578_12170	10578	12170	+	hypothetical protein	
555	3381	NEOS13_0544	peg	3381_13359_12628	13359	12628	-	putative lipoate-protein ligase	
556	3381	NEOS13_0545	peg	3381_15211_13352	15211	13352	-	tRNA uridine 5-carboxymethylaminomethyl modification enzyme GidA	
557	3381	NEOS13_0546	peg	3381_17207_15762	17207	15762	-	Replicative DNA helicase (EC 3.6.1.-)	
558	3381	NEOS13_0547	peg	3381_17614_19098	17614	19098	+	RNA polymerase sigma-54 factor RpoN	
559	3381	NEOS13_0548	peg	3381_19654_21324	19654	21324	+	Sulfate permease	
560	3381	NEOS13_0549	peg	3381_21919_21767	21919	21767	-	Mobile element protein	
561	3381	NEOS13_0550	peg	3381_22195_22317	22195	22317	+	hypothetical protein	
562	3381	NEOS13_0551	peg	3381_22498_22623	22498	22623	+	hypothetical protein	
563	3381	NEOS13_0552	peg	3381_22605_24203	22605	24203	+	Leucine-rich repeat containing protein	Leucine-rich repeat
564	3381	NEOS13_0553	peg	3381_24223_24366	24223	24366	+	hypothetical protein	
565	3383	NEOS13_0554	peg	3383_376_702	376	702	+	hypothetical protein	
566	3383	NEOS13_0555	peg	3383_965_1081	965	1081	+	hypothetical protein	
567	3385	NEOS13_0556	peg	3385_293_901	293	901	+	hypothetical protein	
568	3385	NEOS13_0557	peg	3385_1765_1631	1765	1631	-	hypothetical protein	

569	3386	NEOS13_0558	peg	3386_48_668	48	668	+	Leucine-rich repeat containing protein	Leucine-rich repeat
570	3387	NEOS13_0559	peg	3387_48_668	48	668	+	Leucine-rich repeat containing protein	Leucine-rich repeat
571	3388	NEOS13_0560	peg	3388_1723_1571	1723	1571	-	Leucine-rich repeat containing protein	Leucine-rich repeat
572	3389	NEOS13_0561	peg	3389_336_70	336	70	-	hypothetical protein	
573	3389	NEOS13_0562	peg	3389_5416_413	5416	413	-	hypothetical protein	
574	3389	NEOS13_0563	peg	3389_9581_5802	9581	5802	-	hypothetical protein	
575	3389	NEOS13_0564	peg	3389_9861_9697	9861	9697	-	hypothetical protein	
576	3389	NEOS13_0565	peg	3389_10058_11161	10058	11161	+	UDP-N-acetylglucosamine--N-acetylmuramyl-(pentapeptide) pyrophosphoryl-undecaprenol N-acetylglucosamine transferase (EC 2.4.1.227)	
577	3389	NEOS13_0566	peg	3389_11158_13593	11158	13593	+	UDP-N-acetylmuramate--alanine ligase (EC 6.3.2.8)	
578	3389	NEOS13_0567	peg	3389_13788_14690	13788	14690	+	hypothetical protein	
579	3389	NEOS13_0568	peg	3389_14915_15193	14915	15193	+	hypothetical protein	
580	3389	NEOS13_0569	peg	3389_15586_15705	15586	15705	+	hypothetical protein	
581	3389	NEOS13_0570	peg	3389_16248_17279	16248	17279	+	tRNA dimethylallyltransferase (EC 2.5.1.75)	
582	3389	NEOS13_0571	peg	3389_17374_19095	17374	19095	+	hypothetical protein	
583	3389	NEOS13_0572	peg	3389_19058_19732	19058	19732	+	hypothetical protein	
584	3389	NEOS13_0573	peg	3389_20359_20994	20359	20994	+	Nicotinate-nucleotide adenylyltransferase (EC 2.7.7.18)	
585	3389	NEOS13_0574	peg	3389_21009_21374	21009	21374	+	Iojap protein	
586	3389	NEOS13_0575	peg	3389_21392_22639	21392	22639	+	3-oxoacyl-[acyl-carrier-protein] synthase, KASII (EC 2.3.1.41)	Fatty _acid_initiation_elongation
587	3389	NEOS13_0576	peg	3389_22639_23061	22639	23061	+	putative dGTP pyrophosphohydrolase, mutT	
588	3389	NEOS13_0577	peg	3389_23668_23411	23668	23411	-	Antitoxin of toxin-antitoxin system Phd	
589	3389	NEOS13_0578	peg	3389_24295_24059	24295	24059	-	hypothetical protein	
590	3389	NEOS13_0579	peg	3389_24645_24496	24645	24496	-	hypothetical protein	
591	3390	NEOS13_0580	peg	3390_1211_381	1211	381	-	Mobile element protein	
592	3391	NEOS13_0581	peg	3391_42_1190	42	1190	+	hypothetical protein	
593	3392	NEOS13_0582	peg	3392_60_263	60	263	+	hypothetical protein	
594	3392	NEOS13_0583	peg	3392_1150_2226	1150	2226	+	Branched-chain alpha-keto acid dehydrogenase, E1 component, alpha subunit (EC 1.2.4.4)	
595	3392	NEOS13_0584	peg	3392_2256_3233	2256	3233	+	Branched-chain alpha-keto acid dehydrogenase, E1 component, beta subunit (EC 1.2.4.4)	Glycolysis_Glucogenesis
596	3392	NEOS13_0585	peg	3392_3246_4472	3246	4472	+	Dihydrolipoamide acyltransferase component of branched-chain alpha-keto acid dehydrogenase complex (EC 2.3.1.168)	Glycolysis_Glucogenesis
597	3392	NEOS13_0586	peg	3392_4435_4572	4435	4572	+	hypothetical protein	
598	3392	NEOS13_0587	peg	3392_4726_6417	4726	6417	+	Potassium-transporting ATPase A chain (EC 3.6.3.12) (TC 3.A.3.7.1)	
599	3392	NEOS13_0588	peg	3392_6488_8584	6488	8584	+	Potassium-transporting ATPase B chain (EC 3.6.3.12) (TC 3.A.3.7.1)	
600	3392	NEOS13_0589	peg	3392_8622_9182	8622	9182	+	Potassium-transporting ATPase C chain (EC 3.6.3.12) (TC 3.A.3.7.1)	
601	3392	NEOS13_0590	peg	3392_10789_12501	10789	12501	+	hypothetical protein	
602	3392	NEOS13_0591	peg	3392_12967_14544	12967	14544	+	Leucine-rich repeat containing protein	Leucine-rich repeat
603	3392	NEOS13_0592	peg	3392_15297_15172	15297	15172	-	hypothetical protein	
604	3392	NEOS13_0593	peg	3392_15614_15495	15614	15495	-	hypothetical protein	
605	3392	NEOS13_0594	peg	3392_16026_15802	16026	15802	-	hypothetical protein	
606	3392	NEOS13_0595	peg	3392_16346_17755	16346	17755	+	hypothetical protein	
607	3392	NEOS13_0596	peg	3392_19313_18027	19313	18027	-	RNA methyltransferase, TrmA family	
608	3392	NEOS13_0597	peg	3392_21063_19306	21063	19306	-	Single-stranded-DNA-specific exonuclease RecJ (EC 3.1.-.-)	
609	3392	NEOS13_0598	peg	3392_26196_21622	26196	21622	-	Protein-export membrane protein SecD (TC 3.A.5.1.1) / Protein-export membrane protein SecF (TC 3.A.5.1.1)	Sec_T2SS
610	3392	NEOS13_0599	peg	3392_28129_26576	28129	26576	-	ADP/ATP Translocase, NTT1	NTT
611	3392	NEOS13_0600	peg	3392_29321_28806	29321	28806	-	FIG00493911: hypothetical protein	
612	3392	NEOS13_0601	peg	3392_29674_30789	29674	30789	+	hypothetical protein	
613	3392	NEOS13_0602	peg	3392_30786_31778	30786	31778	+	Manganese ABC transporter, periplasmic-binding protein SitA	ABC_transporter
614	3392	NEOS13_0603	peg	3392_31775_32563	31775	32563	+	Manganese ABC transporter, ATP-binding protein SitB	ABC_transporter
615	3392	NEOS13_0604	peg	3392_32568_33920	32568	33920	+	Manganese ABC transporter, inner membrane permease protein SitC	ABC_transporter
616	3392	NEOS13_0605	peg	3392_33917_34927	33917	34927	+	Manganese ABC transporter, inner membrane permease protein SitD	ABC_transporter
617	3392	NEOS13_0606	peg	3392_35711_34941	35711	34941	-	hypothetical protein	
618	3392	NEOS13_0607	peg	3392_36382_36263	36382	36263	-	hypothetical protein	
619	3393	NEOS13_0608	peg	3393_1282_8	1282	8	-	Leucine-rich repeat containing protein	Leucine-rich repeat
620	3393	NEOS13_0609	peg	3393_1638_1345	1638	1345	-	hypothetical protein	
621	3393	NEOS13_0610	peg	3393_1673_1786	1673	1786	+	hypothetical protein	
622	3393	NEOS13_0611	peg	3393_2101_1886	2101	1886	-	hypothetical protein	
623	3393	NEOS13_0612	peg	3393_2088_2477	2088	2477	+	hypothetical protein	
624	3393	NEOS13_0613	peg	3393_2495_3331	2495	3331	+	hypothetical protein	
625	3393	NEOS13_0614	peg	3393_4020_3427	4020	3427	-	DNA-3-methyladenine glycosylase (EC 3.2.2.20)	
626	3393	NEOS13_0615	peg	3393_4152_6374	4152	6374	+	hypothetical protein	
627	3393	NEOS13_0616	peg	3393_6857_7534	6857	7534	+	Aquaporin Z	
628	3393	NEOS13_0617	peg	3393_7757_7572	7757	7572	-	hypothetical protein	
629	3393	NEOS13_0618	peg	3393_8142_7900	8142	7900	-	hypothetical protein	

630	3393	NEOS13_0619	peg	3393_8276_10453	8276	10453	+	POTASSIUM/PROTON ANTIPORTER ROSB	
631	3393	NEOS13_0620	peg	3393_10605_10462	10605	10462	-	hypothetical protein	
632	3393	NEOS13_0621	peg	3393_11458_12309	11458	12309	+	FIG01045360: hypothetical protein	
633	3393	NEOS13_0622	peg	3393_12296_12457	12296	12457	+	hypothetical protein	
634	3393	NEOS13_0623	peg	3393_12556_12678	12556	12678	+	hypothetical protein	
635	3393	NEOS13_0624	peg	3393_12994_14454	12994	14454	+	hypothetical protein	
636	3393	NEOS13_0625	peg	3393_14544_25139	14544	25139	+	hypothetical protein	
637	3393	NEOS13_0626	peg	3393_25230_27572	25230	27572	+	hypothetical protein	
638	3393	NEOS13_0627	peg	3393_28381_28220	28381	28220	-	hypothetical protein	
639	3394	NEOS13_0628	peg	3394_626_474	626	474	-	Leucine-rich repeat containing protein	Leucine-rich repeat
640	3394	NEOS13_0629	peg	3394_1317_673	1317	673	-	Leucine-rich repeat containing protein	Leucine-rich repeat
641	3395	NEOS13_0630	peg	3395_1025_486	1025	486	-	hypothetical protein	
642	3395	NEOS13_0631	peg	3395_1644_1147	1644	1147	-	hypothetical protein	
643	3395	NEOS13_0632	peg	3395_3210_3395	3210	3395	+	Mobile element protein	
644	3395	NEOS13_0633	peg	3395_4039_4248	4039	4248	+	Integrase, catalytic region	
645	3395	NEOS13_0634	peg	3395_4651_5802	4651	5802	+	hypothetical protein	
646	3396	NEOS13_0635	peg	3396_915_1457	915	1457	+	hypothetical protein	
647	3396	NEOS13_0636	peg	3396_1674_1808	1674	1808	+	hypothetical protein	
648	3397	NEOS13_0637	peg	3397_1781_570	1781	570	-	Leucine-rich repeat containing protein	Leucine-rich repeat
649	3399	NEOS13_0638	peg	3399_467_207	467	207	-	hypothetical protein	
650	3399	NEOS13_0639	peg	3399_622_1905	622	1905	+	Proton/glutamate symport protein @ Sodium/glutamate symport protein	
651	3399	NEOS13_0640	peg	3399_2033_3061	2033	3061	+	tRNA-specific 2-thiouridylase MnmA	
652	3399	NEOS13_0641	peg	3399_3806_3126	3806	3126	-	hypothetical protein	
653	3399	NEOS13_0642	peg	3399_4239_4379	4239	4379	+	hypothetical protein	
654	3399	NEOS13_0643	peg	3399_4390_4542	4390	4542	+	hypothetical protein	
655	3400	NEOS13_0644	peg	3400_467_207	467	207	-	hypothetical protein	
656	3400	NEOS13_0645	peg	3400_680_937	680	937	+	KH domain RNA binding protein YlqC	
657	3400	NEOS13_0012rna	rna	3400_1351_1279	1351	1279	-	tRNA-Arg-TCT	RNA
658	3400	NEOS13_0646	peg	3400_2906_1857	2906	1857	-	hypothetical protein	
659	3400	NEOS13_0647	peg	3400_3191_3078	3191	3078	-	hypothetical protein	
660	3400	NEOS13_0648	peg	3400_3383_3997	3383	3997	+	archaeal ATPase, fused to C-terminal DUF234 domain	
661	3400	NEOS13_0649	peg	3400_3967_4782	3967	4782	+	archaeal ATPase, fused to C-terminal DUF234 domain	
662	3400	NEOS13_0650	peg	3400_6431_5088	6431	5088	-	Leucine-rich repeat containing protein	Leucine-rich repeat
663	3400	NEOS13_0651	peg	3400_7146_7024	7146	7024	-	hypothetical protein	
664	3400	NEOS13_0652	peg	3400_7325_7185	7325	7185	-	hypothetical protein	
665	3400	NEOS13_0653	peg	3400_7492_8079	7492	8079	+	2-amino-4-hydroxy-6-hydroxymethyldihydropteridine pyrophosphokinase (EC 2.7.6.3)	
666	3400	NEOS13_0654	peg	3400_8085_8849	8085	8849	+	2-Keto-3-deoxy-D-manno-octulosonate-8-phosphate synthase (EC 2.5.1.55)	
667	3400	NEOS13_0655	peg	3400_9011_9700	9011	9700	+	hypothetical protein	
668	3400	NEOS13_0656	peg	3400_9697_11229	9697	11229	+	hypothetical protein	
669	3400	NEOS13_0657	peg	3400_11344_11973	11344	11973	+	hypothetical protein	ABC_transporter
670	3400	NEOS13_0658	peg	3400_12605_12102	12605	12102	-	Nucleoside diphosphate kinase (EC 2.7.4.6)	
671	3400	NEOS13_0659	peg	3400_13059_12625	13059	12625	-	Nucleoside diphosphate kinase (EC 2.7.4.6)	
672	3400	NEOS13_0660	peg	3400_13157_14401	13157	14401	+	Aminopeptidase S (Leu, Val, Phe, Tyr preference) (EC 3.4.11.24)	
673	3400	NEOS13_0661	peg	3400_15061_15660	15061	15660	+	hypothetical protein	
674	3400	NEOS13_0662	peg	3400_15811_16188	15811	16188	+	hypothetical protein	
675	3400	NEOS13_0663	peg	3400_18024_16501	18024	16501	-	EF hand domain/PKD domain protein	
676	3400	NEOS13_0664	peg	3400_18417_18121	18417	18121	-	Transposase, ISLbp3	Transposase
677	3400	NEOS13_0665	peg	3400_18902_19108	18902	19108	+	hypothetical protein	
678	3400	NEOS13_0666	peg	3400_19421_19546	19421	19546	+	hypothetical protein	
679	3401	NEOS13_0667	peg	3401_55_915	55	915	+	conserved hypothetical protein	
680	3401	NEOS13_0668	peg	3401_1469_1335	1469	1335	-	hypothetical protein	
681	3401	NEOS13_0669	peg	3401_2343_1666	2343	1666	-	Cytidylate kinase (EC 2.7.4.14)	
682	3401	NEOS13_0670	peg	3401_3222_2368	3222	2368	-	Phosphatidate cytidylyltransferase (EC 2.7.7.41)	
683	3401	NEOS13_0671	peg	3401_3977_3219	3977	3219	-	Undecaprenyl pyrophosphate synthetase (EC 2.5.1.31)	
684	3401	NEOS13_0672	peg	3401_5306_3987	5306	3987	-	Adenylosuccinate synthetase (EC 6.3.4.4)	
685	3401	NEOS13_0673	peg	3401_7138_5330	7138	5330	-	Translation elongation factor LepA	
686	3401	NEOS13_0674	peg	3401_7966_7331	7966	7331	-	hypothetical protein	
687	3401	NEOS13_0013rna	rna	3401_8083_8000	8083	8000	-	tRNA-Leu-CAG	RNA
688	3401	NEOS13_0675	peg	3401_8749_8138	8749	8138	-	hypothetical protein	
689	3401	NEOS13_0676	peg	3401_8917_10068	8917	10068	+	Sulfite reductase [NADPH] flavoprotein alpha-component (EC 1.8.1.2)	
690	3401	NEOS13_0677	peg	3401_10655_10158	10655	10158	-	2-C-methyl-D-erythritol 2,4-cyclodiphosphate synthase (EC 4.6.1.12)	
691	3401	NEOS13_0678	peg	3401_11006_12022	11006	12022	+	hypothetical protein	
692	3401	NEOS13_0679	peg	3401_12078_13625	12078	13625	+	UDP-N-acetylglucosamine 1-carboxyvinyltransferase (EC 2.5.1.7)	
693	3401	NEOS13_0680	peg	3401_14409_14996	14409	14996	+	hypothetical protein	
694	3401	NEOS13_0681	peg	3401_15226_16725	15226	16725	+	hypothetical protein	
695	3401	NEOS13_0682	peg	3401_17079_16912	17079	16912	-	hypothetical protein	
696	3401	NEOS13_0683	peg	3401_17210_17404	17210	17404	+	Mobile element protein	

697	3401	NEOS13_0684	peg	3401_17661_18530	17661	18530	+	hypothetical protein	
698	3401	NEOS13_0685	peg	3401_18858_18556	18858	18556	-	hypothetical protein	
699	3401	NEOS13_0686	peg	3401_19051_18938	19051	18938	-	hypothetical protein	
700	3403	NEOS13_0687	peg	3403_775_662	775	662	-	hypothetical protein	
701	3403	NEOS13_0688	peg	3403_2128_1271	2128	1271	-	AMP nucleosidase (EC 3.2.2.4)	
702	3403	NEOS13_0689	peg	3403_3773_2544	3773	2544	-	hypothetical protein	
703	3403	NEOS13_0690	peg	3403_4015_5619	4015	5619	+	Peptide chain release factor 3	
704	3403	NEOS13_0691	peg	3403_5701_5579	5701	5579	-	hypothetical protein	
705	3403	NEOS13_0692	peg	3403_6329_5817	6329	5817	-	hypothetical protein	
706	3403	NEOS13_0693	peg	3403_6402_7451	6402	7451	+	A/G-specific adenine glycosylase (EC 3.2.2.-)	
707	3403	NEOS13_0694	peg	3403_7681_9597	7681	9597	+	Leucine-rich repeat containing protein	Leucine-rich repeat
708	3403	NEOS13_0695	peg	3403_9607_9726	9607	9726	+	Leucine-rich repeat containing protein	Leucine-rich repeat
709	3404	NEOS13_0696	peg	3404_835_659	835	659	-	Leucine-rich repeat containing protein	Leucine-rich repeat
710	3404	NEOS13_0697	peg	3404_2783_1182	2783	1182	-	Leucine-rich repeat containing protein	Leucine-rich repeat
711	3404	NEOS13_0698	peg	3404_3050_2826	3050	2826	-	hypothetical protein	
712	3404	NEOS13_0699	peg	3404_3239_3105	3239	3105	-	hypothetical protein	
713	3404	NEOS13_0700	peg	3404_3582_3698	3582	3698	+	Mobile element protein	
714	3405	NEOS13_0701	peg	3405_440_216	440	216	-	hypothetical protein	
715	3405	NEOS13_0702	peg	3405_629_495	629	495	-	hypothetical protein	
716	3405	NEOS13_0703	peg	3405_972_1088	972	1088	+	Mobile element protein	
717	3405	NEOS13_0704	peg	3405_1339_1488	1339	1488	+	hypothetical protein	
718	3405	NEOS13_0705	peg	3405_1765_1577	1765	1577	-	hypothetical protein	
719	3405	NEOS13_0706	peg	3405_2363_2740	2363	2740	+	probable histone H1-like protein	
720	3405	NEOS13_0707	peg	3405_2970_4427	2970	4427	+	RNA methyltransferase, TrmA family	
721	3405	NEOS13_0708	peg	3405_4435_5535	4435	5535	+	tRNA-guanine transglycosylase (EC 2.4.2.29)	
722	3405	NEOS13_0709	peg	3405_5547_5969	5547	5969	+	Preprotein translocase subunit YajC (TC 3.A.5.1.1)	
723	3405	NEOS13_0710	peg	3405_5981_6637	5981	6637	+	Protein-L-isoaspartate O-methyltransferase (EC 2.1.1.77)	
724	3405	NEOS13_0711	peg	3405_6920_7567	6920	7567	+	hypothetical protein	
725	3405	NEOS13_0712	peg	3405_8079_10043	8079	10043	+	Leucine-rich repeat containing protein	Leucine-rich repeat
726	3405	NEOS13_0014ma	rna	3405_10911_10984	10911	10984	+	tRNA-Arg-ACG	RNA
727	3405	NEOS13_0713	peg	3405_10985_11620	10985	11620	+	Hydroxyacylglutathione hydrolase (EC 3.1.2.6)	
728	3405	NEOS13_0714	peg	3405_11648_12235	11648	12235	+	Alkyl hydroperoxide reductase subunit C-like protein	
729	3407	NEOS13_0715	peg	3407_554_2293	554	2293	+	Leucine-rich repeat containing protein	Leucine-rich repeat
730	3407	NEOS13_0716	peg	3407_2488_2372	2488	2372	-	hypothetical protein	
731	3407	NEOS13_0717	peg	3407_2600_3487	2600	3487	+	Leucine-rich repeat containing protein	Leucine-rich repeat
732	3407	NEOS13_0718	peg	3407_3710_3907	3710	3907	+	Leucine-rich repeat containing protein	Leucine-rich repeat
733	3407	NEOS13_0719	peg	3407_3996_4256	3996	4256	+	hypothetical protein	
734	3407	NEOS13_0720	peg	3407_4271_4441	4271	4441	+	hypothetical protein	
735	3407	NEOS13_0721	peg	3407_5518_4799	5518	4799	-	Competence protein F homolog, phosphoribosyltransferase domain; protein YhgH required for utilization of DNA as sole source of carbon and energy	Genetic_competence
736	3407	NEOS13_0722	peg	3407_6468_5518	6468	5518	-	unknown protein	
737	3407	NEOS13_0723	peg	3407_7215_8750	7215	8750	+	Na(+)-translocating NADH-quinone reductase subunit B (EC 1.6.5.-)	Oxidative_Phosphorylation
738	3407	NEOS13_0724	peg	3407_8731_9663	8731	9663	+	Na(+)-translocating NADH-quinone reductase subunit C (EC 1.6.5.-)	Oxidative_Phosphorylation
739	3407	NEOS13_0725	peg	3407_9802_10440	9802	10440	+	Na(+)-translocating NADH-quinone reductase subunit D (EC 1.6.5.-)	Oxidative_Phosphorylation
740	3407	NEOS13_0726	peg	3407_10447_11184	10447	11184	+	Na(+)-translocating NADH-quinone reductase subunit E (EC 1.6.5.-)	Oxidative_Phosphorylation
741	3407	NEOS13_0727	peg	3407_12119_13741	12119	13741	+	hypothetical protein	
742	3407	NEOS13_0728	peg	3407_13810_14133	13810	14133	+	hypothetical protein	
743	3407	NEOS13_0729	peg	3407_14652_14248	14652	14248	-	Glycine cleavage system H protein	
744	3407	NEOS13_0730	peg	3407_15392_14763	15392	14763	-	unknown protein	
745	3407	NEOS13_0731	peg	3407_17903_15510	17903	15510	-	hypothetical protein	
746	3407	NEOS13_0732	peg	3407_18975_18262	18975	18262	-	Lipoate-protein ligase A	
747	3407	NEOS13_0733	peg	3407_19731_19033	19731	19033	-	hypothetical protein	
748	3407	NEOS13_0734	peg	3407_19921_20631	19921	20631	+	Ribonuclease III (EC 3.1.26.3)	
749	3407	NEOS13_0735	peg	3407_20639_22015	20639	22015	+	DNA repair protein RadA	
750	3407	NEOS13_0736	peg	3407_22086_23399	22086	23399	+	Porphobilinogen deaminase (EC 2.5.1.61)	
751	3407	NEOS13_0737	peg	3407_23653_25023	23653	25023	+	Leucine-rich repeat containing protein	Leucine-rich repeat
752	3407	NEOS13_0738	peg	3407_25260_26864	25260	26864	+	Leucine-rich repeat containing protein	Leucine-rich repeat
753	3407	NEOS13_0739	peg	3407_27333_27214	27333	27214	-	hypothetical protein	
754	3408	NEOS13_0740	peg	3408_508_206	508	206	-	Leucine-rich repeat containing protein	Leucine-rich repeat
755	3408	NEOS13_0741	peg	3408_1564_563	1564	563	-	Leucine-rich repeat containing protein	Leucine-rich repeat
756	3410	NEOS13_0742	peg	3410_118_348	118	348	+	hypothetical protein	
757	3410	NEOS13_0743	peg	3410_348_680	348	680	+	hypothetical protein	
758	3410	NEOS13_0744	peg	3410_2889_1174	2889	1174	-	hypothetical protein	
759	3410	NEOS13_0745	peg	3410_3290_3820	3290	3820	+	Putative preQ0 transporter	
760	3410	NEOS13_0746	peg	3410_5228_4086	5228	4086	-	Branched-chain amino acid transport system carrier protein	
761	3410	NEOS13_0747	peg	3410_5660_6199	5660	6199	+	hypothetical protein	
762	3410	NEOS13_0748	peg	3410_7112_6516	7112	6516	-	hypothetical protein-signal peptide prediction	
763	3410	NEOS13_0749	peg	3410_8008_8229	8008	8229	+	hypothetical protein	
764	3410	NEOS13_0750	peg	3410_8440_8189	8440	8189	-	hypothetical protein	
765	3410	NEOS13_0751	peg	3410_8496_9467	8496	9467	+	CBS domain protein	
766	3410	NEOS13_0752	peg	3410_9474_10718	9474	10718	+	Hemolysin	

767	3410	NEOS13_0753	peg	3410_10814_10960	10814	10960	+	hypothetical protein	
768	3410	NEOS13_0754	peg	3410_13519_11000	13519	11000	-	Leucine-rich repeat containing protein	Leucine-rich repeat
769	3410	NEOS13_0755	peg	3410_15578_13647	15578	13647	-	Leucine-rich repeat containing protein	Leucine-rich repeat
770	3410	NEOS13_0756	peg	3410_18450_15997	18450	15997	-	Leucine-rich repeat containing protein	Leucine-rich repeat
771	3410	NEOS13_0757	peg	3410_18750_18893	18750	18893	+	hypothetical protein	
772	3410	NEOS13_0758	peg	3410_21444_18868	21444	18868	-	hypothetical protein	
773	3410	NEOS13_0759	peg	3410_21555_21683	21555	21683	+	hypothetical protein	
774	3410	NEOS13_0760	peg	3410_23071_22001	23071	22001	-	Cysteine desulfurase (EC 2.8.1.7)	
775	3410	NEOS13_0761	peg	3410_23894_23127	23894	23127	-	Protein serine/threonine phosphatase PrpC, regulation of stationary phase	
776	3410	NEOS13_0762	peg	3410_23980_24096	23980	24096	+	hypothetical protein	
777	3410	NEOS13_0763	peg	3410_25351_24083	25351	24083	-	proteinase inhibitor I4, serpin	
778	3410	NEOS13_0764	peg	3410_26241_25351	26241	25351	-	hypothetical protein	
779	3410	NEOS13_0765	peg	3410_26855_26319	26855	26319	-	hypothetical protein	
780	3410	NEOS13_0766	peg	3410_28006_27341	28006	27341	-	Nucleoside triphosphate pyrophosphohydrolase MazG (EC 3.6.1.8)	
781	3410	NEOS13_0767	peg	3410_28317_28781	28317	28781	+	hypothetical protein	
782	3410	NEOS13_0768	peg	3410_30885_29269	30885	29269	-	hypothetical protein	
783	3410	NEOS13_0769	peg	3410_32045_30978	32045	30978	-	Lipopolysaccharide heptosyltransferase I (EC 2.4.1.-)	
784	3410	NEOS13_0770	peg	3410_32917_32042	32917	32042	-	hypothetical protein	
785	3410	NEOS13_0771	peg	3410_34037_33009	34037	33009	-	Phenylalanyl-tRNA synthetase alpha chain (EC 6.1.1.20)	
786	3410	NEOS13_0772	peg	3410_34478_34122	34478	34122	-	LSU ribosomal protein L20p	
787	3410	NEOS13_0773	peg	3410_35369_34803	35369	34803	-	Translation initiation factor 3	
788	3410	NEOS13_0774	peg	3410_37162_35462	37162	35462	-	Threonyl-tRNA synthetase (EC 6.1.1.3)	
789	3410	NEOS13_0015m a	rna	3410_37249_37176	37249	37176	-	tRNA-Val-GAC	RNA
790	3410	NEOS13_0775	peg	3410_39176_37350	39176	37350	-	Excinuclease ABC subunit C	ABC_transporter
791	3410	NEOS13_0776	peg	3410_39390_39232	39390	39232	-	hypothetical protein	
792	3410	NEOS13_0777	peg	3410_39808_39602	39808	39602	-	hypothetical protein	
793	3410	NEOS13_0778	peg	3410_40991_39924	40991	39924	-	Ribosomal RNA large subunit methyltransferase N (EC 2.1.1.-)	
794	3410	NEOS13_0779	peg	3410_41438_41301	41438	41301	-	hypothetical protein	
795	3410	NEOS13_0780	peg	3410_41468_44524	41468	44524	+	Glycyl-tRNA synthetase alpha chain (EC 6.1.1.14)	
796	3410	NEOS13_0781	peg	3410_45696_44845	45696	44845	-	Heme O synthase, protoheme IX farnesyltransferase (EC 2.5.1.-) COX10-CtaB	Oxidative_Phosphorylation
797	3410	NEOS13_0782	peg	3410_46111_45746	46111	45746	-	Cytochrome O ubiquinol oxidase subunit IV (EC 1.10.3.-)	Oxidative_Phosphorylation
798	3410	NEOS13_0783	peg	3410_46721_46113	46721	46113	-	Cytochrome O ubiquinol oxidase subunit III (EC 1.10.3.-)	Oxidative_Phosphorylation
799	3410	NEOS13_0784	peg	3410_48412_46724	48412	46724	-	Cytochrome O ubiquinol oxidase subunit I (EC 1.10.3.-)	Oxidative_Phosphorylation
800	3413	NEOS13_0785	peg	3413_1679_615	1679	615	-	Leucine-rich repeat containing protein	Leucine-rich repeat
801	3413	NEOS13_0786	peg	3413_1674_1805	1674	1805	+	hypothetical protein	
802	3413	NEOS13_0787	peg	3413_3368_2433	3368	2433	-	YrdC/Sua5 family protein, required for threonylcarbamoyladenosine (t(6)A) formation in tRNA	
803	3413	NEOS13_0788	peg	3413_4591_3353	4591	3353	-	Cysteine desulfurase (EC 2.8.1.7), SufS subfamily	
804	3413	NEOS13_0789	peg	3413_5918_4572	5918	4572	-	Iron-sulfur cluster assembly protein SufD	
805	3413	NEOS13_0790	peg	3413_6700_5915	6700	5915	-	Iron-sulfur cluster assembly ATPase protein SufC	
806	3413	NEOS13_0791	peg	3413_8146_6707	8146	6707	-	Iron-sulfur cluster assembly protein SufB	
807	3413	NEOS13_0792	peg	3413_11203_8639	11203	8639	-	hypothetical protein	
808	3413	NEOS13_0793	peg	3413_12332_11229	12332	11229	-	hypothetical protein	
809	3413	NEOS13_0794	peg	3413_12649_12801	12649	12801	+	hypothetical protein	
810	3413	NEOS13_0795	peg	3413_12798_12962	12798	12962	+	hypothetical protein	
811	3413	NEOS13_0796	peg	3413_13078_13338	13078	13338	+	hypothetical protein	
812	3414	NEOS13_0797	peg	3414_22_225	22	225	+	hypothetical protein	
813	3414	NEOS13_0798	peg	3414_719_925	719	925	+	hypothetical protein	
814	3414	NEOS13_0799	peg	3414_1071_1445	1071	1445	+	hypothetical protein	
815	3416	NEOS13_0800	peg	3416_36_1076	36	1076	+	Leucine-rich repeat containing protein	Leucine-rich repeat
816	3416	NEOS13_0801	peg	3416_1096_1239	1096	1239	+	hypothetical protein	
817	3417	NEOS13_0802	peg	3417_2308_968	2308	968	-	Leucine-rich repeat containing protein	Leucine-rich repeat
818	3417	NEOS13_0803	peg	3417_2533_2417	2533	2417	-	hypothetical protein	
819	3417	NEOS13_0804	peg	3417_2696_2565	2696	2565	-	hypothetical protein	
820	3417	NEOS13_0805	peg	3417_3300_3184	3300	3184	-	hypothetical protein	
821	3417	NEOS13_0806	peg	3417_3442_3597	3442	3597	+	Mobile element protein	
822	3417	NEOS13_0807	peg	3417_3814_3674	3814	3674	-	hypothetical protein	
823	3417	NEOS13_0808	peg	3417_6274_4478	6274	4478	-	hypothetical protein	
824	3417	NEOS13_0809	peg	3417_6645_6806	6645	6806	+	hypothetical protein	
825	3417	NEOS13_0810	peg	3417_7058_10480	7058	10480	+	Exodeoxyribonuclease V gamma chain (EC 3.1.11.5)	
826	3417	NEOS13_0811	peg	3417_10640_11461	10640	11461	+	hypothetical protein	
827	3417	NEOS13_0812	peg	3417_12600_16106	12600	16106	+	Exodeoxyribonuclease V beta chain (EC 3.1.11.5)	
828	3417	NEOS13_0813	peg	3417_16163_17911	16163	17911	+	Exodeoxyribonuclease V alpha chain (EC 3.1.11.5)	
829	3417	NEOS13_0814	peg	3417_18745_18356	18745	18356	-	hypothetical protein	
830	3417	NEOS13_0815	peg	3417_19992_18823	19992	18823	-	hypothetical protein	
831	3417	NEOS13_0816	peg	3417_21339_20854	21339	20854	-	hypothetical protein	
832	3417	NEOS13_0817	peg	3417_21497_21634	21497	21634	+	hypothetical protein	

833	3417	NEOS13_0818	peg	3417_21651_23816	21651	23816	+	Transcription elongation factor GreA	
834	3417	NEOS13_0819	peg	3417_23817_24425	23817	24425	+	Nucleoside 5-triphosphatase RdgB (dHAPTP, dITP, XTP-specific) (EC 3.6.1.15)	
835	3417	NEOS13_0820	peg	3417_24434_25129	24434	25129	+	hypothetical protein	
836	3417	NEOS13_0821	peg	3417_25339_26322	25339	26322	+	hypothetical protein	
837	3417	NEOS13_0016rna	rna	3417_26467_26539	26467	26539	+	tRNA-Ala-GGC	RNA
838	3417	NEOS13_0822	peg	3417_26619_26768	26619	26768	+	hypothetical protein	
839	3417	NEOS13_0823	peg	3417_27243_27010	27243	27010	-	hypothetical protein	
840	3417	NEOS13_0824	peg	3417_28112_27384	28112	27384	-	hypothetical protein	
841	3417	NEOS13_0825	peg	3417_28906_28175	28906	28175	-	hypothetical protein	
842	3417	NEOS13_0826	peg	3417_28906_29022	28906	29022	+	hypothetical protein	
843	3417	NEOS13_0827	peg	3417_30266_29307	30266	29307	-	hypothetical protein	
844	3417	NEOS13_0828	peg	3417_32127_30685	32127	30685	-	hypothetical protein	Outer_membrane_protein
845	3417	NEOS13_0829	peg	3417_35956_33017	35956	33017	-	hypothetical protein	
846	3417	NEOS13_0830	peg	3417_36975_36154	36975	36154	-	hypothetical protein	
847	3417	NEOS13_0831	peg	3417_37298_39235	37298	39235	+	Threonyl-tRNA synthetase (EC 6.1.1.3)	
848	3417	NEOS13_0832	peg	3417_39223_39975	39223	39975	+	Septum site-determining protein MinD	
849	3417	NEOS13_0833	peg	3417_40112_40858	40112	40858	+	Virulence plasmid protein pGP6-D	
850	3417	NEOS13_0834	peg	3417_41447_41331	41447	41331	-	hypothetical protein	
851	3417	NEOS13_0835	peg	3417_41495_41641	41495	41641	+	hypothetical protein	
852	3417	NEOS13_0836	peg	3417_41770_42477	41770	42477	+	hypothetical protein	
853	3418	NEOS13_0837	peg	3418_63_248	63	248	+	hypothetical protein	
854	3418	NEOS13_0838	peg	3418_1318_638	1318	638	-	Leucine-rich repeat containing protein	Leucine-rich repeat
855	3419	NEOS13_0839	peg	3419_314_54	314	54	-	hypothetical protein	
856	3419	NEOS13_0840	peg	3419_328_480	328	480	+	hypothetical protein	
857	3419	NEOS13_0841	peg	3419_500_1387	500	1387	+	Protein YicC	
858	3419	NEOS13_0842	peg	3419_1377_1958	1377	1958	+	Guanylate kinase (EC 2.7.4.8)	
859	3419	NEOS13_0843	peg	3419_1983_2306	1983	2306	+	hypothetical protein	
860	3419	NEOS13_0844	peg	3419_2328_2939	2328	2939	+	hypothetical protein	
861	3419	NEOS13_0845	peg	3419_2993_5044	2993	5044	+	Methionyl-tRNA synthetase (EC 6.1.1.10)	
862	3419	NEOS13_0846	peg	3419_5883_5608	5883	5608	-	LSU ribosomal protein L28p	
863	3419	NEOS13_0847	peg	3419_6434_5997	6434	5997	-	hypothetical protein	
864	3419	NEOS13_0848	peg	3419_6598_8013	6598	8013	+	Soluble pyridine nucleotide transhydrogenase (EC 1.6.1.1)	
865	3419	NEOS13_0849	peg	3419_8000_8521	8000	8521	+	hypothetical protein	
866	3419	NEOS13_0850	peg	3419_11182_8576	11182	8576	-	ClpB protein	
867	3419	NEOS13_0851	peg	3419_11420_11286	11420	11286	-	hypothetical protein	
868	3419	NEOS13_0852	peg	3419_12311_11688	12311	11688	-	hypothetical protein	
869	3419	NEOS13_0853	peg	3419_12473_12610	12473	12610	+	hypothetical protein	
870	3419	NEOS13_0854	peg	3419_12846_14762	12846	14762	+	1-hydroxy-2-methyl-2-(E)-butenyl 4-diphosphate synthase (EC 1.17.7.1)	
871	3419	NEOS13_0855	peg	3419_14889_15002	14889	15002	+	hypothetical protein	
872	3419	NEOS13_0856	peg	3419_15209_16147	15209	16147	+	ankyrin repeat protein	Ankyrin
873	3419	NEOS13_0857	peg	3419_16987_16388	16987	16388	-	Cob(I)alamin adenosyltransferase PduO (EC 2.5.1.17)	
874	3419	NEOS13_0858	peg	3419_17838_16990	17838	16990	-	hypothetical protein	
875	3419	NEOS13_0859	peg	3419_17963_18091	17963	18091	+	hypothetical protein	
876	3419	NEOS13_0860	peg	3419_19794_18232	19794	18232	-	hypothetical protein	
877	3419	NEOS13_0861	peg	3419_21396_19795	21396	19795	-	4-alpha-glucanotransferase (amylomaltase) (EC 2.4.1.25)	T3SA1
878	3419	NEOS13_0862	peg	3419_21518_21634	21518	21634	+	hypothetical protein	T3SA1
879	3419	NEOS13_0863	peg	3419_22060_21587	22060	21587	-	hypothetical protein	T3SA1
880	3419	NEOS13_0864	peg	3419_22388_22068	22388	22068	-	hypothetical protein	T3SA1
881	3419	NEOS13_0865	peg	3419_23318_22410	23318	22410	-	hypothetical protein	T3SA1
882	3419	NEOS13_0866	peg	3419_25536_23356	25536	23356	-	Low Calcium Response D (Type III secretion inner membrane protein SctV)	T3SA1
883	3419	NEOS13_0867	peg	3419_26613_25540	26613	25540	-	Type III secretion inner membrane protein (YscU,SpaS,EscU,HrcU,SsaU, homologous to flagellar export components)	T3SA1
884	3419	NEOS13_0868	peg	3419_27179_27607	27179	27607	+	transposase, IS4 family protein	T3SA1, Transposase
885	3420	NEOS13_0869	peg	3420_22_189	22	189	+	hypothetical protein	
886	3420	NEOS13_0870	peg	3420_448_1848	448	1848	+	hypothetical protein	
887	3420	NEOS13_0871	peg	3420_2076_2783	2076	2783	+	hypothetical protein	
888	3420	NEOS13_0872	peg	3420_2799_3467	2799	3467	+	hypothetical protein	
889	3420	NEOS13_0873	peg	3420_3580_3711	3580	3711	+	hypothetical protein	
890	3420	NEOS13_0874	peg	3420_3713_3844	3713	3844	+	hypothetical protein	
891	3420	NEOS13_0875	peg	3420_3914_4087	3914	4087	+	hypothetical protein	
892	3420	NEOS13_0876	peg	3420_6442_4466	6442	4466	-	Chaperone protein DnaK	
893	3420	NEOS13_0877	peg	3420_7202_6582	7202	6582	-	Heat shock protein GrpE	
894	3420	NEOS13_0878	peg	3420_8410_7217	8410	7217	-	Heat-inducible transcription repressor HrcA	
895	3420	NEOS13_0879	peg	3420_9401_8628	9401	8628	-	hypothetical protein	
896	3420	NEOS13_0880	peg	3420_10928_9561	10928	9561	-	hypothetical protein	
897	3420	NEOS13_0881	peg	3420_12952_11216	12952	11216	-	hypothetical protein	
898	3420	NEOS13_0882	peg	3420_15326_13338	15326	13338	-	Lead, cadmium, zinc and mercury transporting ATPase (EC 3.6.3.3) (EC 3.6.3.5); Copper-translocating P-type ATPase (EC 3.6.3.4)	
899	3420	NEOS13_0883	peg	3420_16090_15332	16090	15332	-	hypothetical protein	
900	3420	NEOS13_0884	peg	3420_16302_17219	16302	17219	+	hypothetical protein	
901	3420	NEOS13_0885	peg	3420_19404_17341	19404	17341	-	hypothetical protein	
902	3421	NEOS13_0886	peg	3421_35_982	35	982	+	Leucine-rich repeat containing protein	Leucine-rich repeat
903	3421	NEOS13_0887	peg	3421_1002_1145	1002	1145	+	hypothetical protein	

904	3422	NEOS13_0888	peg	3422_1894_1037	1894	1037	-	hypothetical protein	
905	3423	NEOS13_0889	peg	3423_1112_348	1112	348	-	Leucine-rich repeat containing protein	Leucine-rich repeat
906	3424	NEOS13_0890	peg	3424_31_204	31	204	+	hypothetical protein	
907	3424	NEOS13_0891	peg	3424_468_1886	468	1886	+	Leucine-rich repeat containing protein	Leucine-rich repeat
908	3424	NEOS13_0892	peg	3424_2868_2080	2868	2080	-	nifU protein, putative	
909	3424	NEOS13_0893	peg	3424_4045_2879	4045	2879	-	Cysteine desulfurase (EC 2.8.1.7)	
910	3424	NEOS13_0894	peg	3424_4703_4047	4703	4047	-	Phosphoglycerate mutase (EC 5.4.2.1)	Glycolysis_Glucogenesis
911	3424	NEOS13_0895	peg	3424_4972_5649	4972	5649	+	putative ribosomal large chain pseudouridine synthase B	
912	3424	NEOS13_0896	peg	3424_5649_6815	5649	6815	+	hypothetical protein	
913	3424	NEOS13_0897	peg	3424_6828_8219	6828	8219	+	hypothetical protein	
914	3424	NEOS13_0898	peg	3424_9268_8222	9268	8222	-	hypothetical protein	
915	3424	NEOS13_0899	peg	3424_10125_9436	10125	9436	-	hypothetical protein	
916	3424	NEOS13_0900	peg	3424_10908_10141	10908	10141	-	hypothetical protein	
917	3425	NEOS13_0901	peg	3425_22_1353	22	1353	+	Leucine-rich repeat containing protein	Leucine-rich repeat
918	3425	NEOS13_0902	peg	3425_1723_1854	1723	1854	+	hypothetical protein	
919	3425	NEOS13_0903	peg	3425_2604_2269	2604	2269	-	hypothetical protein	
920	3425	NEOS13_0904	peg	3425_3396_2674	3396	2674	-	hypothetical protein	
921	3426	NEOS13_0905	peg	3426_15_1049	15	1049	+	Leucine-rich repeat containing protein	Leucine-rich repeat
922	3426	NEOS13_0906	peg	3426_1543_1361	1543	1361	-	hypothetical protein	
923	3426	NEOS13_0907	peg	3426_1538_2740	1538	2740	+	Leucine-rich repeat containing protein	Leucine-rich repeat
924	3426	NEOS13_0908	peg	3426_5011_4262	5011	4262	-	tRNA pseudouridine synthase A (EC 4.2.1.70)	
925	3426	NEOS13_0909	peg	3426_5698_5015	5698	5015	-	2-C-methyl-D-erythritol 4-phosphate cytidylyltransferase (EC 2.7.7.60)	
926	3426	NEOS13_0910	peg	3426_6683_5703	6683	5703	-	hypothetical protein	
927	3426	NEOS13_0911	peg	3426_7057_6785	7057	6785	-	SWIB (YM74) complex protein	
928	3426	NEOS13_0912	peg	3426_7291_9501	7291	9501	+	hypothetical protein	
929	3426	NEOS13_0913	peg	3426_9918_9532	9918	9532	-	hypothetical protein	
930	3426	NEOS13_0914	peg	3426_9944_10096	9944	10096	+	hypothetical protein	
931	3426	NEOS13_0915	peg	3426_11596_10289	11596	10289	-	hypothetical protein	
932	3426	NEOS13_0916	peg	3426_12172_13152	12172	13152	+	Peptide chain release factor 2; programmed frameshift-containing	
933	3426	NEOS13_0917	peg	3426_13149_13661	13149	13661	+	Amino Group Acetyl Transferase	
934	3426	NEOS13_0918	peg	3426_13708_14385	13708	14385	+	hypothetical protein	
935	3426	NEOS13_0919	peg	3426_14431_15156	14431	15156	+	FIG000859: hypothetical protein YebC	
936	3426	NEOS13_0920	peg	3426_15782_16759	15782	16759	+	hypothetical protein	
937	3426	NEOS13_0921	peg	3426_17086_18609	17086	18609	+	Prolyl-tRNA synthetase (EC 6.1.1.15)	
938	3426	NEOS13_0922	peg	3426_19148_18831	19148	18831	-	hypothetical protein	
939	3426	NEOS13_0923	peg	3426_19359_19709	19359	19709	+	hypothetical protein	
940	3426	NEOS13_0924	peg	3426_20606_19683	20606	19683	-	Integral membrane protein TerC	
941	3426	NEOS13_0925	peg	3426_20649_20771	20649	20771	+	hypothetical protein	
942	3426	NEOS13_0926	peg	3426_21068_24619	21068	24619	+	Chromosome partition protein smc	
943	3427	NEOS13_0927	peg	3427_18_1205	18	1205	+	Leucine-rich repeat containing protein	Leucine-rich repeat
944	3428	NEOS13_0928	peg	3428_148_291	148	291	+	hypothetical protein	
945	3428	NEOS13_0929	peg	3428_545_333	545	333	-	hypothetical protein	
946	3428	NEOS13_0930	peg	3428_1645_3864	1645	3864	+	hypothetical protein	
947	3428	NEOS13_0931	peg	3428_4753_5727	4753	5727	+	hypothetical protein	
948	3428	NEOS13_0932	peg	3428_8314_5918	8314	5918	-	Xylulose-5-phosphate phosphoketolase (EC 4.1.2.9); Fructose-6-phosphate phosphoketolase (EC 4.1.2.22)	
949	3428	NEOS13_0933	peg	3428_9914_8889	9914	8889	-	Porphobilinogen synthase (EC 4.2.1.24)	
950	3428	NEOS13_0934	peg	3428_10450_10301	10450	10301	-	hypothetical protein	
951	3428	NEOS13_0935	peg	3428_10683_11282	10683	11282	+	Putative two-domain glycosyltransferase	
952	3428	NEOS13_0936	peg	3428_12776_11568	12776	11568	-	Leucine-rich repeat containing protein	Leucine-rich repeat
953	3428	NEOS13_0937	peg	3428_13166_13008	13166	13008	-	hypothetical protein	
954	3428	NEOS13_0938	peg	3428_13344_14963	13344	14963	+	Leucine-rich repeat containing protein	Leucine-rich repeat
955	3428	NEOS13_0939	peg	3428_15259_15116	15259	15116	-	hypothetical protein	
956	3429	NEOS13_0940	peg	3429_139_279	139	279	+	hypothetical protein	
957	3429	NEOS13_0941	peg	3429_1195_503	1195	503	-	hypothetical protein	
958	3429	NEOS13_0942	peg	3429_2503_1232	2503	1232	-	hypothetical protein	
959	3429	NEOS13_0943	peg	3429_3731_2658	3731	2658	-	hypothetical protein	
960	3429	NEOS13_0944	peg	3429_3964_3827	3964	3827	-	hypothetical protein	
961	3429	NEOS13_0945	peg	3429_4086_4286	4086	4286	+	hypothetical protein	
962	3429	NEOS13_0946	peg	3429_4570_4337	4570	4337	-	hypothetical protein	
963	3429	NEOS13_0947	peg	3429_4826_5080	4826	5080	+	Mobile element protein	
964	3429	NEOS13_0948	peg	3429_5184_5038	5184	5038	-	hypothetical protein	
965	3429	NEOS13_0949	peg	3429_5347_5895	5347	5895	+	hypothetical protein	
966	3429	NEOS13_0950	peg	3429_6041_6385	6041	6385	+	transposase family protein	Transposase
967	3429	NEOS13_0951	peg	3429_6460_6744	6460	6744	+	Mobile element protein	
968	3429	NEOS13_0952	peg	3429_6800_6991	6800	6991	+	Integrase, catalytic region	
969	3429	NEOS13_0953	peg	3429_6997_7170	6997	7170	+	hypothetical protein	
970	3430	NEOS13_0954	peg	3430_130_1092	130	1092	+	hypothetical protein	
971	3430	NEOS13_0955	peg	3430_1651_1812	1651	1812	+	hypothetical protein	
972	3431	NEOS13_0956	peg	3431_973_815	973	815	-	hypothetical protein	
973	3431	NEOS13_0957	peg	3431_1620_1165	1620	1165	-	Holo-[acyl-carrier protein] synthase (EC 2.7.8.7)	
974	3431	NEOS13_0958	peg	3431_1678_1535	1678	1535	-	hypothetical protein	
975	3431	NEOS13_0959	peg	3431_2065_2565	2065	2565	+	hypothetical protein	
976	3431	NEOS13_0960	peg	3431_3068_2646	3068	2646	-	LSU m3Psi1915 methyltransferase RlmH	
977	3431	NEOS13_0961	peg	3431_3571_3116	3571	3116	-	tmRNA-binding protein SmpB	
978	3431	NEOS13_0962	peg	3431_3794_4930	3794	4930	+	DNA polymerase III beta subunit (EC 2.7.7.7)	
979	3431	NEOS13_0963	peg	3431_4952_5986	4952	5986	+	DNA recombination and repair protein RecF	

980	3431	NEOS13_0964	peg	3431_7346_6015	7346	6015	-	hypothetical protein	
981	3431	NEOS13_0965	peg	3431_7496_7618	7496	7618	+	hypothetical protein	
982	3431	NEOS13_0966	peg	3431_7644_7871	7644	7871	+	hypothetical protein	
983	3431	NEOS13_0967	peg	3431_8913_7861	8913	7861	-	Thiamin biosynthesis lipoprotein ApbE	
984	3431	NEOS13_0968	peg	3431_9825_8935	9825	8935	-	Methylenetetrahydrofolate dehydrogenase (NADP+) (EC 1.5.1.5) / Methenyltetrahydrofolate cyclohydrolase (EC 3.5.4.9)	
985	3431	NEOS13_0969	peg	3431_9988_9869	9988	9869	-	hypothetical protein	
986	3431	NEOS13_0970	peg	3431_10640_11188	10640	11188	+	hypothetical protein	
987	3431	NEOS13_0971	peg	3431_12996_11713	12996	11713	-	hypothetical protein	
988	3431	NEOS13_0972	peg	3431_13336_12959	13336	12959	-	Ferredoxin, 2Fe-2S	
989	3431	NEOS13_0973	peg	3431_13841_13338	13841	13338	-	hypothetical protein	
990	3431	NEOS13_0974	peg	3431_14714_14007	14714	14007	-	Undecaprenyl-phosphate galactosephosphotransferase (EC 2.7.8.6)	
991	3431	NEOS13_0975	peg	3431_16258_15383	16258	15383	-	Octaprenyl-diphosphate synthase (EC 2.5.1.-) / Dimethylallyltransferase (EC 2.5.1.1) / Geranyltranstransferase (farnesyldiphosphate synthase) (EC 2.5.1.10) / Geranylgeranyl pyrophosphate synthetase (EC 2.5.1.29)	
992	3431	NEOS13_0976	peg	3431_17667_16309	17667	16309	-	UDP-glucose dehydrogenase (EC 1.1.1.22)	
993	3431	NEOS13_0977	peg	3431_19936_17750	19936	17750	-	hypothetical protein	
994	3431	NEOS13_0978	peg	3431_22044_20335	22044	20335	-	hypothetical protein	
995	3431	NEOS13_0979	peg	3431_22618_22034	22618	22034	-	Septum formation protein Maf	
996	3431	NEOS13_0980	peg	3431_22964_22644	22964	22644	-	hypothetical protein	
997	3431	NEOS13_0981	peg	3431_24868_23234	24868	23234	-	hypothetical protein	Outer_membrane_protein
998	3431	NEOS13_0982	peg	3431_25662_25495	25662	25495	-	hypothetical protein	
999	3431	NEOS13_0983	peg	3431_26977_26126	26977	26126	-	NAD kinase (EC 2.7.1.23)	
1000	3431	NEOS13_0984	peg	3431_28888_26987	28888	26987	-	1-deoxy-D-xylulose 5-phosphate synthase (EC 2.2.1.7)	
1001	3431	NEOS13_0985	peg	3431_29159_28908	29159	28908	-	Exodeoxyribonuclease VII small subunit (EC 3.1.11.6)	
1002	3431	NEOS13_0986	peg	3431_30507_29152	30507	29152	-	Exodeoxyribonuclease VII large subunit (EC 3.1.11.6)	
1003	3431	NEOS13_0987	peg	3431_30879_30754	30879	30754	-	hypothetical protein	
1004	3431	NEOS13_0988	peg	3431_30851_31642	30851	31642	+	Triosephosphate isomerase (EC 5.3.1.1)	Glycolysis_Glucogenesis
1005	3431	NEOS13_0989	peg	3431_31639_31935	31639	31935	+	Preprotein translocase subunit SecG (TC 3.A.5.1.1)	
1006	3431	NEOS13_0990	peg	3431_32431_31982	32431	31982	-	hypothetical protein	
1007	3431	NEOS13_0991	peg	3431_33251_32715	33251	32715	-	Peptide deformylase (EC 3.5.1.88)	
1008	3431	NEOS13_0992	peg	3431_33620_33787	33620	33787	+	hypothetical protein	
1009	3431	NEOS13_0993	peg	3431_34233_34096	34233	34096	-	hypothetical protein	
1010	3431	NEOS13_0994	peg	3431_34357_34482	34357	34482	+	hypothetical protein	
1011	3431	NEOS13_0995	peg	3431_34504_34881	34504	34881	+	hypothetical protein	
1012	3431	NEOS13_0996	peg	3431_34944_35222	34944	35222	+	hypothetical protein	
1013	3431	NEOS13_0997	peg	3431_35661_35783	35661	35783	+	hypothetical protein	
1014	3431	NEOS13_0998	peg	3431_36247_39489	36247	39489	+	hypothetical protein	
1015	3431	NEOS13_0999	peg	3431_41026_39677	41026	39677	-	Magnesium and cobalt efflux protein CorC	
1016	3431	NEOS13_1000	peg	3431_41566_41045	41566	41045	-	Metal-dependent hydrolase YbeY, involved in rRNA and/or ribosome maturation and assembly	
1017	3431	NEOS13_1001	peg	3431_41787_41629	41787	41629	-	hypothetical protein	
1018	3431	NEOS13_1002	peg	3431_41976_41803	41976	41803	-	hypothetical protein	
1019	3431	NEOS13_1003	peg	3431_42166_43650	42166	43650	+	hypothetical protein	
1020	3431	NEOS13_1004	peg	3431_43822_43706	43822	43706	-	hypothetical protein	
1021	3431	NEOS13_1005	peg	3431_44077_43949	44077	43949	-	hypothetical protein	
1022	3431	NEOS13_1006	peg	3431_44318_44178	44318	44178	-	hypothetical protein	
1023	3431	NEOS13_1007	peg	3431_44477_44914	44477	44914	+	Mobile element protein	
1024	3432	NEOS13_1008	peg	3432_258_398	258	398	+	hypothetical protein	
1025	3432	NEOS13_1009	peg	3432_499_627	499	627	+	hypothetical protein	
1026	3432	NEOS13_1010	peg	3432_754_870	754	870	+	hypothetical protein	
1027	3432	NEOS13_1011	peg	3432_2410_926	2410	926	-	hypothetical protein	
1028	3432	NEOS13_1012	peg	3432_2600_2773	2600	2773	+	hypothetical protein	
1029	3432	NEOS13_1013	peg	3432_2789_2947	2789	2947	+	hypothetical protein	
1030	3432	NEOS13_1014	peg	3432_3010_3531	3010	3531	+	Metal-dependent hydrolase YbeY, involved in rRNA and/or ribosome maturation and assembly	
1031	3432	NEOS13_1015	peg	3432_3550_4899	3550	4899	+	Magnesium and cobalt efflux protein CorC	
1032	3432	NEOS13_1016	peg	3432_8329_5087	8329	5087	-	hypothetical protein	
1033	3432	NEOS13_1017	peg	3432_8915_8793	8915	8793	-	hypothetical protein	
1034	3432	NEOS13_1018	peg	3432_9632_9354	9632	9354	-	hypothetical protein	
1035	3432	NEOS13_1019	peg	3432_10072_9695	10072	9695	-	hypothetical protein	
1036	3432	NEOS13_1020	peg	3432_10219_10094	10219	10094	-	hypothetical protein	
1037	3432	NEOS13_1021	peg	3432_10343_10480	10343	10480	+	hypothetical protein	
1038	3432	NEOS13_1022	peg	3432_10956_10789	10956	10789	-	hypothetical protein	
1039	3432	NEOS13_1023	peg	3432_11325_11861	11325	11861	+	Peptide deformylase (EC 3.5.1.88)	
1040	3432	NEOS13_1024	peg	3432_12145_12594	12145	12594	+	hypothetical protein	
1041	3432	NEOS13_1025	peg	3432_12937_12641	12937	12641	-	Preprotein translocase subunit SecG (TC 3.A.5.1.1)	
1042	3432	NEOS13_1026	peg	3432_13725_12934	13725	12934	-	Triosephosphate isomerase (EC 5.3.1.1)	Glycolysis_Glucogenesis
1043	3432	NEOS13_1027	peg	3432_13697_13822	13697	13822	+	hypothetical protein	
1044	3432	NEOS13_1028	peg	3432_14069_15424	14069	15424	+	Exodeoxyribonuclease VII large subunit (EC 3.1.11.6)	

1045	3432	NEOS13_1029	peg	3432_15417_15668	15417	15668	+	Exodeoxyribonuclease VII small subunit (EC 3.1.11.6)	
1046	3432	NEOS13_1030	peg	3432_15688_17589	15688	17589	+	1-deoxy-D-xylulose 5-phosphate synthase (EC 2.2.1.7)	
1047	3432	NEOS13_1031	peg	3432_17599_18450	17599	18450	+	NAD kinase (EC 2.7.1.23)	
1048	3432	NEOS13_1032	peg	3432_18914_19081	18914	19081	+	hypothetical protein	
1049	3432	NEOS13_1033	peg	3432_19708_21342	19708	21342	+	hypothetical protein	Outer_membrane_protein
1050	3432	NEOS13_1034	peg	3432_21612_21932	21612	21932	+	hypothetical protein	
1051	3432	NEOS13_1035	peg	3432_21958_22542	21958	22542	+	Septum formation protein Maf	
1052	3432	NEOS13_1036	peg	3432_22532_24241	22532	24241	+	hypothetical protein	
1053	3432	NEOS13_1037	peg	3432_24640_26826	24640	26826	+	hypothetical protein	
1054	3432	NEOS13_1038	peg	3432_26909_28267	26909	28267	+	UDP-glucose dehydrogenase (EC 1.1.1.22)	
1055	3432	NEOS13_1039	peg	3432_28318_29193	28318	29193	+	Octaprenyl-diphosphate synthase (EC 2.5.1.-) / Dimethylallyltransferase (EC 2.5.1.1) / Geranyltranstransferase (farnesyldiphosphate synthase) (EC 2.5.1.10) / Geranylgeranyl pyrophosphate synthetase (EC 2.5.1.29)	
1056	3432	NEOS13_1040	peg	3432_29862_30569	29862	30569	+	Undecaprenyl-phosphate galactosephosphotransferase (EC 2.7.8.6)	
1057	3432	NEOS13_1041	peg	3432_30735_31238	30735	31238	+	hypothetical protein	
1058	3432	NEOS13_1042	peg	3432_31240_31617	31240	31617	+	Ferredoxin, 2Fe-2S	
1059	3432	NEOS13_1043	peg	3432_31580_32863	31580	32863	+	ATPase, AAA family protein	
1060	3432	NEOS13_1044	peg	3432_33936_33388	33936	33388	-	hypothetical protein	
1061	3432	NEOS13_1045	peg	3432_34588_34707	34588	34707	+	hypothetical protein	
1062	3432	NEOS13_1046	peg	3432_34751_35641	34751	35641	+	Methylenetetrahydrofolate dehydrogenase (NADP+) (EC 1.5.1.5) / Methenyltetrahydrofolate cyclohydrolase (EC 3.5.4.9)	
1063	3432	NEOS13_1047	peg	3432_35663_36715	35663	36715	+	Thiamin biosynthesis lipoprotein ApbE	
1064	3432	NEOS13_1048	peg	3432_36932_36705	36932	36705	-	hypothetical protein	
1065	3432	NEOS13_1049	peg	3432_37080_36958	37080	36958	-	hypothetical protein	
1066	3432	NEOS13_1050	peg	3432_37230_38561	37230	38561	+	hypothetical protein	
1067	3432	NEOS13_1051	peg	3432_39624_38590	39624	38590	-	DNA recombination and repair protein RecF	
1068	3432	NEOS13_1052	peg	3432_40782_39646	40782	39646	-	DNA polymerase III beta subunit (EC 2.7.7.7)	
1069	3432	NEOS13_1053	peg	3432_41005_41460	41005	41460	+	tmRNA-binding protein SmpB	
1070	3432	NEOS13_1054	peg	3432_41508_41930	41508	41930	+	LSU m3Psi1915 methyltransferase RlmH	
1071	3432	NEOS13_1055	peg	3432_42511_42011	42511	42011	-	hypothetical protein	
1072	3432	NEOS13_1056	peg	3432_42898_43041	42898	43041	+	hypothetical protein	
1073	3432	NEOS13_1057	peg	3432_42956_43411	42956	43411	+	Holo-[acyl-carrier protein] synthase (EC 2.7.8.7)	
1074	3432	NEOS13_1058	peg	3432_43603_43761	43603	43761	+	hypothetical protein	
1075	3433	NEOS13_1059	peg	3433_638_78	638	78	-	Leucine-rich repeat containing protein	Leucine-rich repeat
1076	3434	NEOS13_1060	peg	3434_1458_1273	1458	1273	-	hypothetical protein	
1077	3435	NEOS13_1061	peg	3435_1331_138	1331	138	-	Leucine-rich repeat containing protein	Leucine-rich repeat
1078	3436	NEOS13_1062	peg	3436_1838_138	1838	138	-	Leucine-rich repeat containing protein	Leucine-rich repeat
1079	3436	NEOS13_1063	peg	3436_3384_2326	3384	2326	-	Leucine-rich repeat containing protein	Leucine-rich repeat
1080	3437	NEOS13_1064	peg	3437_1215_952	1215	952	-	Leucine-rich repeat containing protein	Leucine-rich repeat
1081	3438	NEOS13_1065	peg	3438_2374_1025	2374	1025	-	Leucine-rich repeat containing protein	Leucine-rich repeat
1082	3439	NEOS13_1066	peg	3439_193_327	193	327	+	hypothetical protein	
1083	3439	NEOS13_1067	peg	3439_561_448	561	448	-	hypothetical protein	
1084	3439	NEOS13_1068	peg	3439_908_735	908	735	-	Mobile element protein	
1085	3439	NEOS13_1069	peg	3439_1381_944	1381	944	-	Mobile element protein	
1086	3440	NEOS13_1070	peg	3440_229_438	229	438	+	hypothetical protein	
1087	3440	NEOS13_1071	peg	3440_539_748	539	748	+	hypothetical protein	
1088	3440	NEOS13_1072	peg	3440_2540_1467	2540	1467	-	dTDP-glucose 4,6-dehydratase (EC 4.2.1.46)	
1089	3440	NEOS13_1073	peg	3440_3430_2537	3430	2537	-	Glucose-1-phosphate thymidylyltransferase (EC 2.7.7.24)	
1090	3440	NEOS13_1074	peg	3440_3728_5374	3728	5374	+	Thymidylate synthase thyX (EC 2.1.1.-)	
1091	3440	NEOS13_1075	peg	3440_5850_7067	5850	7067	+	Tyrosine-specific transport protein	
1092	3440	NEOS13_1076	peg	3440_7152_8309	7152	8309	+	Tyrosine-specific transport protein	
1093	3440	NEOS13_1077	peg	3440_8322_9404	8322	9404	+	Tryptophanyl-tRNA synthetase (EC 6.1.1.2)	
1094	3440	NEOS13_1078	peg	3440_9972_9850	9972	9850	-	hypothetical protein	
1095	3440	NEOS13_1079	peg	3440_10027_12021	10027	12021	+	Excinuclease ABC subunit B	ABC_transporter
1096	3440	NEOS13_1080	peg	3440_12632_12841	12632	12841	+	conserved hypothetical protein	
1097	3440	NEOS13_1081	peg	3440_13343_14686	13343	14686	+	Mobile element protein	
1098	3441	NEOS13_1082	peg	3441_6326_609	6326	609	-	hypothetical protein	
1099	3441	NEOS13_1083	peg	3441_7079_9841	7079	9841	+	hypothetical protein	
1100	3441	NEOS13_1084	peg	3441_13004_10488	13004	10488	-	Leucyl-tRNA synthetase (EC 6.1.1.4)	
1101	3441	NEOS13_1085	peg	3441_13061_15373	13061	15373	+	3'-to-5' exoribonuclease RNase R	
1102	3441	NEOS13_1086	peg	3441_15373_15957	15373	15957	+	DNA-3-methyladenine glycosylase II (EC 3.2.2.21)	
1103	3441	NEOS13_1087	peg	3441_16069_16740	16069	16740	+	hypothetical protein	
1104	3441	NEOS13_1088	peg	3441_16737_17396	16737	17396	+	hypothetical protein	
1105	3441	NEOS13_1089	peg	3441_17383_17811	17383	17811	+	hypothetical protein	
1106	3441	NEOS13_1090	peg	3441_18119_17874	18119	17874	-	hypothetical protein	
1107	3441	NEOS13_1091	peg	3441_19404_19285	19404	19285	-	hypothetical protein	
1108	3441	NEOS13_1092	peg	3441_19740_20267	19740	20267	+	putative NADH-ubiquinone oxidoreductase chain C/D	Oxidative_Phosphorylation
1109	3441	NEOS13_1093	peg	3441_20949_20458	20949	20458	-	transposase	Transposase

1110	3441	NEOS13_1094	peg	3441_21356_21060	21356	21060	-	Transposase	Transposase
1111	3441	NEOS13_1095	peg	3441_21532_22947	21532	22947	+	hypothetical protein	
1112	3441	NEOS13_1096	peg	3441_23273_23157	23273	23157	-	hypothetical protein	
1113	3441	NEOS13_1097	peg	3441_23603_23469	23603	23469	-	hypothetical protein	
1114	3441	NEOS13_1098	peg	3441_25637_24063	25637	24063	-	peptidase C26	
1115	3441	NEOS13_1099	peg	3441_26836_26096	26836	26096	-	hypothetical protein	
1116	3441	NEOS13_1100	peg	3441_28547_28684	28547	28684	+	hypothetical protein	
1117	3441	NEOS13_1101	peg	3441_28881_29108	28881	29108	+	hypothetical protein	
1118	3441	NEOS13_1102	peg	3441_29120_29422	29120	29422	+	hypothetical protein	
1119	3441	NEOS13_1103	peg	3441_29878_30054	29878	30054	+	hypothetical protein	
1120	3441	NEOS13_0017ma	rna	3441_30549_30478	30549	30478	-	tRNA-Asn-GTT	RNA
1121	3441	NEOS13_1104	peg	3441_32252_30627	32252	30627	-	Heat shock protein 60 family chaperone GroEL	
1122	3441	NEOS13_1105	peg	3441_32603_32277	32603	32277	-	Heat shock protein 60 family co-chaperone GroES	
1123	3441	NEOS13_1106	peg	3441_34571_32727	34571	32727	-	Oligoendopeptidase F	
1124	3441	NEOS13_1107	peg	3441_37211_35562	37211	35562	-	hypothetical protein	
1125	3441	NEOS13_1108	peg	3441_37510_37397	37510	37397	-	hypothetical protein	
1126	3441	NEOS13_1109	peg	3441_39363_37786	39363	37786	-	hypothetical protein	
1127	3441	NEOS13_1110	peg	3441_39661_39476	39661	39476	-	hypothetical protein	
1128	3441	NEOS13_1111	peg	3441_40305_40421	40305	40421	+	hypothetical protein	
1129	3441	NEOS13_1112	peg	3441_40850_40434	40850	40434	-	FIG00494004: hypothetical protein	
1130	3441	NEOS13_1113	peg	3441_41785_41078	41785	41078	-	Ribose 5-phosphate isomerase A (EC 5.3.1.6)	Pentose_phospate_pathway
1131	3441	NEOS13_1114	peg	3441_41827_42582	41827	42582	+	FIG137478: Hypothetical protein YbgI	
1132	3441	NEOS13_1115	peg	3441_42780_42896	42780	42896	+	hypothetical protein	
1133	3441	NEOS13_1116	peg	3441_43183_44457	43183	44457	+	Tyrosyl-tRNA synthetase (EC 6.1.1.1)	
1134	3441	NEOS13_1117	peg	3441_44690_45007	44690	45007	+	Integration host factor alpha/beta	
1135	3441	NEOS13_1118	peg	3441_45296_46381	45296	46381	+	FIG00899523: hypothetical protein	
1136	3441	NEOS13_1119	peg	3441_46458_47417	46458	47417	+	Acetyl-coenzyme A carboxyl transferase alpha chain (EC 6.4.1.2)	Fatty _acid_initiation_elongation
1137	3441	NEOS13_1120	peg	3441_47610_49529	47610	49529	+	Lipid A export ATP-binding/permease protein MsbA (EC 3.6.3.25)	
1138	3441	NEOS13_1121	peg	3441_49510_50226	49510	50226	+	Queuosine Biosynthesis QueC ATPase	
1139	3441	NEOS13_1122	peg	3441_50819_50535	50819	50535	-	hypothetical protein	
1140	3441	NEOS13_1123	peg	3441_51381_51004	51381	51004	-	hypothetical protein	
1141	3441	NEOS13_1124	peg	3441_52146_54074	52146	54074	+	hypothetical protein	
1142	3441	NEOS13_1125	peg	3441_55295_55170	55295	55170	-	hypothetical protein	
1143	3441	NEOS13_1126	peg	3441_55257_55379	55257	55379	+	hypothetical protein	
1144	3441	NEOS13_1127	peg	3441_55857_55354	55857	55354	-	Mobile element protein	
1145	3441	NEOS13_1128	peg	3441_56911_56090	56911	56090	-	Nucleoside-diphosphate-sugar epimerases	
1146	3441	NEOS13_1129	peg	3441_57618_56926	57618	56926	-	DNA polymerase III epsilon subunit (EC 2.7.7.7)	
1147	3441	NEOS13_1130	peg	3441_58371_57901	58371	57901	-	hypothetical protein	
1148	3441	NEOS13_1131	peg	3441_58565_58389	58565	58389	-	hypothetical protein	
1149	3441	NEOS13_1132	peg	3441_59470_58844	59470	58844	-	hypothetical protein	
1150	3441	NEOS13_1133	peg	3441_59533_60213	59533	60213	+	hypothetical protein	
1151	3441	NEOS13_1134	peg	3441_60215_60985	60215	60985	+	hypothetical protein	
1152	3441	NEOS13_1135	peg	3441_60992_63220	60992	63220	+	Helicase PriA essential for oriC/DnaA-independent DNA replication	
1153	3441	NEOS13_1136	peg	3441_64419_63313	64419	63313	-	hypothetical protein	
1154	3441	NEOS13_1137	peg	3441_65228_66718	65228	66718	+	Ribosomal protein S12p Asp88 (E. coli) methylthiotransferase	
1155	3441	NEOS13_1138	peg	3441_67216_68835	67216	68835	+	Lysyl-tRNA synthetase (class II) (EC 6.1.1.6)	
1156	3441	NEOS13_1139	peg	3441_69064_68948	69064	68948	-	hypothetical protein	
1157	3441	NEOS13_1140	peg	3441_69311_75283	69311	75283	+	hypothetical protein	
1158	3441	NEOS13_1141	peg	3441_75917_75666	75917	75666	-	hypothetical protein	
1159	3441	NEOS13_1142	peg	3441_77373_75925	77373	75925	-	Cysteinyl-tRNA synthetase (EC 6.1.1.16)	
1160	3441	NEOS13_1143	peg	3441_80195_77463	80195	77463	-	Long-chain-fatty-acid--CoA ligase (EC 6.2.1.3)	
1161	3441	NEOS13_1144	peg	3441_80687_81232	80687	81232	+	Substrate-specific component BioY of biotin ECF transporter	
1162	3441	NEOS13_1145	peg	3441_82836_81661	82836	81661	-	hypothetical protein	
1163	3441	NEOS13_1146	peg	3441_84146_83529	84146	83529	-	OmpA/MotB domain protein	Outer_membrane_protein
1164	3441	NEOS13_1147	peg	3441_84544_84750	84544	84750	+	hypothetical protein	
1165	3441	NEOS13_1148	peg	3441_86209_85613	86209	85613	-	NADPH:quinone oxidoreductase	
1166	3441	NEOS13_1149	peg	3441_86877_86320	86877	86320	-	hypothetical protein	
1167	3441	NEOS13_1150	peg	3441_86839_87018	86839	87018	+	hypothetical protein	
1168	3441	NEOS13_1151	peg	3441_87470_87676	87470	87676	+	hypothetical protein	
1169	3441	NEOS13_1152	peg	3441_87922_88434	87922	88434	+	hypothetical protein	
1170	3441	NEOS13_1153	peg	3441_89427_88903	89427	88903	-	hypothetical protein	
1171	3444	NEOS13_1154	peg	3444_2126_780	2126	780	-	hypothetical protein	
1172	3444	NEOS13_1155	peg	3444_3910_2123	3910	2123	-	Phosphoenolpyruvate carboxykinase [GTP] (EC 4.1.1.32)	TCA
1173	3444	NEOS13_1156	peg	3444_5299_4208	5299	4208	-	Rod shape-determining protein MreB	
1174	3444	NEOS13_1157	peg	3444_8804_5319	8804	5319	-	FIG00899436: hypothetical protein	
1175	3444	NEOS13_0018ma	rna	3444_9592_9663	9592	9663	+	tRNA-Gly-GCC	RNA
1176	3444	NEOS13_0019ma	rna	3444_9697_9768	9697	9768	+	tRNA-Gly-GCC	RNA
1177	3444	NEOS13_1158	peg	3444_9881_11215	9881	11215	+	Cell division trigger factor (EC 5.2.1.8)	
1178	3444	NEOS13_1159	peg	3444_11279_11884	11279	11884	+	ATP-dependent Clp protease proteolytic subunit (EC 3.4.21.92)	

1179	3444	NEOS13_1160	peg	3444_11902_13158	11902	13158	+	ATP-dependent Clp protease ATP-binding subunit ClpX	
1180	3444	NEOS13_1161	peg	3444_13283_14203	13283	14203	+	COG0454: Histone acetyltransferase HPA2 and related acetyltransferases	
1181	3444	NEOS13_1162	peg	3444_14311_14442	14311	14442	+	hypothetical protein	
1182	3444	NEOS13_1163	peg	3444_14785_14663	14785	14663	-	hypothetical protein	
1183	3444	NEOS13_1164	peg	3444_14875_17622	14875	17622	+	hypothetical protein	
1184	3444	NEOS13_1165	peg	3444_17802_19136	17802	19136	+	hypothetical protein	
1185	3444	NEOS13_1166	peg	3444_19280_20608	19280	20608	+	hypothetical protein	
1186	3444	NEOS13_1167	peg	3444_20640_21194	20640	21194	+	putative low calcium response protein H	T3ST1
1187	3444	NEOS13_1168	peg	3444_21353_21979	21353	21979	+	putative low calcium response protein H	T3ST1
1188	3444	NEOS13_1169	peg	3444_21983_22669	21983	22669	+	hypothetical protein	T3ST1
1189	3444	NEOS13_1170	peg	3444_22666_23628	22666	23628	+	hypothetical protein	
1190	3444	NEOS13_1171	peg	3444_23674_24909	23674	24909	+	hypothetical protein	
1191	3444	NEOS13_1172	peg	3444_24954_25130	24954	25130	+	hypothetical protein	
1192	3444	NEOS13_1173	peg	3444_25203_27680	25203	27680	+	hypothetical protein	
1193	3445	NEOS13_1174	peg	3445_1274_1714	1274	1714	+	hypothetical protein	
1194	3445	NEOS13_1175	peg	3445_1931_2359	1931	2359	+	hypothetical protein	
1195	3445	NEOS13_1176	peg	3445_2377_2496	2377	2496	+	hypothetical protein	
1196	3445	NEOS13_1177	peg	3445_2579_5308	2579	5308	+	hypothetical protein	
1197	3446	NEOS13_1178	peg	3446_770_594	770	594	-	hypothetical protein	
1198	3446	NEOS13_1179	peg	3446_886_1029	886	1029	+	hypothetical protein	
1199	3447	NEOS13_1180	peg	3447_89_343	89	343	+	Leucine-rich repeat containing protein	Leucine-rich repeat
1200	3447	NEOS13_1181	peg	3447_931_2757	931	2757	+	Leucine-rich repeat containing protein	Leucine-rich repeat
1201	3448	NEOS13_1182	peg	3448_2417_693	2417	693	-	Leucine-rich repeat containing protein	Leucine-rich repeat
1202	3450	NEOS13_1183	peg	3450_40_687	40	687	+	Leucine-rich repeat containing protein	Leucine-rich repeat
1203	3450	NEOS13_1184	peg	3450_899_3346	899	3346	+	Leucine-rich repeat containing protein	Leucine-rich repeat
1204	3450	NEOS13_1185	peg	3450_3629_3949	3629	3949	+	Leucine-rich repeat containing protein	Leucine-rich repeat
1205	3450	NEOS13_1186	peg	3450_4005_4907	4005	4907	+	Leucine-rich repeat containing protein	Leucine-rich repeat
1206	3450	NEOS13_1187	peg	3450_5126_4941	5126	4941	-	hypothetical protein	
1207	3450	NEOS13_1188	peg	3450_5967_5107	5967	5107	-	hypothetical protein	
1208	3450	NEOS13_1189	peg	3450_6920_5967	6920	5967	-	Thioredoxin reductase (EC 1.8.1.9)	
1209	3450	NEOS13_1190	peg	3450_7193_7459	7193	7459	+	hypothetical protein	
1210	3450	NEOS13_1191	peg	3450_8365_7577	8365	7577	-	hypothetical protein	
1211	3450	NEOS13_1192	peg	3450_9988_8396	9988	8396	-	hypothetical protein	
1212	3450	NEOS13_1193	peg	3450_10700_12955	10700	12955	+	putative bumetanide-sensitive Na-K-Cl	
1213	3450	NEOS13_1194	peg	3450_13179_14678	13179	14678	+	Proline/sodium symporter PutP (TC 2.A.21.2.1) @ Propionate/sodium symporter	
1214	3450	NEOS13_1195	peg	3450_18459_14989	18459	14989	-	hypothetical protein	
1215	3450	NEOS13_1196	peg	3450_18647_18841	18647	18841	+	hypothetical protein	
1216	3450	NEOS13_1197	peg	3450_20047_19808	20047	19808	-	Acyl carrier protein	
1217	3450	NEOS13_1198	peg	3450_20870_20130	20870	20130	-	3-oxoacyl-[acyl-carrier protein] reductase (EC 1.1.1.100)	Fatty _acid_initiation_elongation
1218	3450	NEOS13_1199	peg	3450_21823_20882	21823	20882	-	Malonyl CoA-acyl carrier protein transacylase (EC 2.3.1.39)	
1219	3450	NEOS13_1200	peg	3450_22862_21846	22862	21846	-	3-oxoacyl-[acyl-carrier-protein] synthase, KASIII (EC 2.3.1.41)	Fatty _acid_initiation_elongation
1220	3450	NEOS13_1201	peg	3450_23236_23832	23236	23832	+	Recombination protein RecR	
1221	3450	NEOS13_1202	peg	3450_23932_26340	23932	26340	+	Outer membrane protein assembly factor YaeT precursor	
1222	3450	NEOS13_1203	peg	3450_26368_26964	26368	26964	+	Outer membrane protein H precursor	
1223	3450	NEOS13_1204	peg	3450_26982_28040	26982	28040	+	UDP-3-O-[3-hydroxymyristoyl] glucosamine N-acyltransferase (EC 2.3.1.-)	
1224	3450	NEOS13_1205	peg	3450_30421_28346	30421	28346	-	hypothetical protein	
1225	3450	NEOS13_1206	peg	3450_30577_30437	30577	30437	-	hypothetical protein	
1226	3452	NEOS13_1207	peg	3452_542_721	542	721	+	hypothetical protein	
1227	3452	NEOS13_1208	peg	3452_1730_1599	1730	1599	-	hypothetical protein	
1228	3452	NEOS13_1209	peg	3452_2345_2962	2345	2962	+	hypothetical protein	
1229	3452	NEOS13_1210	peg	3452_3909_3010	3909	3010	-	Octaprenyl-diphosphate synthase (EC 2.5.1.-) / Dimethylallyltransferase (EC 2.5.1.1) / Geranyltranstransferase (farnesyldiphosphate synthase) (EC 2.5.1.10) / Geranylgeranyl pyrophosphate synthetase (EC 2.5.1.29)	
1230	3452	NEOS13_1211	peg	3452_4552_3959	4552	3959	-	Glucosamine-1-phosphate N-acetyltransferase (EC 2.3.1.157)	
1231	3452	NEOS13_1212	peg	3452_4929_5111	4929	5111	+	hypothetical protein	
1232	3452	NEOS13_1213	peg	3452_5209_6333	5209	6333	+	putative 23S rRNA (Uracil-5-)-methyltransferase	
1233	3452	NEOS13_1214	peg	3452_6476_6357	6476	6357	-	hypothetical protein	
1234	3452	NEOS13_0020rna	rna	3452_7029_6955	7029	6955	-	tRNA-Pro-GGG	RNA
1235	3452	NEOS13_1215	peg	3452_7295_7041	7295	7041	-	hypothetical protein	
1236	3452	NEOS13_1216	peg	3452_7424_7305	7424	7305	-	hypothetical protein	
1237	3452	NEOS13_1217	peg	3452_7502_8191	7502	8191	+	Uracil-DNA glycosylase, family 1	
1238	3452	NEOS13_1218	peg	3452_8201_8341	8201	8341	+	hypothetical protein	
1239	3452	NEOS13_1219	peg	3452_8533_8931	8533	8931	+	hypothetical protein	
1240	3452	NEOS13_1220	peg	3452_8996_10159	8996	10159	+	hypothetical protein	
1241	3452	NEOS13_1221	peg	3452_10296_10454	10296	10454	+	hypothetical protein	
1242	3452	NEOS13_1222	peg	3452_11083_10817	11083	10817	-	Mobile element protein	
1243	3452	NEOS13_1223	peg	3452_12274_11654	12274	11654	-	Leucine-rich repeat containing protein	Leucine-rich repeat
1244	3453	NEOS13_1224	peg	3453_232_1557	232	1557	+	hypothetical protein	
1245	3453	NEOS13_1225	peg	3453_1723_1893	1723	1893	+	Mobile element protein	
1246	3453	NEOS13_1226	peg	3453_2135_2275	2135	2275	+	Mobile element protein	

1247	3453	NEOS13_1227	peg	3453_2611_2775	2611	2775	+	Integrase, catalytic region	
1248	3453	NEOS13_1228	peg	3453_3053_6259	3053	6259	+	hypothetical protein	
1249	3453	NEOS13_1229	peg	3453_6730_8781	6730	8781	+	hypothetical protein	
1250	3453	NEOS13_1230	peg	3453_9241_9128	9241	9128	-	hypothetical protein	
1251	3453	NEOS13_1231	peg	3453_9505_9389	9505	9389	-	hypothetical protein	
1252	3453	NEOS13_1232	peg	3453_9603_10487	9603	10487	+	hypothetical protein	
1253	3453	NEOS13_1233	peg	3453_10451_11071	10451	11071	+	putative ATP/GTP-binding protein	
1254	3453	NEOS13_1234	peg	3453_11209_12297	11209	12297	+	hypothetical protein	
1255	3453	NEOS13_1235	peg	3453_12768_13979	12768	13979	+	hypothetical protein	
1256	3453	NEOS13_1236	peg	3453_14231_14106	14231	14106	-	hypothetical protein	
1257	3453	NEOS13_1237	peg	3453_14305_17160	14305	17160	+	hypothetical protein	
1258	3453	NEOS13_1238	peg	3453_17581_17447	17581	17447	-	hypothetical protein	
1259	3453	NEOS13_1239	peg	3453_19881_18007	19881	18007	-	hypothetical protein	
1260	3453	NEOS13_1240	peg	3453_20456_20001	20456	20001	-	hypothetical protein	
1261	3453	NEOS13_1241	peg	3453_20765_20574	20765	20574	-	hypothetical protein	
1262	3453	NEOS13_1242	peg	3453_20867_21607	20867	21607	+	putative signal peptide	
1263	3453	NEOS13_1243	peg	3453_22194_22078	22194	22078	-	hypothetical protein	
1264	3453	NEOS13_1244	peg	3453_22312_22196	22312	22196	-	hypothetical protein	
1265	3454	NEOS13_1245	peg	3454_1036_344	1036	344	-	Leucine-rich repeat containing protein	Leucine-rich repeat
1266	3455	NEOS13_1246	peg	3455_170_1057	170	1057	+	hypothetical protein	
1267	3455	NEOS13_1247	peg	3455_1359_1123	1359	1123	-	Mobile element protein	
1268	3456	NEOS13_1248	peg	3456_170_1057	170	1057	+	transposase	Transposase
1269	3457	NEOS13_1249	peg	3457_1339_344	1339	344	-	Leucine-rich repeat containing protein	Leucine-rich repeat
1270	3458	NEOS13_1250	peg	3458_42_1367	42	1367	+	Leucine-rich repeat containing protein	Leucine-rich repeat
1271	3459	NEOS13_1251	peg	3459_52_918	52	918	+	Leucine-rich repeat containing protein	Leucine-rich repeat
1272	3460	NEOS13_1252	peg	3460_429_295	429	295	-	hypothetical protein	
1273	3460	NEOS13_1253	peg	3460_1303_626	1303	626	-	Cytidylate kinase (EC 2.7.4.14)	
1274	3460	NEOS13_1254	peg	3460_2182_1328	2182	1328	-	Phosphatidate cytidylyltransferase (EC 2.7.7.41)	
1275	3460	NEOS13_1255	peg	3460_2937_2179	2937	2179	-	Undecaprenyl pyrophosphate synthetase (EC 2.5.1.31)	
1276	3460	NEOS13_1256	peg	3460_4266_2947	4266	2947	-	Adenylosuccinate synthetase (EC 6.3.4.4)	
1277	3460	NEOS13_1257	peg	3460_6098_4290	6098	4290	-	Translation elongation factor LepA	
1278	3460	NEOS13_1258	peg	3460_6926_6291	6926	6291	-	Thiamin pyrophosphokinase (EC 2.7.6.2)	
1279	3460	NEOS13_0021ma	rna	3460_7043_6960	7043	6960	-	tRNA-Leu-CAG	RNA
1280	3460	NEOS13_1259	peg	3460_7709_7098	7709	7098	-	hypothetical protein	
1281	3460	NEOS13_1260	peg	3460_7877_9028	7877	9028	+	Sulfite reductase [NADPH] flavoprotein alpha-component (EC 1.8.1.2)	
1282	3460	NEOS13_1261	peg	3460_9615_9118	9615	9118	-	2-C-methyl-D-erythritol 2,4-cyclodiphosphate synthase (EC 4.6.1.12)	
1283	3460	NEOS13_1262	peg	3460_9966_10982	9966	10982	+	putative lipase transmembrane protein	
1284	3460	NEOS13_1263	peg	3460_11038_12585	11038	12585	+	UDP-N-acetylglucosamine 1-carboxyvinyltransferase (EC 2.5.1.7)	
1285	3460	NEOS13_1264	peg	3460_13369_13956	13369	13956	+	hypothetical protein	
1286	3460	NEOS13_1265	peg	3460_14186_15685	14186	15685	+	hypothetical protein	
1287	3460	NEOS13_1266	peg	3460_16039_15872	16039	15872	-	hypothetical protein	
1288	3460	NEOS13_1267	peg	3460_16170_16364	16170	16364	+	Mobile element protein	
1289	3460	NEOS13_1268	peg	3460_16621_17490	16621	17490	+	Lysyl-lysine 2,3-aminomutase	
1290	3460	NEOS13_1269	peg	3460_17818_17516	17818	17516	-	hypothetical protein	
1291	3460	NEOS13_1270	peg	3460_18011_17898	18011	17898	-	hypothetical protein	
1292	3461	NEOS13_1271	peg	3461_1086_652	1086	652	-	hypothetical protein	
1293	3461	NEOS13_1272	peg	3461_1604_2995	1604	2995	+	hypothetical protein	
1294	3461	NEOS13_1273	peg	3461_3149_3298	3149	3298	+	hypothetical protein	
1295	3461	NEOS13_1274	peg	3461_3328_3465	3328	3465	+	hypothetical protein	
1296	3461	NEOS13_1275	peg	3461_3906_3739	3906	3739	-	hypothetical protein	
1297	3461	NEOS13_1276	peg	3461_4431_4616	4431	4616	+	hypothetical protein	
1298	3461	NEOS13_1277	peg	3461_4774_4965	4774	4965	+	hypothetical protein	
1299	3461	NEOS13_1278	peg	3461_5010_5207	5010	5207	+	hypothetical protein	
1300	3461	NEOS13_1279	peg	3461_5580_5762	5580	5762	+	Mobile element protein	
1301	3461	NEOS13_1280	peg	3461_6379_6573	6379	6573	+	hypothetical protein	
1302	3461	NEOS13_1281	peg	3461_6619_6867	6619	6867	+	hypothetical protein	
1303	3461	NEOS13_1282	peg	3461_7344_7219	7344	7219	-	hypothetical protein	
1304	3461	NEOS13_1283	peg	3461_7454_7741	7454	7741	+	hypothetical protein	
1305	3461	NEOS13_1284	peg	3461_8050_8244	8050	8244	+	hypothetical protein	
1306	3461	NEOS13_1285	peg	3461_11279_8592	11279	8592	-	COG0553: Superfamily II DNA/RNA helicases, SNF2 family	
1307	3461	NEOS13_1286	peg	3461_11356_12183	11356	12183	+	hypothetical protein	
1308	3461	NEOS13_1287	peg	3461_13227_12265	13227	12265	-	Rhodanese domain protein UPF0176, Firmicutes subgroup	
1309	3461	NEOS13_1288	peg	3461_14112_13252	14112	13252	-	hypothetical protein	
1310	3461	NEOS13_1289	peg	3461_14749_15069	14749	15069	+	Thioredoxin	
1311	3461	NEOS13_1290	peg	3461_15836_15480	15836	15480	-	tRNA (cytosine34-2'-O-)-methyltransferase (EC 2.1.1.-)	
1312	3461	NEOS13_1291	peg	3461_16834_16016	16834	16016	-	putative mip (macrophage infectivity potentiator, fkbp-type peptidyl-prolyl cis-trans isomerase)	
1313	3461	NEOS13_1292	peg	3461_18680_16893	18680	16893	-	Aspartyl-tRNA synthetase (EC 6.1.1.12)	
1314	3461	NEOS13_1293	peg	3461_20138_18690	20138	18690	-	Histidyl-tRNA synthetase (EC 6.1.1.21)	
1315	3461	NEOS13_1294	peg	3461_20375_21529	20375	21529	+	hypothetical protein	
1316	3461	NEOS13_1295	peg	3461_24292_21728	24292	21728	-	hypothetical protein	
1317	3461	NEOS13_1296	peg	3461_24532_25884	24532	25884	+	Hexose phosphate uptake regulatory protein UhpC	

1318	3461	NEOS13_1297	peg	3461_26465_30214	26465	30214	+	DNA polymerase III alpha subunit (EC 2.7.7.7)	
1319	3461	NEOS13_1298	peg	3461_31368_32372	31368	32372	+	hypothetical protein	
1320	3461	NEOS13_1299	peg	3461_32379_32939	32379	32939	+	hypothetical protein	
1321	3461	NEOS13_1300	peg	3461_33376_34908	33376	34908	+	transposase	Transposase
1322	3461	NEOS13_1301	peg	3461_35505_35080	35505	35080	-	hypothetical protein	
1323	3461	NEOS13_1302	peg	3461_35782_36981	35782	36981	+	D-alanyl-D-alanine carboxypeptidase (EC 3.4.16.4)	
1324	3461	NEOS13_1303	peg	3461_37874_37996	37874	37996	+	hypothetical protein	
1325	3461	NEOS13_1304	peg	3461_38263_38469	38263	38469	+	hypothetical protein	
1326	3461	NEOS13_1305	peg	3461_39301_38453	39301	38453	-	SSU rRNA (adenine(1518)-N(6)/adenine(1519)-N(6))-dimethyltransferase (EC 2.1.1.182)	
1327	3461	NEOS13_1306	peg	3461_40486_39404	40486	39404	-	hypothetical protein	
1328	3461	NEOS13_1307	peg	3461_41731_40604	41731	40604	-	Ribosomal RNA small subunit methyltransferase B (EC 2.1.1.-)	
1329	3461	NEOS13_1308	peg	3461_41854_41741	41854	41741	-	hypothetical protein	
1330	3461	NEOS13_1309	peg	3461_42204_42320	42204	42320	+	hypothetical protein	
1331	3461	NEOS13_1310	peg	3461_42476_42360	42476	42360	-	hypothetical protein	
1332	3461	NEOS13_1311	peg	3461_42538_44202	42538	44202	+	Apolipoprotein N-acyltransferase (EC 2.3.1.-)	
1333	3461	NEOS13_1312	peg	3461_45020_45898	45020	45898	+	UDP-3-O-[3-hydroxymyristoyl] N-acetylglucosamine deacetylase (EC 3.5.1.-)	
1334	3461	NEOS13_1313	peg	3461_45898_46365	45898	46365	+	(3R)-hydroxymyristoyl-[acyl carrier protein] dehydratase (EC 4.2.1.-)	Fatty _acid_initiation_elongation
1335	3461	NEOS13_1314	peg	3461_46409_47272	46409	47272	+	Acyl-[acyl-carrier-protein]--UDP-N-acetylglucosamine O-acyltransferase (EC 2.3.1.129)	
1336	3461	NEOS13_1315	peg	3461_47344_48291	47344	48291	+	Methionyl-tRNA formyltransferase (EC 2.1.2.9)	
1337	3461	NEOS13_1316	peg	3461_48608_48754	48608	48754	+	hypothetical protein	
1338	3461	NEOS13_1317	peg	3461_49055_49972	49055	49972	+	hypothetical protein	
1339	3461	NEOS13_1318	peg	3461_50029_51312	50029	51312	+	hypothetical protein	
1340	3461	NEOS13_1319	peg	3461_51605_51342	51605	51342	-	hypothetical protein	
1341	3461	NEOS13_1320	peg	3461_51747_51634	51747	51634	-	hypothetical protein	
1342	3461	NEOS13_1321	peg	3461_52410_52523	52410	52523	+	hypothetical protein	
1343	3461	NEOS13_1322	peg	3461_52885_53448	52885	53448	+	hypothetical protein	
1344	3461	NEOS13_1323	peg	3461_53683_54354	53683	54354	+	LSU ribosomal protein L3p (L3e)	
1345	3461	NEOS13_1324	peg	3461_54378_55067	54378	55067	+	LSU ribosomal protein L4p (L1e)	
1346	3461	NEOS13_1325	peg	3461_55112_55447	55112	55447	+	LSU ribosomal protein L23p (L23Ae)	
1347	3461	NEOS13_1326	peg	3461_55467_56312	55467	56312	+	LSU ribosomal protein L2p (L8e)	
1348	3461	NEOS13_1327	peg	3461_56315_56596	56315	56596	+	SSU ribosomal protein S19p (S15e)	
1349	3461	NEOS13_1328	peg	3461_56593_56934	56593	56934	+	LSU ribosomal protein L22p (L17e)	
1350	3461	NEOS13_1329	peg	3461_56938_57582	56938	57582	+	SSU ribosomal protein S3p (S3e)	
1351	3461	NEOS13_1330	peg	3461_57585_58004	57585	58004	+	LSU ribosomal protein L16p (L10e)	
1352	3461	NEOS13_1331	peg	3461_58014_58214	58014	58214	+	putative 50S ribosomal protein L29	
1353	3461	NEOS13_1332	peg	3461_58242_58481	58242	58481	+	SSU ribosomal protein S17p (S11e)	
1354	3461	NEOS13_1333	peg	3461_58515_58883	58515	58883	+	LSU ribosomal protein L14p (L23e)	
1355	3461	NEOS13_1334	peg	3461_58899_59258	58899	59258	+	50S ribosomal protein L24	
1356	3461	NEOS13_1335	peg	3461_59267_59824	59267	59824	+	LSU ribosomal protein L5p (L11e)	
1357	3461	NEOS13_1336	peg	3461_59841_60245	59841	60245	+	SSU ribosomal protein S8p (S15Ae)	
1358	3461	NEOS13_1337	peg	3461_60284_60829	60284	60829	+	LSU ribosomal protein L6p (L9e)	
1359	3461	NEOS13_1338	peg	3461_60987_61232	60987	61232	+	LSU ribosomal protein L18p (L5e)	
1360	3461	NEOS13_1339	peg	3461_61264_61764	61264	61764	+	SSU ribosomal protein S5p (S2e)	
1361	3461	NEOS13_1340	peg	3461_61761_62210	61761	62210	+	LSU ribosomal protein L15p (L27Ae)	
1362	3461	NEOS13_1341	peg	3461_62244_63617	62244	63617	+	Preprotein translocase secY subunit (TC 3.A.5.1.1)	
1363	3461	NEOS13_1342	peg	3461_63777_64145	63777	64145	+	SSU ribosomal protein S13p (S18e)	
1364	3461	NEOS13_1343	peg	3461_64167_64580	64167	64580	+	SSU ribosomal protein S11p (S14e)	
1365	3461	NEOS13_1344	peg	3461_64620_65735	64620	65735	+	DNA-directed RNA polymerase alpha subunit (EC 2.7.7.6)	
1366	3461	NEOS13_1345	peg	3461_65810_66181	65810	66181	+	LSU ribosomal protein L17p	
1367	3461	NEOS13_1346	peg	3461_66277_67521	66277	67521	+	FIG047466: hypothetical protein	
1368	3461	NEOS13_1347	peg	3461_69569_68136	69569	68136	-	hypothetical protein	
1369	3462	NEOS13_1348	peg	3462_958_110	958	110	-	Leucine-rich repeat containing protein	Leucine-rich repeat
1370	3463	NEOS13_1349	peg	3463_166_279	166	279	+	hypothetical protein	
1371	3463	NEOS13_1350	peg	3463_308_649	308	649	+	Transposase, IS4 family protein	Transposase
1372	3463	NEOS13_1351	peg	3463_2078_1014	2078	1014	-	Leucine-rich repeat containing protein	Leucine-rich repeat
1373	3463	NEOS13_1352	peg	3463_2053_2184	2053	2184	+	hypothetical protein	
1374	3463	NEOS13_1353	peg	3463_3984_2362	3984	2362	-	Leucine-rich repeat containing protein	Leucine-rich repeat
1375	3463	NEOS13_1354	peg	3463_6029_4269	6029	4269	-	Leucine-rich repeat containing protein	Leucine-rich repeat
1376	3463	NEOS13_1355	peg	3463_7518_6316	7518	6316	-	Leucine-rich repeat containing protein	Leucine-rich repeat
1377	3463	NEOS13_1356	peg	3463_9580_7751	9580	7751	-	Leucine-rich repeat containing protein	Leucine-rich repeat
1378	3463	NEOS13_1357	peg	3463_10932_9868	10932	9868	-	Leucine-rich repeat containing protein	Leucine-rich repeat
1379	3463	NEOS13_1358	peg	3463_10931_11068	10931	11068	+	hypothetical protein	
1380	3463	NEOS13_1359	peg	3463_11796_11323	11796	11323	-	hypothetical protein	
1381	3463	NEOS13_1360	peg	3463_12799_11894	12799	11894	-	Translation elongation factor P Lys34:lysine transferase	
1382	3463	NEOS13_1361	peg	3463_13417_12848	13417	12848	-	Translation elongation factor P	
1383	3463	NEOS13_1362	peg	3463_13685_13810	13685	13810	+	hypothetical protein	
1384	3463	NEOS13_1363	peg	3463_13851_14012	13851	14012	+	hypothetical protein	
1385	3463	NEOS13_1364	peg	3463_19465_14045	19465	14045	-	hypothetical protein	
1386	3463	NEOS13_1365	peg	3463_19851_19976	19851	19976	+	hypothetical protein	
1387	3464	NEOS13_1366	peg	3464_1044_595	1044	595	-	hypothetical protein	
1388	3465	NEOS13_1367	peg	3465_188_394	188	394	+	Leucine-rich repeat containing protein	Leucine-rich repeat
1389	3466	NEOS13_1368	peg	3466_263_96	263	96	-	hypothetical protein	
1390	3466	NEOS13_1369	peg	3466_804_454	804	454	-	Leucine-rich repeat containing protein	Leucine-rich repeat

1391	3467	NEOS13_1370	peg	3467_345_136	345	136	-	hypothetical protein	
1392	3467	NEOS13_1371	peg	3467_1500_712	1500	712	-	hypothetical protein	
1393	3467	NEOS13_1372	peg	3467_1643_1783	1643	1783	+	hypothetical protein	
1394	3468	NEOS13_1373	peg	3468_251_99	251	99	-	hypothetical protein	
1395	3469	NEOS13_1374	peg	3469_61_621	61	621	+	hypothetical protein	
1396	3469	NEOS13_1375	peg	3469_853_4506	853	4506	+	hypothetical protein	
1397	3469	NEOS13_1376	peg	3469_5659_4496	5659	4496	-	Cell division protein FtsW	
1398	3469	NEOS13_1377	peg	3469_6446_5697	6446	5697	-	Membrane-bound lytic murein transglycosylase D precursor (EC 3.2.1.-)	
1399	3469	NEOS13_1378	peg	3469_7813_6473	7813	6473	-	UDP-N-acetylmuramoylalanine--D-glutamate ligase (EC 6.3.2.9)	
1400	3469	NEOS13_1379	peg	3469_9021_7825	9021	7825	-	Phospho-N-acetylmuramoyl-pentapeptide-transferase (EC 2.7.8.13)	
1401	3469	NEOS13_1380	peg	3469_10429_9056	10429	9056	-	UDP-N-acetylmuramoylalanyl-D-glutamyl-2,6-diaminopimelate--D-alanyl-D-alanine ligase (EC 6.3.2.10)	
1402	3469	NEOS13_1381	peg	3469_11017_10841	11017	10841	-	hypothetical protein	
1403	3469	NEOS13_1382	peg	3469_11724_11101	11724	11101	-	hypothetical protein	
1404	3470	NEOS13_1383	peg	3470_376_1017	376	1017	+	Leucine-rich repeat containing protein	Leucine-rich repeat
1405	3470	NEOS13_1384	peg	3470_1327_1172	1327	1172	-	hypothetical protein	
1406	3470	NEOS13_1385	peg	3470_1373_1594	1373	1594	+	hypothetical protein	
1407	3470	NEOS13_1386	peg	3470_1563_1871	1563	1871	+	Leucine-rich repeat containing protein	Leucine-rich repeat
1408	3470	NEOS13_1387	peg	3470_2018_2131	2018	2131	+	hypothetical protein	
1409	3471	NEOS13_1388	peg	3471_110_1003	110	1003	+	Leucine-rich repeat containing protein	Leucine-rich repeat
1410	3471	NEOS13_1389	peg	3471_1519_2004	1519	2004	+	Leucine-rich repeat containing protein	Leucine-rich repeat
1411	3471	NEOS13_1390	peg	3471_2029_2514	2029	2514	+	Leucine-rich repeat containing protein	Leucine-rich repeat
1412	3471	NEOS13_1391	peg	3471_3090_2929	3090	2929	-	hypothetical protein	
1413	3472	NEOS13_1392	peg	3472_496_1047	496	1047	+	DNA polymerase III beta subunit (EC 2.7.7.7)	
1414	3472	NEOS13_1393	peg	3472_1270_1148	1270	1148	-	hypothetical protein	
1415	3473	NEOS13_1394	peg	3473_358_633	358	633	+	cytoplasmic membrane protein	
1416	3473	NEOS13_1395	peg	3473_1296_1159	1296	1159	-	hypothetical protein	
1417	3473	NEOS13_1396	peg	3473_1600_1391	1600	1391	-	Protein YidD	
1418	3473	NEOS13_1397	peg	3473_2391_1597	2391	1597	-	hypothetical protein	
1419	3473	NEOS13_0022rna	rna	3473_2648_2565	2648	2565	-	tRNA-Leu-GAG	RNA
1420	3473	NEOS13_1397	peg	3473_3461_2778	3461	2778	-	DNA repair protein RecO	
1421	3473	NEOS13_0023rna	rna	3473_3549_3621	3549	3621	+	tRNA-Arg-TCG	RNA
1422	3473	NEOS13_1399	peg	3473_3636_4703	3636	4703	+	Ribosomal RNA large subunit methyltransferase N (EC 2.1.1.-)	
1423	3473	NEOS13_1400	peg	3473_4989_4828	4989	4828	-	hypothetical protein	
1424	3473	NEOS13_1401	peg	3473_5562_6200	5562	6200	+	Ribosomal subunit interface protein	
1425	3473	NEOS13_1402	peg	3473_6260_6718	6260	6718	+	hypothetical protein	
1426	3473	NEOS13_1403	peg	3473_6778_9660	6778	9660	+	hypothetical protein	
1427	3473	NEOS13_1404	peg	3473_11240_9657	11240	9657	-	hypothetical protein	
1428	3473	NEOS13_1405	peg	3473_11432_11914	11432	11914	+	hypothetical protein	
1429	3473	NEOS13_1406	peg	3473_12020_12439	12020	12439	+	hypothetical protein	
1430	3473	NEOS13_1407	peg	3473_12478_15498	12478	15498	+	hypothetical protein	
1431	3473	NEOS13_1408	peg	3473_15669_17912	15669	17912	+	hypothetical protein	
1432	3473	NEOS13_1409	peg	3473_18434_18012	18434	18012	-	Mobile element protein	
1433	3474	NEOS13_1410	peg	3474_59_1270	59	1270	+	Leucine-rich repeat containing protein	Leucine-rich repeat
1434	3476	NEOS13_1411	peg	3476_464_195	464	195	-	hypothetical protein	
1435	3477	NEOS13_1412	peg	3477_333_986	333	986	+	hypothetical protein	
1436	3477	NEOS13_1413	peg	3477_1604_1026	1604	1026	-	Pyridoxine biosynthesis glutamine amidotransferase, glutaminase subunit (EC 2.4.2.-)	
1437	3477	NEOS13_1414	peg	3477_2510_1605	2510	1605	-	Pyridoxine biosynthesis glutamine amidotransferase, synthase subunit (EC 2.4.2.-)	
1438	3477	NEOS13_1415	peg	3477_3447_2698	3447	2698	-	DNA polymerase III subunit epsilon	
1439	3477	NEOS13_1416	peg	3477_4064_3444	4064	3444	-	GTP-binding protein EngB	
1440	3477	NEOS13_1417	peg	3477_4480_4061	4480	4061	-	ATPase YjeE, predicted to have essential role in cell wall biosynthesis	
1441	3477	NEOS13_1418	peg	3477_5205_4483	5205	4483	-	FIG00899449: hypothetical protein	
1442	3477	NEOS13_1419	peg	3477_5322_6401	5322	6401	+	Leucine dehydrogenase (EC 1.4.1.9)	
1443	3477	NEOS13_1420	peg	3477_6420_8186	6420	8186	+	Arginyl-tRNA synthetase (EC 6.1.1.19)	
1444	3477	NEOS13_1421	peg	3477_8530_8745	8530	8745	+	hypothetical protein	
1445	3478	NEOS13_1422	peg	3478_316_137	316	137	-	hypothetical protein	
1446	3478	NEOS13_1423	peg	3478_715_323	715	323	-	hypothetical protein	
1447	3478	NEOS13_1424	peg	3478_714_830	714	830	+	hypothetical protein	
1448	3478	NEOS13_1425	peg	3478_2092_1160	2092	1160	-	Mobile element protein	
1449	3479	NEOS13_1426	peg	3479_516_388	516	388	-	hypothetical protein	
1450	3479	NEOS13_1427	peg	3479_882_1100	882	1100	+	hypothetical protein	
1451	3479	NEOS13_1428	peg	3479_1975_1397	1975	1397	-	hypothetical protein	
1452	3479	NEOS13_1429	peg	3479_2319_2447	2319	2447	+	hypothetical protein	
1453	3479	NEOS13_1430	peg	3479_2673_3104	2673	3104	+	hypothetical protein	
1454	3479	NEOS13_1431	peg	3479_3712_4248	3712	4248	+	hypothetical protein	
1455	3479	NEOS13_1432	peg	3479_4445_4948	4445	4948	+	hypothetical protein	
1456	3479	NEOS13_1433	peg	3479_5028_5906	5028	5906	+	Uncharacterized protein conserved in bacteria, NMA0228-like	
1457	3479	NEOS13_1434	peg	3479_5903_6802	5903	6802	+	hypothetical protein	
1458	3479	NEOS13_1435	peg	3479_6786_8027	6786	8027	+	Ribosomal RNA small subunit methyltransferase B (EC 2.1.1.-)	
1459	3479	NEOS13_1436	peg	3479_8909_8244	8909	8244	-	hypothetical protein	
1460	3479	NEOS13_1437	peg	3479_9361_8918	9361	8918	-	hypothetical protein	
1461	3479	NEOS13_1438	peg	3479_14630_9621	14630	9621	-	hypothetical protein	

1462	3479	NEOS13_1439	peg	3479_14706_14828	14706	14828	+	hypothetical protein	
1463	3479	NEOS13_1440	peg	3479_15858_15244	15858	15244	-	hypothetical protein	
1464	3479	NEOS13_1441	peg	3479_16723_15908	16723	15908	-	hypothetical protein	
1465	3479	NEOS13_1442	peg	3479_16926_16723	16926	16723	-	hypothetical protein	
1466	3479	NEOS13_1443	peg	3479_17541_18368	17541	18368	+	Mobile element protein	
1467	3479	NEOS13_1444	peg	3479_19907_18852	19907	18852	-	Leucine-rich repeat containing protein	Leucine-rich repeat
1468	3480	NEOS13_1445	peg	3480_155_24	155	24	-	hypothetical protein	
1469	3480	NEOS13_1446	peg	3480_880_194	880	194	-	hypothetical protein	
1470	3480	NEOS13_1447	peg	3480_1410_925	1410	925	-	hypothetical protein	
1471	3481	NEOS13_1448	peg	3481_76_1242	76	1242	+	transposase	Transposase
1472	3483	NEOS13_1449	peg	3483_312_569	312	569	+	transposase	Transposase
1473	3483	NEOS13_1450	peg	3483_807_1601	807	1601	+	Putative deoxyribonuclease YcfH	
1474	3483	NEOS13_1451	peg	3483_1669_2529	1669	2529	+	hypothetical protein	
1475	3483	NEOS13_1452	peg	3483_2791_2522	2791	2522	-	hypothetical protein	
1476	3483	NEOS13_1453	peg	3483_2841_3200	2841	3200	+	hypothetical protein	
1477	3483	NEOS13_1454	peg	3483_3206_4117	3206	4117	+	Phosphatidylserine decarboxylase (EC 4.1.1.65)	
1478	3483	NEOS13_1455	peg	3483_4134_4283	4134	4283	+	hypothetical protein	
1479	3483	NEOS13_1456	peg	3483_4705_7800	4705	7800	+	Protein export cytoplasm protein SecA ATPase RNA helicase (TC 3.A.5.1.1)	Sec_T2SS
1480	3483	NEOS13_1457	peg	3483_7990_8130	7990	8130	+	hypothetical protein	
1481	3483	NEOS13_1458	peg	3483_8651_8199	8651	8199	-	hypothetical protein	
1482	3483	NEOS13_1459	peg	3483_8745_9440	8745	9440	+	hypothetical protein	
1483	3483	NEOS13_1460	peg	3483_9573_9695	9573	9695	+	hypothetical protein	
1484	3483	NEOS13_1461	peg	3483_9850_9966	9850	9966	+	hypothetical protein	
1485	3483	NEOS13_1462	peg	3483_10196_10402	10196	10402	+	hypothetical protein	
1486	3483	NEOS13_1463	peg	3483_10494_10733	10494	10733	+	hypothetical protein	
1487	3483	NEOS13_1464	peg	3483_10807_11787	10807	11787	+	Lipoate synthase	
1488	3483	NEOS13_1465	peg	3483_11784_12710	11784	12710	+	hypothetical protein	
1489	3483	NEOS13_1466	peg	3483_12834_13880	12834	13880	+	Leucine-rich repeat containing protein	Leucine-rich repeat
1490	3483	NEOS13_1467	peg	3483_14163_15578	14163	15578	+	Leucine-rich repeat containing protein	Leucine-rich repeat
1491	3483	NEOS13_1468	peg	3483_15627_16337	15627	16337	+	Leucine-rich repeat containing protein	Leucine-rich repeat
1492	3483	NEOS13_1469	peg	3483_16632_18392	16632	18392	+	Leucine-rich repeat containing protein	Leucine-rich repeat
1493	3483	NEOS13_1470	peg	3483_18672_20570	18672	20570	+	Leucine-rich repeat containing protein	Leucine-rich repeat
1494	3483	NEOS13_1471	peg	3483_20866_22650	20866	22650	+	Leucine-rich repeat containing protein	Leucine-rich repeat
1495	3483	NEOS13_1472	peg	3483_22672_22833	22672	22833	+	Leucine-rich repeat containing protein	Leucine-rich repeat
1496	3483	NEOS13_1473	peg	3483_22954_22823	22954	22823	-	hypothetical protein	
1497	3483	NEOS13_1474	peg	3483_24308_22935	24308	22935	-	similar to transport protein	
1498	3483	NEOS13_1475	peg	3483_24669_24532	24669	24532	-	hypothetical protein	
1499	3483	NEOS13_1476	peg	3483_24854_24738	24854	24738	-	hypothetical protein	
1500	3483	NEOS13_1477	peg	3483_24931_25923	24931	25923	+	Type III secretion protein SctJ	T3SA2
1501	3483	NEOS13_1478	peg	3483_25935_26798	25935	26798	+	hypothetical protein	T3SA2
1502	3483	NEOS13_1479	peg	3483_26795_27451	26795	27451	+	Type III secretion translocase SctL	T3SA2
1503	3483	NEOS13_1480	peg	3483_27444_28331	27444	28331	+	Type III secretion inner membrane protein SctR	T3SA2
1504	3483	NEOS13_1481	peg	3483_28342_28623	28342	28623	+	probable type III secretion inner membrane protein SctS	T3SA2
1505	3483	NEOS13_1482	peg	3483_28721_29605	28721	29605	+	Type III secretion inner membrane protein SctT	T3SA2
1506	3483	NEOS13_1483	peg	3483_31242_30448	31242	30448	-	Leucine-rich repeat containing protein	Leucine-rich repeat
1507	3484	NEOS13_1484	peg	3484_560_375	560	375	-	hypothetical protein	
1508	3484	NEOS13_1485	peg	3484_2118_631	2118	631	-	hypothetical protein	
1509	3484	NEOS13_1486	peg	3484_2470_2303	2470	2303	-	hypothetical protein	
1510	3484	NEOS13_1487	peg	3484_2536_3036	2536	3036	+	ADP-heptose synthase (EC 2.7.-.-) / D-glycero-beta-D-manno-heptose 7-phosphate kinase	
1511	3484	NEOS13_1488	peg	3484_3048_4226	3048	4226	+	putative glpG protein	
1512	3484	NEOS13_1489	peg	3484_4293_5150	4293	5150	+	Ribonuclease Z (EC 3.1.26.11)	
1513	3484	NEOS13_1490	peg	3484_5162_6157	5162	6157	+	Tyrosine recombinase XerC	
1514	3484	NEOS13_1491	peg	3484_6409_8004	6409	8004	+	ABC transporter ATP-binding protein uup	ABC_transporter
1515	3484	NEOS13_1492	peg	3484_8021_8773	8021	8773	+	putative tRNA/rRNA methyltransferase(EC:2.1.1.-)	
1516	3484	NEOS13_1493	peg	3484_8754_9458	8754	9458	+	GTP cyclohydrolase I (EC 3.5.4.16) type 1	
1517	3484	NEOS13_1494	peg	3484_11104_10331	11104	10331	-	FIG137478: Hypothetical protein	
1518	3484	NEOS13_1495	peg	3484_11648_13132	11648	13132	+	Serine hydroxymethyltransferase (EC 2.1.2.1)	
1519	3484	NEOS13_1496	peg	3484_13190_13801	13190	13801	+	ATP-dependent Clp protease proteolytic subunit (EC 3.4.21.92)	
1520	3484	NEOS13_1497	peg	3484_13951_13838	13951	13838	-	hypothetical protein	
1521	3484	NEOS13_1498	peg	3484_13905_14714	13905	14714	+	Diaminopimelate epimerase (EC 5.1.1.7)	
1522	3484	NEOS13_1499	peg	3484_15648_14677	15648	14677	-	histone deacetylase superfamily	
1523	3484	NEOS13_1500	peg	3484_18519_16330	18519	16330	-	hypothetical protein	
1524	3484	NEOS13_1501	peg	3484_19610_19735	19610	19735	+	hypothetical protein	
1525	3484	NEOS13_1502	peg	3484_20069_20194	20069	20194	+	hypothetical protein	
1526	3484	NEOS13_1503	peg	3484_20302_20418	20302	20418	+	hypothetical protein	
1527	3484	NEOS13_1504	peg	3484_20595_20753	20595	20753	+	hypothetical protein	
1528	3484	NEOS13_0024ma	rna	3484_21686_21604	21686	21604	-	tRNA-Leu-TAA	RNA
1529	3484	NEOS13_1505	peg	3484_22716_21748	22716	21748	-	hypothetical protein	
1530	3484	NEOS13_1506	peg	3484_23815_22805	23815	22805	-	NAD-dependent glyceraldehyde-3-phosphate dehydrogenase (EC 1.2.1.12)	Glycolysis_Glucogenesis
1531	3484	NEOS13_1507	peg	3484_24057_26183	24057	26183	+	hypothetical protein	
1532	3487	NEOS13_1508	peg	3487_70_1125	70	1125	+	Leucine-rich repeat containing protein	Leucine-rich repeat
1533	3488	NEOS13_1509	peg	3488_22_402	22	402	+	Leucine-rich repeat containing protein	Leucine-rich repeat
1534	3489	NEOS13_1510	peg	3489_797_675	797	675	-	hypothetical protein	

1535	3489	NEOS13_1511	peg	3489_1233_3203	1233	3203	+	hypothetical protein	
1536	3489	NEOS13_1512	peg	3489_3658_3209	3658	3209	-	hypothetical protein	
1537	3490	NEOS13_1513	peg	3490_33_302	33	302	+	hypothetical protein	
1538	3491	NEOS13_1514	peg	3491_115_246	115	246	+	hypothetical protein	
1539	3491	NEOS13_1515	peg	3491_1342_683	1342	683	-	Queuosine Biosynthesis QueC ATPase	
1540	3491	NEOS13_1516	peg	3491_1992_1345	1992	1345	-	Queuosine Biosynthesis QueE Radical SAM	
1541	3491	NEOS13_1517	peg	3491_2303_4144	2303	4144	+	ankyrin repeat protein	Ankyrin
1542	3491	NEOS13_1518	peg	3491_4212_4325	4212	4325	+	hypothetical protein	
1543	3491	NEOS13_1519	peg	3491_4327_4440	4327	4440	+	hypothetical protein	
1544	3491	NEOS13_1520	peg	3491_4533_5543	4533	5543	+	Malate dehydrogenase (EC 1.1.1.37)	TCA
1545	3491	NEOS13_1521	peg	3491_5802_6629	5802	6629	+	hypothetical protein	
1546	3491	NEOS13_1522	peg	3491_7802_6786	7802	6786	-	hypothetical protein	
1547	3491	NEOS13_1523	peg	3491_8019_8204	8019	8204	+	hypothetical protein	
1548	3491	NEOS13_1524	peg	3491_8201_9007	8201	9007	+	Metal-dependent hydrolases of the beta-lactamase superfamily I; PhnP protein	
1549	3491	NEOS13_1525	peg	3491_9019_10344	9019	10344	+	GTP-binding protein HflX	
1550	3491	NEOS13_1526	peg	3491_12112_10634	12112	10634	-	Cytosol aminopeptidase PepA (EC 3.4.11.1)	
1551	3491	NEOS13_1527	peg	3491_12651_12133	12651	12133	-	Single-stranded DNA-binding protein	
1552	3491	NEOS13_1528	peg	3491_12863_12708	12863	12708	-	hypothetical protein	
1553	3491	NEOS13_1529	peg	3491_13597_13076	13597	13076	-	FIG00493912: hypothetical protein	
1554	3491	NEOS13_1530	peg	3491_14109_16154	14109	16154	+	Glycogen debranching enzyme (EC 3.2.1.-)	
1555	3491	NEOS13_1531	peg	3491_16814_16683	16814	16683	-	hypothetical protein	
1556	3491	NEOS13_1532	peg	3491_17049_19325	17049	19325	+	hypothetical protein	
1557	3491	NEOS13_1533	peg	3491_21274_19661	21274	19661	-	DNA repair protein RecN	
1558	3491	NEOS13_1534	peg	3491_21376_21537	21376	21537	+	hypothetical protein	
1559	3491	NEOS13_1535	peg	3491_22633_21719	22633	21719	-	Ribonuclease HIII (EC 3.1.26.4)	
1560	3491	NEOS13_1536	peg	3491_22735_23145	22735	23145	+	FIG00899448: hypothetical protein	
1561	3491	NEOS13_1537	peg	3491_23290_23174	23290	23174	-	hypothetical protein	
1562	3491	NEOS13_1538	peg	3491_23436_24380	23436	24380	+	hypothetical protein	
1563	3491	NEOS13_1539	peg	3491_25251_24637	25251	24637	-	tRNA pseudouridine synthase A (EC 4.2.1.70)	
1564	3491	NEOS13_1540	peg	3491_25383_25264	25383	25264	-	hypothetical protein	
1565	3491	NEOS13_1541	peg	3491_26010_25411	26010	25411	-	FIG00899512: hypothetical protein	
1566	3491	NEOS13_1542	peg	3491_26620_26021	26620	26021	-	FIG00899458: hypothetical protein	
1567	3491	NEOS13_1543	peg	3491_28662_27241	28662	27241	-	hypothetical protein	
1568	3491	NEOS13_1544	peg	3491_28791_28973	28791	28973	+	hypothetical protein	
1569	3491	NEOS13_1545	peg	3491_29932_29051	29932	29051	-	hypothetical protein	
1570	3491	NEOS13_1546	peg	3491_31369_30338	31369	30338	-	hypothetical protein	
1571	3491	NEOS13_1547	peg	3491_32556_31396	32556	31396	-	hypothetical protein	
1572	3491	NEOS13_1548	peg	3491_33122_32571	33122	32571	-	hypothetical protein	
1573	3491	NEOS13_1549	peg	3491_33604_34962	33604	34962	+	hypothetical protein	
1574	3491	NEOS13_1550	peg	3491_34972_35973	34972	35973	+	Holliday junction DNA helicase RuvB	
1575	3491	NEOS13_1551	peg	3491_36168_35956	36168	35956	-	hypothetical protein	
1576	3491	NEOS13_1552	peg	3491_36143_36826	36143	36826	+	hypothetical protein	
1577	3491	NEOS13_1553	peg	3491_37080_38117	37080	38117	+	S-adenosylmethionine:tRNA ribosyltransferase-isomerase (EC 5.-.-.-)	
1578	3491	NEOS13_1554	peg	3491_38657_40723	38657	40723	+	hypothetical protein	
1579	3491	NEOS13_1555	peg	3491_40736_40861	40736	40861	+	hypothetical protein	
1580	3491	NEOS13_1556	peg	3491_41058_42740	41058	42740	+	Leucine-rich repeat containing protein	Leucine-rich repeat
1581	3491	NEOS13_1557	peg	3491_43203_44732	43203	44732	+	Thermostable carboxypeptidase 1 (EC 3.4.17.19)	
1582	3491	NEOS13_1558	peg	3491_44933_46399	44933	46399	+	Leucine-rich repeat containing protein	Leucine-rich repeat
1583	3491	NEOS13_0025rna	rna	3491_46787_46873	46787	46873	+	tRNA-Ser-TGA	RNA
1584	3491	NEOS13_1559	peg	3491_48259_47477	48259	47477	-	hypothetical protein	
1585	3492	NEOS13_1560	peg	3492_115_246	115	246	+	hypothetical protein	
1586	3492	NEOS13_1561	peg	3492_1342_683	1342	683	-	Queuosine Biosynthesis QueC ATPase	
1587	3492	NEOS13_1562	peg	3492_1992_1345	1992	1345	-	Queuosine Biosynthesis QueE Radical SAM	
1588	3492	NEOS13_1563	peg	3492_2303_4144	2303	4144	+	ankyrin repeat protein	Ankyrin
1589	3492	NEOS13_1564	peg	3492_4212_4325	4212	4325	+	hypothetical protein	
1590	3492	NEOS13_1565	peg	3492_4327_4440	4327	4440	+	hypothetical protein	
1591	3492	NEOS13_1566	peg	3492_4533_5543	4533	5543	+	Malate dehydrogenase (EC 1.1.1.37)	TCA
1592	3492	NEOS13_1567	peg	3492_5802_6629	5802	6629	+	hypothetical protein	
1593	3492	NEOS13_1568	peg	3492_7802_6786	7802	6786	-	hypothetical protein	
1594	3492	NEOS13_1569	peg	3492_8019_8204	8019	8204	+	hypothetical protein	
1595	3492	NEOS13_1570	peg	3492_8201_9007	8201	9007	+	Metal-dependent hydrolases of the beta-lactamase superfamily I; PhnP protein	
1596	3492	NEOS13_1571	peg	3492_9019_10344	9019	10344	+	GTP-binding protein HflX	
1597	3492	NEOS13_1572	peg	3492_12112_10634	12112	10634	-	Cytosol aminopeptidase PepA (EC 3.4.11.1)	
1598	3492	NEOS13_1573	peg	3492_12651_12133	12651	12133	-	Single-stranded DNA-binding protein	
1599	3492	NEOS13_1574	peg	3492_12863_12708	12863	12708	-	hypothetical protein	
1600	3492	NEOS13_1575	peg	3492_13597_13076	13597	13076	-	FIG00493912: hypothetical protein	
1601	3492	NEOS13_1576	peg	3492_14109_16154	14109	16154	+	Glycogen debranching enzyme (EC 3.2.1.-)	
1602	3492	NEOS13_1577	peg	3492_16814_16683	16814	16683	-	hypothetical protein	
1603	3492	NEOS13_1578	peg	3492_17049_19325	17049	19325	+	hypothetical protein	
1604	3492	NEOS13_1579	peg	3492_21274_19661	21274	19661	-	DNA repair protein RecN	
1605	3492	NEOS13_1580	peg	3492_21376_21537	21376	21537	+	hypothetical protein	
1606	3492	NEOS13_1581	peg	3492_22633_21719	22633	21719	-	Ribonuclease HIII (EC 3.1.26.4)	
1607	3492	NEOS13_1582	peg	3492_22735_23145	22735	23145	+	FIG00899448: hypothetical protein	

1608	3492	NEOS13_1583	peg	3492_23290_23174	23290	23174	-	hypothetical protein	
1609	3492	NEOS13_1584	peg	3492_23436_24380	23436	24380	+	hypothetical protein	
1610	3492	NEOS13_1585	peg	3492_25251_24637	25251	24637	-	tRNA pseudouridine synthase A (EC 4.2.1.70)	
1611	3492	NEOS13_1586	peg	3492_25383_25264	25383	25264	-	hypothetical protein	
1612	3492	NEOS13_1587	peg	3492_26010_25411	26010	25411	-	FIG00899512: hypothetical protein	
1613	3492	NEOS13_1588	peg	3492_26620_26021	26620	26021	-	FIG00899458: hypothetical protein	
1614	3492	NEOS13_1589	peg	3492_28662_27241	28662	27241	-	hypothetical protein	
1615	3492	NEOS13_1590	peg	3492_28791_28973	28791	28973	+	hypothetical protein	
1616	3492	NEOS13_1591	peg	3492_29932_29051	29932	29051	-	hypothetical protein	
1617	3492	NEOS13_1592	peg	3492_31369_30338	31369	30338	-	hypothetical protein	
1618	3492	NEOS13_1593	peg	3492_32556_31396	32556	31396	-	hypothetical protein	
1619	3492	NEOS13_1594	peg	3492_33122_32571	33122	32571	-	hypothetical protein	
1620	3492	NEOS13_1595	peg	3492_33604_34962	33604	34962	+	hypothetical protein	
1621	3492	NEOS13_1596	peg	3492_34972_35973	34972	35973	+	Holliday junction DNA helicase RuvB	
1622	3492	NEOS13_1597	peg	3492_36168_35956	36168	35956	-	hypothetical protein	
1623	3492	NEOS13_1598	peg	3492_36143_36826	36143	36826	+	hypothetical protein	
1624	3492	NEOS13_1599	peg	3492_37080_38117	37080	38117	+	S-adenosylmethionine:tRNA ribosyltransferase-isomerase (EC 5.-.-.-)	
1625	3492	NEOS13_1600	peg	3492_38657_40723	38657	40723	+	hypothetical protein	
1626	3492	NEOS13_1601	peg	3492_40736_40861	40736	40861	+	hypothetical protein	
1627	3492	NEOS13_1602	peg	3492_41058_42740	41058	42740	+	Leucine-rich repeat containing protein	Leucine-rich repeat
1628	3492	NEOS13_1603	peg	3492_43203_44732	43203	44732	+	Thermostable carboxypeptidase 1 (EC 3.4.17.19)	
1629	3492	NEOS13_1604	peg	3492_44933_46399	44933	46399	+	Leucine-rich repeat containing protein	Leucine-rich repeat
1630	3492	NEOS13_0026ma	rna	3492_46787_46873	46787	46873	+	tRNA-Ser-TGA	RNA
1631	3492	NEOS13_1605	peg	3492_48259_47477	48259	47477	-	hypothetical protein	
1632	3493	NEOS13_1606	peg	3493_1982_1038	1982	1038	-	hypothetical protein	
1633	3493	NEOS13_1607	peg	3493_2808_2215	2808	2215	-	hypothetical protein	
1634	3493	NEOS13_1608	peg	3493_3859_2864	3859	2864	-	hypothetical protein	
1635	3493	NEOS13_1609	peg	3493_4976_3888	4976	3888	-	hypothetical protein	
1636	3495	NEOS13_1610	peg	3495_399_623	399	623	+	transposase	Transposase
1637	3495	NEOS13_1611	peg	3495_843_3065	843	3065	+	hypothetical protein	
1638	3495	NEOS13_1612	peg	3495_3236_3421	3236	3421	+	hypothetical protein	
1639	3495	NEOS13_1613	peg	3495_4053_3610	4053	3610	-	hypothetical protein	
1640	3495	NEOS13_1614	peg	3495_5128_4067	5128	4067	-	2-keto-3-deoxy-D-arabino-heptulosonate-7-phosphate synthase I alpha (EC 2.5.1.54)	
1641	3495	NEOS13_1615	peg	3495_5320_6873	5320	6873	+	hypothetical protein	
1642	3495	NEOS13_1616	peg	3495_7045_8436	7045	8436	+	Sodium/glycine symporter GlyP	
1643	3495	NEOS13_1617	peg	3495_9408_9875	9408	9875	+	Sodium/glycine symporter GlyP	
1644	3496	NEOS13_1618	peg	3496_46_1302	46	1302	+	DNA polymerase III beta subunit (EC 2.7.7.7)	
1645	3497	NEOS13_1619	peg	3497_120_251	120	251	+	hypothetical protein	
1646	3497	NEOS13_1620	peg	3497_437_781	437	781	+	Uncharacterized protein y4pE/y4sA	
1647	3497	NEOS13_1621	peg	3497_1539_904	1539	904	-	Pyridoxamine 5'-phosphate oxidase (EC 1.4.3.5)	
1648	3497	NEOS13_1622	peg	3497_1657_2859	1657	2859	+	Phosphoglycerate kinase (EC 2.7.2.3)	Glycolysis_Glucogenesis
1649	3497	NEOS13_1623	peg	3497_3186_4784	3186	4784	+	ADP/ATP Translocase, NTT1	NTT
1650	3497	NEOS13_1624	peg	3497_4983_6551	4983	6551	+	ADP/ATP Translocase, NTT1	NTT
1651	3497	NEOS13_1625	peg	3497_6753_6890	6753	6890	+	hypothetical protein	
1652	3497	NEOS13_1626	peg	3497_7602_7189	7602	7189	-	CDP-alcohol phosphatidyltransferase	
1653	3497	NEOS13_1627	peg	3497_7592_7705	7592	7705	+	hypothetical protein	
1654	3497	NEOS13_1628	peg	3497_8471_9106	8471	9106	+	hypothetical protein	
1655	3497	NEOS13_1629	peg	3497_10341_9418	10341	9418	-	hypothetical protein	
1656	3497	NEOS13_1630	peg	3497_11905_10631	11905	10631	-	hypothetical protein	
1657	3497	NEOS13_1631	peg	3497_13514_12420	13514	12420	-	hypothetical protein	
1658	3497	NEOS13_1632	peg	3497_15069_13552	15069	13552	-	Glutamyl-tRNA synthetase (EC 6.1.1.17) @ Glutamyl-tRNA(Gln) synthetase (EC 6.1.1.24)	
1659	3497	NEOS13_1633	peg	3497_17232_15283	17232	15283	-	Peptidase, S41 family	
1660	3497	NEOS13_1634	peg	3497_17586_18014	17586	18014	+	CHLPS Euo Protein	
1661	3497	NEOS13_1635	peg	3497_21632_18657	21632	18657	-	metalloprotease, insulinase family	
1662	3497	NEOS13_1636	peg	3497_21908_22759	21908	22759	+	CDP-diacylglycerol--serine O-phosphatidyltransferase (EC 2.7.8.8)	
1663	3497	NEOS13_1637	peg	3497_23520_22993	23520	22993	-	SOS-response repressor and protease LexA (EC 3.4.21.88)	
1664	3497	NEOS13_1638	peg	3497_24283_23585	24283	23585	-	hypothetical protein	
1665	3497	NEOS13_1639	peg	3497_25214_26644	25214	26644	+	DNA recombination protein RmuC	
1666	3497	NEOS13_1640	peg	3497_28017_26752	28017	26752	-	hypothetical protein	
1667	3497	NEOS13_1641	peg	3497_28929_28225	28929	28225	-	hypothetical protein	
1668	3497	NEOS13_1642	peg	3497_29268_30566	29268	30566	+	Predicted ATPase related to phosphate starvation-inducible protein PhoH	
1669	3497	NEOS13_1643	peg	3497_30805_31425	30805	31425	+	putative exopolysaccharide synthesis protein	
1670	3497	NEOS13_1644	peg	3497_31933_32301	31933	32301	+	hypothetical protein	
1671	3497	NEOS13_1645	peg	3497_32508_32768	32508	32768	+	hypothetical protein	
1672	3497	NEOS13_1646	peg	3497_33883_32861	33883	32861	-	hypothetical protein	
1673	3497	NEOS13_1647	peg	3497_34488_33880	34488	33880	-	hypothetical protein	
1674	3497	NEOS13_1648	peg	3497_34771_35091	34771	35091	+	hypothetical protein	
1675	3497	NEOS13_1649	peg	3497_35407_35117	35407	35117	-	putative Mitomycin resistance protein mcrB	
1676	3497	NEOS13_1650	peg	3497_36092_35955	36092	35955	-	hypothetical protein	
1677	3497	NEOS13_1651	peg	3497_36334_39456	36334	39456	+	Isoleucyl-tRNA synthetase (EC 6.1.1.5)	
1678	3497	NEOS13_1652	peg	3497_40079_41038	40079	41038	+	Arabinose 5-phosphate isomerase (EC 5.3.1.13)	

1679	3497	NEOS13_1653	peg	3497_41817_41260	41817	41260	-	hypothetical protein	
1680	3497	NEOS13_1654	peg	3497_41896_43185	41896	43185	+	Na+/H+ antiporter NhaD type	
1681	3497	NEOS13_0027ma	rna	3497_44355_45893	44355	45893	+	Small Subunit Ribosomal RNA; ssuRNA; SSU rRNA	RNA
1682	3498	NEOS13_1655	peg	3498_2066_726	2066	726	-	Leucine-rich repeat containing protein	Leucine-rich repeat
1683	3498	NEOS13_1656	peg	3498_2291_2175	2291	2175	-	hypothetical protein	
1684	3498	NEOS13_1657	peg	3498_2454_2323	2454	2323	-	hypothetical protein	
1685	3498	NEOS13_1658	peg	3498_3058_2942	3058	2942	-	hypothetical protein	
1686	3498	NEOS13_1659	peg	3498_3200_3355	3200	3355	+	Mobile element protein	
1687	3498	NEOS13_1660	peg	3498_3572_3432	3572	3432	-	hypothetical protein	
1688	3498	NEOS13_1661	peg	3498_6032_4236	6032	4236	-	hypothetical protein	
1689	3498	NEOS13_1662	peg	3498_6403_6564	6403	6564	+	hypothetical protein	
1690	3498	NEOS13_1663	peg	3498_6816_10238	6816	10238	+	Exodeoxyribonuclease V gamma chain (EC 3.1.11.5)	
1691	3498	NEOS13_1664	peg	3498_10398_11219	10398	11219	+	hypothetical protein	
1692	3498	NEOS13_1665	peg	3498_12358_15864	12358	15864	+	Exodeoxyribonuclease V beta chain (EC 3.1.11.5)	
1693	3498	NEOS13_1666	peg	3498_15921_17669	15921	17669	+	Exodeoxyribonuclease V alpha chain (EC 3.1.11.5)	
1694	3498	NEOS13_1667	peg	3498_18503_18114	18503	18114	-	hypothetical protein	
1695	3498	NEOS13_1668	peg	3498_19750_18581	19750	18581	-	hypothetical protein	
1696	3498	NEOS13_1669	peg	3498_21097_20612	21097	20612	-	hypothetical protein	
1697	3498	NEOS13_1670	peg	3498_21255_21392	21255	21392	+	hypothetical protein	
1698	3498	NEOS13_1671	peg	3498_21409_23574	21409	23574	+	Transcription elongation factor GreA	
1699	3498	NEOS13_1672	peg	3498_23575_24183	23575	24183	+	Nucleoside 5-triphosphatase RdgB (dHAPTP, dITP, XTP-specific) (EC 3.6.1.15)	
1700	3498	NEOS13_1673	peg	3498_24192_24887	24192	24887	+	hypothetical protein	
1701	3498	NEOS13_1674	peg	3498_25097_26080	25097	26080	+	hypothetical protein	
1702	3498	NEOS13_0028ma	rna	3498_26225_26297	26225	26297	+	tRNA-Ala-GGC	RNA
1703	3498	NEOS13_1675	peg	3498_26377_26526	26377	26526	+	hypothetical protein	
1704	3498	NEOS13_1676	peg	3498_27001_26768	27001	26768	-	hypothetical protein	
1705	3498	NEOS13_1677	peg	3498_27870_27142	27870	27142	-	hypothetical protein	
1706	3498	NEOS13_1678	peg	3498_28664_27933	28664	27933	-	hypothetical protein	
1707	3498	NEOS13_1679	peg	3498_28664_28780	28664	28780	+	hypothetical protein	
1708	3498	NEOS13_1680	peg	3498_30024_29065	30024	29065	-	hypothetical protein	
1709	3498	NEOS13_1681	peg	3498_31885_30443	31885	30443	-	hypothetical protein	Outer_membrane_protein
1710	3498	NEOS13_1682	peg	3498_35714_32775	35714	32775	-	hypothetical protein	
1711	3498	NEOS13_1683	peg	3498_36733_35912	36733	35912	-	hypothetical protein	
1712	3498	NEOS13_1684	peg	3498_37056_38993	37056	38993	+	Threonyl-tRNA synthetase (EC 6.1.1.3)	
1713	3498	NEOS13_1685	peg	3498_38981_39733	38981	39733	+	Septum site-determining protein MinD	
1714	3498	NEOS13_1686	peg	3498_39870_40616	39870	40616	+	Virulence plasmid protein pGP6-D	
1715	3498	NEOS13_1687	peg	3498_41205_41089	41205	41089	-	hypothetical protein	
1716	3498	NEOS13_1688	peg	3498_41253_41399	41253	41399	+	hypothetical protein	
1717	3498	NEOS13_1689	peg	3498_41528_42235	41528	42235	+	hypothetical protein	
1718	3499	NEOS13_1690	peg	3499_2347_305	2347	305	-	Leucine-rich repeat containing protein	Leucine-rich repeat
1719	3500	NEOS13_1691	peg	3500_424_245	424	245	-	hypothetical protein	
1720	3500	NEOS13_1692	peg	3500_823_431	823	431	-	transposase, IS4 family	Transposase
1721	3500	NEOS13_1693	peg	3500_822_938	822	938	+	hypothetical protein	
1722	3500	NEOS13_1694	peg	3500_2200_1268	2200	1268	-	Mobile element protein	
1723	3501	NEOS13_1695	peg	3501_1651_503	1651	503	-	Leucine-rich repeat containing protein	Leucine-rich repeat
1724	3502	NEOS13_1696	peg	3502_1656_370	1656	370	-	Leucine-rich repeat containing protein	Leucine-rich repeat
1725	3504	NEOS13_1697	peg	3504_20_202	20	202	+	hypothetical protein	
1726	3504	NEOS13_1698	peg	3504_637_524	637	524	-	hypothetical protein	
1727	3504	NEOS13_1699	peg	3504_1990_1133	1990	1133	-	AMP nucleosidase (EC 3.2.2.4)	
1728	3504	NEOS13_1700	peg	3504_3635_2406	3635	2406	-	hypothetical protein	
1729	3504	NEOS13_1701	peg	3504_3877_5481	3877	5481	+	Peptide chain release factor 3	
1730	3504	NEOS13_1702	peg	3504_5563_5441	5563	5441	-	hypothetical protein	
1731	3504	NEOS13_1703	peg	3504_6191_5679	6191	5679	-	hypothetical protein	
1732	3504	NEOS13_1704	peg	3504_6264_7313	6264	7313	+	A/G-specific adenine glycosylase (EC 3.2.2.-)	
1733	3504	NEOS13_1705	peg	3504_7543_9459	7543	9459	+	Leucine-rich repeat containing protein	Leucine-rich repeat
1734	3504	NEOS13_1706	peg	3504_9469_9588	9469	9588	+	Leucine-rich repeat containing protein	Leucine-rich repeat
1735	3506	NEOS13_1707	peg	3506_56_202	56	202	+	hypothetical protein	
1736	3506	NEOS13_1708	peg	3506_962_825	962	825	-	hypothetical protein	
1737	3507	NEOS13_1709	peg	3507_736_452	736	452	-	hypothetical protein	
1738	3509	NEOS13_1710	peg	3509_427_287	427	287	-	hypothetical protein	
1739	3509	NEOS13_1711	peg	3509_570_1358	570	1358	+	hypothetical protein	
1740	3510	NEOS13_1712	peg	3510_84_617	84	617	+	hypothetical protein	
1741	3510	NEOS13_1713	peg	3510_658_1740	658	1740	+	hypothetical protein	
1742	3510	NEOS13_1714	peg	3510_2026_2370	2026	2370	+	Mobile element protein	
1743	3511	NEOS13_1715	peg	3511_45_1028	45	1028	+	Retron-type RNA-directed DNA polymerase (EC 2.7.7.49)	
1744	3511	NEOS13_1716	peg	3511_1150_2442	1150	2442	+	hypothetical protein	
1745	3511	NEOS13_1717	peg	3511_2698_2853	2698	2853	+	hypothetical protein	
1746	3511	NEOS13_1718	peg	3511_2864_3061	2864	3061	+	Mobile element protein	
1747	3511	NEOS13_1719	peg	3511_3058_3174	3058	3174	+	hypothetical protein	
1748	3511	NEOS13_1720	peg	3511_3181_3423	3181	3423	+	Mobile element protein	
1749	3512	NEOS13_1721	peg	3512_45_1028	45	1028	+	Retron-type RNA-directed DNA polymerase (EC 2.7.7.49)	
1750	3512	NEOS13_1722	peg	3512_3968_1224	3968	1224	-	Tetratricopeptide TPR_2 repeat protein	
1751	3512	NEOS13_1723	peg	3512_4562_8761	4562	8761	+	hypothetical protein	
1752	3512	NEOS13_1724	peg	3512_8779_8928	8779	8928	+	hypothetical protein	
1753	3512	NEOS13_1725	peg	3512_8930_9076	8930	9076	+	hypothetical protein	
1754	3512	NEOS13_1726	peg	3512_9395_9135	9395	9135	-	hypothetical protein	
1755	3512	NEOS13_1727	peg	3512_9748_9446	9748	9446	-	reverse transcriptase/retron type, probably fragment	

1756	3513	NEOS13_1728	peg	3513_45_1028	45	1028	+	Retron-type RNA-directed DNA polymerase (EC 2.7.7.49)	
1757	3513	NEOS13_1729	peg	3513_1348_5238	1348	5238	+	hypothetical protein	
1758	3514	NEOS13_1730	peg	3514_45_1028	45	1028	+	Retron-type RNA-directed DNA polymerase (EC 2.7.7.49)	
1759	3514	NEOS13_1731	peg	3514_1181_1294	1181	1294	+	hypothetical protein	
1760	3514	NEOS13_1732	peg	3514_1763_1647	1763	1647	-	hypothetical protein	
1761	3514	NEOS13_1733	peg	3514_1938_2366	1938	2366	+	hypothetical protein	
1762	3514	NEOS13_1734	peg	3514_2638_2486	2638	2486	-	hypothetical protein	
1763	3514	NEOS13_1735	peg	3514_3264_4058	3264	4058	+	hypothetical protein	
1764	3514	NEOS13_1736	peg	3514_4549_4409	4549	4409	-	hypothetical protein	
1765	3514	NEOS13_1737	peg	3514_5116_4550	5116	4550	-	hypothetical protein	
1766	3514	NEOS13_1738	peg	3514_5961_5650	5961	5650	-	hypothetical protein	
1767	3514	NEOS13_1739	peg	3514_7216_6602	7216	6602	-	Alkaline phosphatase like protein	
1768	3515	NEOS13_1740	peg	3515_45_1028	45	1028	+	Retron-type RNA-directed DNA polymerase (EC 2.7.7.49)	
1769	3515	NEOS13_1741	peg	3515_4116_1255	4116	1255	-	Valyl-tRNA synthetase (EC 6.1.1.9)	
1770	3515	NEOS13_1742	peg	3515_7747_4670	7747	4670	-	hypothetical protein	
1771	3515	NEOS13_1743	peg	3515_7967_8089	7967	8089	+	hypothetical protein	
1772	3515	NEOS13_1744	peg	3515_8357_8512	8357	8512	+	hypothetical protein	
1773	3515	NEOS13_1745	peg	3515_9230_8742	9230	8742	-	protein of unknown function DUF192	
1774	3515	NEOS13_1746	peg	3515_15057_9361	15057	9361	-	Excinuclease ABC subunit A, dimeric form	ABC_transporter
1775	3515	NEOS13_1747	peg	3515_15584_15453	15584	15453	-	hypothetical protein	
1776	3515	NEOS13_1748	peg	3515_15747_17516	15747	17516	+	Pyruvate kinase (EC 2.7.1.40)	Glycolysis_Glucogenesis
1777	3515	NEOS13_1749	peg	3515_20468_17883	20468	17883	-	DNA topoisomerase I (EC 5.99.1.2)	
1778	3515	NEOS13_1750	peg	3515_20811_20954	20811	20954	+	hypothetical protein	
1779	3515	NEOS13_1751	peg	3515_22059_20956	22059	20956	-	3-dehydroquinate synthase (EC 4.2.3.4)	
1780	3515	NEOS13_1752	peg	3515_23971_23156	23971	23156	-	hypothetical protein	
1781	3515	NEOS13_1753	peg	3515_24061_24882	24061	24882	+	Chromosome (plasmid) partitioning protein ParB / Stage 0 sporulation protein J	
1782	3515	NEOS13_1754	peg	3515_25392_25526	25392	25526	+	hypothetical protein	
1783	3515	NEOS13_1755	peg	3515_26253_27362	26253	27362	+	hypothetical protein	
1784	3515	NEOS13_1756	peg	3515_27561_27394	27561	27394	-	hypothetical protein	
1785	3515	NEOS13_1757	peg	3515_27843_28976	27843	28976	+	BatA (Bacteroides aerotolerance operon)	
1786	3515	NEOS13_1758	peg	3515_28973_31864	28973	31864	+	hypothetical protein	
1787	3515	NEOS13_1759	peg	3515_31858_33606	31858	33606	+	hypothetical protein	
1788	3515	NEOS13_1760	peg	3515_33599_34417	33599	34417	+	putative batE protein	
1789	3515	NEOS13_1761	peg	3515_34665_34552	34665	34552	-	hypothetical protein	
1790	3515	NEOS13_1762	peg	3515_34982_35962	34982	35962	+	hypothetical protein	
1791	3515	NEOS13_1763	peg	3515_36199_36059	36199	36059	-	hypothetical protein	
1792	3515	NEOS13_1764	peg	3515_36271_38283	36271	38283	+	Ribonucleotide reductase of class II (coenzyme B12-dependent) (EC 1.17.4.1)	
1793	3515	NEOS13_1765	peg	3515_39710_39045	39710	39045	-	hypothetical protein	
1794	3515	NEOS13_1766	peg	3515_41117_39732	41117	39732	-	hypothetical protein	
1795	3515	NEOS13_1767	peg	3515_41462_41632	41462	41632	+	hypothetical protein	
1796	3516	NEOS13_1768	peg	3516_45_1028	45	1028	+	Retron-type RNA-directed DNA polymerase (EC 2.7.7.49)	
1797	3516	NEOS13_1769	peg	3516_1956_4037	1956	4037	+	Leucine-rich repeat containing protein	Leucine-rich repeat
1798	3516	NEOS13_1770	peg	3516_4079_4237	4079	4237	+	hypothetical protein	
1799	3516	NEOS13_1771	peg	3516_7211_4530	7211	4530	-	Mg(2+) transport ATPase, P-type (EC 3.6.3.2)	
1800	3516	NEOS13_1772	peg	3516_9977_7455	9977	7455	-	hypothetical protein	
1801	3516	NEOS13_1773	peg	3516_11391_11224	11391	11224	-	hypothetical protein	
1802	3516	NEOS13_1774	peg	3516_11345_14116	11345	14116	+	Chaperone protein DnaK	
1803	3516	NEOS13_1775	peg	3516_16144_14276	16144	14276	-	putative sulfate transport protein	
1804	3516	NEOS13_1776	peg	3516_16260_17687	16260	17687	+	GTP-binding protein EngA	
1805	3516	NEOS13_1777	peg	3516_17920_18759	17920	18759	+	Ribosomal protein L11 methyltransferase (EC 2.1.1.-)	
1806	3516	NEOS13_1778	peg	3516_19433_19179	19433	19179	-	hypothetical protein	
1807	3516	NEOS13_1779	peg	3516_21175_19508	21175	19508	-	Heat shock protein 60 family chaperone GroEL	
1808	3516	NEOS13_1780	peg	3516_21505_21212	21505	21212	-	Heat shock protein 60 family co-chaperone GroES	
1809	3516	NEOS13_1781	peg	3516_22877_21618	22877	21618	-	alternate gene name: yzbB	
1810	3516	NEOS13_1782	peg	3516_23479_24912	23479	24912	+	hypothetical protein	
1811	3516	NEOS13_1783	peg	3516_25429_25202	25429	25202	-	Mobile element protein	
1812	3516	NEOS13_1784	peg	3516_25904_25698	25904	25698	-	Mobile element protein	
1813	3516	NEOS13_1785	peg	3516_26327_26202	26327	26202	-	hypothetical protein	
1814	3516	NEOS13_1786	peg	3516_27972_26779	27972	26779	-	Leucine-rich repeat containing protein	Leucine-rich repeat
1815	3517	NEOS13_1787	peg	3517_45_1028	45	1028	+	Retron-type RNA-directed DNA polymerase (EC 2.7.7.49)	
1816	3517	NEOS13_1788	peg	3517_2126_1332	2126	1332	-	5-nucleotidase SurE (EC 3.1.3.5)	
1817	3517	NEOS13_1789	peg	3517_2277_3299	2277	3299	+	hypothetical protein	
1818	3517	NEOS13_1790	peg	3517_5221_3368	5221	3368	-	hypothetical protein	
1819	3517	NEOS13_1791	peg	3517_6081_5227	6081	5227	-	hypothetical protein	
1820	3517	NEOS13_1792	peg	3517_7071_6325	7071	6325	-	tRNA(Cytosine32)-2-thiocytidine synthetase	
1821	3517	NEOS13_1793	peg	3517_7191_7445	7191	7445	+	hypothetical protein	
1822	3517	NEOS13_0029rna	rna	3517_7675_7747	7675	7747	+	tRNA-Thr-TGT	RNA
1823	3517	NEOS13_0030rna	rna	3517_7755_7836	7755	7836	+	tRNA-Tyr-GTA	RNA
1824	3517	NEOS13_1794	peg	3517_8922_8032	8922	8032	-	Cassette chromosome recombinase B	
1825	3517	NEOS13_1795	peg	3517_9519_9220	9519	9220	-	hypothetical protein	

1826	3517	NEOS13_1796	peg	3517_9824_9651	9824	9651	-	hypothetical protein	
1827	3517	NEOS13_1797	peg	3517_11935_10715	11935	10715	-	putative Na(+)/H(+) antiporter	
1828	3517	NEOS13_1798	peg	3517_12692_12808	12692	12808	+	hypothetical protein	
1829	3517	NEOS13_1799	peg	3517_12820_12939	12820	12939	+	hypothetical protein	
1830	3517	NEOS13_1800	peg	3517_13164_13592	13164	13592	+	heat shock protein Hsp20	
1831	3517	NEOS13_1801	peg	3517_14266_13928	14266	13928	-	hypothetical protein	
1832	3517	NEOS13_1802	peg	3517_14781_14656	14781	14656	-	hypothetical protein	
1833	3517	NEOS13_1803	peg	3517_14827_16203	14827	16203	+	Mg/Co/Ni transporter MgtE / CBS domain	
1834	3517	NEOS13_1804	peg	3517_16791_17183	16791	17183	+	putative hyperosmotically inducible periplasmic protein	
1835	3517	NEOS13_1805	peg	3517_17199_17786	17199	17786	+	hypothetical protein	
1836	3517	NEOS13_1806	peg	3517_18207_19196	18207	19196	+	hypothetical protein	
1837	3517	NEOS13_1807	peg	3517_19753_19337	19753	19337	-	hypothetical protein	
1838	3517	NEOS13_1808	peg	3517_20169_19750	20169	19750	-	hypothetical protein	
1839	3517	NEOS13_1809	peg	3517_20712_20599	20712	20599	-	hypothetical protein	
1840	3517	NEOS13_1810	peg	3517_21389_20709	21389	20709	-	Probable O-methyltransferase	
1841	3517	NEOS13_1811	peg	3517_21494_21853	21494	21853	+	putative arsenate reductase(EC:1.-)	
1842	3517	NEOS13_1812	peg	3517_22197_22979	22197	22979	+	hypothetical protein	
1843	3517	NEOS13_1813	peg	3517_23251_23394	23251	23394	+	hypothetical protein	
1844	3517	NEOS13_1814	peg	3517_24256_24122	24256	24122	-	hypothetical protein	
1845	3517	NEOS13_1815	peg	3517_24629_25138	24629	25138	+	hypothetical protein	
1846	3517	NEOS13_1816	peg	3517_26235_25237	26235	25237	-	hypothetical protein	
1847	3518	NEOS13_1817	peg	3518_45_1028	45	1028	+	Retron-type RNA-directed DNA polymerase (EC 2.7.7.49)	
1848	3518	NEOS13_1818	peg	3518_1121_1282	1121	1282	+	hypothetical protein	
1849	3518	NEOS13_1819	peg	3518_1385_2095	1385	2095	+	hypothetical protein	
1850	3518	NEOS13_1820	peg	3518_2844_2635	2844	2635	-	hypothetical protein	
1851	3518	NEOS13_1821	peg	3518_2945_3244	2945	3244	+	HigA protein (antitoxin to HigB)	
1852	3518	NEOS13_1822	peg	3518_3680_3871	3680	3871	+	hypothetical protein	
1853	3519	NEOS13_1823	peg	3519_1026_2102	1026	2102	+	Branched-chain alpha-keto acid dehydrogenase, E1 component, alpha subunit (EC 1.2.4.4)	
1854	3519	NEOS13_1824	peg	3519_2132_3109	2132	3109	+	Branched-chain alpha-keto acid dehydrogenase, E1 component, beta subunit (EC 1.2.4.4)	Glycolysis_Glucogenesis
1855	3519	NEOS13_1825	peg	3519_3122_4348	3122	4348	+	Dihydrolipoamide acyltransferase component of branched-chain alpha-keto acid dehydrogenase complex (EC 2.3.1.168)	Glycolysis_Glucogenesis
1856	3519	NEOS13_1826	peg	3519_4311_4448	4311	4448	+	hypothetical protein	
1857	3519	NEOS13_1827	peg	3519_4602_6293	4602	6293	+	Potassium-transporting ATPase A chain (EC 3.6.3.12) (TC 3.A.3.7.1)	
1858	3519	NEOS13_1828	peg	3519_6364_8460	6364	8460	+	Potassium-transporting ATPase B chain (EC 3.6.3.12) (TC 3.A.3.7.1)	
1859	3519	NEOS13_1829	peg	3519_8498_9058	8498	9058	+	Potassium-transporting ATPase C chain (EC 3.6.3.12) (TC 3.A.3.7.1)	
1860	3519	NEOS13_1830	peg	3519_10665_12377	10665	12377	+	hypothetical protein	
1861	3519	NEOS13_1831	peg	3519_12843_14420	12843	14420	+	Leucine-rich repeat containing protein	Leucine-rich repeat
1862	3519	NEOS13_1832	peg	3519_15173_15048	15173	15048	-	hypothetical protein	
1863	3519	NEOS13_1833	peg	3519_15490_15371	15490	15371	-	hypothetical protein	
1864	3519	NEOS13_1834	peg	3519_15902_15678	15902	15678	-	hypothetical protein	
1865	3519	NEOS13_1835	peg	3519_16222_17631	16222	17631	+	hypothetical protein	
1866	3519	NEOS13_1836	peg	3519_19189_17903	19189	17903	-	RNA methyltransferase, TrmA family	
1867	3519	NEOS13_1837	peg	3519_20939_19182	20939	19182	-	Single-stranded-DNA-specific exonuclease RecJ (EC 3.1.-.-)	
1868	3519	NEOS13_1838	peg	3519_26072_21498	26072	21498	-	Protein-export membrane protein SecD (TC 3.A.5.1.1) / Protein-export membrane protein SecF (TC 3.A.5.1.1)	
1869	3519	NEOS13_1839	peg	3519_28005_26452	28005	26452	-	ADP/ATP Translocase, NTT1	NTT
1870	3519	NEOS13_1840	peg	3519_29197_28682	29197	28682	-	FIG00493911: hypothetical protein	
1871	3519	NEOS13_1841	peg	3519_29550_30665	29550	30665	+	hypothetical protein	
1872	3519	NEOS13_1842	peg	3519_30662_31654	30662	31654	+	Manganese ABC transporter, periplasmic-binding protein SitA	ABC_transporter
1873	3519	NEOS13_1843	peg	3519_31651_32439	31651	32439	+	Manganese ABC transporter, ATP-binding protein SitB	ABC_transporter
1874	3519	NEOS13_1844	peg	3519_32444_33796	32444	33796	+	Manganese ABC transporter, inner membrane permease protein SitC	ABC_transporter
1875	3519	NEOS13_1845	peg	3519_33793_34803	33793	34803	+	Manganese ABC transporter, inner membrane permease protein SitD	ABC_transporter
1876	3519	NEOS13_1846	peg	3519_35587_34817	35587	34817	-	hypothetical protein	
1877	3519	NEOS13_1847	peg	3519_36258_36139	36258	36139	-	hypothetical protein	
1878	3520	NEOS13_0031ma	rna	3520_311_3338	311	3338	+	Large Subunit Ribosomal RNA; lsuRNA; LSU rRNA	RNA
1879	3520	NEOS13_1848	peg	3520_3643_3765	3643	3765	+	hypothetical protein	
1880	3520	NEOS13_1849	peg	3520_5773_3740	5773	3740	-	hypothetical protein	
1881	3520	NEOS13_1850	peg	3520_6047_7234	6047	7234	+	hypothetical protein	
1882	3520	NEOS13_1851	peg	3520_7954_7256	7954	7256	-	Ribosomal RNA small subunit methyltransferase E (EC 2.1.1.-)	
1883	3520	NEOS13_1852	peg	3520_9060_7957	9060	7957	-	FIG00493852: hypothetical protein	
1884	3520	NEOS13_1853	peg	3520_9902_9195	9902	9195	-	Hypothetical protein YbbP, contains nucleotide-binding domain of DisA bacterial checkpoint controller	
1885	3520	NEOS13_1854	peg	3520_10783_10007	10783	10007	-	Dihydropteroate synthase (EC 2.5.1.15)	
1886	3520	NEOS13_1855	peg	3520_10913_12316	10913	12316	+	Cytochrome d ubiquinol oxidase subunit I (EC 1.10.3.-)	Oxidative_Phosphorylation
1887	3520	NEOS13_1856	peg	3520_12313_13344	12313	13344	+	Cytochrome d ubiquinol oxidase subunit II (EC 1.10.3.-)	Oxidative_Phosphorylation

1888	3520	NEOS13_1857	peg	3520_14584_13358	14584	13358	-	Dihydrofolate synthase (EC 6.3.2.12) / Folylpolyglutamate synthase (EC 6.3.2.17)	
1889	3520	NEOS13_1858	peg	3520_15438_14584	15438	14584	-	COG0613, Predicted metal-dependent phosphoesterases (PHP family)	
1890	3520	NEOS13_1859	peg	3520_16325_15435	16325	15435	-	UDP-N-acetylenolpyruvoylglucosamine reductase (EC 1.1.1.158)	
1891	3520	NEOS13_1860	peg	3520_16842_16330	16842	16330	-	Transcription termination protein NusB	
1892	3520	NEOS13_0032rna	rna	3520_17271_17344	17271	17344	+	tRNA-Met-CAT	RNA
1893	3520	NEOS13_1861	peg	3520_17611_17471	17611	17471	-	hypothetical protein	
1894	3520	NEOS13_1862	peg	3520_17929_17621	17929	17621	-	hypothetical protein	
1895	3520	NEOS13_1863	peg	3520_18302_17913	18302	17913	-	hypothetical protein	
1896	3520	NEOS13_1864	peg	3520_18822_19094	18822	19094	+	hypothetical protein	
1897	3520	NEOS13_1865	peg	3520_19396_19548	19396	19548	+	hypothetical protein	
1898	3520	NEOS13_1866	peg	3520_20728_19787	20728	19787	-	putative MutT/nudix family protein	
1899	3520	NEOS13_1867	peg	3520_21027_20749	21027	20749	-	hypothetical protein	
1900	3520	NEOS13_1868	peg	3520_21682_21158	21682	21158	-	conserved protein	
1901	3520	NEOS13_1869	peg	3520_21876_22103	21876	22103	+	hypothetical protein	
1902	3520	NEOS13_1870	peg	3520_23204_22959	23204	22959	-	hypothetical protein	
1903	3520	NEOS13_1871	peg	3520_23658_23885	23658	23885	+	hypothetical protein	
1904	3520	NEOS13_1872	peg	3520_24862_24722	24862	24722	-	hypothetical protein	
1905	3520	NEOS13_1873	peg	3520_25130_25270	25130	25270	+	hypothetical protein	
1906	3520	NEOS13_1874	peg	3520_25837_27114	25837	27114	+	hypothetical protein	
1907	3520	NEOS13_1875	peg	3520_27675_29894	27675	29894	+	hypothetical protein	
1908	3520	NEOS13_1876	peg	3520_30506_30682	30506	30682	+	hypothetical protein	
1909	3520	NEOS13_1877	peg	3520_31000_30734	31000	30734	-	hypothetical protein	
1910	3520	NEOS13_1878	peg	3520_31234_31001	31234	31001	-	hypothetical protein	
1911	3520	NEOS13_1879	peg	3520_31468_31710	31468	31710	+	hypothetical protein	
1912	3520	NEOS13_1880	peg	3520_32214_32005	32214	32005	-	hypothetical protein	
1913	3520	NEOS13_1881	peg	3520_32362_34233	32362	34233	+	hypothetical protein	
1914	3520	NEOS13_1882	peg	3520_35439_34975	35439	34975	-	hypothetical protein	
1915	3520	NEOS13_1883	peg	3520_35605_35459	35605	35459	-	hypothetical protein	
1916	3520	NEOS13_1884	peg	3520_36071_36190	36071	36190	+	hypothetical protein	
1917	3520	NEOS13_1885	peg	3520_36408_36527	36408	36527	+	hypothetical protein	
1918	3520	NEOS13_1886	peg	3520_37055_37237	37055	37237	+	hypothetical protein	
1919	3520	NEOS13_1887	peg	3520_38807_37314	38807	37314	-	hypothetical protein	
1920	3520	NEOS13_1888	peg	3520_39058_39648	39058	39648	+	GCN5-related N-acetyltransferase	
1921	3520	NEOS13_1889	peg	3520_39938_40114	39938	40114	+	hypothetical protein	
1922	3520	NEOS13_1890	peg	3520_40372_40575	40372	40575	+	DUF378 domain-containing protein	
1923	3520	NEOS13_1891	peg	3520_41520_41071	41520	41071	-	Membrane-associated phospholipid phosphatase	
1924	3520	NEOS13_1892	peg	3520_42319_41834	42319	41834	-	hypothetical protein	
1925	3520	NEOS13_1893	peg	3520_42593_42730	42593	42730	+	hypothetical protein	
1926	3520	NEOS13_1894	peg	3520_42845_43288	42845	43288	+	hypothetical protein	
1927	3520	NEOS13_1895	peg	3520_43390_45432	43390	45432	+	Thymidylate kinase (EC 2.7.4.9)	
1928	3521	NEOS13_1896	peg	3521_879_995	879	995	+	hypothetical protein	
1929	3521	NEOS13_1897	peg	3521_1053_1469	1053	1469	+	hypothetical protein	
1930	3521	NEOS13_1898	peg	3521_1841_2413	1841	2413	+	hypothetical protein	
1931	3521	NEOS13_1899	peg	3521_2403_2657	2403	2657	+	hypothetical protein	
1932	3521	NEOS13_1900	peg	3521_2862_3035	2862	3035	+	hypothetical protein	
1933	3523	NEOS13_1901	peg	3523_1882_605	1882	605	-	Leucine-rich repeat containing protein	Leucine-rich repeat
1934	3523	NEOS13_1902	peg	3523_2213_2010	2213	2010	-	hypothetical protein	
1935	3523	NEOS13_1903	peg	3523_3509_2367	3509	2367	-	Leucine-rich repeat containing protein	Leucine-rich repeat
1936	3524	NEOS13_1904	peg	3524_1208_420	1208	420	-	hypothetical protein	
1937	3524	NEOS13_1905	peg	3524_1351_1491	1351	1491	+	hypothetical protein	
1938	3524	NEOS13_1906	peg	3524_1826_1584	1826	1584	-	hypothetical protein	
1939	3525	NEOS13_1907	peg	3525_55_927	55	927	+	hypothetical protein	
1940	3526	NEOS13_1908	peg	3526_40_168	40	168	+	hypothetical protein	
1941	3526	NEOS13_1909	peg	3526_283_726	283	726	+	transposase, IS4 family protein	Transposase
1942	3526	NEOS13_1910	peg	3526_1257_5834	1257	5834	+	hypothetical protein	
1943	3526	NEOS13_1911	peg	3526_5818_5931	5818	5931	+	hypothetical protein	
1944	3528	NEOS13_1912	peg	3528_393_172	393	172	-	Mobile element protein	
1945	3529	NEOS13_1913	peg	3529_76_240	76	240	+	hypothetical protein	
1946	3529	NEOS13_1914	peg	3529_557_1213	557	1213	+	Carbonic anhydrase (EC 4.2.1.1)	
1947	3529	NEOS13_1915	peg	3529_2932_1601	2932	1601	-	Enolase (EC 4.2.1.11)	Glycolysis_Glucogenesis
1948	3529	NEOS13_1916	peg	3529_4394_3240	4394	3240	-	Ribosome small subunit-stimulated GTPase EngC	
1949	3529	NEOS13_1917	peg	3529_5146_4391	5146	4391	-	hypothetical protein	
1950	3529	NEOS13_1918	peg	3529_6513_5446	6513	5446	-	hypothetical protein	
1951	3529	NEOS13_1919	peg	3529_7583_6720	7583	6720	-	hypothetical protein	
1952	3529	NEOS13_1920	peg	3529_8571_8446	8571	8446	-	hypothetical protein	
1953	3529	NEOS13_1921	peg	3529_8834_8995	8834	8995	+	hypothetical protein	
1954	3529	NEOS13_1922	peg	3529_9007_9162	9007	9162	+	hypothetical protein	
1955	3529	NEOS13_1923	peg	3529_9442_9242	9442	9242	-	hypothetical protein	
1956	3529	NEOS13_1924	peg	3529_10844_9609	10844	9609	-	tRNA nucleotidyltransferase (EC 2.7.7.21) (EC 2.7.7.25)	
1957	3529	NEOS13_1925	peg	3529_10898_12868	10898	12868	+	DNA mismatch repair protein MutL	
1958	3529	NEOS13_1926	peg	3529_12872_13915	12872	13915	+	Aminopeptidase YpdF (MP-, MA-, MS-, AP-, NP- specific)	
1959	3529	NEOS13_1927	peg	3529_15922_13904	15922	13904	-	hypothetical protein	
1960	3529	NEOS13_1928	peg	3529_16449_18239	16449	18239	+	Phosphate regulon sensor protein PhoR (SphS) (EC 2.7.13.3)	
1961	3529	NEOS13_1929	peg	3529_18363_19355	18363	19355	+	MoxR-like ATPase in aerotolerance operon	
1962	3529	NEOS13_1930	peg	3529_19371_20258	19371	20258	+	hypothetical protein PA3071	
1963	3529	NEOS13_1931	peg	3529_20255_21295	20255	21295	+	hypothetical protein	

1964	3529	NEOS13_1932	peg	3529_21489_22109	21489	22109	+	SSU ribosomal protein S4p (S9e)	
1965	3529	NEOS13_1933	peg	3529_22130_22243	22130	22243	+	hypothetical protein	
1966	3529	NEOS13_1934	peg	3529_24249_22735	24249	22735	-	integral membrane protein MviN	
1967	3529	NEOS13_1935	peg	3529_24517_25473	24517	25473	+	Endonuclease IV (EC 3.1.21.2)	
1968	3529	NEOS13_1936	peg	3529_25484_26887	25484	26887	+	Asparaginyl-tRNA synthetase (EC 6.1.1.22)	
1969	3529	NEOS13_1937	peg	3529_27951_28655	27951	28655	+	hypothetical protein	
1970	3529	NEOS13_1938	peg	3529_28633_28776	28633	28776	+	hypothetical protein	
1971	3529	NEOS13_1939	peg	3529_28808_28969	28808	28969	+	hypothetical protein	
1972	3529	NEOS13_1940	peg	3529_29357_29662	29357	29662	+	hypothetical protein	
1973	3529	NEOS13_1941	peg	3529_31344_29707	31344	29707	-	NAD synthetase (EC 6.3.1.5) / Glutamine amidotransferase chain of NAD synthetase	
1974	3529	NEOS13_1942	peg	3529_32693_31362	32693	31362	-	Na(+)-translocating NADH-quinone reductase subunit F (EC 1.6.5.-)	Oxidative_Phosphorylation
1975	3529	NEOS13_1943	peg	3529_32859_34715	32859	34715	+	Chaperone protein HtpG	
1976	3529	NEOS13_1944	peg	3529_34798_35934	34798	35934	+	hypothetical protein	
1977	3529	NEOS13_1945	peg	3529_36016_36189	36016	36189	+	hypothetical protein	
1978	3529	NEOS13_1946	peg	3529_36250_36933	36250	36933	+	hypothetical protein	
1979	3529	NEOS13_1947	peg	3529_37436_41953	37436	41953	+	hypothetical protein	
1980	3529	NEOS13_1948	peg	3529_42168_43589	42168	43589	+	hypothetical protein	
1981	3529	NEOS13_1949	peg	3529_44312_43848	44312	43848	-	Mobile element protein	
1982	3531	NEOS13_1950	peg	3531_1962_4133	1962	4133	+	Lead, cadmium, zinc and mercury transporting ATPase (EC 3.6.3.3) (EC 3.6.3.5); Copper-translocating P-type ATPase (EC 3.6.3.4)	
1983	3531	NEOS13_1951	peg	3531_4836_4717	4836	4717	-	hypothetical protein	
1984	3531	NEOS13_1952	peg	3531_4939_4811	4939	4811	-	hypothetical protein	
1985	3531	NEOS13_1953	peg	3531_4942_6072	4942	6072	+	hypothetical protein	
1986	3531	NEOS13_1954	peg	3531_6432_6289	6432	6289	-	hypothetical protein	
1987	3531	NEOS13_1955	peg	3531_6618_8033	6618	8033	+	DNA methylation and regulatory protein Ada(EC:2.1.1.63)	
1988	3531	NEOS13_1956	peg	3531_8062_8550	8062	8550	+	Methylated-DNA--protein-cysteine methyltransferase (EC 2.1.1.63)	
1989	3531	NEOS13_1957	peg	3531_8987_8817	8987	8817	-	hypothetical protein	
1990	3532	NEOS13_1958	peg	3532_33_1193	33	1193	+	hypothetical protein	
1991	3532	NEOS13_1959	peg	3532_1877_2053	1877	2053	+	transmembrane protein	
1992	3532	NEOS13_1960	peg	3532_2328_2588	2328	2588	+	hypothetical protein	
1993	3532	NEOS13_1961	peg	3532_2718_3419	2718	3419	+	MgtC/SapB transporter	
1994	3532	NEOS13_1962	peg	3532_3591_3749	3591	3749	+	hypothetical protein	
1995	3532	NEOS13_1963	peg	3532_4193_4975	4193	4975	+	hypothetical protein	
1996	3532	NEOS13_1964	peg	3532_5872_5453	5872	5453	-	hypothetical protein	
1997	3532	NEOS13_1965	peg	3532_7728_6220	7728	6220	-	hypothetical protein	
1998	3532	NEOS13_1966	peg	3532_8758_7685	8758	7685	-	hypothetical protein	
1999	3532	NEOS13_1967	peg	3532_8768_8977	8768	8977	+	hypothetical protein	
2000	3532	NEOS13_1968	peg	3532_9680_8994	9680	8994	-	ABC transporter-related protein	ABC_transporter
2001	3532	NEOS13_1969	peg	3532_10846_9689	10846	9689	-	ABC-transporter permease protein, putative	ABC_transporter
2002	3532	NEOS13_1970	peg	3532_11061_11996	11061	11996	+	heme ABC transporter, ATP-binding protein	ABC_transporter
2003	3532	NEOS13_1971	peg	3532_11986_12768	11986	12768	+	ABC-2 type transporter	ABC_transporter
2004	3532	NEOS13_1972	peg	3532_12809_13087	12809	13087	+	hypothetical protein	
2005	3532	NEOS13_1973	peg	3532_13210_13097	13210	13097	-	hypothetical protein	
2006	3532	NEOS13_1974	peg	3532_13764_13351	13764	13351	-	hypothetical protein	
2007	3532	NEOS13_1975	peg	3532_14197_13910	14197	13910	-	hypothetical protein	
2008	3532	NEOS13_1976	peg	3532_14271_14399	14271	14399	+	hypothetical protein	
2009	3532	NEOS13_1977	peg	3532_14624_15088	14624	15088	+	hypothetical protein	
2010	3532	NEOS13_1978	peg	3532_15089_15418	15089	15418	+	hypothetical protein	
2011	3532	NEOS13_1979	peg	3532_15735_16322	15735	16322	+	hypothetical protein	
2012	3532	NEOS13_1980	peg	3532_16319_17173	16319	17173	+	hypothetical protein	
2013	3532	NEOS13_0033rna	rna	3532_17694_17612	17694	17612	-	tRNA-Leu-CAA	RNA
2014	3532	NEOS13_1981	peg	3532_19234_17834	19234	17834	-	hypothetical protein	
2015	3532	NEOS13_1982	peg	3532_20497_19328	20497	19328	-	conserved hypothetical protein	
2016	3532	NEOS13_1983	peg	3532_20598_21290	20598	21290	+	PqqC-like protein	
2017	3532	NEOS13_1984	peg	3532_21324_22355	21324	22355	+	putative proteinase IV	
2018	3532	NEOS13_1985	peg	3532_22521_25202	22521	25202	+	DNA polymerase I (EC 2.7.7.7)	
2019	3532	NEOS13_1986	peg	3532_25436_25801	25436	25801	+	Dephospho-CoA kinase (EC 2.7.1.24)	Coenzyme_A_biosynthesis
2020	3532	NEOS13_1987	peg	3532_25823_27190	25823	27190	+	Transcription termination factor Rho	
2021	3532	NEOS13_1988	peg	3532_27286_28146	27286	28146	+	hypothetical protein	
2022	3532	NEOS13_1989	peg	3532_28516_28656	28516	28656	+	hypothetical protein	
2023	3533	NEOS13_1990	peg	3533_33_1193	33	1193	+	hypothetical protein	
2024	3533	NEOS13_1991	peg	3533_2621_1404	2621	1404	-	Leucine-rich repeat containing protein	Leucine-rich repeat
2025	3534	NEOS13_1992	peg	3534_33_1193	33	1193	+	hypothetical protein	
2026	3537	NEOS13_1993	peg	3537_404_33	404	33	-	hypothetical protein	
2027	3537	NEOS13_1994	peg	3537_1046_405	1046	405	-	hypothetical protein	
2028	3537	NEOS13_1995	peg	3537_1688_1047	1688	1047	-	hypothetical protein	
2029	3537	NEOS13_1996	peg	3537_2111_1689	2111	1689	-	hypothetical protein	
2030	3537	NEOS13_1997	peg	3537_3000_2194	3000	2194	-	hypothetical protein	
2031	3537	NEOS13_1998	peg	3537_5602_3440	5602	3440	-	hypothetical protein	
2032	3537	NEOS13_1999	peg	3537_5731_5937	5731	5937	+	hypothetical protein	
2033	3538	NEOS13_2000	peg	3538_46_1299	46	1299	+	Mobile element protein	
2034	3539	NEOS13_2001	peg	3539_24_314	24	314	+	Mobile element protein	
2035	3539	NEOS13_2002	peg	3539_422_904	422	904	+	Transposase, IS4 family protein	Transposase
2036	3539	NEOS13_2003	peg	3539_1230_1111	1230	1111	-	hypothetical protein	
2037	3539	NEOS13_2004	peg	3539_2495_1227	2495	1227	-	NADH dehydrogenase (EC 1.6.99.3)	Oxidative_Phosphorylation
2038	3539	NEOS13_2005	peg	3539_3669_2608	3669	2608	-	Cytochrome c551 peroxidase (EC 1.11.1.5)	

2039	3539	NEOS13_2006	peg	3539_3917_4069	3917	4069	+	hypothetical protein	
2040	3539	NEOS13_2007	peg	3539_5560_4481	5560	4481	-	Fructose-bisphosphate aldolase class I (EC 4.1.2.13)	Glycolysis_Glucogenesis
2041	3539	NEOS13_2008	peg	3539_5790_7505	5790	7505	+	hypothetical protein	
2042	3539	NEOS13_2009	peg	3539_8259_8074	8259	8074	-	hypothetical protein	
2043	3539	NEOS13_2010	peg	3539_8266_8859	8266	8859	+	hypothetical protein	
2044	3539	NEOS13_2011	peg	3539_9184_9363	9184	9363	+	hypothetical protein	
2045	3539	NEOS13_2012	peg	3539_9539_9658	9539	9658	+	hypothetical protein	
2046	3539	NEOS13_2013	peg	3539_12902_9843	12902	9843	-	RND multidrug efflux transporter; Acriflavin resistance protein	
2047	3539	NEOS13_2014	peg	3539_13777_12965	13777	12965	-	RND efflux membrane fusion protein, acriflavin resistance protein E	
2048	3539	NEOS13_2015	peg	3539_14688_13792	14688	13792	-	outer membrane efflux protein, putative	
2049	3539	NEOS13_2016	peg	3539_16291_15305	16291	15305	-	hypothetical protein	
2050	3539	NEOS13_2017	peg	3539_17782_16565	17782	16565	-	DEAD/DEAH box helicase-like	
2051	3539	NEOS13_2018	peg	3539_18458_18339	18458	18339	-	hypothetical protein	
2052	3540	NEOS13_2019	peg	3540_277_1773	277	1773	+	hypothetical protein	
2053	3540	NEOS13_2020	peg	3540_2361_3866	2361	3866	+	Outer membrane component of tripartite multidrug resistance system	
2054	3540	NEOS13_2021	peg	3540_4016_3834	4016	3834	-	hypothetical protein	
2055	3540	NEOS13_2022	peg	3540_4695_5150	4695	5150	+	RND family multidrug resistance secretion protein	
2056	3540	NEOS13_2023	peg	3540_5153_6703	5153	6703	+	Inner membrane component of tripartite multidrug resistance system	
2057	3540	NEOS13_2024	peg	3540_8617_6878	8617	6878	-	hypothetical protein	
2058	3540	NEOS13_2025	peg	3540_9141_8731	9141	8731	-	putative stress-induced protein OsmC	
2059	3540	NEOS13_2026	peg	3540_9478_9341	9478	9341	-	hypothetical protein	
2060	3540	NEOS13_2027	peg	3540_9774_10556	9774	10556	+	hypothetical protein	
2061	3540	NEOS13_2028	peg	3540_10541_10666	10541	10666	+	hypothetical protein	
2062	3540	NEOS13_2029	peg	3540_10975_10733	10975	10733	-	hypothetical protein	
2063	3541	NEOS13_2030	peg	3541_339_145	339	145	-	hypothetical protein	
2064	3541	NEOS13_2031	peg	3541_864_505	864	505	-	Leucine-rich repeat containing protein	Leucine-rich repeat
2065	3542	NEOS13_2032	peg	3542_537_650	537	650	+	hypothetical protein	
2066	3542	NEOS13_2033	peg	3542_1485_1345	1485	1345	-	hypothetical protein	
2067	3542	NEOS13_2034	peg	3542_1451_1888	1451	1888	+	hypothetical protein	
2068	3542	NEOS13_2035	peg	3542_2363_3040	2363	3040	+	Leucine-rich repeat containing protein	Leucine-rich repeat
2069	3542	NEOS13_2036	peg	3542_4015_4149	4015	4149	+	hypothetical protein	
2070	3542	NEOS13_2037	peg	3542_7426_4523	7426	4523	-	hypothetical protein	
2071	3542	NEOS13_2038	peg	3542_10490_8400	10490	8400	-	Polyribonucleotide nucleotidyltransferase (EC 2.7.7.8)	
2072	3542	NEOS13_2039	peg	3542_11052_10783	11052	10783	-	SSU ribosomal protein S15p (S13e)	
2073	3542	NEOS13_2040	peg	3542_11392_12066	11392	12066	+	hypothetical protein	
2074	3542	NEOS13_2041	peg	3542_12069_12572	12069	12572	+	tRNA-specific adenosine-34 deaminase (EC 3.5.4.-)	
2075	3542	NEOS13_2042	peg	3542_12959_12837	12959	12837	-	hypothetical protein	
2076	3542	NEOS13_2043	peg	3542_14550_13195	14550	13195	-	WD-40 repeat protein	
2077	3542	NEOS13_2044	peg	3542_15335_15096	15335	15096	-	hypothetical protein	
2078	3542	NEOS13_2045	peg	3542_16520_15669	16520	15669	-	hypothetical protein	
2079	3542	NEOS13_2046	peg	3542_16840_17175	16840	17175	+	LSU ribosomal protein L31p	
2080	3542	NEOS13_2047	peg	3542_17253_18320	17253	18320	+	Peptide chain release factor 1	
2081	3542	NEOS13_2048	peg	3542_18560_19408	18560	19408	+	Methylase of polypeptide chain release factors	
2082	3542	NEOS13_2049	peg	3542_19450_20778	19450	20778	+	Signal recognition particle, subunit Ffh SRP54 (TC 3.A.5.1.1)	Sec_T2SS
2083	3542	NEOS13_2050	peg	3542_20769_21101	20769	21101	+	SSU ribosomal protein S16p	
2084	3542	NEOS13_2051	peg	3542_21180_21803	21180	21803	+	tRNA (Guanine37-N1) -methyltransferase (EC 2.1.1.31)	
2085	3542	NEOS13_2052	peg	3542_21809_22225	21809	22225	+	LSU ribosomal protein L19p	
2086	3542	NEOS13_2053	peg	3542_22235_22918	22235	22918	+	Ribonuclease HII (EC 3.1.26.4)	
2087	3542	NEOS13_2054	peg	3542_23039_23791	23039	23791	+	hypothetical protein	
2088	3542	NEOS13_2055	peg	3542_24725_24853	24725	24853	+	hypothetical protein	
2089	3543	NEOS13_2056	peg	3543_540_2618	540	2618	+	Protein export cytoplasm protein SecA ATPase RNA helicase (TC 3.A.5.1.1)	
2090	3543	NEOS13_2057	peg	3543_2588_3097	2588	3097	+	hypothetical protein	
2091	3543	NEOS13_2058	peg	3543_3372_3911	3372	3911	+	hypothetical protein	
2092	3543	NEOS13_2059	peg	3543_4708_4920	4708	4920	+	hypothetical protein	
2093	3543	NEOS13_2060	peg	3543_5960_5031	5960	5031	-	Mobile element protein	
2094	3543	NEOS13_2061	peg	3543_6091_6267	6091	6267	+	hypothetical protein	
2095	3543	NEOS13_2062	peg	3543_6322_6465	6322	6465	+	hypothetical protein	
2096	3543	NEOS13_2063	peg	3543_6848_7405	6848	7405	+	Peptidyl-prolyl cis-trans isomerase (EC 5.2.1.8)	
2097	3543	NEOS13_2064	peg	3543_7641_7477	7641	7477	-	hypothetical protein	
2098	3543	NEOS13_2065	peg	3543_8729_7785	8729	7785	-	Predicted hydrolase of the alpha/beta-hydrolase fold	
2099	3543	NEOS13_2066	peg	3543_10033_9140	10033	9140	-	Methionine aminopeptidase (EC 3.4.11.18)	
2100	3543	NEOS13_2067	peg	3543_10394_10645	10394	10645	+	hypothetical protein	
2101	3543	NEOS13_2068	peg	3543_11855_10695	11855	10695	-	hypothetical protein	Outer_membrane_protein
2102	3544	NEOS13_2069	peg	3544_65_664	65	664	+	hypothetical protein	
2103	3544	NEOS13_2070	peg	3544_1406_1567	1406	1567	+	hypothetical protein	
2104	3545	NEOS13_2071	peg	3545_329_454	329	454	+	hypothetical protein	
2105	3545	NEOS13_2072	peg	3545_948_676	948	676	-	DNA-damage-inducible protein J	
2106	3545	NEOS13_2073	peg	3545_1412_1290	1412	1290	-	hypothetical protein	
2107	3547	NEOS13_2074	peg	3547_838_1407	838	1407	+	hypothetical protein	
2108	3547	NEOS13_2075	peg	3547_1658_1533	1658	1533	-	hypothetical protein	
2109	3547	NEOS13_2076	peg	3547_2116_1787	2116	1787	-	hypothetical protein	
2110	3547	NEOS13_2077	peg	3547_2487_2362	2487	2362	-	hypothetical protein	
2111	3547	NEOS13_2078	peg	3547_3233_2628	3233	2628	-	putative rRNA methylase	

2112	3547	NEOS13_2079	peg	3547_3774_3262	3774	3262	-	hypothetical protein	
2113	3547	NEOS13_2080	peg	3547_4254_3778	4254	3778	-	hypothetical protein	
2114	3547	NEOS13_2081	peg	3547_4307_4804	4307	4804	+	[leader (60) peptide-periplasmic]	
2115	3547	NEOS13_2082	peg	3547_5250_4945	5250	4945	-	SSU ribosomal protein S14p (S29e) @ SSU ribosomal protein S14p (S29e), zinc-independent	
2116	3547	NEOS13_2083	peg	3547_5403_5266	5403	5266	-	LSU ribosomal protein L36p	
2117	3547	NEOS13_2084	peg	3547_5722_5859	5722	5859	+	LSU ribosomal protein L34p	
2118	3547	NEOS13_2085	peg	3547_5856_6209	5856	6209	+	ribonuclease P	
2119	3547	NEOS13_2086	peg	3547_6193_8199	6193	8199	+	ATP-dependent DNA helicase UvrD/PcrA	
2120	3547	NEOS13_2087	peg	3547_9512_8472	9512	8472	-	thioredoxin/thiol-disulfide isomerase	
2121	3547	NEOS13_2088	peg	3547_9594_10064	9594	10064	+	hypothetical protein	
2122	3547	NEOS13_2089	peg	3547_10637_12004	10637	12004	+	sodium/alanine symporter family protein	
2123	3547	NEOS13_2090	peg	3547_12509_12703	12509	12703	+	hypothetical protein	
2124	3547	NEOS13_2091	peg	3547_13961_13359	13961	13359	-	CDP-diacylglycerol--glycerol-3-phosphate 3-phosphatidyltransferase (EC 2.7.8.5)	
2125	3547	NEOS13_2092	peg	3547_15580_14054	15580	14054	-	Glycogen synthase, ADP-glucose transglucosylase (EC 2.4.1.21)	
2126	3547	NEOS13_2093	peg	3547_15730_15873	15730	15873	+	hypothetical protein	
2127	3547	NEOS13_0034ma	rna	3547_16124_16195	16124	16195	+	tRNA-Gln-TTG	RNA
2128	3547	NEOS13_2094	peg	3547_16222_17148	16222	17148	+	Ribose-phosphate pyrophosphokinase (EC 2.7.6.1)	
2129	3547	NEOS13_2095	peg	3547_17228_17791	17228	17791	+	LSU ribosomal protein L25p	
2130	3547	NEOS13_2096	peg	3547_17828_18400	17828	18400	+	Peptidyl-tRNA hydrolase (EC 3.1.1.29)	
2131	3547	NEOS13_2097	peg	3547_18442_18789	18442	18789	+	SSU ribosomal protein S6p	
2132	3547	NEOS13_2098	peg	3547_18802_19059	18802	19059	+	SSU ribosomal protein S18p @ SSU ribosomal protein S18p, zinc-independent	
2133	3547	NEOS13_2099	peg	3547_19078_19575	19078	19575	+	LSU ribosomal protein L9p	
2134	3547	NEOS13_2100	peg	3547_19671_20516	19671	20516	+	4-diphosphocytidyl-2-C-methyl-D-erythritol kinase (EC 2.7.1.148)	
2135	3547	NEOS13_2101	peg	3547_20749_21735	20749	21735	+	ADP-L-glycero-D-manno-heptose-6-epimerase (EC 5.1.3.20)	
2136	3547	NEOS13_2102	peg	3547_21795_23021	21795	23021	+	ADP-heptose synthase (EC 2.7.-.-) / D-glycero-beta-D-manno-heptose 7-phosphate kinase	
2137	3547	NEOS13_2103	peg	3547_23447_25234	23447	25234	+	DNA primase (EC 2.7.7.-)	
2138	3547	NEOS13_2104	peg	3547_25315_26994	25315	26994	+	Leucine-rich repeat containing protein	Leucine-rich repeat
2139	3547	NEOS13_2105	peg	3547_28532_27348	28532	27348	-	Outer membrane autotransporter barrel	
2140	3547	NEOS13_2106	peg	3547_29105_28695	29105	28695	-	hypothetical protein	
2141	3547	NEOS13_2107	peg	3547_29480_29277	29480	29277	-	hypothetical protein	
2142	3547	NEOS13_2108	peg	3547_31301_29637	31301	29637	-	Phosphoglucomutase (EC 5.4.2.2)	
2143	3547	NEOS13_2109	peg	3547_31666_32172	31666	32172	+	Non-specific DNA-binding protein Dps / Iron-binding ferritin-like antioxidant protein / Ferroxidase (EC 1.16.3.1)	
2144	3547	NEOS13_2110	peg	3547_32177_32296	32177	32296	+	hypothetical protein	
2145	3547	NEOS13_2111	peg	3547_32622_33518	32622	33518	+	Cytochrome O ubiquinol oxidase subunit II (EC 1.10.3.-)	Oxidative_Phosphorylation
2146	3547	NEOS13_2112	peg	3547_33558_33866	33558	33866	+	Cytochrome O ubiquinol oxidase subunit I (EC 1.10.3.-)	Oxidative_Phosphorylation
2147	3548	NEOS13_2113	peg	3548_295_182	295	182	-	hypothetical protein	
2148	3548	NEOS13_2114	peg	3548_1102_524	1102	524	-	hypothetical protein	
2149	3548	NEOS13_2115	peg	3548_2936_1659	2936	1659	-	putative aminopeptidase 2	
2150	3548	NEOS13_2116	peg	3548_4579_3302	4579	3302	-	Seryl-tRNA synthetase (EC 6.1.1.11)	
2151	3548	NEOS13_2117	peg	3548_4854_7778	4854	7778	+	peptidase M16 domain protein	
2152	3548	NEOS13_2118	peg	3548_9651_8311	9651	8311	-	High-affnity carbon uptake protein Hat/HatR	
2153	3548	NEOS13_2119	peg	3548_9819_9974	9819	9974	+	hypothetical protein	
2154	3548	NEOS13_2120	peg	3548_11067_10042	11067	10042	-	putative glycerol-3-phosphate acyltransferase	
2155	3548	NEOS13_2121	peg	3548_12614_11076	12614	11076	-	Cytoplasmic axial filament protein CafA and Ribonuclease G (EC 3.1.4.-)	
2156	3548	NEOS13_2122	peg	3548_13100_13615	13100	13615	+	hypothetical protein	
2157	3548	NEOS13_2123	peg	3548_13647_13826	13647	13826	+	LSU ribosomal protein L32p	
2158	3548	NEOS13_2124	peg	3548_13847_14863	13847	14863	+	Phosphate:acyl-ACP acyltransferase PlsX	
2159	3548	NEOS13_2125	peg	3548_15464_15006	15464	15006	-	Error-prone repair protein UmuD	
2160	3548	NEOS13_2126	peg	3548_16267_15584	16267	15584	-	hypothetical protein	
2161	3548	NEOS13_2127	peg	3548_17427_16264	17427	16264	-	Lipid-A-disaccharide synthase (EC 2.4.1.182)	
2162	3548	NEOS13_2128	peg	3548_18121_17417	18121	17417	-	Lipid-A-disaccharide synthase (EC 2.4.1.182)	
2163	3548	NEOS13_2129	peg	3548_18834_18118	18834	18118	-	putative dolichol-phosphate mannosyltransferase	
2164	3548	NEOS13_2130	peg	3548_19790_19047	19790	19047	-	unknown protein	
2165	3548	NEOS13_2131	peg	3548_21085_19811	21085	19811	-	Poly(A) polymerase (EC 2.7.7.19)	
2166	3548	NEOS13_2132	peg	3548_21435_22802	21435	22802	+	Phosphoglucosamine mutase (EC 5.4.2.10)	
2167	3548	NEOS13_2133	peg	3548_22824_24653	22824	24653	+	Glucosamine--fructose-6-phosphate aminotransferase [isomerizing] (EC 2.6.1.16)	
2168	3548	NEOS13_2134	peg	3548_25024_25149	25024	25149	+	hypothetical protein	
2169	3550	NEOS13_2135	peg	3550_48_470	48	470	+	Mobile element protein	
2170	3550	NEOS13_2136	peg	3550_513_701	513	701	+	hypothetical protein	

2171	3550	NEOS13_2137	peg	3550_834_2024	834	2024	+	hypothetical protein	
2172	3550	NEOS13_2138	peg	3550_2310_2065	2310	2065	-	hypothetical protein	
2173	3550	NEOS13_2139	peg	3550_2792_2394	2792	2394	-	hypothetical protein	
2174	3550	NEOS13_2140	peg	3550_3488_3610	3488	3610	+	hypothetical protein	
2175	3550	NEOS13_2141	peg	3550_3993_3631	3993	3631	-	hypothetical protein	
2176	3550	NEOS13_2142	peg	3550_5186_4257	5186	4257	-	Signal recognition particle receptor protein FtsY (=alpha subunit) (TC 3.A.5.1.1)	Sec_T2SS
2177	3550	NEOS13_2143	peg	3550_5723_5595	5723	5595	-	hypothetical protein	
2178	3550	NEOS13_2144	peg	3550_5868_7049	5868	7049	+	hypothetical protein	
2179	3550	NEOS13_2145	peg	3550_7164_7595	7164	7595	+	hypothetical protein	
2180	3550	NEOS13_2146	peg	3550_7903_8136	7903	8136	+	hypothetical protein	
2181	3550	NEOS13_2147	peg	3550_9607_8189	9607	8189	-	HtrA protease/chaperone protein	
2182	3550	NEOS13_2148	peg	3550_11003_9618	11003	9618	-	Catalyzes the cleavage of p-aminobenzoyl-glutamate to p-aminobenzoate and glutamate, subunit A	
2183	3550	NEOS13_2149	peg	3550_12426_11119	12426	11119	-	Peptidase M48, Ste24p	
2184	3550	NEOS13_2150	peg	3550_13876_13154	13876	13154	-	hypothetical protein	
2185	3551	NEOS13_2151	peg	3551_116_1510	116	1510	+	Leucine-rich repeat containing protein	Leucine-rich repeat
2186	3552	NEOS13_2152	peg	3552_1029_226	1029	226	-	hypothetical protein	
2187	3552	NEOS13_2153	peg	3552_1763_1032	1763	1032	-	hypothetical protein	
2188	3552	NEOS13_2154	peg	3552_1811_1924	1811	1924	+	hypothetical protein	
2189	3552	NEOS13_2155	peg	3552_3934_2570	3934	2570	-	putative protoporphyrinogen oxidase	
2190	3552	NEOS13_2156	peg	3552_4968_3931	4968	3931	-	Uroporphyrinogen III decarboxylase (EC 4.1.1.37)	
2191	3552	NEOS13_2157	peg	3552_7016_4968	7016	4968	-	Transketolase (EC 2.2.1.1)	Pentose_phospate_pathway
2192	3552	NEOS13_2158	peg	3552_7091_8050	7091	8050	+	hypothetical protein	
2193	3552	NEOS13_2159	peg	3552_8753_8235	8753	8235	-	Mobile element protein	
2194	3553	NEOS13_2160	peg	3553_819_253	819	253	-	DNA polymerase III beta subunit (EC 2.7.7.7)	
2195	3554	NEOS13_2161	peg	3554_1030_227	1030	227	-	Leucine-rich repeat containing protein	Leucine-rich repeat
2196	3556	NEOS13_2162	peg	3556_74_415	74	415	+	Transposase, IS4 family protein	Transposase
2197	3556	NEOS13_2163	peg	3556_852_2048	852	2048	+	Leucine-rich repeat containing protein	Leucine-rich repeat
2198	3556	NEOS13_2164	peg	3556_2553_2756	2553	2756	+	hypothetical protein	
2199	3556	NEOS13_2165	peg	3556_2737_3009	2737	3009	+	transposase, IS4 family	Transposase
2200	3556	NEOS13_2166	peg	3556_2972_3181	2972	3181	+	hypothetical protein	
2201	3557	NEOS13_2167	peg	3557_966_622	966	622	-	Leucine-rich repeat containing protein	Leucine-rich repeat
2202	3558	NEOS13_2168	peg	3558_576_463	576	463	-	hypothetical protein	
2203	3558	NEOS13_2169	peg	3558_1031_723	1031	723	-	Leucine-rich repeat containing protein	Leucine-rich repeat
2204	3558	NEOS13_2170	peg	3558_1221_1000	1221	1000	-	hypothetical protein	
2205	3558	NEOS13_2171	peg	3558_1267_1422	1267	1422	+	hypothetical protein	
2206	3558	NEOS13_2172	peg	3558_2254_1577	2254	1577	-	Leucine-rich repeat containing protein	Leucine-rich repeat
2207	3559	NEOS13_2173	peg	3559_121_447	121	447	+	transposase, IS4 family	Transposase
2208	3559	NEOS13_2174	peg	3559_763_2454	763	2454	+	Leucine-rich repeat containing protein	Leucine-rich repeat
2209	3560	NEOS13_2175	peg	3560_58_576	58	576	+	Mobile element protein	
2210	3561	NEOS13_2176	peg	3561_1971_1852	1971	1852	-	hypothetical protein	
2211	3562	NEOS13_2177	peg	3562_1542_2003	1542	2003	+	hypothetical protein	
2212	3562	NEOS13_2178	peg	3562_2070_3389	2070	3389	+	hypothetical protein	
2213	3562	NEOS13_2179	peg	3562_3451_3597	3451	3597	+	hypothetical protein	
2214	3562	NEOS13_2180	peg	3562_3810_3691	3810	3691	-	hypothetical protein	
2215	3562	NEOS13_2181	peg	3562_3869_4069	3869	4069	+	hypothetical protein	
2216	3562	NEOS13_2182	peg	3562_4406_4266	4406	4266	-	hypothetical protein	
2217	3562	NEOS13_2183	peg	3562_4419_7067	4419	7067	+	Alanyl-tRNA synthetase (EC 6.1.1.7)	
2218	3562	NEOS13_2184	peg	3562_7396_7617	7396	7617	+	hypothetical protein	
2219	3562	NEOS13_2185	peg	3562_8993_10906	8993	10906	+	Transcription-repair coupling factor	
2220	3562	NEOS13_2186	peg	3562_11641_11036	11641	11036	-	Holliday junction DNA helicase RuvA	
2221	3562	NEOS13_2187	peg	3562_12159_11662	12159	11662	-	Crossover junction endodeoxyribonuclease RuvC (EC 3.1.22.4)	
2222	3562	NEOS13_2188	peg	3562_14220_12169	14220	12169	-	liver stage antigen, putative	
2223	3562	NEOS13_2189	peg	3562_14340_15404	14340	15404	+	ADP-heptose--lipooligosaccharide heptosyltransferase II (EC 2.4.1.-)	
2224	3562	NEOS13_2190	peg	3562_16065_17420	16065	17420	+	N-acetylglucosamine-1-phosphate uridyltransferase eukaryotic (EC 2.7.7.23)	
2225	3562	NEOS13_2191	peg	3562_17405_17551	17405	17551	+	hypothetical protein	
2226	3562	NEOS13_2192	peg	3562_17634_18632	17634	18632	+	Glycerol-3-phosphate dehydrogenase [NAD(P)+] (EC 1.1.1.94)	
2227	3562	NEOS13_2193	peg	3562_18683_20176	18683	20176	+	NAD(P)HX epimerase / NAD(P)HX dehydratase	
2228	3562	NEOS13_2194	peg	3562_21798_20569	21798	20569	-	Leucine-rich repeat containing protein	Leucine-rich repeat
2229	3562	NEOS13_2195	peg	3562_21802_21993	21802	21993	+	hypothetical protein	
2230	3562	NEOS13_2196	peg	3562_22271_22591	22271	22591	+	hypothetical protein	
2231	3562	NEOS13_2197	peg	3562_22599_22745	22599	22745	+	Mobile element protein	
2232	3562	NEOS13_2198	peg	3562_22807_22962	22807	22962	+	hypothetical protein	
2233	3562	NEOS13_2199	peg	3562_23296_23171	23296	23171	-	hypothetical protein	
2234	3562	NEOS13_2200	peg	3562_24099_23563	24099	23563	-	hypothetical protein	
2235	3562	NEOS13_2201	peg	3562_26315_24096	26315	24096	-	hypothetical protein	
2236	3562	NEOS13_2202	peg	3562_26795_26325	26795	26325	-	hypothetical protein	
2237	3562	NEOS13_2203	peg	3562_27226_27032	27226	27032	-	hypothetical protein	
2238	3562	NEOS13_2204	peg	3562_27280_27432	27280	27432	+	hypothetical protein	
2239	3562	NEOS13_2205	peg	3562_27541_30714	27541	30714	+	hypothetical protein	
2240	3562	NEOS13_2206	peg	3562_33747_30772	33747	30772	-	hypothetical protein	
2241	3562	NEOS13_2207	peg	3562_34502_34320	34502	34320	-	hypothetical protein	
2242	3562	NEOS13_2208	peg	3562_34686_34567	34686	34567	-	hypothetical protein	
2243	3562	NEOS13_2209	peg	3562_34835_34716	34835	34716	-	hypothetical protein	
2244	3562	NEOS13_2210	peg	3562_35071_34853	35071	34853	-	hypothetical protein	

2245	3562	NEOS13_2211	peg	3562_35223_37283	35223	37283	+	hypothetical protein	
2246	3562	NEOS13_2212	peg	3562_37360_38493	37360	38493	+	hypothetical protein	
2247	3562	NEOS13_2213	peg	3562_38562_40220	38562	40220	+	hypothetical protein	
2248	3562	NEOS13_2214	peg	3562_40213_42912	40213	42912	+	WD-40 repeat protein	
2249	3562	NEOS13_2215	peg	3562_43061_43255	43061	43255	+	hypothetical protein	
2250	3562	NEOS13_2216	peg	3562_43784_43590	43784	43590	-	hypothetical protein	
2251	3563	NEOS13_2217	peg	3563_780_352	780	352	-	transposase, IS4 family protein	Transposase
2252	3563	NEOS13_2218	peg	3563_898_1134	898	1134	+	hypothetical protein	
2253	3563	NEOS13_2219	peg	3563_1392_1505	1392	1505	+	hypothetical protein	
2254	3563	NEOS13_2220	peg	3563_1506_1955	1506	1955	+	putative rhs core protein with extension	
2255	3563	NEOS13_2221	peg	3563_2037_2519	2037	2519	+	unknown protein	
2256	3563	NEOS13_2222	peg	3563_2778_2939	2778	2939	+	hypothetical protein	
2257	3563	NEOS13_2223	peg	3563_4004_2928	4004	2928	-	hypothetical protein	ABC_transporter
2258	3563	NEOS13_2224	peg	3563_5152_4004	5152	4004	-	hypothetical protein	ABC_transporter
2259	3563	NEOS13_2225	peg	3563_5441_5779	5441	5779	+	hypothetical protein	
2260	3563	NEOS13_2226	peg	3563_5784_7109	5784	7109	+	tRNA(Ile)-lysidine synthetase	
2261	3563	NEOS13_2227	peg	3563_7503_10268	7503	10268	+	Cell division protein FtsH (EC 3.4.24.-)	
2262	3563	NEOS13_2228	peg	3563_10289_10417	10289	10417	+	hypothetical protein	
2263	3563	NEOS13_2229	peg	3563_10494_11030	10494	11030	+	Adenylate kinase (EC 2.7.4.3)	
2264	3563	NEOS13_2230	peg	3563_11166_12044	11166	12044	+	hypothetical protein	
2265	3563	NEOS13_2231	peg	3563_14149_12908	14149	12908	-	hypothetical protein	
2266	3565	NEOS13_2232	peg	3565_196_1029	196	1029	+	transposase, IS4	Transposase
2267	3565	NEOS13_2233	peg	3565_1246_1362	1246	1362	+	hypothetical protein	
2268	3565	NEOS13_2234	peg	3565_3075_1630	3075	1630	-	RND efflux system, outer membrane lipoprotein CmeC	
2269	3565	NEOS13_2235	peg	3565_6229_3068	6229	3068	-	RND efflux system, inner membrane transporter CmeB	
2270	3565	NEOS13_2236	peg	3565_7411_6233	7411	6233	-	hypothetical protein	
2271	3565	NEOS13_2237	peg	3565_7563_8687	7563	8687	+	Fatty acid desaturase (EC 1.14.19.1); Delta-9 fatty acid desaturase (EC 1.14.19.1)	
2272	3565	NEOS13_2238	peg	3565_10186_8768	10186	8768	-	hypothetical protein	
2273	3565	NEOS13_0035ma	rna	3565_10402_10472	10402	10472	+	tRNA-Cys-GCA	RNA
2274	3565	NEOS13_2239	peg	3565_10555_11385	10555	11385	+	rRNA small subunit methyltransferase I	
2275	3565	NEOS13_2240	peg	3565_11477_12754	11477	12754	+	putative facilitator of salicylate uptake	
2276	3565	NEOS13_2241	peg	3565_15009_13318	15009	13318	-	Leucine-rich repeat containing protein	Leucine-rich repeat
2277	3565	NEOS13_2242	peg	3565_15509_15673	15509	15673	+	hypothetical protein	
2278	3565	NEOS13_2243	peg	3565_15660_15797	15660	15797	+	hypothetical protein	
2279	3565	NEOS13_2244	peg	3565_16299_16526	16299	16526	+	hypothetical protein	
2280	3565	NEOS13_2245	peg	3565_16665_16856	16665	16856	+	hypothetical protein	
2281	3565	NEOS13_2246	peg	3565_17114_18715	17114	18715	+	Heat shock protein 60 family chaperone GroEL	
2282	3565	NEOS13_2247	peg	3565_18825_20078	18825	20078	+	hypothetical protein	
2283	3565	NEOS13_2248	peg	3565_20095_21153	20095	21153	+	Hemin ABC transporter, permease protein	ABC_transporter
2284	3565	NEOS13_2249	peg	3565_21146_21856	21146	21856	+	hypothetical protein	
2285	3565	NEOS13_2250	peg	3565_22125_23918	22125	23918	+	Phosphoglucomutase (EC 5.4.2.2)	
2286	3565	NEOS13_2251	peg	3565_24042_25937	24042	25937	+	Glutamine amidotransferase	
2287	3565	NEOS13_2252	peg	3565_27388_26222	27388	26222	-	Cold-shock DEAD-box protein A	
2288	3565	NEOS13_2253	peg	3565_28194_27385	28194	27385	-	hypothetical protein	
2289	3565	NEOS13_2254	peg	3565_30343_28163	30343	28163	-	Membrane protein containing HD superfamily hydrolase domain, YQFF ortholog	
2290	3565	NEOS13_2255	peg	3565_30719_31702	30719	31702	+	UDP-glucose 4-epimerase (EC 5.1.3.2)	
2291	3565	NEOS13_2256	peg	3565_32210_32085	32210	32085	-	hypothetical protein	
2292	3565	NEOS13_2257	peg	3565_32220_33758	32220	33758	+	DNA polymerase III subunits gamma and tau (EC 2.7.7.7)	
2293	3565	NEOS13_2258	peg	3565_33767_34081	33767	34081	+	conserved hypothetical protein 103	
2294	3565	NEOS13_2259	peg	3565_36224_34464	36224	34464	-	Phosphoenolpyruvate-protein phosphotransferase of PTS system (EC 2.7.3.9)	
2295	3565	NEOS13_2260	peg	3565_36457_36227	36457	36227	-	Phosphocarrier protein of PTS system	
2296	3565	NEOS13_2261	peg	3565_37442_36465	37442	36465	-	HPr kinase/phosphorylase (EC 2.7.1.-) (EC 2.7.4.-)	
2297	3565	NEOS13_2262	peg	3565_37582_37448	37582	37448	-	hypothetical protein	
2298	3565	NEOS13_2263	peg	3565_37931_37809	37931	37809	-	hypothetical protein	
2299	3565	NEOS13_2264	peg	3565_38079_37885	38079	37885	-	hypothetical protein	
2300	3565	NEOS13_2265	peg	3565_38396_39004	38396	39004	+	hypothetical protein	
2301	3565	NEOS13_2266	peg	3565_39144_40553	39144	40553	+	Late competence protein ComEC, DNA transport	Genetic_competence
2302	3565	NEOS13_2267	peg	3565_44647_40790	44647	40790	-	hypothetical protein	
2303	3565	NEOS13_2268	peg	3565_44834_44706	44834	44706	-	hypothetical protein	
2304	3565	NEOS13_2269	peg	3565_48539_45015	48539	45015	-	hypothetical protein	
2305	3565	NEOS13_2270	peg	3565_49396_49043	49396	49043	-	hypothetical protein	
2306	3565	NEOS13_2271	peg	3565_52604_49377	52604	49377	-	hypothetical protein	
2307	3565	NEOS13_2272	peg	3565_52879_53037	52879	53037	+	hypothetical protein	
2308	3565	NEOS13_0036ma	rna	3565_53028_53101	53028	53101	+	tRNA-His-GTG	RNA
2309	3565	NEOS13_2273	peg	3565_53127_53921	53127	53921	+	Undecaprenyl-diphosphatase (EC 3.6.1.27)	
2310	3565	NEOS13_2274	peg	3565_53930_56437	53930	56437	+	Cell division protein FtsK	
2311	3565	NEOS13_2275	peg	3565_56935_56549	56935	56549	-	hypothetical protein	
2312	3565	NEOS13_2276	peg	3565_57105_57878	57105	57878	+	metal dependent hydrolase, putative	
2313	3565	NEOS13_2277	peg	3565_58836_58045	58836	58045	-	oxidoreductase, short chain dehydrogenase/reductase family	
2314	3565	NEOS13_2278	peg	3565_59141_59257	59141	59257	+	hypothetical protein	
2315	3565	NEOS13_2279	peg	3565_59747_59595	59747	59595	-	hypothetical protein	

2316	3565	NEOS13_2280	peg	3565_61096_60122	61096	60122	-	hypothetical protein	
2317	3565	NEOS13_2281	peg	3565_63154_61229	63154	61229	-	hypothetical protein	
2318	3565	NEOS13_2282	peg	3565_67566_63190	67566	63190	-	hypothetical protein	
2319	3565	NEOS13_2283	peg	3565_68125_67529	68125	67529	-	hypothetical protein	
2320	3565	NEOS13_2284	peg	3565_69036_68530	69036	68530	-	hypothetical protein	
2321	3565	NEOS13_2285	peg	3565_69253_69116	69253	69116	-	hypothetical protein	
2322	3565	NEOS13_2286	peg	3565_69504_69373	69504	69373	-	hypothetical protein	
2323	3565	NEOS13_2287	peg	3565_69735_70043	69735	70043	+	hypothetical protein	
2324	3566	NEOS13_2288	peg	3566_2915_1998	2915	1998	-	Riboflavin kinase (EC 2.7.1.26) / FMN adenylyltransferase (EC 2.7.7.2)	
2325	3566	NEOS13_2289	peg	3566_3035_2919	3035	2919	-	hypothetical protein	
2326	3566	NEOS13_2290	peg	3566_3907_3185	3907	3185	-	tRNA pseudouridine synthase B (EC 4.2.1.70)	
2327	3566	NEOS13_2291	peg	3566_4268_3900	4268	3900	-	Ribosome-binding factor A	
2328	3566	NEOS13_2292	peg	3566_7062_4294	7062	4294	-	Translation initiation factor 2	
2329	3566	NEOS13_2293	peg	3566_8341_7034	8341	7034	-	Transcription termination protein NusA	
2330	3566	NEOS13_2294	peg	3566_10234_8492	10234	8492	-	SSU ribosomal protein S1p	
2331	3566	NEOS13_2295	peg	3566_10847_10482	10847	10482	-	Queuosine biosynthesis QueD, PTPS-I	
2332	3566	NEOS13_2296	peg	3566_10980_11750	10980	11750	+	Aspartokinase (EC 2.7.2.4)	
2333	3566	NEOS13_2297	peg	3566_11779_13704	11779	13704	+	unknown protein	
2334	3566	NEOS13_2298	peg	3566_14042_14752	14042	14752	+	Ribulose-phosphate 3-epimerase (EC 5.1.3.1)	Pentose_phospate_pathway
2335	3566	NEOS13_2299	peg	3566_15015_15572	15015	15572	+	Translation elongation factor P-related protein	
2336	3566	NEOS13_2300	peg	3566_15586_16077	15586	16077	+	Biotin carboxyl carrier protein of acetyl-CoA carboxylase	Fatty _acid_initiation_elongation
2337	3566	NEOS13_2301	peg	3566_16087_17451	16087	17451	+	Biotin carboxylase of acetyl-CoA carboxylase (EC 6.3.4.14)	Fatty _acid_initiation_elongation
2338	3566	NEOS13_2302	peg	3566_18055_23004	18055	23004	+	hypothetical protein	
2339	3566	NEOS13_2303	peg	3566_23493_23639	23493	23639	+	Mobile element protein	
2340	3566	NEOS13_2304	peg	3566_23851_24177	23851	24177	+	ISSod13, transposase	Transposase
2341	3566	NEOS13_2305	peg	3566_24338_24679	24338	24679	+	hypothetical protein	
2342	3566	NEOS13_2306	peg	3566_26108_24687	26108	24687	-	hypothetical protein	
2343	3566	NEOS13_2307	peg	3566_27146_27649	27146	27649	+	Transposase	Transposase
2344	3566	NEOS13_2308	peg	3566_28517_27834	28517	27834	-	hypothetical protein	
2345	3566	NEOS13_2309	peg	3566_29804_28884	29804	28884	-	Epoxyqueuosine (oQ) reductase QueG	
2346	3566	NEOS13_2310	peg	3566_30733_29801	30733	29801	-	Uncharacterized membrane protein Bsu2508 (YqfU)	
2347	3566	NEOS13_0037rna	rna	3566_30992_31064	30992	31064	+	tRNA-Val-TAC	RNA
2348	3566	NEOS13_0038rna	rna	3566_31076_31149	31076	31149	+	tRNA-Asp-GTC	RNA
2349	3568	NEOS13_2311	peg	3568_714_190	714	190	-	hypothetical protein	
2350	3568	NEOS13_2312	peg	3568_1178_1065	1178	1065	-	hypothetical protein	
2351	3568	NEOS13_2313	peg	3568_1627_1496	1627	1496	-	hypothetical protein	
2352	3568	NEOS13_2314	peg	3568_2206_2730	2206	2730	+	hypothetical protein	
2353	3569	NEOS13_2315	peg	3569_35_4213	35	4213	+	hypothetical protein	
2354	3571	NEOS13_2316	peg	3571_79_999	79	999	+	hypothetical protein	
2355	3573	NEOS13_2317	peg	3573_2261_2413	2261	2413	+	hypothetical protein	
2356	3573	NEOS13_2318	peg	3573_2678_2415	2678	2415	-	hypothetical protein	
2357	3573	NEOS13_2319	peg	3573_3004_3264	3004	3264	+	Mobile element protein	
2358	3573	NEOS13_2320	peg	3573_4591_3590	4591	3590	-	hypothetical protein	
2359	3573	NEOS13_2321	peg	3573_5068_4751	5068	4751	-	hypothetical protein	
2360	3573	NEOS13_2322	peg	3573_5881_6381	5881	6381	+	Mobile element protein	
2361	3573	NEOS13_2323	peg	3573_6933_7355	6933	7355	+	hypothetical protein	
2362	3573	NEOS13_2324	peg	3573_7566_7441	7566	7441	-	hypothetical protein	
2363	3573	NEOS13_2325	peg	3573_7933_10458	7933	10458	+	putative alanine racemase	
2364	3573	NEOS13_2326	peg	3573_11360_10455	11360	10455	-	Cell division inhibitor	
2365	3573	NEOS13_2327	peg	3573_11963_11382	11963	11382	-	PPO candidate 1	
2366	3573	NEOS13_2328	peg	3573_13616_12189	13616	12189	-	Deoxyribodipyrimidine photolyase (EC 4.1.99.3)	
2367	3574	NEOS13_2329	peg	3574_165_995	165	995	+	Mobile element protein	
2368	3575	NEOS13_2330	peg	3575_165_995	165	995	+	Mobile element protein	
2369	3575	NEOS13_2331	peg	3575_992_1774	992	1774	+	Mobile element protein	
2370	3576	NEOS13_2332	peg	3576_1477_134	1477	134	-	Mobile element protein	
2371	3576	NEOS13_2333	peg	3576_1840_2526	1840	2526	+	Uracil-DNA glycosylase, family 5	
2372	3576	NEOS13_2334	peg	3576_2527_2655	2527	2655	+	hypothetical protein	
2373	3576	NEOS13_2335	peg	3576_2994_5747	2994	5747	+	Leucine-rich repeat containing protein	Leucine-rich repeat
2374	3576	NEOS13_2336	peg	3576_6740_5856	6740	5856	-	Dihydrodipicolinate synthase (EC 4.2.1.52)	
2375	3576	NEOS13_2337	peg	3576_7512_6730	7512	6730	-	Dihydrodipicolinate reductase (EC 1.3.1.26)	
2376	3576	NEOS13_2338	peg	3576_8720_7671	8720	7671	-	hypothetical protein	
2377	3576	NEOS13_2339	peg	3576_10903_8720	10903	8720	-	RecD-like DNA helicase YrrC	
2378	3576	NEOS13_2340	peg	3576_11209_11451	11209	11451	+	hypothetical protein	
2379	3576	NEOS13_2341	peg	3576_11818_12021	11818	12021	+	hypothetical protein	
2380	3576	NEOS13_2342	peg	3576_12520_14574	12520	14574	+	hypothetical protein	
2381	3576	NEOS13_2343	peg	3576_15593_14700	15593	14700	-	Methionine aminopeptidase (EC 3.4.11.18)	
2382	3576	NEOS13_2344	peg	3576_15810_16496	15810	16496	+	tRNA (guanine46-N7-)-methyltransferase (EC 2.1.1.33)	
2383	3576	NEOS13_2345	peg	3576_16493_17539	16493	17539	+	hypothetical protein	
2384	3576	NEOS13_2346	peg	3576_17717_19336	17717	19336	+	Glucose-6-phosphate isomerase (EC 5.3.1.9)	Glycolysis_Glucogenesis Pentose_phospate_pathway
2385	3576	NEOS13_2347	peg	3576_19355_19861	19355	19861	+	hypothetical protein	
2386	3576	NEOS13_2348	peg	3576_19839_20768	19839	20768	+	probable XerD protein	
2387	3576	NEOS13_2349	peg	3576_21316_20810	21316	20810	-	type III secretion chaperone	
2388	3576	NEOS13_2350	peg	3576_21437_21841	21437	21841	+	hypothetical protein	

2389	3576	NEOS13_2351	peg	3576_21953_21804	21953	21804	-	hypothetical protein	
2390	3576	NEOS13_2352	peg	3576_23712_22252	23712	22252	-	Aspartyl-tRNA(Asn) amidotransferase subunit B (EC 6.3.5.6) @ Glutamyl-tRNA(Gln) amidotransferase subunit B (EC 6.3.5.7)	
2391	3576	NEOS13_2353	peg	3576_25180_23723	25180	23723	-	Aspartyl-tRNA(Asn) amidotransferase subunit A (EC 6.3.5.6) @ Glutamyl-tRNA(Gln) amidotransferase subunit A (EC 6.3.5.7)	
2392	3576	NEOS13_2354	peg	3576_25497_25198	25497	25198	-	Aspartyl-tRNA(Asn) amidotransferase subunit C (EC 6.3.5.6) @ Glutamyl-tRNA(Gln) amidotransferase subunit C (EC 6.3.5.7)	
2393	3576	NEOS13_2355	peg	3576_28465_26573	28465	26573	-	hypothetical protein	
2394	3578	NEOS13_2356	peg	3578_165_49	165	49	-	Mobile element protein	
2395	3578	NEOS13_2357	peg	3578_508_642	508	642	+	hypothetical protein	
2396	3578	NEOS13_2358	peg	3578_697_921	697	921	+	hypothetical protein	
2397	3581	NEOS13_2359	peg	3581_254_2635	254	2635	+	hypothetical protein	
2398	3581	NEOS13_2360	peg	3581_2639_3190	2639	3190	+	hypothetical protein	
2399	3581	NEOS13_2361	peg	3581_3245_3376	3245	3376	+	hypothetical protein	
2400	3581	NEOS13_2362	peg	3581_4472_3813	4472	3813	-	Queuosine Biosynthesis QueC ATPase	
2401	3581	NEOS13_2363	peg	3581_5122_4475	5122	4475	-	Queuosine Biosynthesis QueE Radical SAM	
2402	3581	NEOS13_2364	peg	3581_5433_7274	5433	7274	+	ankyrin repeat protein	Ankyrin
2403	3581	NEOS13_2365	peg	3581_7342_7455	7342	7455	+	hypothetical protein	
2404	3581	NEOS13_2366	peg	3581_7457_7570	7457	7570	+	hypothetical protein	
2405	3581	NEOS13_2367	peg	3581_7663_8673	7663	8673	+	Malate dehydrogenase (EC 1.1.1.37)	TCA
2406	3581	NEOS13_2368	peg	3581_8932_9759	8932	9759	+	hypothetical protein	
2407	3581	NEOS13_2369	peg	3581_10932_9916	10932	9916	-	hypothetical protein	
2408	3581	NEOS13_2370	peg	3581_11149_11334	11149	11334	+	hypothetical protein	
2409	3581	NEOS13_2371	peg	3581_11331_12137	11331	12137	+	Metal-dependent hydrolases of the beta-lactamase superfamily I; PhnP protein	
2410	3581	NEOS13_2372	peg	3581_12149_13474	12149	13474	+	GTP-binding protein HflX	
2411	3581	NEOS13_2373	peg	3581_15242_13764	15242	13764	-	Cytosol aminopeptidase PepA (EC 3.4.11.1)	
2412	3581	NEOS13_2374	peg	3581_15781_15263	15781	15263	-	Single-stranded DNA-binding protein	
2413	3581	NEOS13_2375	peg	3581_15993_15838	15993	15838	-	hypothetical protein	
2414	3581	NEOS13_2376	peg	3581_16727_16206	16727	16206	-	FIG00493912: hypothetical protein	
2415	3581	NEOS13_2377	peg	3581_17239_19284	17239	19284	+	Glycogen debranching enzyme (EC 3.2.1.-)	
2416	3581	NEOS13_2378	peg	3581_19944_19813	19944	19813	-	hypothetical protein	
2417	3581	NEOS13_2379	peg	3581_20179_22455	20179	22455	+	hypothetical protein	
2418	3581	NEOS13_2380	peg	3581_24404_22791	24404	22791	-	DNA repair protein RecN	
2419	3581	NEOS13_2381	peg	3581_24506_24667	24506	24667	+	hypothetical protein	
2420	3581	NEOS13_2382	peg	3581_25763_24849	25763	24849	-	Ribonuclease HIII (EC 3.1.26.4)	
2421	3581	NEOS13_2383	peg	3581_25865_26275	25865	26275	+	FIG00899448: hypothetical protein	
2422	3581	NEOS13_2384	peg	3581_26420_26304	26420	26304	-	hypothetical protein	
2423	3581	NEOS13_2385	peg	3581_26566_27510	26566	27510	+	FIG028593: membrane protein	
2424	3581	NEOS13_2386	peg	3581_28381_27767	28381	27767	-	tRNA pseudouridine synthase A (EC 4.2.1.70)	
2425	3581	NEOS13_2387	peg	3581_28513_28394	28513	28394	-	hypothetical protein	
2426	3581	NEOS13_2388	peg	3581_29140_28541	29140	28541	-	FIG00899512: hypothetical protein	
2427	3581	NEOS13_2389	peg	3581_29750_29151	29750	29151	-	FIG00899458: hypothetical protein	
2428	3581	NEOS13_2390	peg	3581_31792_30371	31792	30371	-	hypothetical protein	
2429	3581	NEOS13_2391	peg	3581_31921_32103	31921	32103	+	hypothetical protein	
2430	3581	NEOS13_2392	peg	3581_33062_32181	33062	32181	-	hypothetical protein	
2431	3581	NEOS13_2393	peg	3581_34499_33468	34499	33468	-	hypothetical protein	
2432	3581	NEOS13_2394	peg	3581_35686_34526	35686	34526	-	hypothetical protein	
2433	3581	NEOS13_2395	peg	3581_36252_35701	36252	35701	-	hypothetical protein	
2434	3581	NEOS13_2396	peg	3581_36734_38092	36734	38092	+	sodium:neurotransmitter symporter	
2435	3581	NEOS13_2397	peg	3581_38102_39103	38102	39103	+	Holliday junction DNA helicase RuvB	
2436	3581	NEOS13_2398	peg	3581_39298_39086	39298	39086	-	hypothetical protein	
2437	3581	NEOS13_2399	peg	3581_39273_39956	39273	39956	+	hypothetical protein	
2438	3581	NEOS13_2400	peg	3581_40210_41247	40210	41247	+	S-adenosylmethionine:tRNA ribosyltransferase-isomerase (EC 5.-.-.-)	
2439	3581	NEOS13_2401	peg	3581_41787_43853	41787	43853	+	hypothetical protein	
2440	3581	NEOS13_2402	peg	3581_43866_43991	43866	43991	+	hypothetical protein	
2441	3581	NEOS13_2403	peg	3581_44188_45870	44188	45870	+	Leucine-rich repeat containing protein	Leucine-rich repeat
2442	3581	NEOS13_2404	peg	3581_46333_47862	46333	47862	+	Thermostable carboxypeptidase 1 (EC 3.4.17.19)	
2443	3581	NEOS13_2405	peg	3581_48063_49529	48063	49529	+	Leucine-rich repeat containing protein	Leucine-rich repeat
2444	3581	NEOS13_0039rna	rna	3581_49917_50003	49917	50003	+	tRNA-Ser-TGA	RNA
2445	3581	NEOS13_2406	peg	3581_51389_50607	51389	50607	-	hypothetical protein	
2446	3582	NEOS13_2407	peg	3582_254_2638	254	2638	+	hypothetical protein	
2447	3582	NEOS13_2408	peg	3582_2651_3202	2651	3202	+	unknown protein	
2448	3582	NEOS13_2409	peg	3582_3703_4185	3703	4185	+	hypothetical protein	
2449	3582	NEOS13_2410	peg	3582_4235_4357	4235	4357	+	hypothetical protein	
2450	3582	NEOS13_2411	peg	3582_4553_4368	4553	4368	-	hypothetical protein	
2451	3582	NEOS13_2412	peg	3582_4629_4979	4629	4979	+	hypothetical protein	
2452	3583	NEOS13_2413	peg	3583_658_374	658	374	-	hypothetical protein	
2453	3583	NEOS13_2414	peg	3583_1410_1535	1410	1535	+	hypothetical protein	
2454	3583	NEOS13_2415	peg	3583_1600_1743	1600	1743	+	hypothetical protein	
2455	3583	NEOS13_2416	peg	3583_2758_2315	2758	2315	-	hypothetical protein	
2456	3583	NEOS13_2417	peg	3583_3085_4104	3085	4104	+	hypothetical protein	
2457	3583	NEOS13_2418	peg	3583_5688_4171	5688	4171	-	general stress protein	

2458	3583	NEOS13_2419	peg	3583_6289_6498	6289	6498	+	hypothetical protein	
2459	3583	NEOS13_2420	peg	3583_7173_7688	7173	7688	+	Ribonucleotide reductase transcriptional regulator NrdR	
2460	3583	NEOS13_2421	peg	3583_7716_8081	7716	8081	+	DnaK Suppressor	
2461	3583	NEOS13_2422	peg	3583_8084_8611	8084	8611	+	Lipoprotein signal peptidase (EC 3.4.23.36)	
2462	3583	NEOS13_2423	peg	3583_8621_9331	8621	9331	+	hypothetical protein	
2463	3583	NEOS13_2424	peg	3583_9522_10370	9522	10370	+	SAM dependent methyltransferase	
2464	3583	NEOS13_2425	peg	3583_10360_11184	10360	11184	+	macromolecule metabolism; macromolecule synthesis, modification; rna synthesis, modification , dna transcription	
2465	3583	NEOS13_2426	peg	3583_11190_11849	11190	11849	+	Similar to tRNA pseudouridine synthase C, group TruC1	
2466	3583	NEOS13_2427	peg	3583_13173_11947	13173	11947	-	hypothetical protein	
2467	3583	NEOS13_2428	peg	3583_13669_14358	13669	14358	+	hypothetical protein	
2468	3583	NEOS13_2429	peg	3583_15103_15702	15103	15702	+	Phosphoheptose isomerase (EC 5.3.1.-)	
2469	3583	NEOS13_2430	peg	3583_15748_16860	15748	16860	+	Tetraacyldisaccharide 4'-kinase (EC 2.7.1.130)	
2470	3583	NEOS13_2431	peg	3583_19399_17513	19399	17513	-	Signal peptidase I (EC 3.4.21.89)	
2471	3583	NEOS13_2432	peg	3583_19483_19671	19483	19671	+	hypothetical protein	
2472	3583	NEOS13_2433	peg	3583_20533_19778	20533	19778	-	hypothetical protein	
2473	3583	NEOS13_2434	peg	3583_20667_22169	20667	22169	+	Nicotinate phosphoribosyltransferase (EC 2.4.2.11)	
2474	3583	NEOS13_2435	peg	3583_22173_22814	22173	22814	+	Nicotinamidase (EC 3.5.1.19)	
2475	3583	NEOS13_2436	peg	3583_23209_23027	23209	23027	-	hypothetical protein	
2476	3583	NEOS13_2437	peg	3583_23286_25370	23286	25370	+	hypothetical protein	
2477	3583	NEOS13_2438	peg	3583_26053_27654	26053	27654	+	Carboxypeptidase-related protein	
2478	3583	NEOS13_2439	peg	3583_27859_27719	27859	27719	-	hypothetical protein	
2479	3583	NEOS13_2440	peg	3583_28188_27886	28188	27886	-	Leucine-rich repeat containing protein	Leucine-rich repeat
2480	3583	NEOS13_2441	peg	3583_28208_28321	28208	28321	+	hypothetical protein	
2481	3583	NEOS13_2442	peg	3583_30130_29057	30130	29057	-	Leucine-rich repeat containing protein	Leucine-rich repeat
2482	3584	NEOS13_2443	peg	3584_714_28	714	28	-	hypothetical protein	
2483	3584	NEOS13_2444	peg	3584_1802_759	1802	759	-	hypothetical protein	
2484	3584	NEOS13_2445	peg	3584_3077_2016	3077	2016	-	hypothetical protein	
2485	3584	NEOS13_2446	peg	3584_3343_3558	3343	3558	+	Mobile element protein	
2486	3584	NEOS13_2447	peg	3584_3795_4106	3795	4106	+	Mobile element protein	
2487	3584	NEOS13_2448	peg	3584_4110_4283	4110	4283	+	hypothetical protein	
2488	3584	NEOS13_2449	peg	3584_4276_4482	4276	4482	+	hypothetical protein	
2489	3584	NEOS13_2450	peg	3584_4708_5529	4708	5529	+	hypothetical protein	
2490	3584	NEOS13_2451	peg	3584_5593_5796	5593	5796	+	hypothetical protein	
2491	3584	NEOS13_2452	peg	3584_5813_6385	5813	6385	+	hypothetical protein	
2492	3584	NEOS13_2453	peg	3584_6370_6531	6370	6531	+	hypothetical protein	
2493	3584	NEOS13_2454	peg	3584_6588_7637	6588	7637	+	hypothetical protein	
2494	3584	NEOS13_2455	peg	3584_7682_8368	7682	8368	+	hypothetical protein	
2495	3585	NEOS13_2456	peg	3585_38_2110	38	2110	+	hypothetical protein	
2496	3585	NEOS13_2457	peg	3585_2284_3534	2284	3534	+	hypothetical protein	
2497	3586	NEOS13_2458	peg	3586_38_3577	38	3577	+	hypothetical protein	
2498	3586	NEOS13_2459	peg	3586_4631_3720	4631	3720	-	Probable protease htpX homolog (EC 3.4.24.-)	
2499	3586	NEOS13_2460	peg	3586_6290_5298	6290	5298	-	ROK family protein	Glycolysis_Glucogenesis
2500	3586	NEOS13_2461	peg	3586_6527_7813	6527	7813	+	5-Enolpyruvylshikimate-3-phosphate synthase (EC 2.5.1.19)	
2501	3586	NEOS13_2462	peg	3586_7880_8407	7880	8407	+	Shikimate kinase I (EC 2.7.1.71) # AroE I	
2502	3586	NEOS13_2463	peg	3586_8412_9485	8412	9485	+	Chorismate synthase (EC 4.2.3.5)	
2503	3586	NEOS13_2464	peg	3586_9482_10954	9482	10954	+	shikimate 5-dehydrogenase(EC:1.1.1.25)	
2504	3586	NEOS13_2465	peg	3586_11114_12244	11114	12244	+	Putrescine transport ATP-binding protein PotA (TC 3.A.1.11.1)	ABC_transporter
2505	3586	NEOS13_2466	peg	3586_12246_13115	12246	13115	+	Spermidine Putrescine ABC transporter permease component PotB (TC 3.A.1.11.1)	ABC_transporter
2506	3586	NEOS13_2467	peg	3586_13112_13876	13112	13876	+	Spermidine Putrescine ABC transporter permease component potC (TC_3.A.1.11.1)	ABC_transporter
2507	3586	NEOS13_2468	peg	3586_13878_14915	13878	14915	+	ABC transporter, periplasmic spermidine putrescine-binding protein PotD (TC 3.A.1.11.1)	ABC_transporter
2508	3586	NEOS13_2469	peg	3586_15331_15083	15331	15083	-	HicB protein	
2509	3586	NEOS13_2470	peg	3586_15428_15550	15428	15550	+	hypothetical protein	
2510	3586	NEOS13_2471	peg	3586_17600_15516	17600	15516	-	hypothetical protein	
2511	3586	NEOS13_2472	peg	3586_19382_18198	19382	18198	-	hypothetical protein	
2512	3586	NEOS13_2473	peg	3586_19679_19810	19679	19810	+	hypothetical protein	
2513	3586	NEOS13_2474	peg	3586_20248_19832	20248	19832	-	COG1720: Uncharacterized conserved protein	
2514	3586	NEOS13_2475	peg	3586_20311_20886	20311	20886	+	hypothetical protein	
2515	3586	NEOS13_2476	peg	3586_21076_22101	21076	22101	+	DNA polymerase III delta subunit (EC 2.7.7.7)	
2516	3586	NEOS13_2477	peg	3586_22113_22829	22113	22829	+	SAM-dependent methyltransferase	
2517	3586	NEOS13_2478	peg	3586_23988_22843	23988	22843	-	Hypothetical radical SAM family enzyme, NOT coproporphyrinogen III oxidase, oxygen-independent	
2518	3586	NEOS13_2479	peg	3586_24530_24039	24530	24039	-	FIG00494315: hypothetical protein	
2519	3586	NEOS13_2480	peg	3586_26729_24609	26729	24609	-	hypothetical protein	
2520	3586	NEOS13_2481	peg	3586_26936_28135	26936	28135	+	hypothetical protein	
2521	3586	NEOS13_2482	peg	3586_28411_29802	28411	29802	+	hypothetical protein	
2522	3586	NEOS13_2483	peg	3586_32686_30083	32686	30083	-	hypothetical protein	

2523	3586	NEOS13_2484	peg	3586_33336_32686	33336	32686	-	hypothetical protein	
2524	3586	NEOS13_2485	peg	3586_34045_33734	34045	33734	-	hypothetical protein	
2525	3586	NEOS13_2486	peg	3586_34425_34709	34425	34709	+	hypothetical protein	
2526	3587	NEOS13_2487	peg	3587_119_391	119	391	+	Transposase, IS4 family protein	Transposase
2527	3587	NEOS13_2488	peg	3587_1809_601	1809	601	-	hypothetical protein	
2528	3587	NEOS13_2489	peg	3587_1854_1976	1854	1976	+	hypothetical protein	
2529	3587	NEOS13_2490	peg	3587_2141_5239	2141	5239	+	hypothetical protein	
2530	3587	NEOS13_2491	peg	3587_7231_5885	7231	5885	-	Chromosomal replication initiator protein DnaA	
2531	3587	NEOS13_2492	peg	3587_7401_7547	7401	7547	+	hypothetical protein	
2532	3587	NEOS13_2493	peg	3587_8207_9385	8207	9385	+	Bacillosamine/Legionaminic acid biosynthesis aminotransferase PglE; 4-keto-6-deoxy-N-Acetyl-D-hexosaminyl-(Lipid carrier) aminotransferase	
2533	3587	NEOS13_2494	peg	3587_9797_9402	9797	9402	-	type III secretion chaperone, putative	
2534	3587	NEOS13_2495	peg	3587_10186_9800	10186	9800	-	hypothetical protein	
2535	3587	NEOS13_2496	peg	3587_10518_11450	10518	11450	+	rRNA small subunit methyltransferase H	
2536	3587	NEOS13_2497	peg	3587_11447_11797	11447	11797	+	hypothetical protein	
2537	3587	NEOS13_2498	peg	3587_11854_13839	11854	13839	+	Cell division protein FtsI [Peptidoglycan synthetase] (EC 2.4.1.129)	
2538	3587	NEOS13_2499	peg	3587_13878_15377	13878	15377	+	UDP-N-acetylmuramoylalanyl-D-glutamate--2,6-diaminopimelate ligase (EC 6.3.2.13)	
2539	3587	NEOS13_2500	peg	3587_15382_16149	15382	16149	+	N-acetylmuramoyl-L-alanine amidase (EC 3.5.1.28)	
2540	3587	NEOS13_0040ma	rna	3587_16271_16344	16271	16344	+	tRNA-Arg-CCT	RNA
2541	3587	NEOS13_2501	peg	3587_16432_17883	16432	17883	+	6-phosphogluconate dehydrogenase, decarboxylating (EC 1.1.1.44)	Pentose_phospate_pathway
2542	3587	NEOS13_2502	peg	3587_19325_18441	19325	18441	-	hypothetical protein	
2543	3587	NEOS13_2503	peg	3587_20034_19444	20034	19444	-	1-acyl-sn-glycerol-3-phosphate acyltransferase (EC 2.3.1.51)	
2544	3587	NEOS13_2504	peg	3587_20582_20343	20582	20343	-	hypothetical protein	
2545	3588	NEOS13_2505	peg	3588_2110_284	2110	284	-	Leucine-rich repeat containing protein	Leucine-rich repeat
2546	3588	NEOS13_2506	peg	3588_3288_2698	3288	2698	-	Leucine-rich repeat containing protein	Leucine-rich repeat
2547	3588	NEOS13_2507	peg	3588_4644_3529	4644	3529	-	Leucine-rich repeat containing protein	Leucine-rich repeat
2548	3588	NEOS13_2508	peg	3588_6614_4923	6614	4923	-	Leucine-rich repeat containing protein	Leucine-rich repeat
2549	3588	NEOS13_2509	peg	3588_7519_6965	7519	6965	-	Leucine-rich repeat containing protein	Leucine-rich repeat
2550	3589	NEOS13_2510	peg	3589_516_388	516	388	-	hypothetical protein	
2551	3589	NEOS13_2511	peg	3589_882_1100	882	1100	+	hypothetical protein	
2552	3589	NEOS13_2512	peg	3589_1975_1397	1975	1397	-	protein of unknown function DUF892	
2553	3589	NEOS13_2513	peg	3589_2319_2447	2319	2447	+	hypothetical protein	
2554	3589	NEOS13_2514	peg	3589_2673_3104	2673	3104	+	hypothetical protein	
2555	3589	NEOS13_2515	peg	3589_3712_4248	3712	4248	+	hypothetical protein	
2556	3589	NEOS13_2516	peg	3589_4445_4948	4445	4948	+	hypothetical protein	
2557	3589	NEOS13_2517	peg	3589_5028_5906	5028	5906	+	Uncharacterized protein conserved in bacteria, NMA0228-like	
2558	3589	NEOS13_2518	peg	3589_5903_6802	5903	6802	+	hypothetical protein	
2559	3589	NEOS13_2519	peg	3589_6786_8027	6786	8027	+	Ribosomal RNA small subunit methyltransferase B (EC 2.1.1.-)	
2560	3589	NEOS13_2520	peg	3589_8909_8244	8909	8244	-	hypothetical protein	
2561	3589	NEOS13_2521	peg	3589_9361_8918	9361	8918	-	hypothetical protein	
2562	3589	NEOS13_2522	peg	3589_14630_9621	14630	9621	-	hypothetical protein	
2563	3589	NEOS13_2523	peg	3589_14706_14828	14706	14828	+	hypothetical protein	
2564	3589	NEOS13_2524	peg	3589_15858_15244	15858	15244	-	putative transcriptional regulator	
2565	3589	NEOS13_2525	peg	3589_16723_15908	16723	15908	-	putative transcriptional regulator	
2566	3589	NEOS13_2526	peg	3589_16926_16723	16926	16723	-	hypothetical protein	
2567	3589	NEOS13_2527	peg	3589_17541_18368	17541	18368	+	Mobile element protein	
2568	3589	NEOS13_2528	peg	3589_20015_18852	20015	18852	-	Leucine-rich repeat containing protein	Leucine-rich repeat
2569	3589	NEOS13_2529	peg	3589_22190_20865	22190	20865	-	Leucine-rich repeat containing protein	Leucine-rich repeat
2570	3589	NEOS13_2530	peg	3589_24914_22665	24914	22665	-	Leucine-rich repeat containing protein	Leucine-rich repeat
2571	3589	NEOS13_2531	peg	3589_25255_25109	25255	25109	-	hypothetical protein	
2572	3589	NEOS13_2532	peg	3589_25453_25301	25453	25301	-	hypothetical protein	
2573	3589	NEOS13_2533	peg	3589_29171_25422	29171	25422	-	hypothetical protein	
2574	3589	NEOS13_2534	peg	3589_29364_29173	29364	29173	-	hypothetical protein	
2575	3589	NEOS13_2535	peg	3589_29874_29473	29874	29473	-	conserved hypothetical protein, partial length	
2576	3589	NEOS13_2536	peg	3589_30831_29875	30831	29875	-	hypothetical protein	
2577	3589	NEOS13_2537	peg	3589_33730_30809	33730	30809	-	Type III secretion protein SctC	
2578	3589	NEOS13_2538	peg	3589_34247_34363	34247	34363	+	hypothetical protein	
2579	3589	NEOS13_2539	peg	3589_34781_34665	34781	34665	-	Mobile element protein	
2580	3589	NEOS13_2540	peg	3589_35124_35258	35124	35258	+	hypothetical protein	
2581	3589	NEOS13_2541	peg	3589_35313_35537	35313	35537	+	hypothetical protein	
2582	3590	NEOS13_2542	peg	3590_1317_574	1317	574	-	Segregation and condensation protein B	
2583	3590	NEOS13_2543	peg	3590_2052_1324	2052	1324	-	Segregation and condensation protein A	
2584	3590	NEOS13_2544	peg	3590_2280_4376	2280	4376	+	Peptidase, S41 family	
2585	3590	NEOS13_0041ma	rna	3590_4442_4514	4442	4514	+	tRNA-Phe-GAA	RNA
2586	3590	NEOS13_2545	peg	3590_7593_5329	7593	5329	-	hypothetical protein	
2587	3590	NEOS13_2546	peg	3590_7786_7908	7786	7908	+	hypothetical protein	
2588	3590	NEOS13_2547	peg	3590_8081_8326	8081	8326	+	50S ribosomal protein L21	
2589	3590	NEOS13_2548	peg	3590_8417_8590	8417	8590	+	LSU ribosomal protein L27p	
2590	3590	NEOS13_2549	peg	3590_8694_9695	8694	9695	+	COG0536: GTP-binding protein Obg	
2591	3590	NEOS13_2550	peg	3590_10671_9811	10671	9811	-	dTDP-4-dehydrorhamnose reductase (EC 1.1.1.133)	

2592	3590	NEOS13_2551	peg	3590_11216_10668	11216	10668	-	dTDP-4-dehydrorhamnose 3,5-epimerase (EC 5.1.3.13)	
2593	3590	NEOS13_2552	peg	3590_11901_13004	11901	13004	+	Acetate kinase (EC 2.7.2.1)	
2594	3590	NEOS13_2553	peg	3590_13309_13187	13309	13187	-	hypothetical protein	
2595	3590	NEOS13_2554	peg	3590_13451_13329	13451	13329	-	hypothetical protein	
2596	3590	NEOS13_2555	peg	3590_13652_15121	13652	15121	+	Leucine-rich repeat containing protein	Leucine-rich repeat
2597	3590	NEOS13_2556	peg	3590_15276_15404	15276	15404	+	hypothetical protein	
2598	3590	NEOS13_0042rna	rna	3590_15552_15639	15552	15639	+	tRNA-Ser-GCT	RNA
2599	3590	NEOS13_2557	peg	3590_16571_15696	16571	15696	-	Zinc ABC transporter, inner membrane permease protein ZnuB	ABC_transporter
2600	3590	NEOS13_2558	peg	3590_17296_16568	17296	16568	-	Zinc ABC transporter, ATP-binding protein ZnuC	ABC_transporter
2601	3590	NEOS13_2559	peg	3590_17765_17577	17765	17577	-	hypothetical protein	
2602	3590	NEOS13_2560	peg	3590_18696_17806	18696	17806	-	Zinc ABC transporter, periplasmic-binding protein ZnuA	ABC_transporter
2603	3590	NEOS13_0043rna	rna	3590_18930_19018	18930	19018	+	tRNA-Ser-GGA	RNA
2604	3590	NEOS13_2561	peg	3590_19322_19188	19322	19188	-	hypothetical protein	
2605	3590	NEOS13_2562	peg	3590_20081_19566	20081	19566	-	hypothetical protein	
2606	3590	NEOS13_2563	peg	3590_20761_20393	20761	20393	-	hypothetical protein	
2607	3590	NEOS13_2564	peg	3590_21881_20895	21881	20895	-	hypothetical protein	
2608	3590	NEOS13_2565	peg	3590_22321_22434	22321	22434	+	hypothetical protein	
2609	3590	NEOS13_2566	peg	3590_22586_23560	22586	23560	+	Ribonucleotide reductase of class Ia (aerobic), beta subunit (EC 1.17.4.1)	
2610	3590	NEOS13_2567	peg	3590_23571_25889	23571	25889	+	Ribonucleotide reductase of class Ia (aerobic), alpha subunit (EC 1.17.4.1)	
2611	3590	NEOS13_2568	peg	3590_26400_27659	26400	27659	+	Gamma-glutamyl phosphate reductase (EC 1.2.1.41)	
2612	3590	NEOS13_2569	peg	3590_27864_29744	27864	29744	+	hypothetical protein	
2613	3590	NEOS13_2570	peg	3590_30145_31119	30145	31119	+	hypothetical protein	
2614	3590	NEOS13_2571	peg	3590_31129_31293	31129	31293	+	hypothetical protein	
2615	3590	NEOS13_2572	peg	3590_32000_31773	32000	31773	-	hypothetical protein	
2616	3590	NEOS13_2573	peg	3590_32390_32031	32390	32031	-	hypothetical protein	
2617	3590	NEOS13_2574	peg	3590_34039_33071	34039	33071	-	putative 1-aminocyclopropane-1-carboxylate deaminase (ACC deaminase)	
2618	3590	NEOS13_2575	peg	3590_34206_34069	34206	34069	-	hypothetical protein	
2619	3590	NEOS13_2576	peg	3590_34284_34415	34284	34415	+	hypothetical protein	
2620	3590	NEOS13_2577	peg	3590_34498_35742	34498	35742	+	NAD(FAD)-utilizing dehydrogenases	
2621	3590	NEOS13_2578	peg	3590_36678_35872	36678	35872	-	Coproporphyrinogen III oxidase, aerobic (EC 1.3.3.3)	
2622	3590	NEOS13_2579	peg	3590_36919_36728	36919	36728	-	hypothetical protein	
2623	3590	NEOS13_2580	peg	3590_36944_37063	36944	37063	+	hypothetical protein	
2624	3590	NEOS13_2581	peg	3590_38856_37303	38856	37303	-	Leucine-rich repeat containing protein	Leucine-rich repeat
2625	3591	NEOS13_2582	peg	3591_1919_579	1919	579	-	Leucine-rich repeat containing protein	Leucine-rich repeat
2626	3591	NEOS13_2583	peg	3591_2144_2028	2144	2028	-	hypothetical protein	
2627	3591	NEOS13_2584	peg	3591_2307_2176	2307	2176	-	hypothetical protein	
2628	3591	NEOS13_2585	peg	3591_2911_2795	2911	2795	-	hypothetical protein	
2629	3591	NEOS13_2586	peg	3591_3053_3208	3053	3208	+	Mobile element protein	
2630	3591	NEOS13_2587	peg	3591_3425_3285	3425	3285	-	hypothetical protein	
2631	3591	NEOS13_2588	peg	3591_5885_4089	5885	4089	-	hypothetical protein	
2632	3591	NEOS13_2589	peg	3591_6256_6417	6256	6417	+	hypothetical protein	
2633	3591	NEOS13_2590	peg	3591_6669_10091	6669	10091	+	Exodeoxyribonuclease V gamma chain (EC 3.1.11.5)	
2634	3591	NEOS13_2591	peg	3591_10251_11072	10251	11072	+	hypothetical protein	
2635	3591	NEOS13_2592	peg	3591_12211_15717	12211	15717	+	Exodeoxyribonuclease V beta chain (EC 3.1.11.5)	
2636	3591	NEOS13_2593	peg	3591_15774_17522	15774	17522	+	Exodeoxyribonuclease V alpha chain (EC 3.1.11.5)	
2637	3591	NEOS13_2594	peg	3591_18356_17967	18356	17967	-	hypothetical protein	
2638	3591	NEOS13_2595	peg	3591_19603_18434	19603	18434	-	hypothetical protein	
2639	3591	NEOS13_2596	peg	3591_20950_20465	20950	20465	-	hypothetical protein	
2640	3591	NEOS13_2597	peg	3591_21108_21245	21108	21245	+	hypothetical protein	
2641	3591	NEOS13_2598	peg	3591_21262_23427	21262	23427	+	Transcription elongation factor GreA	
2642	3591	NEOS13_2599	peg	3591_23428_24036	23428	24036	+	Nucleoside 5-triphosphatase RdgB (dHAPTP, dITP, XTP-specific) (EC 3.6.1.15)	
2643	3591	NEOS13_2600	peg	3591_24045_24740	24045	24740	+	hypothetical protein	
2644	3591	NEOS13_2601	peg	3591_24950_25933	24950	25933	+	hypothetical protein	
2645	3591	NEOS13_0044rna	rna	3591_26078_26150	26078	26150	+	tRNA-Ala-GGC	RNA
2646	3591	NEOS13_2602	peg	3591_26230_26379	26230	26379	+	hypothetical protein	
2647	3591	NEOS13_2603	peg	3591_26854_26621	26854	26621	-	hypothetical protein	
2648	3591	NEOS13_2604	peg	3591_27723_26995	27723	26995	-	hypothetical protein	
2649	3591	NEOS13_2605	peg	3591_28517_27786	28517	27786	-	hypothetical protein	
2650	3591	NEOS13_2606	peg	3591_28517_28633	28517	28633	+	hypothetical protein	
2651	3591	NEOS13_2607	peg	3591_29877_28918	29877	28918	-	hypothetical protein	
2652	3591	NEOS13_2608	peg	3591_31738_30296	31738	30296	-	hypothetical protein	
2653	3591	NEOS13_2609	peg	3591_35567_32628	35567	32628	-	hypothetical protein	
2654	3591	NEOS13_2610	peg	3591_36586_35765	36586	35765	-	hypothetical protein	
2655	3591	NEOS13_2611	peg	3591_36909_38846	36909	38846	+	Threonyl-tRNA synthetase (EC 6.1.1.3)	
2656	3591	NEOS13_2612	peg	3591_38834_39586	38834	39586	+	Septum site-determining protein MinD	
2657	3591	NEOS13_2613	peg	3591_39723_40469	39723	40469	+	Virulence plasmid protein pGP6-D	
2658	3591	NEOS13_2614	peg	3591_41058_40942	41058	40942	-	hypothetical protein	
2659	3591	NEOS13_2615	peg	3591_41106_41252	41106	41252	+	hypothetical protein	
2660	3591	NEOS13_2616	peg	3591_41381_42088	41381	42088	+	hypothetical protein	
2661	3591	NEOS13_2617	peg	3591_42070_43437	42070	43437	+	Leucine-rich repeat containing protein	Leucine-rich repeat

2662	3591	NEOS13_2618	peg	3591_43972_43844	43972	43844	-	hypothetical protein	
2663	3591	NEOS13_2619	peg	3591_43982_45325	43982	45325	+	Leucine-rich repeat containing protein	Leucine-rich repeat
2664	3592	NEOS13_2620	peg	3592_751_137	751	137	-	hypothetical protein	
2665	3592	NEOS13_2621	peg	3592_1029_1286	1029	1286	+	hypothetical protein	
2666	3592	NEOS13_2622	peg	3592_1675_1490	1675	1490	-	hypothetical protein	
2667	3592	NEOS13_2623	peg	3592_2441_2142	2441	2142	-	hypothetical protein	
2668	3592	NEOS13_2624	peg	3592_2935_3129	2935	3129	+	hypothetical protein	
2669	3592	NEOS13_2625	peg	3592_4358_3231	4358	3231	-	Branched-chain amino acid transport system carrier protein	
2670	3592	NEOS13_2626	peg	3592_6196_5012	6196	5012	-	L,L-diaminopimelate aminotransferase (EC 2.6.1.83)	
2671	3592	NEOS13_2627	peg	3592_7277_6201	7277	6201	-	Aspartate-semialdehyde dehydrogenase (EC 1.2.1.11)	
2672	3592	NEOS13_2628	peg	3592_7613_7735	7613	7735	+	hypothetical protein	
2673	3592	NEOS13_2629	peg	3592_8162_9409	8162	9409	+	putative oxidoreductase iron/ascorbatefamily protein	
2674	3592	NEOS13_2630	peg	3592_11752_9632	11752	9632	-	FIG00494344: hypothetical protein	
2675	3592	NEOS13_2631	peg	3592_12298_13173	12298	13173	+	FIG00493905: hypothetical protein	
2676	3592	NEOS13_2632	peg	3592_13185_13523	13185	13523	+	HIT family protein	
2677	3592	NEOS13_2633	peg	3592_14073_15809	14073	15809	+	hypothetical protein	
2678	3592	NEOS13_2634	peg	3592_15916_17556	15916	17556	+	hypothetical protein	
2679	3592	NEOS13_2635	peg	3592_18092_17823	18092	17823	-	Thioredoxin	
2680	3592	NEOS13_2636	peg	3592_19038_18925	19038	18925	-	hypothetical protein	
2681	3592	NEOS13_2637	peg	3592_19675_21135	19675	21135	+	hypothetical protein	
2682	3592	NEOS13_2638	peg	3592_21397_21254	21397	21254	-	hypothetical protein	
2683	3592	NEOS13_2639	peg	3592_21854_23854	21854	23854	+	hypothetical protein	
2684	3592	NEOS13_2640	peg	3592_25311_23851	25311	23851	-	hypothetical protein	
2685	3592	NEOS13_2641	peg	3592_26175_25945	26175	25945	-	hypothetical protein	
2686	3592	NEOS13_2642	peg	3592_26896_26354	26896	26354	-	putative ATP-binding cassette protein, nasD	
2687	3592	NEOS13_2643	peg	3592_27449_28717	27449	28717	+	Tyrosine-specific transport protein	
2688	3592	NEOS13_2644	peg	3592_28729_29217	28729	29217	+	hypothetical protein	
2689	3592	NEOS13_2645	peg	3592_29268_31091	29268	31091	+	DnaK-related protein	
2690	3592	NEOS13_2646	peg	3592_31185_31298	31185	31298	+	hypothetical protein	
2691	3592	NEOS13_2647	peg	3592_31751_31326	31751	31326	-	Sulfur acceptor protein SufE for iron-sulfur cluster assembly	
2692	3592	NEOS13_2648	peg	3592_32520_32738	32520	32738	+	Translation initiation factor 1	
2693	3592	NEOS13_0045ma	rna	3592_32826_32897	32826	32897	+	tRNA-Thr-GGT	RNA
2694	3592	NEOS13_2649	peg	3592_33350_34159	33350	34159	+	Translation elongation factor Tu	
2695	3592	NEOS13_0046ma	rna	3592_34230_34302	34230	34302	+	tRNA-Trp-CCA	RNA
2696	3592	NEOS13_2650	peg	3592_34343_34636	34343	34636	+	putative preprotein translocase SecE	
2697	3592	NEOS13_2651	peg	3592_34658_35206	34658	35206	+	Transcription antitermination protein NusG	
2698	3592	NEOS13_2652	peg	3592_35368_35796	35368	35796	+	LSU ribosomal protein L11p (L12e)	
2699	3592	NEOS13_2653	peg	3592_35818_36516	35818	36516	+	LSU ribosomal protein L1p (L10Ae)	
2700	3592	NEOS13_2654	peg	3592_36529_37056	36529	37056	+	LSU ribosomal protein L10p (P0)	
2701	3592	NEOS13_2655	peg	3592_37098_37481	37098	37481	+	LSU ribosomal protein L7/L12 (P1/P2)	
2702	3592	NEOS13_2656	peg	3592_38082_41504	38082	41504	+	DNA-directed RNA polymerase beta subunit (EC 2.7.7.6)	
2703	3592	NEOS13_2657	peg	3592_41526_45725	41526	45725	+	DNA-directed RNA polymerase beta' subunit (EC 2.7.7.6)	
2704	3592	NEOS13_2658	peg	3592_47389_46418	47389	46418	-	Transaldolase (EC 2.2.1.2)	Pentose_phospate_pathway
2705	3592	NEOS13_2659	peg	3592_47768_47382	47768	47382	-	hypothetical protein	
2706	3592	NEOS13_2660	peg	3592_48350_48192	48350	48192	-	hypothetical protein	
2707	3592	NEOS13_2661	peg	3592_48591_48478	48591	48478	-	hypothetical protein	
2708	3592	NEOS13_2662	peg	3592_48698_48558	48698	48558	-	hypothetical protein	
2709	3592	NEOS13_2663	peg	3592_49326_48823	49326	48823	-	hypothetical protein	
2710	3592	NEOS13_2664	peg	3592_49574_49338	49574	49338	-	hypothetical protein	
2711	3592	NEOS13_2665	peg	3592_50251_49775	50251	49775	-	hypothetical protein	
2712	3592	NEOS13_2666	peg	3592_50454_50606	50454	50606	+	hypothetical protein	
2713	3592	NEOS13_2667	peg	3592_50793_51419	50793	51419	+	V-type ATP synthase subunit E (EC 3.6.3.14)	Oxidative_Phosphorylation
2714	3592	NEOS13_2668	peg	3592_51425_52210	51425	52210	+	hypothetical protein	
2715	3592	NEOS13_2669	peg	3592_52207_53991	52207	53991	+	V-type ATP synthase subunit A (EC 3.6.3.14)	Oxidative_Phosphorylation
2716	3592	NEOS13_2670	peg	3592_54005_55324	54005	55324	+	V-type ATP synthase subunit B (EC 3.6.3.14)	Oxidative_Phosphorylation
2717	3592	NEOS13_2671	peg	3592_55495_56142	55495	56142	+	V-type ATP synthase subunit D (EC 3.6.3.14)	Oxidative_Phosphorylation
2718	3592	NEOS13_2672	peg	3592_56139_58064	56139	58064	+	V-type ATP synthase subunit I (EC 3.6.3.14)	Oxidative_Phosphorylation
2719	3592	NEOS13_2673	peg	3592_58095_58517	58095	58517	+	V-type ATP synthase subunit K (EC 3.6.3.14)	Oxidative_Phosphorylation
2720	3592	NEOS13_2674	peg	3592_58905_59030	58905	59030	+	hypothetical protein	
2721	3592	NEOS13_2675	peg	3592_59355_60584	59355	60584	+	hypothetical protein	
2722	3592	NEOS13_2676	peg	3592_61029_61319	61029	61319	+	hypothetical protein	
2723	3592	NEOS13_2677	peg	3592_61678_61559	61678	61559	-	hypothetical protein	
2724	3592	NEOS13_2678	peg	3592_63568_62096	63568	62096	-	Sodium-dependent transporter	
2725	3592	NEOS13_2679	peg	3592_64007_63846	64007	63846	-	hypothetical protein	
2726	3592	NEOS13_2680	peg	3592_64060_64698	64060	64698	+	hypothetical protein	
2727	3592	NEOS13_2681	peg	3592_65021_65134	65021	65134	+	hypothetical protein	
2728	3592	NEOS13_0047ma	rna	3592_65248_65162	65248	65162	-	tRNA-Ser-CGA	RNA
2729	3592	NEOS13_2682	peg	3592_65395_65832	65395	65832	+	Mobile element protein	
2730	3592	NEOS13_2683	peg	3592_66497_66610	66497	66610	+	hypothetical protein	
2731	3592	NEOS13_2684	peg	3592_66649_66861	66649	66861	+	hypothetical protein	

2732	3592	NEOS13_2685	peg	3592_66864_67373	66864	67373	+	Protein yciF	
2733	3592	NEOS13_2686	peg	3592_67886_68458	67886	68458	+	ATP-dependent DNA ligase (EC 6.5.1.1) clustered with Ku protein, LigD	
2734	3592	NEOS13_2687	peg	3592_69100_68960	69100	68960	-	hypothetical protein	
2735	3592	NEOS13_2688	peg	3592_70337_70546	70337	70546	+	hypothetical protein	
2736	3593	NEOS13_2689	peg	3593_22_225	22	225	+	hypothetical protein	
2737	3593	NEOS13_2690	peg	3593_719_925	719	925	+	hypothetical protein	
2738	3593	NEOS13_2691	peg	3593_1071_1445	1071	1445	+	hypothetical protein	
2739	3593	NEOS13_2692	peg	3593_1695_1967	1695	1967	+	hypothetical protein	
2740	3593	NEOS13_2693	peg	3593_2080_2490	2080	2490	+	hypothetical protein	
2741	3593	NEOS13_2694	peg	3593_2635_3009	2635	3009	+	hypothetical protein	
2742	3593	NEOS13_2695	peg	3593_3157_3531	3157	3531	+	hypothetical protein	
2743	3593	NEOS13_2696	peg	3593_3851_4087	3851	4087	+	hypothetical protein	
2744	3593	NEOS13_2697	peg	3593_4650_4922	4650	4922	+	hypothetical protein	
2745	3593	NEOS13_2698	peg	3593_5241_5456	5241	5456	+	hypothetical protein	
2746	3593	NEOS13_2699	peg	3593_7096_5750	7096	5750	-	hypothetical protein	
2747	3593	NEOS13_2700	peg	3593_7120_7233	7120	7233	+	hypothetical protein	
2748	3593	NEOS13_2701	peg	3593_7372_7491	7372	7491	+	hypothetical protein	
2749	3593	NEOS13_2702	peg	3593_9469_8078	9469	8078	-	Na(+)-translocating NADH-quinone reductase subunit A (EC 1.6.5.-)	Oxidative_Phosphorylation
2750	3593	NEOS13_2703	peg	3593_10201_9794	10201	9794	-	hypothetical protein	
2751	3593	NEOS13_2704	peg	3593_10568_11974	10568	11974	+	hypothetical protein	
2752	3593	NEOS13_2705	peg	3593_11971_14550	11971	14550	+	General secretion pathway protein D	Sec_T2SS
2753	3593	NEOS13_2706	peg	3593_14537_16165	14537	16165	+	General secretion pathway protein E / Type II secretory pathway, ATPase PulE/Tfp pilus assembly pathway, ATPase PilB	Sec_T2SS
2754	3593	NEOS13_2707	peg	3593_16172_17356	16172	17356	+	general secretion pathway protein F	Sec_T2SS
2755	3593	NEOS13_2708	peg	3593_17377_17766	17377	17766	+	hypothetical protein	
2756	3593	NEOS13_2709	peg	3593_17807_18397	17807	18397	+	hypothetical protein	
2757	3593	NEOS13_2710	peg	3593_22943_18696	22943	18696	-	hypothetical protein	
2758	3593	NEOS13_2711	peg	3593_26380_23027	26380	23027	-	hypothetical protein	
2759	3593	NEOS13_2712	peg	3593_27181_27327	27181	27327	+	hypothetical protein	
2760	3593	NEOS13_2713	peg	3593_27497_28051	27497	28051	+	hypothetical protein	
2761	3593	NEOS13_2714	peg	3593_34565_28296	34565	28296	-	hypothetical protein	
2762	3593	NEOS13_2715	peg	3593_34591_34773	34591	34773	+	hypothetical protein	
2763	3593	NEOS13_2716	peg	3593_36329_35298	36329	35298	-	Oxidoreductase	
2764	3593	NEOS13_2717	peg	3593_36710_37312	36710	37312	+	hypothetical protein	
2765	3593	NEOS13_2718	peg	3593_37357_38034	37357	38034	+	hypothetical protein	
2766	3593	NEOS13_2719	peg	3593_38051_39001	38051	39001	+	hypothetical protein	
2767	3593	NEOS13_2720	peg	3593_38998_39981	38998	39981	+	hypothetical protein	
2768	3593	NEOS13_2721	peg	3593_40247_40651	40247	40651	+	hypothetical protein	
2769	3593	NEOS13_2722	peg	3593_40954_42330	40954	42330	+	probable two-component response regulator	
2770	3593	NEOS13_2723	peg	3593_42816_43721	42816	43721	+	hypothetical protein	
2771	3593	NEOS13_2724	peg	3593_43865_43713	43865	43713	-	hypothetical protein	
2772	3593	NEOS13_2725	peg	3593_45881_43971	45881	43971	-	Leucine-rich repeat containing protein	Leucine-rich repeat
2773	3593	NEOS13_2726	peg	3593_46244_45924	46244	45924	-	Leucine-rich repeat containing protein	Leucine-rich repeat
2774	3594	NEOS13_2727	peg	3594_128_370	128	370	+	hypothetical protein	
2775	3595	NEOS13_2728	peg	3595_110_508	110	508	+	Transposase	Transposase
2776	3595	NEOS13_2729	peg	3595_501_782	501	782	+	transposase	Transposase
2777	3595	NEOS13_2730	peg	3595_814_978	814	978	+	hypothetical protein	
2778	3595	NEOS13_2731	peg	3595_2078_1005	2078	1005	-	Mobile element protein	
2779	3596	NEOS13_2732	peg	3596_293_550	293	550	+	transposase	Transposase
2780	3596	NEOS13_2733	peg	3596_760_1359	760	1359	+	LemA protein	
2781	3596	NEOS13_2734	peg	3596_1434_2423	1434	2423	+	Heat shock protein HtpX (EC 3.4.24.-)	
2782	3596	NEOS13_2735	peg	3596_2942_2826	2942	2826	-	hypothetical protein	
2783	3596	NEOS13_2736	peg	3596_3620_3162	3620	3162	-	DoxX	
2784	3596	NEOS13_2737	peg	3596_4117_4308	4117	4308	+	hypothetical protein	
2785	3596	NEOS13_2738	peg	3596_4475_6853	4475	6853	+	hypothetical protein	
2786	3596	NEOS13_2739	peg	3596_7066_8010	7066	8010	+	hypothetical protein	
2787	3596	NEOS13_2740	peg	3596_8594_8100	8594	8100	-	hypothetical protein	
2788	3596	NEOS13_2741	peg	3596_9571_8627	9571	8627	-	Cobalt-zinc-cadmium resistance protein	
2789	3596	NEOS13_2742	peg	3596_10269_14324	10269	14324	+	hypothetical protein	
2790	3596	NEOS13_2743	peg	3596_14616_14732	14616	14732	+	hypothetical protein	
2791	3596	NEOS13_2744	peg	3596_18258_14830	18258	14830	-	hypothetical protein	
2792	3596	NEOS13_2745	peg	3596_20591_18321	20591	18321	-	hypothetical protein	
2793	3596	NEOS13_2746	peg	3596_21088_20867	21088	20867	-	hypothetical protein	
2794	3596	NEOS13_2747	peg	3596_21693_21340	21693	21340	-	RNA-binding protein	
2795	3596	NEOS13_2748	peg	3596_22456_22100	22456	22100	-	RNA-binding protein	
2796	3596	NEOS13_2749	peg	3596_23291_22842	23291	22842	-	hypothetical protein	
2797	3596	NEOS13_2750	peg	3596_23299_23412	23299	23412	+	hypothetical protein	
2798	3596	NEOS13_2751	peg	3596_24186_23542	24186	23542	-	hypothetical protein	
2799	3596	NEOS13_2752	peg	3596_24496_26091	24496	26091	+	Oligopeptide ABC transporter, periplasmic oligopeptide-binding protein OppA (TC 3.A.1.5.1)	ABC_transporter
2800	3596	NEOS13_2753	peg	3596_26229_26062	26229	26062	-	hypothetical protein	
2801	3596	NEOS13_2754	peg	3596_28069_26417	28069	26417	-	Pyrophosphate--fructose 6-phosphate 1-phosphotransferase, beta subunit (EC 2.7.1.90)	
2802	3596	NEOS13_2755	peg	3596_28899_28105	28899	28105	-	hypothetical protein	
2803	3596	NEOS13_2756	peg	3596_29306_29184	29306	29184	-	hypothetical protein	
2804	3596	NEOS13_2757	peg	3596_29829_29653	29829	29653	-	hypothetical protein	
2805	3596	NEOS13_2758	peg	3596_31267_29846	31267	29846	-	hypothetical protein	
2806	3596	NEOS13_2759	peg	3596_32256_31972	32256	31972	-	hypothetical protein	
2807	3596	NEOS13_2760	peg	3596_32351_32878	32351	32878	+	hypothetical protein	
2808	3596	NEOS13_2761	peg	3596_32954_33082	32954	33082	+	hypothetical protein	

2809	3596	NEOS13_2762	peg	3596_33364_34218	33364	34218	+	Formamidopyrimidine-DNA glycosylase (EC 3.2.2.23)	
2810	3596	NEOS13_2763	peg	3596_34199_34807	34199	34807	+	HAD-superfamily hydrolase, subfamily IA, variant 3	
2811	3596	NEOS13_2764	peg	3596_35011_35550	35011	35550	+	hypothetical protein	
2812	3596	NEOS13_2765	peg	3596_35756_36943	35756	36943	+	2-amino-3-ketobutyrate coenzyme A ligase (EC 2.3.1.29)	
2813	3596	NEOS13_2766	peg	3596_36982_38016	36982	38016	+	L-threonine 3-dehydrogenase (EC 1.1.1.103)	
2814	3596	NEOS13_0048rna	rna	3596_39199_39127	39199	39127	-	tRNA-Met-CAT	RNA
2815	3596	NEOS13_0049rna	rna	3596_39278_39206	39278	39206	-	tRNA-Met-CAT	RNA
2816	3596	NEOS13_2767	peg	3596_40670_39378	40670	39378	-	3-deoxy-D-manno-octulosonic-acid transferase (EC 2.-.-.-)	
2817	3596	NEOS13_2768	peg	3596_41113_40994	41113	40994	-	hypothetical protein	
2818	3596	NEOS13_2769	peg	3596_43358_41130	43358	41130	-	Transcription accessory protein (S1 RNA-binding domain)	
2819	3596	NEOS13_2770	peg	3596_44164_43721	44164	43721	-	hypothetical protein	
2820	3596	NEOS13_2771	peg	3596_44309_44196	44309	44196	-	hypothetical protein	
2821	3596	NEOS13_2772	peg	3596_44409_44573	44409	44573	+	hypothetical protein	
2822	3596	NEOS13_2773	peg	3596_44692_45408	44692	45408	+	dienelactone hydrolase family protein	
2823	3596	NEOS13_2774	peg	3596_45414_45743	45414	45743	+	hypothetical protein	
2824	3596	NEOS13_2775	peg	3596_45977_46510	45977	46510	+	hypothetical protein	
2825	3596	NEOS13_2776	peg	3596_46577_47395	46577	47395	+	hypothetical protein	
2826	3596	NEOS13_2777	peg	3596_47421_47936	47421	47936	+	Peptidyl-prolyl cis-trans isomerase (EC 5.2.1.8)	
2827	3596	NEOS13_2778	peg	3596_47942_48097	47942	48097	+	hypothetical protein	
2828	3596	NEOS13_2779	peg	3596_48567_48439	48567	48439	-	hypothetical protein	
2829	3596	NEOS13_2780	peg	3596_51409_48587	51409	48587	-	Leucine-rich repeat containing protein	Leucine-rich repeat
2830	365	NEOS13_2781	peg	365_170_54	170	54	-	hypothetical protein	
2831	389	NEOS13_2782	peg	389_240_34	240	34	-	hypothetical protein	
2832	484	NEOS13_2783	peg	484_1281_148	1281	148	-	Leucine-rich repeat containing protein	Leucine-rich repeat
2833	484	NEOS13_2784	peg	484_1470_1625	1470	1625	+	hypothetical protein	
2834	484	NEOS13_2785	peg	484_2384_1662	2384	1662	-	putative biotin-[acetyl-CoA-carboxylase] ligase/biotin repressor (bifunctional)	
2835	484	NEOS13_2786	peg	484_2438_3574	2438	3574	+	Rod shape-determining protein RodA	
2836	484	NEOS13_2787	peg	484_3574_4023	3574	4023	+	hypothetical protein	
2837	484	NEOS13_2788	peg	484_4155_4313	4155	4313	+	hypothetical protein	
2838	484	NEOS13_2789	peg	484_5008_4652	5008	4652	-	SSU ribosomal protein S10p (S20e)	
2839	484	NEOS13_2790	peg	484_7109_5022	7109	5022	-	Translation elongation factor G	
2840	484	NEOS13_2791	peg	484_7601_7128	7601	7128	-	SSU ribosomal protein S7p (S5e)	
2841	484	NEOS13_2792	peg	484_8160_7789	8160	7789	-	SSU ribosomal protein S12p (S23e)	
2842	484	NEOS13_2793	peg	484_8259_9509	8259	9509	+	1-deoxy-D-xylulose 5-phosphate reductoisomerase (EC 1.1.1.267)	
2843	484	NEOS13_2794	peg	484_9972_11852	9972	11852	+	Membrane-associated zinc metalloprotease	
2844	529	NEOS13_2795	peg	529_205_32	205	32	-	hypothetical protein	
2845	571	NEOS13_2796	peg	571_3774_577	3774	577	-	ankyrin repeat protein	Ankyrin
2846	571	NEOS13_2797	peg	571_5313_3904	5313	3904	-	Protein RtcB	
2847	685	NEOS13_2798	peg	685_122_9	122	9	-	hypothetical protein	
2848	775	NEOS13_2799	peg	775_178_29	178	29	-	Leucine-rich repeat containing protein	Leucine-rich repeat
2849	865	NEOS13_2800	peg	865_365_204	365	204	-	hypothetical protein	
2850	866	NEOS13_2801	peg	866_270_61	270	61	-	Leucine-rich repeat containing protein	Leucine-rich repeat
2851	866	NEOS13_2802	peg	866_258_407	258	407	+	hypothetical protein	
2852	728	NEOS13_2803	peg	728_3_134	3	134	+	transposase	Transposase
2853	635	NEOS13_2804	peg	635_2_121	2	121	+	transposase	Transposase
2854	2642	NEOS13_2805	peg	2642_3_233	3	233	+	Leucine-rich repeat containing protein	Leucine-rich repeat
2855	1116	NEOS13_2806	peg	1116_1_219	1	219	-	Leucine-rich repeat containing protein	Leucine-rich repeat
2856	2733	NEOS13_2807	peg	2733_2_211	2	211	+	Leucine-rich repeat containing protein	Leucine-rich repeat
2857	1111	NEOS13_2808	peg	1111_1_210	1	210	-	Leucine-rich repeat containing protein	Leucine-rich repeat
2858	1798	NEOS13_2809	peg	1798_2_202	2	202	-	Leucine-rich repeat containing protein	Leucine-rich repeat
2859	1703	NEOS13_2810	peg	1703_2_193	2	193	-	Leucine-rich repeat containing protein	Leucine-rich repeat
2860	501	NEOS13_2811	peg	501_1_189	1	189	+	Leucine-rich repeat containing protein	Leucine-rich repeat
2861	1551	NEOS13_2812	peg	1551_1_183	1	183	-	Leucine-rich repeat containing protein	Leucine-rich repeat
2862	2456	NEOS13_2813	peg	2456_3_173	3	173	-	Leucine-rich repeat containing protein	Leucine-rich repeat
2863	2575	NEOS13_2814	peg	2575_3_170	3	170	-	Leucine-rich repeat containing protein	Leucine-rich repeat
2864	2486	NEOS13_2815	peg	2486_2_169	2	169	+	Leucine-rich repeat containing protein	Leucine-rich repeat
2865	2732	NEOS13_2816	peg	2732_2_166	2	166	+	Leucine-rich repeat containing protein	Leucine-rich repeat
2866	856	NEOS13_2817	peg	856_3_158	3	158	-	Leucine-rich repeat containing protein	Leucine-rich repeat
2867	3038	NEOS13_2818	peg	3038_3_149	3	149	-	Leucine-rich repeat containing protein	Leucine-rich repeat
2868	803	NEOS13_2819	peg	803_2_139	2	139	+	Leucine-rich repeat containing protein	Leucine-rich repeat
2869	1712	NEOS13_2820	peg	1712_3_134	3	134	+	Leucine-rich repeat containing protein	Leucine-rich repeat
2870	730	NEOS13_2821	peg	730_2_133	2	133	+	Leucine-rich repeat containing protein	Leucine-rich repeat
2871	1787	NEOS13_2822	peg	1787_3_131	3	131	-	Leucine-rich repeat containing protein	Leucine-rich repeat
2872	2417	NEOS13_2823	peg	2417_2_127	2	127	+	Leucine-rich repeat containing protein	Leucine-rich repeat
2873	1921	NEOS13_2824	peg	1921_1_126	1	126	-	Leucine-rich repeat containing protein	Leucine-rich repeat
2874	1178	NEOS13_2825	peg	1178_1_123	1	123	-	Leucine-rich repeat containing protein	Leucine-rich repeat
2875	2984	NEOS13_2826	peg	2984_1_123	1	123	-	Leucine-rich repeat containing protein	Leucine-rich repeat
2876	1631	NEOS13_2827	peg	1631_1_116	3	116	-	Leucine-rich repeat containing protein	Leucine-rich repeat
2877	1191	NEOS13_2828	peg	1191_3_113	3	113	-	Leucine-rich repeat containing protein	Leucine-rich repeat
2878	3484	NEOS13_2829	peg	3484_10062_10217	10062	10217	-	hypothetical protein	
2879	2884	NEOS13_2830	peg	2884_1_240	1	240	-	hypothetical protein	
2880	2195	NEOS13_2831	peg	2195_2_139	2	139	+	hypothetical protein	
2881	691	NEOS13_2832	peg	691_2_199	2	199	+	hypothetical protein	
