# Supplementary material for: Amoebal Endosymbiont Neochlamydia Genome Sequence Illuminates the Bacterial Role in the Defense of the Host Amoebae against Legionella pneumophila
Source: PLoS One. 2014 Apr 18;9(4):e95166. doi: 10.1371/journal.pone.0095166 (PMC3991601; doi:10.1371/journal.pone.0095166)
Supplement: Table S2 — Homologs of eukaryote genes in the Neochlamydia S13 genome encoding predicted LRR-molecules. (PDF) [file pone.0095166.s014.pdf]

Table S2. Homologs of eukaryote genes in *Neochlamydia* S13 genes encoding predicted LRR-molecules

Gene feature ID (RAST)	contig number	strand	nuc length	pep length	nr database BLASTP* (Accession number)	Length	Score	Identities	E value
NEOS13_2442	3583	-	303	100	ref|XP_002507353.1| u-box domain/leucine-rich repeat protein [Micromonas sp. RCC299]gb|ACO68611.1| u-box domain/leucine-rich repeat protein [Micromonas sp. RCC299]	426	71.2 bits (173)	45/99 (45%)	4E-11
NEOS13_0718	3407	+	174	57	|No hits found|				
NEOS13_0110	3329	+	222	73	ref|XP_002503775.1| predicted protein [Micromonas sp. RCC299]gb|ACO65033.1| predicted protein [Micromonas sp. RCC299]	406	80.1 bits (196)	43/69 (62%)	1E-13
NEOS13_1509	3488	+	402	133	ref|XP_002509372.1| predicted protein [Micromonas sp. RCC299]gb|ACO70630.1| predicted protein [Micromonas sp. RCC299]	140	89.0 bits (219)	47/80 (58%)	2E-16
NEOS13_0001	1145	+	150	49	|No hits found|				
NEOS13_2799	775	-	234	77	ref|XP_002508629.1| hypothetical protein MICPUN_62214 [Micromonas sp. RCC299]gb|ACO69887.1| hypothetical protein MICPUN_62214 [Micromonas sp. RCC299]	1098	79.7 bits (195)	40/74 (54%)	1E-13
NEOS13_2805	2642	+	231	77	|No hits found|				
NEOS13_2806	1116	-	219	73	ref|XP_002503775.1| predicted protein [Micromonas sp. RCC299]gb|ACO65033.1| predicted protein [Micromonas sp. RCC299]	406	89.4 bits (220)	48/72 (66%)	2E-16
NEOS13_2807	2733	+	210	70	ref|XP_002507451.1| predicted protein [Micromonas sp. RCC299]gb|ACO68709.1| predicted protein [Micromonas sp. RCC299]	395	86.3 bits (212)	46/70 (65%)	2E-15
NEOS13_2808	1111	-	210	70	ref|XP_002503067.1| predicted protein [Micromonas sp. RCC299]gb|ACO64325.1| predicted protein [Micromonas sp. RCC299]	252	93.6 bits (231)	45/70 (64%)	9E-18
NEOS13_2809	1798	-	201	67	ref|YP_001869272.1| Miro domain-containing protein [Nostoc punctiforme PCC 73102]gb|ACC84329.1| Miro domain protein [Nostoc punctiforme PCC 73102]	1109	83.6 bits (205)	45/66 (68%)	8E-15
NEOS13_2810	1703	-	192	64	ref|XP_002505627.1| predicted protein [Micromonas sp. RCC299]gb|ACO66885.1| predicted protein [Micromonas sp. RCC299]	573	80.9 bits (198)	40/64 (62%)	6E-14
NEOS13_2811	501	+	189	63	ref|XP_002507358.1| predicted protein [Micromonas sp. RCC299]gb|ACO68616.1| predicted protein [Micromonas sp. RCC299]	574	83.2 bits (204)	42/62 (67%)	1E-14
NEOS13_2812	1551	-	183	61	ref|XP_002509372.1| predicted protein [Micromonas sp. RCC299]gb|ACO70630.1| predicted protein [Micromonas sp. RCC299]	140	78.2 bits (191)	39/60 (65%)	4E-13
NEOS13_2813	2456	-	171	57	ref|XP_002503067.1| predicted protein [Micromonas sp. RCC299]gb|ACO64325.1| predicted protein [Micromonas sp. RCC299]	252	73.9 bits (180)	36/57 (63%)	7E-12
NEOS13_2814	2575	-	168	56	ref|YP_001869272.1| Miro domain-containing protein [Nostoc punctiforme PCC 73102]gb|ACC84329.1| Miro domain protein [Nostoc punctiforme PCC 73102]	1109	73.2 bits (178)	38/56 (67%)	1E-11
NEOS13_2815	2486	+	168	56	|No hits found|				
NEOS13_2816	2732	+	165	55	ref|XP_002508688.1| predicted protein [Micromonas sp. RCC299]gb|ACO69946.1| predicted protein [Micromonas sp. RCC299]	518	77.4 bits (189)	41/55 (74%)	7E-13
NEOS13_2817	856	-	156	52	ref|XP_002505627.1| predicted protein [Micromonas sp. RCC299]gb|ACO66885.1| predicted protein [Micromonas sp. RCC299]	573	70.5 bits (171)	33/52 (63%)	9E-11
NEOS13_2818	3038	-	147	49	|No hits found|				
NEOS13_2819	803	+	138	46	|No hits found|				
NEOS13_2820	1712	+	132	44	|No hits found|				
NEOS13_2821	730	+	132	44	|No hits found|				
NEOS13_2822	1787	-	129	43	|No hits found|				
NEOS13_2823	2417	+	126	42	|No hits found|				
NEOS13_2824	1921	-	126	42	|No hits found|				
NEOS13_2825	1178	-	123	41	|No hits found|				
NEOS13_2826	2984	-	123	41	|No hits found|				
NEOS13_2827	1631	-	114	38	|No hits found|				
NEOS13_2828	1191	-	111	37	|No hits found|				

*BLASTP cutoff: 1.00E-10
